# USP25-driven KIFC1 regulates MYCBP expression and promotes the progression of cervical cancer

**DOI:** 10.1038/s41419-025-07713-x

**Published:** 2025-05-16

**Authors:** Zhoujie Ye, Liping Zhu, Yalan Wei, Aizhu Lin, Xiaolu Fan, Xiaojing Fan, Pengming Sun, Xinrui Wang

**Affiliations:** 1 Medical Research Center, Fujian Maternity and Child Health Hospital, College of Clinical Medicine for Obstetrics & Gynecology and Pediatrics, Fujian Medical University, Fuzhou, 350013 Fujian, China; 2 Fujian Key Laboratory of Women and Children’s Critical Diseases Research, Fujian Maternity and Child Health Hospital (Fujian Women and Children’s Hospital), Fuzhou, 350001 Fujian, China; 3 Laboratory of Gynecologic Oncology, Fujian Maternity and Child Health Hospital, College of Clinical Medicine for Obstetrics & Gynecology and Pediatrics, Fujian Medical University, Fuzhou, 350001 Fujian, China; 4 Fujian Clinical Research Center for Gynecological Oncology, Fujian Maternity and Child Health Hospital (Fujian Obstetrics and Gynecology Hospital), Fuzhou, 350001 Fujian, China

**Keywords:** Cervical cancer, Tumour biomarkers

## Abstract

Cervical cancer (CCa) continues to exhibit high mortality rates, compounded by the scarcity of effective therapeutic targets. This study highlights the significant upregulation of Kinesin Family Member C1 (KIFC1), a member of the kinesin-14 family, in CCa tissues. Elevated KIFC1 expression correlates with poorer prognoses in CCa patients. USP25, a deubiquitinating enzyme, stabilizes KIFC1 protein through deubiquitination, facilitating its accumulation in CCa tissues. Our in vitro and in vivo experiments demonstrate KIFC1’s pivotal role in enhancing tumorigenesis and metastasis of CCa cells. Furthermore, we discovered that KIFC1 expression variability could modulate the levels of MYCBP, a known binding partner of the oncogenic protein c-MYC, which influences tumorigenesis. The suppression of USP25 results in decreased KIFC1 and MYCBP protein levels, independent of mRNA changes. However, reintroducing KIFC1 into USP25-deficient cells restores MYCBP expression levels. Simultaneously, targeting USP25, KIFC1 and MYCBP disrupts the malignant phenotype of CCa cells. Collectively, our findings elucidate the previously unknown functions and mechanisms of the USP25/KIFC1/MYCBP signaling axis in CCa progression, underscoring KIFC1 as a promising therapeutic target for cervical cancer.

## Introduction

Cervical cancer (CCa) is the leading cause of cancer death among women in developing countries, with approximately 604,000 new cases and 342,000 deaths reported globally in 2020 [1]. It has emerged as the second most common cause of cancer-related deaths in women aged 20 to 39 [2]. High-risk human papillomavirus (hrHPV) infection is a predominant risk factor for CCa, contributing to over 90% of cases [3]. While cervical cancer vaccination programs and systematic screening have significantly reduced its incidence, they remain crucial in the clinical management of CCa patients. Treatment strategies are determined by the clinical stage [3, 4]: surgical resection is the primary treatment for early-stage disease, whereas tumor debulking combined with chemoradiation is typically employed for advanced stages or in patients who are not candidates for surgery. Platinum-based chemotherapy is the first-line treatment for CCa, but drug resistance, along with tumor recurrence and metastasis, continues to challenge effective management [5]. Thus, a deeper understanding of the molecular pathogenesis of CCa is essential for developing more efficacious treatment alternatives.

Kinesin Family Member C1 (KIFC1) functions as a motor protein, facilitating movement from the positive to the negative ends of microtubules. It enhances cross-linking, sliding, and binding among microtubules, playing a crucial role in the aggregation and assembly of spindle poles during mitosis [6]. KIFC1 can directly interact with CEP215, enhancing the linkage of centrosomes to spindle poles and improving centrosome cohesion [7]. Additionally, KIFC1 is implicated in chromosome alignment, organelle transport during the cell cycle [8]. For tumor cells exhibiting abnormalities in centrosome division, KIFC1 is essential for survival, particularly for cancer cells possessing supernumerary centrosomes [9]. Its overexpression triggers the upregulation of genes that regulate mitotic checkpoints, leading to aneuploidy and hastening cancer progression [10]. KIFC1 is markedly overexpressed in various malignancies, including breast [11], ovarian [12], liver [13], and gastric carcinoma [14], where it influences tumor development through diverse pathways. In cervical cancer, KIFC1 expression levels are significantly elevated compared to adjacent normal tissues, as observed in tumor cohort databases. However, the specific biological functions and underlying molecular mechanisms of KIFC1 in cervical cancer remain poorly understood.

This study is the first comprehensive analysis of KIFC1’s overexpression in CCa tissues, identifying it as a potential oncogene promoting CCa progression. The stabilization of KIFC1 protein is maintained through USP25-mediated deubiquitination, resulting in its accumulation in CCa tissues. Furthermore, KIFC1 regulates the transcription of MYCBP, accelerating CCa progression via the c-MYC protein pathway. These findings underscore the pivotal role of KIFC1 in advancing CCa and establish it as a novel therapeutic target for cervical cancer treatment.

## Materials and method

### Cell culture

The HEK293T cell line, and cervical cancer cell lines HeLa and SiHa, were sourced from Medical Research Center cell bank of Fujian Maternal and Child Health Hospital, with STR identification report and no mycoplasma contamination. HEK293T and HeLa cells were cultured in high-glucose DMEM medium supplemented with 10% FBS (Gibco, #6123139). SiHa cells were maintained in MEM medium containing 10% FBS (Gibco, #6123139) within a 5% CO2 incubator at 37 °C.

### Cell line construction

HeLa^*KIFC1-/-*^ and SiHa^*KIFC1-/-*^ cell lines were developed by employing CRISPR-Cas9 technology to knock out KIFC1 in HeLa and SiHa, the sgRNA sequence is shown in table S1. DNA constructs were transfected into HEK293T cells using TenyiBio DNA transfection reagent (#TF20120) along with a lentiviral packaging plasmid. 48 hours post-transfection, the supernatant containing the virus was collected to harvest KIFC1 overexpression lentivirus. After 24 hours of infection with the harvested virus in cervical cancer cells, the overexpression of KIFC1 or MYCBP were confirmed, and stable KIFC1-overexpressing(OE) or MYCBP-OE CCa cells were successfully established. Lentiviral constructs for knocking down MYCBP and USP25 (synthesized by Shanghai Gima Pharmaceutical Technology Co., Ltd.) were prepared, the shRNA sequence is shown in table S2. When cell density reached 40-60%, HeLa^*shMYCBP*^, SiHa^*shMYCBP*^, HeLa^*shUSP25*^, and SiHa^*shUSP25*^ cell lines were established after infection with lentivirus and selection with puromycin for one week.

### Tissue microarrys and IHC

The cervical cancer tissue chip was obtained from Shanghai Outdo Biotech. Cervical cancer tissue samples provide detailed clinical information, including sex, age, stage and histological type. Follow-up data of 99 patients, a total of 99 patients were included in the correlation analysis, 99 patients were included in the survival analysis, including 30 deaths, 69 survival cases. The EnVision* detection system(Dako) was used per the manufacturer’s instructions. lmmunostained microarrays were scored by multi-plying the intensity (0-3) and extent (0-100) of staining for each tissue points as previously described by Bollag et al. [15]. Three pre-experiments were performed to optimize antibody concentrations, and only one formal experiment was performed. All studies involving human samples were carried out in accordance with the Code of the World Medical Association (Declaration of Helsinki). Before the experiment, all the tissue chips were approved by the Ethics Committee of Shanghai Outdo Biotech Co. Ltd. (No. SHYJS-CP-1801024).

### IHC and expression statistics

Image-Pro Plus version 6.0 software (Media Cybernetics, Inc., Rockville, MD, USA) was used to assess the area and integrated optical density (IOD) density of the chromogenic region. An identical setting was used for all images. The mean density of target protein in each image was calculated as IOD/total area of each image.

### RNA extraction, RT-PCR and RT-qPCR

Total RNA was extracted using the Vazyme Rapid Cell Total RNA Extraction Kit (Vazyme, #7E781J3). Complementary DNA (cDNA) was synthesized using Prime™ RT Master Mix (Takara, #RR036A). Routine reverse transcription-PCR (RT-PCR) was conducted with the 2×Hieff® Robust PCR Master Mix (Yeasen, #10106ES08). PCR products underwent DNA gel electrophoresis and were visualized with YeaRed Nucleic Acid Gel Staining (Yeasen, #10202ES76) using a Chemical Imager 5500 system. Real-time fluorescence quantitative PCR (RT-qPCR) was performed to amplify cDNA using SYBR mix (Vazyme, #Q711-03). Results were analyzed using the 2^(-ΔΔCT) method. Primer sequences for RT-PCR and RT-qPCR are detailed in supplemental tables S3 and S4. We repeated the experiment three times in total.

### Immunoblotting and immunoprecipitation

For immunoblotting, proteins were extracted using NP-40 buffer supplemented with protease and phosphatase inhibitors (Thermo, #78443). Protein concentrations were determined using a BCA Protein Quantification Kit (Thermo, #NCI3225CH). An equal amount of proteins was subjected to SDS-PAGE and transferred onto PVDF membranes (Millipore, #IPVH00010). Membranes were blocked using 5% skim milk powder (ABclonal, #RM00014) and incubated with specific primary antibodies and corresponding secondary antibodies. Visualization was achieved using an Enhanced Chemiluminescence (ECL) kit (Tanon, #180-5001E). The primary antibodies used included anti-KIFC1 (ABclonal, #A3304), anti-USP25 (ABclonal, #A23431), anti-MYCBP (ABclonal, #A4623), anti-GAPDH (ABclonal, #A19056), anti-GLUT1 (ABclonal, #A11208), anti-Hexokinase 2 (ABclonal, #A22319), anti-LDH-A (ABclonal, #A21893), anti-actin (ABclonal, #RP02968LQ), anti-Flag (ABclonal, #AE121), anti-His (ABclonal, #AE086), anti-HA (ABclonal, #AE105), anti-GST (ABclonal, #AE077) and HRP-conjugated Digoxin Rabbit mAb (ABclonal#A24114).

For immunoprecipitation assays, cells were lysed in NP-40 buffer containing protease and phosphatase inhibitors. Proteins were immunoprecipitated overnight at 4 °C using anti-Flag affinity gel (Bimake, #B23102). The immunoprecipitates were washed three times with NP-40 lysis buffer and subsequently analyzed by immunoblotting. Image J 6.0 software was used to analyze the gray value of the results. We repeated the experiment three times in total.

### Glutathione S-transferase (GST) pull-down assay

The pGEX-4T-3 and pET28a vectors were used for the expression of GST and His tagged proteins, respectively. For protein pull-down assay, GST, GST-USP25 and His-KIFC1 plasmids were co-transformed into E. coli BL21 (DE3). After overnight expression induced by 100 μM IPTG at 20 °C, cell cultures were collected in GST binding buffer (50 mM Tris-HCl, pH 8.0, and 150 mM NaCl), and then broken by the supersonic method in the lysis buffer (50 mM Tris-HCl, pH 7.4, 150 mM NaCl, 1 mM EDTA and 1% Triton X-100). Bacterial lysate was incubated with 50 μL of Glutathione Sepharose 4B beads at 4 °C for 3 h, then the supernatant was removed and beads were washed thoroughly with ice-cold PBS buffer for 3 times. The proteins bound to the beads were released with 80 μl of SDS sample loading buffer and boiled for 10 min, then anti-His (CWBIO, CW0285, 1:5,000 dilutionand anti-GST (CWBIO, CW0084,1:5,000 dilution) antibodies were used for immunoblot analyses.

### Immunoprecipitation-mass spectrometry (IP-MS)

The dried peptides were reconstituted in solvent A (0.1% formic acid in water) and analyzed using an Orbitrap Exploris 480 mass spectrometer coupled to an UltiMate 3000RSLC nano system (Thermo Fisher Scientific, MA, USA). A 3 μL peptide sample was loaded onto a 25 cm analytical column (75 μm inner diameter, 1.9 μm resin from Dr Maisch) and eluted with a 90-minute gradient starting at 5% buffer B (80% acetonitrile with 0.1% formic acid). The gradient increased stepwise to 99% over 83 minutes, held constant for 5 minutes. The flow rate was maintained at 300 nL/min, and the column temperature was set at 50 °C. Raw data were analyzed using Proteome Discoverer 2.4 (PD, version 2.4) and searched against the reviewed SwissProt human proteome database.

### Cell proliferation and colony formation experiments

For cell proliferation detection, cells in the logarithmic growth phase (5 × 10^3^) were seeded into 96-well plates, three replicate wells per condition. The CCK-8 assay (Dojindo, #HY-K0301) was employed, with the working solution added at the same time each day and incubated for 2 hours. Optical density (OD) at 450 nm was measured using a BioTek Cytation1 enzyme-labeled instrument (USA), and proliferation curves were plotted. For colony formation assays, cells (1 × 10^3^) were plated in 6-well plates, with the procedure repeated three times. After two weeks of culture, colonies were fixed with tissue fixative for 20 minutes and stained with 0.1% crystal violet (Solarbio, #C8470). We repeated the experiment three times in total.

### Transwell migration and invasion assays

Cells suspended in 200 μL of 5% serum medium were placed into a transfer chamber (Corning Incorporated, #08722022) for migration tests or into a chamber pre-coated with Matrigel for invasion assays. A 600 μL aliquot of 20% serum medium was added to the lower chamber. After 24 hours, cells were fixed with methanol and stained with crystal violet. Positive cells were imaged under a microscope in five independent fields and quantified using Image J software. We repeated the experiment three times in total.

### Cell cycle and apoptosis analysis

For cell cycle analysis, cells (1 × 10^6^) were fixed overnight with 80% ethanol, centrifuged at 2000rpm for 5 mins, and then stained with RNase A and 50 µg/ml propidium iodide (PI) (Elabscience, China, #E-CK-A351) for 30 minutes. Flow cytometry analysis was performed using a BD LSRFortessa or Agilent NovoCyte. For apoptosis assays, cells (1 × 10^6^) were washed with pre-cooled PBS, resuspended in 100 µl of binding buffer (1% BSA in PBS), and stained with Annexin V-FITC/PI (Elabscience, China, #E-CK-A211), One-step TUNEL apoptosis kit (Elabscience, China, #E-CK-A320), or Annexin V-APC/7-AAD apoptosis kit (Elabscience, China, #E-K-A218). Cells were incubated at room temperature for 15 minutes away from light and analyzed by flow cytometry (BD LSRFortessa or Agilent NovoCyte). Early and late apoptosis phases were assessed. We repeated the experiment three times in total.

### Xenografts model

BALB/c-nu mice (6-8 weeks, female, body weight: 20.0 ± 2.0 g) were obtained from SLAC Laboratory Animal Co., Ltd. (Shanghai, China). BALB/c-nu mice were randomly divided into groups of 5 animals per cage, and tumor formation experiments were conducted in the specific pathogen free (SPF) animal house. Before the experiment, we had no specific understanding of the background factors (such as age, size, sex, etc.) and environmental factors (such as temperature, altitude, etc.) of the animals. Cells were harvested and resuspended at a density of 2 × 10^7^/ml in a 1:1 mixture of matrix glue and phosphate buffer. A 100 µl volume of this suspension was subcutaneously injected into the right forearm of each nude mouse. Tumor volume was measured every 3 days. Upon reaching approximately 1000 mm^3^, tumors were collected post-euthanasia by cervical dislocation under isopentane anesthesia. The tumors were then dissected and weighed. All protocols involving animals were approved by the Animal Welfare and Ethics Committee of Fujian Maternal and Child Health Hospital (Approval Number: 2021DK005) and adhered to ethical standards for animal husbandry. We conducted only one experiment, with five tumor animals per component.

### Immunohistochemistry

Paraffin-embedded tissue slides were deparaffinized and hydrated using standard methods. Antigen retrieval was performed using citrate buffer (pH 6.0), followed by a 15-minute incubation in 3% H_2_O_2_ and three 5-minute washes in Tris-HCl buffer with 1% Tween-20 (TBST). Slides were blocked with normal goat serum (Boster, Wuhan, China) for 60 minutes before overnight incubation with primary antibodies at 4 °C. After washing thrice in TBST (15 minutes each), slides were incubated with secondary goat anti-rabbit antibody (Maixin, Fujian, China) for 60 minutes at 37 °C. Visualization was achieved using 3,3′-diaminobenzidine (DAB) (Fuzhou Maixin, China) for 5 minutes and counterstaining with hematoxylin for 45 seconds. Slides were mounted and imaged using a Keyence microscope (USA). Three tumor tissues of different mouse origin were randomly selected for immunohistochemistry in each group.

### Immunofluorescence microscopy

Cells were seeded in 24-well Cell Carrier plates (PerkinElmer Life Sciences) and, after 24 hours, fixed with paraformaldehyde and permeabilized using Triton X-100. They were then blocked and permeabilized in a humidity chamber for 1 hour. Following three washes with PBS, the cells were incubated overnight at 4 °C with primary antibodies. Subsequently, cells were washed three times with PBS and incubated with secondary antibodies for 2 hours at room temperature. After another three washes, cells were stained with 1 µg/ml DAPI for 10 minutes. Images were captured using a Leica TCSSP8 microscope (Germany) and analyzed using Columbus software. We repeated the experiment three times in total.

### Cell metabolism determination

Cellular metabolism, including the extracellular acidification rate (ECAR) and ATP rate, was analyzed using a Seahorse XFe24 Extracellular Flux Analyzer (Agilent Technologies, Santa Clara, CA, USA). Cervical cancer cells (2×10^4^) were seeded into XF 24-well plates 24 hours prior to the assay. Cells were then washed and incubated in Seahorse analysis media in a CO2-free incubator at 37 °C for 1 hour. ECAR was assessed using a Glycolysis Stress Test Kit (Agilent) by sequentially injecting glucose (10 mM), oligomycin (1 mM), and 2-deoxy-D-glucose (2-DG) (100 mM) at specified time points. ATP rates were determined using the Real-Time ATP Rate Test Kit (Agilent) with sequential injections of oligomycin (1.5 mM) and a mixture of rotenone and antimycin A (0.5 mM). Both ECAR and ATP values were normalized to the cell count. Data analysis was performed using Seahorse XFe24 Wave software. We repeated the experiment three times in total.

### Statistical analysis

Most experiments were conducted with three biological replicates. The data from multiple experiments were analyzed using GraphPad Prism 9 and are presented as the mean ± standard deviation (SD). The significance analysis was conducted by Student’s unpaired two-sided t-test. When *p* value < 0.05, it was considered that there were significant differences between the groups.

## Results

### KIFC1 overexpressed in CCa and is a poor prognostic marker or CCa patients

**Fig. 1:**
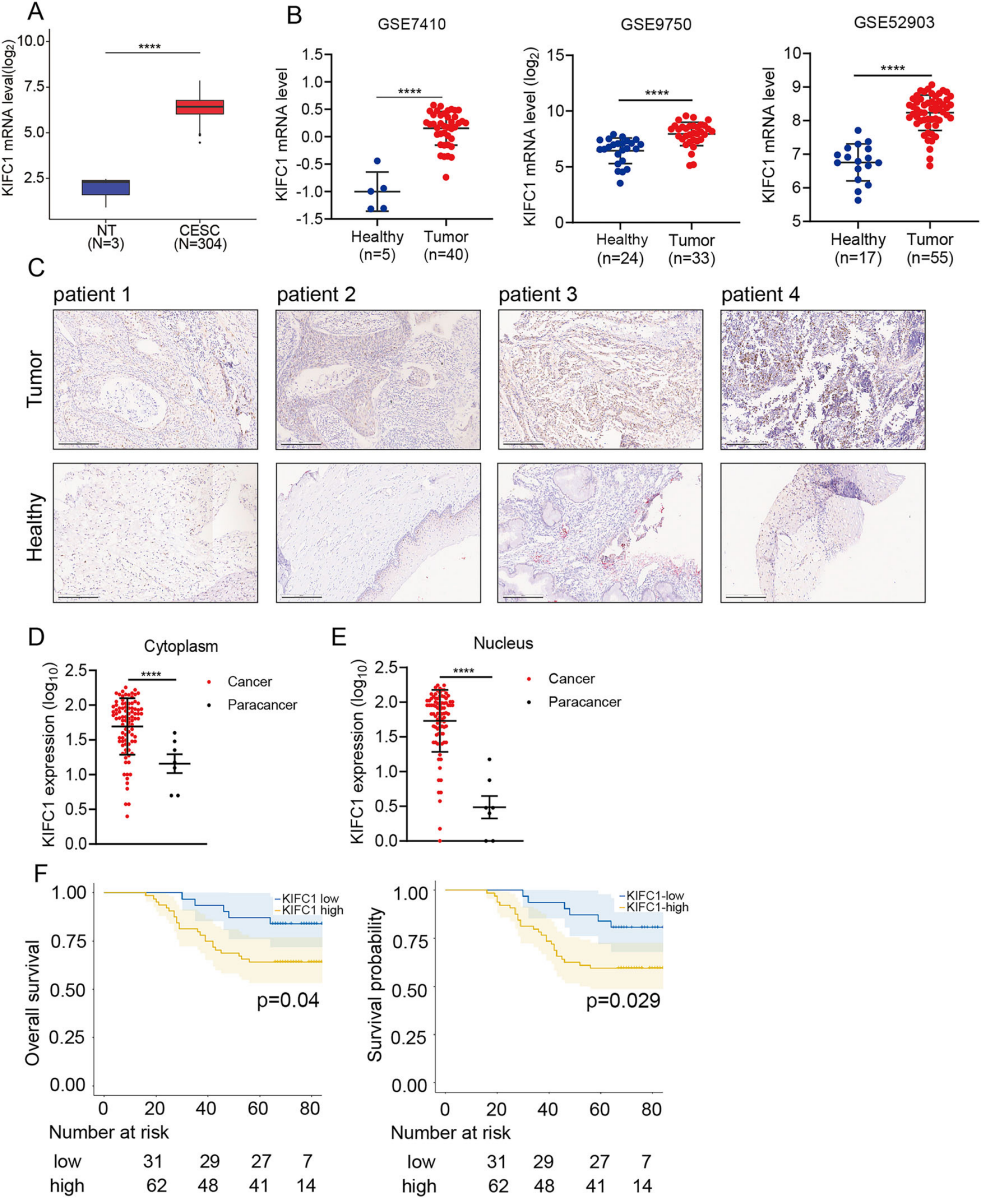
KIFC1 is overexpressed in CCa tissues and is associated with poor disease prognosis. **A** Analysis of KIFC1 mRNA expression levels in cervical cancer using TCGA dataset. **B** The expression of KIFC1 mRNA in cervical cancer was analyzed with different datasets of GEO database. **C** The expression of KIFC1 protein in tumor tissues of CCa patients was detected by immunohistochemistry. Scale bar, 75 μm. **D**, **E** The expression levels of KIFC1 in the nucleus and cytoplasm were analyzed statistically. **F** Prognostic analysis of overall survival and survival probability of CCa patients with KIFC1 protein expression in nucleus. *p < 0.05, **p < 0.001, ***p < 0.001, ****p < 0.0001 by Student’s unpaired two-sided t-test.

Microtubule motor proteins plays a vital role in the dynamic changes of microtubules and intracellular material transport. Recent studies increasingly highlight the involvement of tubulin in tumors, influencing tumorigenesis through various signaling pathways [16***–***18]. This research utilizes tumor cohort databases to investigate KIFC1 expression disparities, KIFC1 mRNA was found to be significantly upregulated in CCa tissues compared to adjacent non-tumor tissues (Fig. 1A). Additionally, gene chip data from the Gene Expression Omnibus (GEO) confirmed higher KIFC1 expression in CCa tissues than in normal tissues (Fig. 1B).

Our cohort study involved a tissue microarray analysis of 99 CCa tumor samples and 12 adjacent normal tissues (Supplementary Fig. 1). Immunohistochemistry identified variable KIFC1 expression in CCa and normal tissues (Fig. 1C). Notably, both cytoplasmic and nuclear KIFC1 levels were markedly elevated in CCa tumor cells (Fig. 1D, E). A lower nuclear expression of KIFC1 was associated with significantly improved overall survival and survival probability, contrasting with the poor overall survival linked to high KIFC1 expression (Fig. 1F). High expression of KIFC1 protein is associated with poor overall survival (OS) in patients with cervical cancer. These findings collectively underscore KIFC1’s upregulation in CCa tissues, suggesting its potential as a valuable prognostic marker for CCa patients.

### KIFC1-deletion inhibits CCa progression in vitro and in vivo

**Fig. 2:**
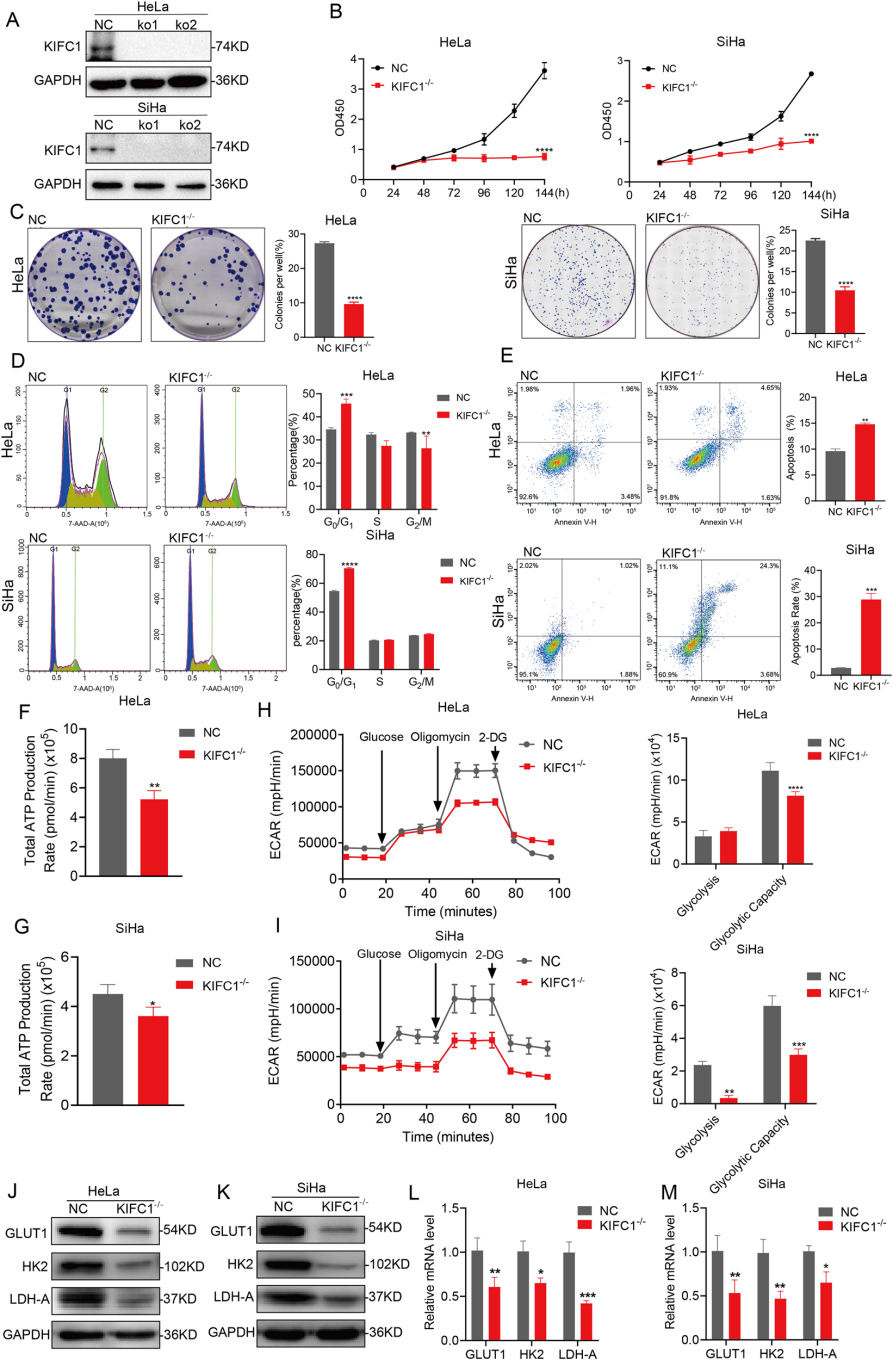
KIFC1 knockout inhibited CCa cells proliferation and energy metabolism in vitro. **A** Immunoblotting analyses the expression of KIFC1 protein knocked out in CCa cells. CCK-8 (**B**) and colony formation (**C**) assays were performed using KIFC1 knockout CCa cells. Representative images of colonies and relevant quantification are displayed in (**C**), respectively. PI cell cycle (**D**) and Annexin V/FITC apoptosis detection (**E**) analysis of KIFC1 knockout CCa cells by flow cytometry, representative images and statistical maps were presented simultaneously. **F**, **G** The total ATP production rate in CCa cells was measured by cell energy metabolism. **H**, **I** Analyze the rate and corresponding statistical extracellular acidification rate (ECAR) of KIFC1 knockout CCa cells in aerobic glycolysis. **J**–**M** CCa cells knocked out KIFC1 or NC (negative control) were subjected to various analyses to measure the expression levels of GLUT1, HK2 and LDH-A involved in glucose metabolism by Immunoblotting (**J**, **K**) and real-time PCR (**L**–**M**). *p < 0.05, **p < 0.001, ***p < 0.001, ****p < 0.0001 by Student’s unpaired two-sided t-test.

To elucidate the biological function of KIFC1 in CCa, we initially assessed KIFC1 protein expression levels in normal cervical epithelial cells (HCerEpic) and seven CCa cell lines (Supplementary Fig. 2A, B). We specifically focused on two human cervical cancer cell lines, HeLa (adenocarcinoma) and SiHa (squamous carcinoma), which exhibited the highest KIFC1 expression. For the convenience of subsequent discussion, we will collectively refer to HeLa and SiHa as CCa cells. Immunoblotting analysis confirmed successful KIFC1 knockout in these cell lines (Fig. 2A). Cell viability assays, including CCK-8 and colony formation tests, revealed that KIFC1 knockout significantly reduced proliferation (Fig. 2B) and colony formation capabilities (Fig. 2C) in CCa cells compared to the empty vector control. Further analysis of cell cycle and apoptosis post-KIFC1 deletion showed cell cycle arrest at the G_0_/G_1_ phase (Fig. 2D) and an increase in late-stage apoptosis rates in CCa cells (Fig. 2E). The Warburg effect which is a recognized cancer hallmark, characterized by cancer cells’ predominant use of glycolysis over oxidative phosphorylation for energy production regardless of oxygen availability [19]. Our findings suggest a close association between cervical cancer progression and glycolysis. Seahorse analysis demonstrated showed that the total ATP production in CCa cells decreased after KIFC1 knockout (Fig. 2F, G). Seahorse analysis also indicated a decreased extracellular acidification rate (ECAR) in KIFC1 knockout CCa cells (Fig. 2H, I). Specifically, KIFC1 knockout reduced the expression levels of several glycolytic genes, including GLUT1, HK2 and LDH-A (Fig. 4J-M).

**Fig. 3:**
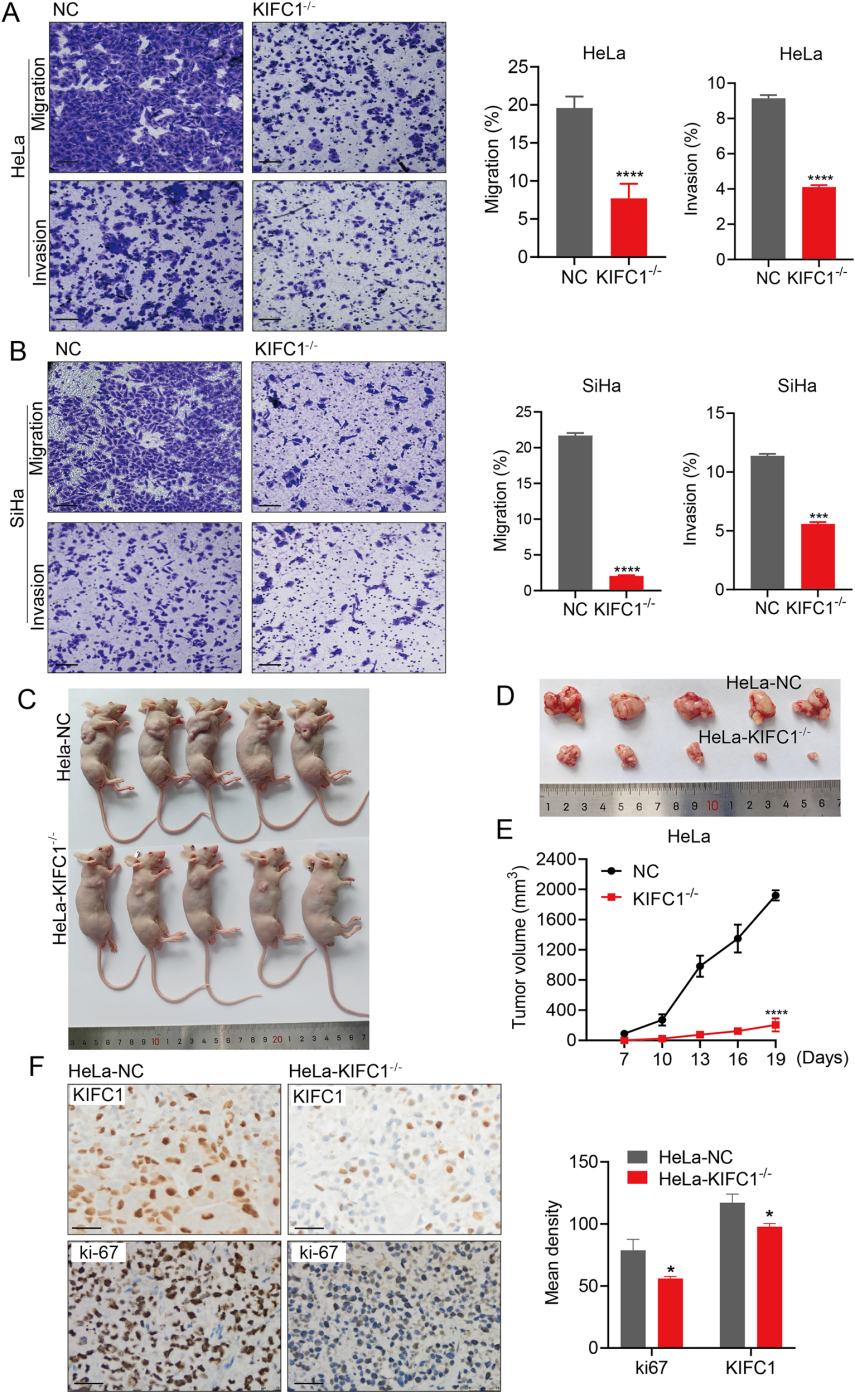
Knocking out KIFC1 impeded the ability of CCa to invade and metastasize in vitro and tumorigenesis in vivo. **A**, **B** Transwell migration and invasion assays were conducted with KIFC1-knocked out CCa cells. Representative images are shown in (**A**) and (**B**). The quantitative results of migrated or invaded cells are displayed in (**A**) and (**B**), respectively. Scale bar, 200 μm. **C**, **F** HeLa and HeLa^*KIFC1-/-*^ cells were injected subcutaneously into nude mice (*n* = 5) to construct tumor xenotransplantation model, the tumor volume was measured every 3 days when the tumor reached a certain size. On the 19th day, all mice were killed and underwent immunohistochemical staining. The size of the tumor was shown in (**C**, **D**), and the growth time of the xenograft was shown in (**E**). Immunohistochemical detection was performed to detect KIFC1 and ki-67 protein expression in the tumor tissue (**F**). Scale bar, 75 μm. *p < 0.05, **p < 0.001, ***p < 0.001, ****p < 0.0001 by Student’s unpaired two-sided t-test.

Given the highly invasive nature and elevated risk of distant metastasis associated with CCa, we investigated the influence of KIFC1 on the migratory and invasive capabilities of CCa cells. Our findings reveal that KIFC1 knockout markedly reduced both migration and invasion abilities of CCa cells (Fig. 3A, B). To further assess the role of KIFC1 in vivo, HeLa^*KIFC1-/-*^ cells and control cells were subcutaneously inoculated into nude mice. The results corroborated our in vitro findings, demonstrating that KIFC1 deletion significantly impeded the growth of xenograft tumors (Fig. 3C-E). Additionally, immunohistochemical analysis indicated a substantial reduction in ki-67 protein expression in the xenograft tumors, signifying a decrease in tumor cell proliferation and a reduction in malignancy (Fig. 3F). These results underscore the pivotal role of KIFC1 in facilitating CCa progression, highlighting its potential as a therapeutic target for curtailing the invasive and metastatic traits of cervical cancer.

### Exogenous expression of KIFC1 promotes malignant proliferation of CCa cells

Upon knocking out KIFC1 in CCa cells, both in vitro and in vivo progression of CCa was significantly halted. To determine whether reintroducing KIFC1 could reverse these effects, we employed a pLVX vector to achieve stable overexpression of KIFC1 protein in HeLa^*KIFC1-/-*^ and SiHa^*KIFC1-/-*^ cells. Immunoblotting analysis confirmed successful KIFC1 overexpression (Supplementary Fig. 3A). Subsequent assays, including CCK-8 and colony formation, indicated that exogenous KIFC1 expression notably enhanced CCa cells proliferation compared to the empty vector control (Supplementary Fig. 3B) and augmented colony-forming capabilities (Supplementary Fig. 3C). Further, overexpression of KIFC1 accelerated cell cycle progression and significantly reduced apoptosis rates (Supplementary Fig. 3D, E), thereby boosting overall cell proliferation. Moreover, while KIFC1 knockout initially suppressed the invasive and metastatic potential of CCa cells, reintroducing KIFC1 in these knockout cells markedly restored and enhanced their invasive and metastatic capacities compared to controls (Supplementary Fig. 3F, G). Collectively, these results highlight KIFC1’s role as a potent oncogene, underscoring its influence on accelerating CCa progression.

### USP25 is a key deubiquitinase (DUBs) that regulates KIFC1 protein stability

**Fig. 4:**
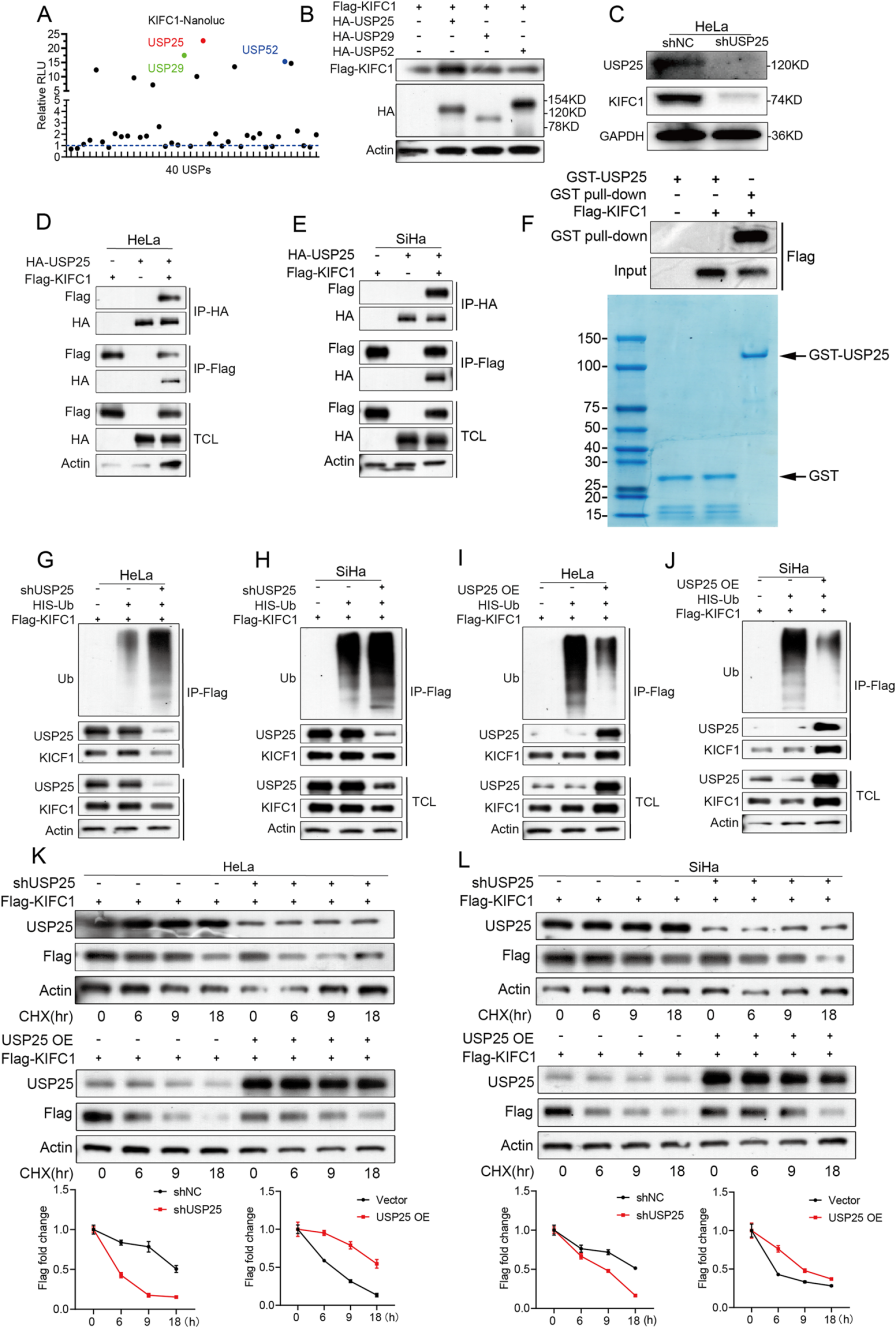
USP25 deubiquitination maintains the stability of KIFC1 protein. **A** Nanoluc luciferase assay for real-time dynamic monitoring of protein-protein interactions in HeLa cells. **B** Immunoblotting analyzed the effects of the deubiquitinating enzymes USP25, USP29 and USP252 proteins on the stability of KIFC1 protein in HeLa cells. In order to facilitate the detection of the HA tag carried by the deubiquitinating proteins and the Flag tag carried by KIFC1. **C** HeLa cell inhibition *USP25* immunoblotting analysis of USP25 and KIFC1 protein expression. **D**, **E** HA-USP25 and Flag-KIFC1 were exogenous expressed in CCa cells, and their interaction was detected by immunoprecipitation. **F** GST pull-down displays the direct interaction between KIFC1 and USP25. **G**, **H** Immunoblotting analysis of ubiquitinated Flag-KIFC1 after immunoprecipitation by Flag-protein beads in USP25-depelted CCa cells transfected with indicated constructs. **I**, **J** Immunoblotting analysis of ubiquitinated Flag-KIFC1 after immunoprecipitation by Flag-protein beads in USP25-overexpressied CCa cells transfected with indicated constructs. **K**, **L** The effect of USP25 on the half-life of exogenous Flag-KIFC1 was analyzed in CCa cells treated with 20 μg/mL cycloheximide(CHX), and the gray value of Flag-KIFC1 protein expression during CHX incubation time in (**K**) and (**L**).

Regulation of protein abundance is crucial for normal cellular functions, with all cellular proteins undergoing continuous degradation and replacement [20]. Given that variations in KIFC1 gene expression in CCa cells contribute to tumor progression, and KIFC1 is highly expressed in CCa tumors, it is vita nanoluc and KIFC1 proteins to analyze the interaction with 40 genes from the USP family of deubiquitinating enzymes. USP25, USP29, and USP52 exhibited the highest relative chemiluminescence intensities when incubated with KIFC1 protein, suggesting significant binding interactions (Fig. 4A and Supplementary Fig. 4).

To determine if these USP proteins could deubiquitinate and stabilize KIFC1 in vivo, we transfected HA-tagged USP25, USP29, and USP52 into HeLa cells expressing Flag-tagged KIFC1. Notably, the presence of HA-USP25 correlated with the highest expression levels of KIFC1 protein (Fig. 4B). Conversely, KIFC1 protein expression was significantly reduced following USP25 knockdown (Fig. 4C). To confirm the direct interaction and consequent deubiquitination of KIFC1 by USP25, FLAG-KIFC1 and HA-USP25 were co-expressed in CCa cells. Co-immunoprecipitation assays demonstrated a interaction between KIFC1 and USP25 proteins in both HeLa and SiHa cells (Fig. 4D, E), GST pull-down indicates that the USP25 can directly interact with the KIFC1(Fig. 4F).

To verify USP25 influences KIFC1 stability by mediating its deubiquitination, we assessed the interaction between KIFC1 protein and ubiquitin in cervical cancer (CCa) cells. Following the exogenous expression of Flag-KIFC1 in CCa cells and subsequent immunoprecipitation, silencing of the USP25 gene resulted in an increased association of ubiquitin with KIFC1 protein, as evidenced by a higher ubiquitin-bound KIFC1 content (Fig. 4G, H). Conversely, overexpression of USP25 led to a reduction in the ubiquitin molecules interacting with KIFC1 compared to the control group, alongside a significant increase in KIFC1 protein levels (Fig. 4I, J). To further substantiate USP25’s effect on KIFC1 protein stability, we employed cycloheximide (CHX) chase assays to monitor changes in the half-life of KIFC1. Upon knockdown of USP25 in CCa cells, the half-life of KIFC1 protein was significantly reduced. In contrast, overexpression of USP25 substantially extended the half-life of KIFC1 protein (Fig. 4K, L).

These results collectively reinforce the pivotal role of USP25 in modulating the stability of KIFC1 protein through deubiquitination, offering valuable insights into the regulatory mechanisms of protein stability in cancer progression.

### USP25-depleted inhibits CCa progression in vitro and in vivo

**Fig. 5:**
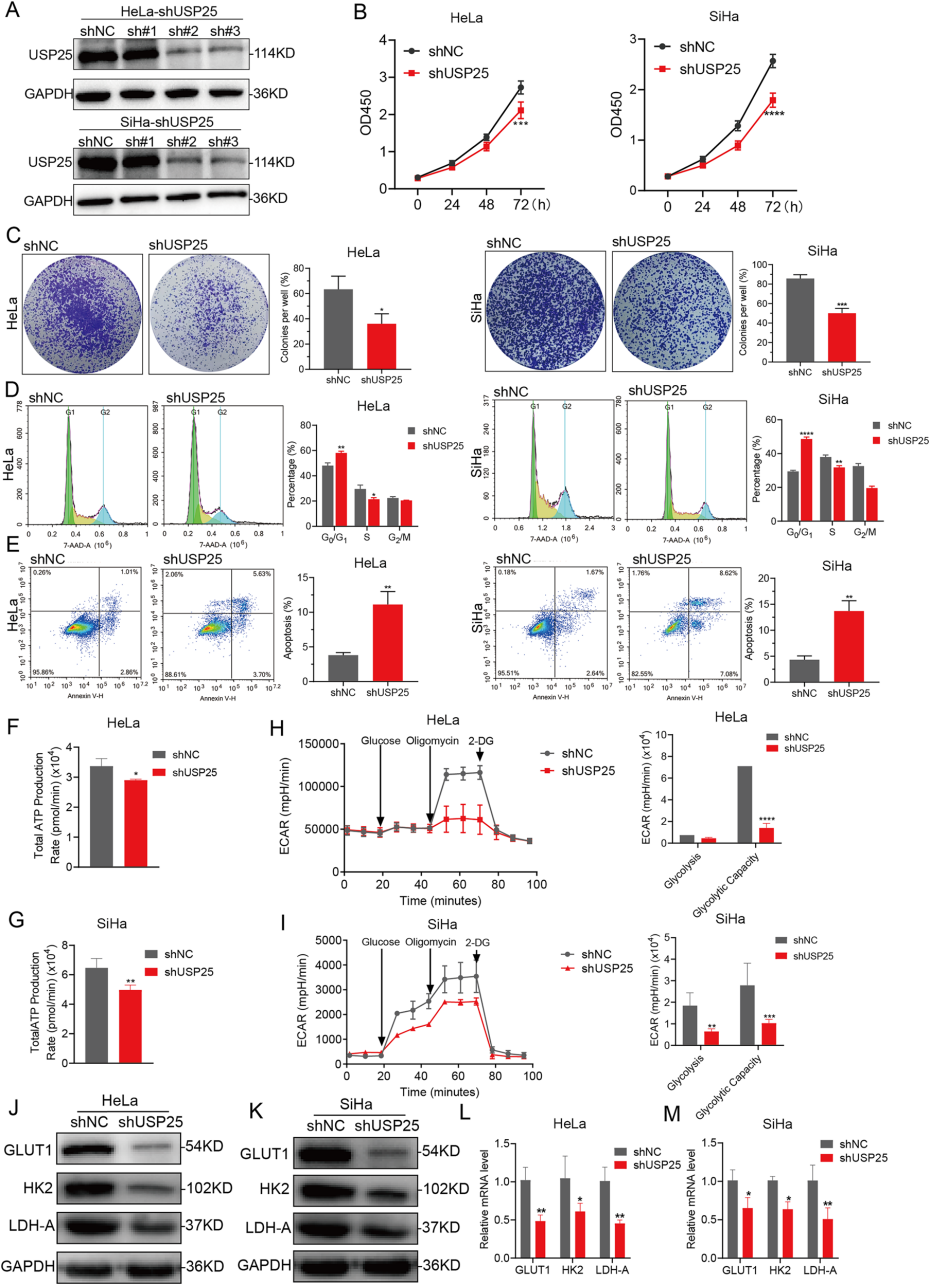
USP25 knockdown inhibited CCa cells proliferation and energy metabolism in vitro. **A** USP25 expression was down-regulated at different RNA interference sites by immunoblot in CCa cells. CCK-8 (**B**) and colony formation (**C**) assays were performed using USP25-depleted CCa cells. Representative images of colonies and relevant quantification are displayed in (**C**), respectively. **D**, **E** PI cell cycle (**D**) and Annexin V/FITC apoptosis detection (**E**) analysis of USP25-depleted CCa cells by flow cytometry, representative images and statistical maps were presented simultaneously. **F**, **G** The total ATP production rate in USP25-depleted CCa cells was measured by cell energy metabolism. **H**, **I** Analyze the rate and corresponding statistical rate of extracellular acidification rate (ECAR) of USP25-depleted CCa cells in aerobic glycolysis. **J**–**M** CCa cells infected with either control shRNA or USP25 shRNA were subjected to various analyses to measure the expression levels of GLUT1, HK2 and LDH-A involved in glucose metabolism by Immunoblotting (**J**, **K**) and real-time PCR (**L**, **M**). *p < 0.05, **p < 0.001, ***p < 0.001, ****p < 0.0001 by Student’s unpaired two-sided t-test.

The dysfunction of ubiquitinating and deubiquitinating enzymes is prevalent in various cancers, underscoring the critical need for comprehensive studies on ubiquitin ligases and deubiquitinating enzymes (DUBs) [21]. To delve deeper into USP25’s impact on CCa, we initially suppressed USP25 expression in CCa cells (Fig. 5A). Given that sh#2 exhibited the lowest USP25 expression, it was chosen for further analyses. Suppression of USP25 significantly hindered CCa cell proliferation (Fig. 5B) and colony formation (Fig. 5C). Compared to the control, USP25 knockdown arrested CCa cells in the G0/G1 phase, concurrently reducing the proportion of cells in the S-phase (Fig. 5D). Moreover, late apoptosis was substantially increased in shUSP25 cells (Fig. 5E). The metabolic profile of CCa cells was altered following USP25 inhibition, there was a notable decrease in total intracellular ATP production (Fig. 5F, G) and a reduction in the extracellular acidification rate (ECAR) (Fig. 5H, I). USP25-depleted reduced the expression levels of several glycolytic genes, including GLUT1, HK2 and LDH-A (Fig. 5J-M).

Additionally, the invasion and metastatic capabilities of CCa cells were significantly curtailed post-USP25 knockdown (Supplementary Fig. 5A, B). In vivo experiments using HeLa^*shUSP25*^ cells subcutaneously inoculated into nude mice further confirmed these findings. Tumor growth was markedly reduced in HeLa-shUSP25 compared to controls (Supplementary Fig. 5C, D). Immunohistochemical analysis revealed a significant decrease in ki-67 protein expression in tumor tissues from shUSP25 cells, indicating diminished malignant proliferation (Supplementary Fig. 5E). These results demonstrate that USP25 plays a critical role in fostering the tumorigenicity of CCa, both in vitro and in vivo.

### UPS25 maintains the stability of KIFC1 protein and promotes the progression of CCa malignant phenotype

**Fig. 6:**
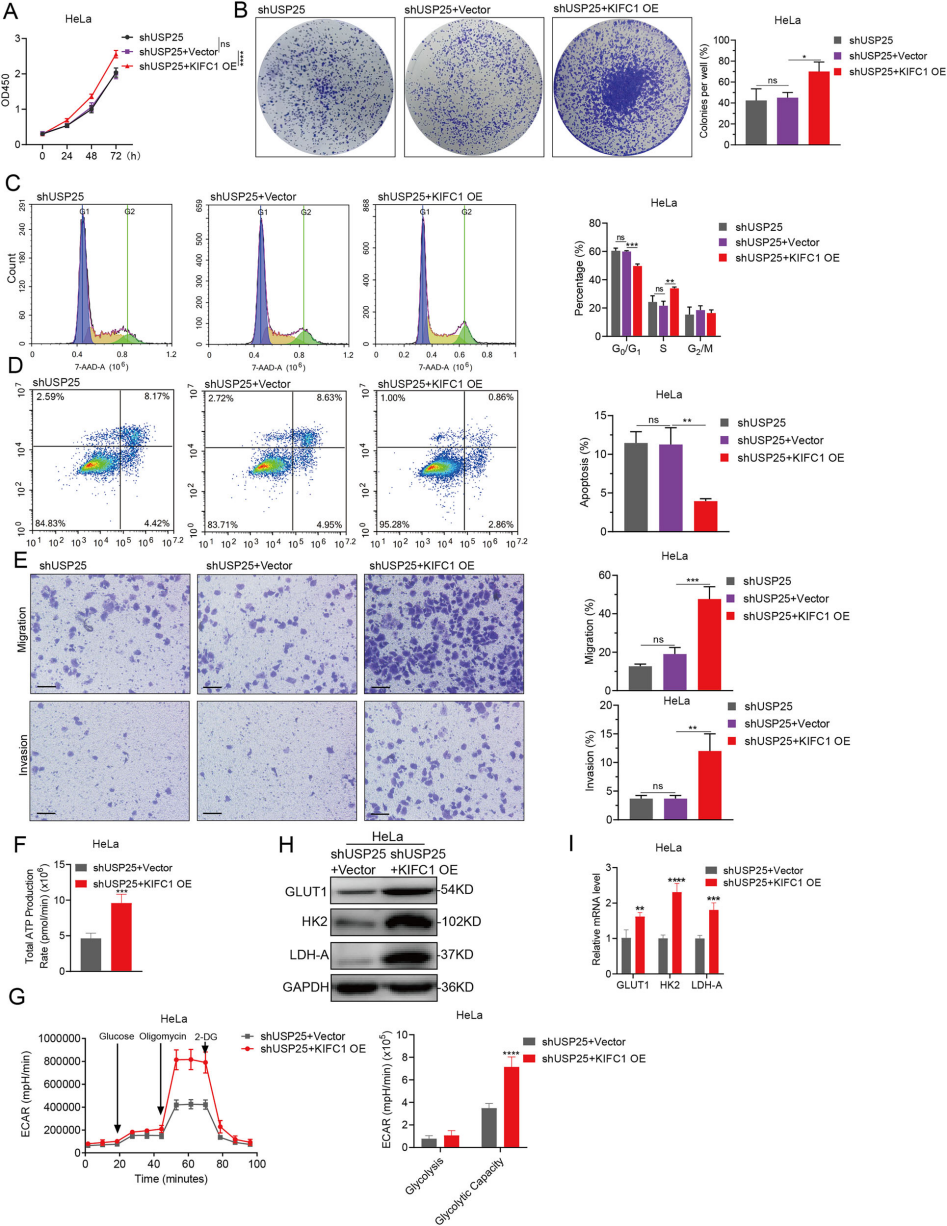
Reintroduction of KIFC1 rescues malignant progression of CCa cells from being hindered by depleted USP25. CCK-8 (**A**) and colony formation (**B**) assays were performed using KIFC1-overexpressing HeLa^*shUSP25*^ cells. Representative images of colonies and relevant quantification are displayed in (**B**), respectively. **C**, **D** The cell cycle (**C**) and apoptosis (**D**) of KIFC1-overexpressing HeLa^*shUSP25*^ cells were detected by flow cytometry, the corresponding data statistics are shown in the figure. **E** Transwell migration and invasion assays were conducted with KIFC1-overexpressing HeLa^*shUSP25*^ cells. Representative images and the quantitative results of migrated or invaded cells are displayed in (**E**), respectively. Scale bar, 200 μm. **F**, **G** Total ATP production of KIFC1-overexpressing HeLa^*shUSP25*^ cells (**F**) and extracellular acidification rate (ECAR) of aerobic glycolysis cells were measured by energy metabolism. **H**, **I** HeLa^*shUSP25 +Vector*^ and HeLa^*shUSP25 +KIFC1 OE*^ cells were subjected to various analyses to measure the expression levels of genes involved in glucose metabolism by Immunoblotting (**H**) and real-time PCR (**I**). *p < 0.05, **p < 0.001, ***p < 0.001, ****p < 0.0001 by Student’s unpaired two-sided t-test.

To explore whether USP25 facilitates the progression of cervical cancer (CCa) through modulating KIFC1, we investigated the effects of KIFC1 overexpression in CCa cells with suppressed USP25 expression. Notably, overexpression of KIFC1 significantly enhanced cell proliferation (Fig. 6A and Supplementary Fig. 6A) and colony formation (Fig. 6B and Supplementary Fig. 6B), compared to the CCa cells with only USP25 knockdown. Additionally, KIFC1 overexpression increased the proportion of S-phase cells (Fig. 6C and Supplementary Fig. 6C) and notably reduced apoptosis rates (Fig. 6D and Supplementary Fig. 6D) compared with control groups. This modification notably restored the invasion and metastasis capabilities of CCa cells following USP25-depleted (Fig. 6E and Supplementary Fig. 6E). In terms of metabolic changes, the total ATP production was elevated in CCa cells overexpressing KIFC1 despite USP25 inhibition (Fig. 6F and Supplementary Fig. 6F).This was accompanied by an increase the extracellular acidification rate (ECAR) (Fig. 6G and Supplementary Fig. 6G), the expression levels of GLUT1, HK2, LDH-A related to the glycolysis pathway were also significantly upregulated (Fig. 6H, I and Supplementary Fig. 6H, I).

These findings indicate that USP25 deubiquitination directly interacts with and stabilizes KIFC1. Loss of USP25 leads to enhanced ubiquitination and subsequent degradation of KIFC1, contributing to decreased malignancy in CCa. Thus, the USP25-KIFC1 axis presents a pivotal regulatory mechanism in cervical cancer progression, offering potential therapeutic targets.

### The USP25/KIFC1 signal axis recruits downstream MYCBP to play a carcinogenic role in CCa

**Fig. 7:**
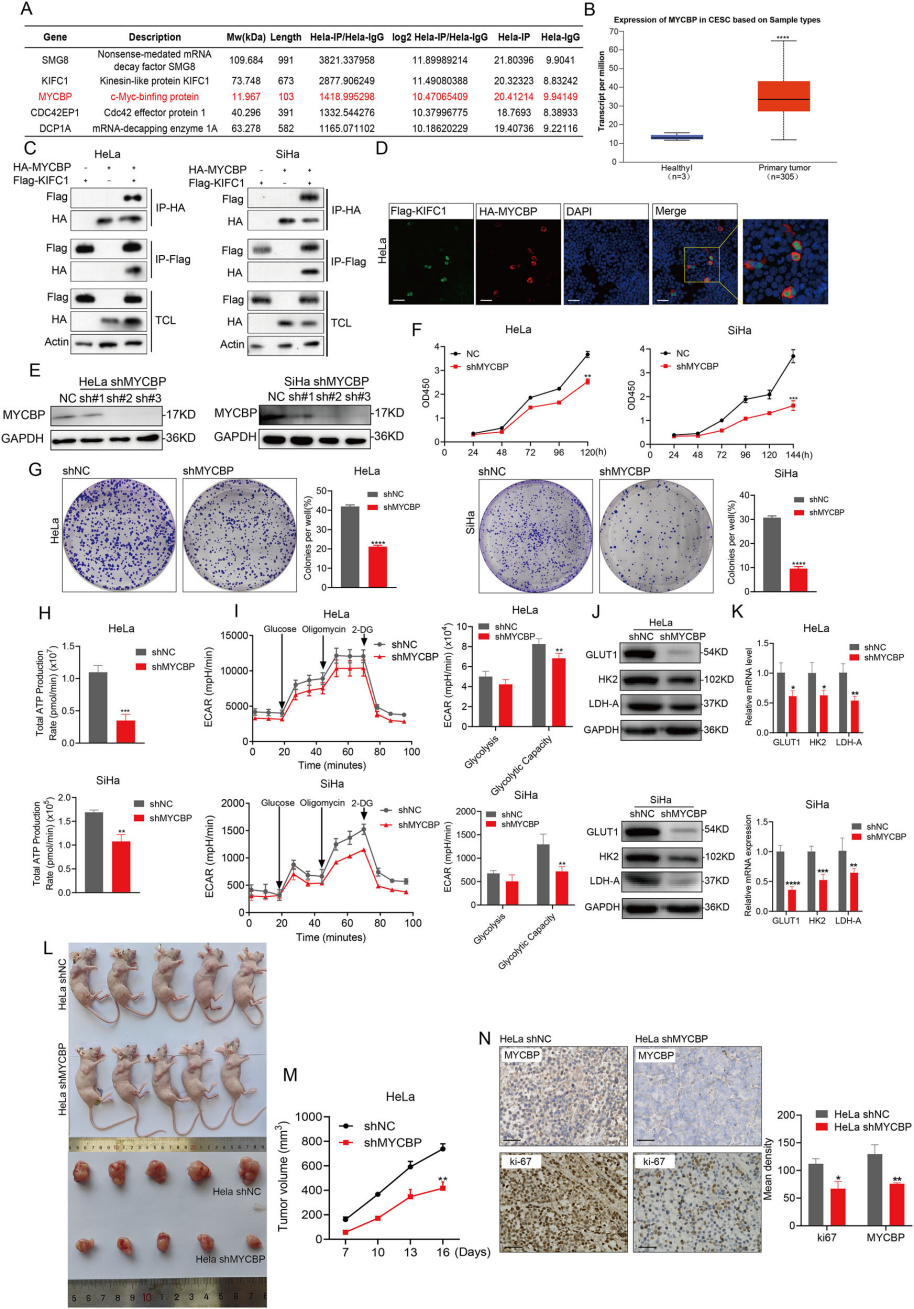
MYCBP interacts with KIFC1 and plays an oncogenic role in CCa both in vitro and in vivo. **A** MYCBP was obtained by IP-MS proteomic screening of proteins interacting with KIFC1. **B** UALCAN database analysis of MYCBP mRNA expression differences in cervical cancer. **C** Exogenous expressions of HA-MYCBP and Flag-KIFC1 were detected in CCa cells, and their interaction was detected by immunoprecipitation. **D** Immunofluorescence of KIFC1 and MYCBP subcellular localization in HeLa cells using Flag and HA protein antibodies. Scale bar, 100 μm. **E** MYCBP expression was down-regulated at different RNA interference sites by immunoblot in CCa cells. CCK-8 (**F**) and colony formation (**G**) assays were performed using MYCBP-depleted CCa cells. Representative images of colonies and relevant quantification are displayed in (**F**), respectively. **H** The total ATP production rate in MYCBP-depleted CCa cells was measured by cell energy metabolism. **I** Analyze the rate and corresponding statistical extracellular acidification rate (ECAR) of MYCBP-depleted CCa cells in aerobic glycolysis. **J**, **K** CCa cells infected with either control shRNA or MYCBP shRNA were subjected to various analyses to measure the expression levels of GLUT1, HK2 and LDH-A involved in glucose metabolism by Immunoblotting (**J**) and real-time PCR (**K**). **L**, **M** HeLa and HeLa^*shMYCBP*^ cells were injected subcutaneously into nude mice (*n* = 5) to construct tumor xenotransplantation model, the tumor volume was measured every 3 days when the tumor reached a certain size (about 7 days). On the 16th day, all mice were killed and underwent immunohistochemical staining. The size of the tumor was shown in (**L**), and the growth time of the xenograft was shown in (**M**). Immunohistochemical detection was performed to detect KIFC1 and ki-67 protein expression in the tumor tissue (**N**). Scale bar, 25 μm. *p < 0.05, **p < 0.001, ***p < 0.001, ****p < 0.0001 by Student’s unpaired two-sided t-test.

Previous studies have demonstrated that USP25 mediates the deubiquitination of KIFC1, enhancing the stability of the KIFC1 protein and thereby advancing the progression of CCa. Given this background, we investigated whether KIFC1 also contributes directly to tumorigenesis. Using IP-MS technology, we screened for proteins that naturally interact with KIFC1. Our results identified several proteins, including SMG8, MYCBP, CDC42EP, and DCP1A, that exhibited robust interactions with KIFC1 (Fig. 7A). Particularly, we focused on MYCBP, a protein known to bind to the N-terminus of the oncogenic protein c-MYC, enhancing its ability to activate E box-dependent transcription and promote tumorigenesis [22]. Analysis of the tumor TCGA database revealed that transcription levels of MYCBP in CCa tumor tissues were higher than in adjacent non-tumor tissues (Fig. 7B). Protein interaction assays confirmed the interaction between KIFC1 and MYCBP in HeLa and SiHa cells (Fig. 7C). Immunofluorescence studies further delineated the colocalization of KIFC1 and MYCBP within HeLa cells, providing visual confirmation of their interaction (Fig. 7D).

We conducted experiments where MYCBP expression was silenced in CCa cells (Fig. 7E). The suppression of MYCBP significantly reduced cell proliferation (Fig. 7F) and clonogenic ability (Fig. 7G) compared to the control group. Additionally, the suppression of MYCBP induced cell cycle arrest in the G0/G1 phase and reduced the proportion of cells in the S and G2/M phases (Supplementary Fig. 7A). This was accompanied by an increase in apoptosis rates compared to the control (Supplementary Fig. 7B). The migratory and invasive capabilities of CCa cells were also markedly reduced upon MYCBP-depleted (Supplementary Fig. 7C, D). Moreover, MYCBP inhibition curtailed aerobic glycolysis, as evidenced by decreased total ATP production (Fig. 7H) and extracellular acidification rate (ECAR) (Fig. 7I). MYCBP-depleted reduce the expression levels of several glycolytic genes, including GLUT1, HK2 and LDH-A (Fig. 7J, K). In vivo, MYCBP-limeted in HeLa cells led to significantly smaller tumors in a xenograft model in nude mice compared to controls (Fig. 7L, M), with immunohistochemistry revealing reduced ki-67 protein levels, indicative of lower proliferative activity (Fig. 7N).

### Cascade regulation of USP25-KIFC1-MYCBP in CCa

**Fig. 8:**
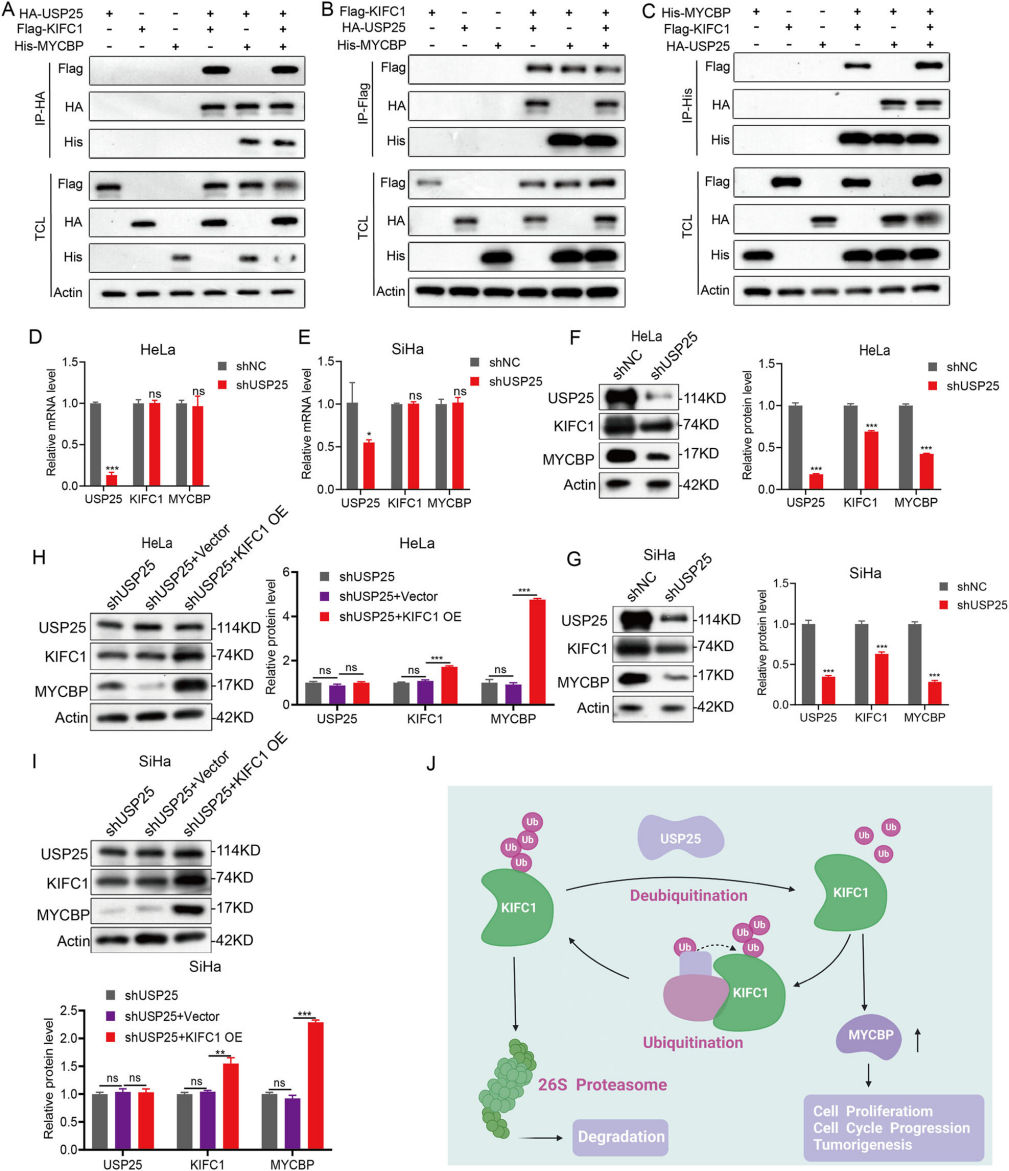
KIFC1 stability is regulated by USP25 and can regulate downstream MYCBP expression. **A**–**C** Co-immunoprecipitation with labeled antibodies showed the interaction between HA-USP25, Flag-KIFC1 and His-MYCBP. **D**, **E** USP25-depleted did not affect the mRNA expression difference of KIFC1 and MYCBP in CCa cells. **F**, **G** USP25-depleted significantly down-regulated the expression levels of KIFC1 and MYCBP in CCa cells. **H**, **I** The expression levels of USP25, KIFC1 and MYCBP proteins in the USP25-depleted CCa cells with different KIFC1 expression levels were analyzed in immunoblotting. **J** The proposed working model. USP25 deubiquitination maintains the stability of KIFC1 protein, and subsequently KIFC1 can regulate the expression level of downstream MYCBP to play a carcinogenic role. *p < 0.05, **p < 0.001, ***p < 0.001, ****p < 0.0001 by Student’s unpaired two-sided t-test.

Previous studies have confirmed that KIFC1 is not only stable by the deubiquitination of USP25, but also can interact with MYCBP. Co-immunoprecipitation of labeled antibodies showed that HA-USP25, Flag-KIFC1, His-MYCBP had interaction (Fig. 8A-C). In additional experiments, USP25 inhibition in CCa cells did not alter the transcript levels of KIFC1 and MYCBP compared to controls (Fig. 8D, E). Interestingly, downregulation of USP25 resulted in decreased protein levels of KIFC1 and MYCBP (Fig. 8F, G). Conversely, overexpression of KIFC1 in USP25-depleted CCa cells led to significant upregulation of both KIFC1 and MYCBP proteins (Fig. 8H, I). It has also been verified in vitro xenotransplantation models. After MYCBP was overexpressed in HeLa^*KIFC1-/-*^, the tumor growth rate was accelerated compared with the empty carrier group (Supplementary Fig. 8A-B), and the expression levels of ki-67 and MYCBP in tumor tissues were significantly upregulated (Supplementary Fig. 8E). After overexpression of KIFC1 and MYCBP in HeLa^*shUSP25*^, the tumor growth rate was also significantly accelerated compared with that in the empty vector group (Supplementary Fig. 8C, D). The expression level of MYCBP was also significantly up-regulated in tumor tissues of HeLa^*shUSP25+KIFC1 OE*^ (Supplementary Fig. 8F). This suggests that USP25 indirectly affects MYCBP stability through its interaction with KIFC1, establishing a crucial role for USP25 in maintaining KIFC1 protein stability, which in turn regulates MYCBP protein levels.

Our findings underline the importance of the USP25/KIFC1/MYCBP axis in the malignant progression of CCa (Fig. 8J). Although the precise mechanisms by which KIFC1 influences MYCBP protein levels require further investigation, the evidence supports a significant role for this axis in modulating CCa tumorigenesis.

## Discussion

CCa ranks among the deadliest malignancies of the female reproductive system worldwide [23, 24]. Despite advancements in CCa treatment over recent decades, challenges such as drug resistance, tumor recurrence, and metastasis persist, complicating treatment for both clinicians and patients [25, 26]. Immune checkpoint inhibitors targeting programmed cell death protein 1 (PD-1), programmed death-ligand 1 (PD-L1), and cytotoxic T-lymphocyte-associated protein 4 (CTLA-4) are under investigation for treating relapsed or metastatic CCa. However, these therapies yield a relatively low overall response rate (4%-26%) [27], underscoring the urgent need for identifying critical factors involved in CCa pathogenesis to develop novel therapeutic strategies.

KIFC1 plays a vital role in organelle transport, cell division, tissue and organ development, and signal transduction [28]. It is known that the attenuation of KIFC1 expression causes defects in mitosis. The KIFC1-delition affects the normal mitosis of cells, forming aneuploid cells [29***–***31]. When aneuploid cells enter the new cell cycle process, the abnormal number of chromosomes may affect the material preparation of the cell cycle process [32]. This may lead to the proliferation of G0/G1 phase cells caused by KIFC1-deletion in CCa cells. Notably, KIFC1 is overexpressed in various tumors, driving gene overexpression that controls mitotic checkpoints and induces aneuploidy, thus accelerating cancer progression [10]. In specific cancers, KIFC1 enhances tumor development through distinct pathways. In endometrial cancer, it binds to HMGA1 to promote c-MYC expression, accelerating the transcription of c-MYC-mediated glycolysis genes [33]. In liver cancer, KIFC1 activates the gankyrin/AKT/TWIST1 pathway, promoting epithelial-stromal transformation and metastasis [17]. Furthermore, KIFC1 has been shown to enhance bladder cancer cell proliferation and induce epithelial-mesenchymal transformation via the Akt/GSK3β signaling pathway [34]. Despite its established role in various cancers, the specific function of KIFC1 in CCa and its regulatory relationships remain inadequately understood.

In this study we analyzed of the TCGA cohort indicates that KIFC1 mRNA expression is elevated in CCa tissues. Tissue microarray analysis corroborates this, revealing that elevated KIFC1 protein levels correlate with poor prognostic outcomes in CCa patients. We confirmed that KIFC1 is indeed involved in the malignant progression of CCa in vitro and vivo. These observations highlight the critical role of KIFC1 protein expression in the biology of CCa tumors. Ubiquitination, a post-translational modification, involves the attachment of ubiquitin molecules to substrate proteins via a cascade catalyzed by ubiquitin ligases, affecting the substrate’s function, localization, stability and protein interactions [35]. Our studies show that USP25 modulates the stability of KIFC1 protein in CCa cells. Deubiquitinating enzymes deubiquitinate by directly interacting with proteins or by regulating the E3 ligase complex and the degron cascade signaling, resulting in indirect deubiquitination of various ubiquitinating proteins [36]. GST pull-down experiment shows that USP25 interacts directly with KIFC1, inhibiting its degradation through deubiquitination, it’s essential to maintain the stability of KIFC1. USP25-depleted can also lead to impaired malignant progression of CCa. Overexpression of KIFC1 can counteract the depleted malignant progression of CCa tumors caused by inhibition of USP25. It has been reported that USP25 depletion significantly changes the glycolytic metabolic pathway in pancreatic cancer [37]. Similarly, in CCa, we found that the depletion of USP25 and KIFC1 can inhibit the aerobic glycolytic process of CCa and significantly reduce the malignant progression of CCa.

Deregulating cellular energetics is one of the signs of malignant tumor [38]. The molecular mechanism of metabolic transformation is related to the activation of oncogenes or the loss of tumor suppressor factors [39], which ultimately leads to the stabilization of hypoxia-inducing factor HIF-1α or the increase of c-MYC oncogene expression. Transcription factors HIF1a and c-MYC promote the expression of glycolytic genes, thereby enhancing lactic acid production and glycolysis [40]. MYCBP was identified as an interacting partner of KIFC1 through IP-MS and protein interaction screening. MYCBP, which binds to the N-terminal of the oncogenic protein c-MYC, enhances c-MYC’s ability to activate E-box-dependent transcription [36]. c-MYC, a well-studied oncogene, plays a critical role in various tumors’ onset and progression. As a member of the “super transcription factor” family, c-MYC regulates transcription processes mediated by all RNA polymerases, affecting essential cellular functions including proliferation, differentiation, cell cycle progression, metabolism, and apoptosis [41–43].

Existing studies have not found a direct relationship between MYCBP and aerobic glycolysis. We found that MYCBP can indeed affect the intracellular acidification rate, inhibit aerobic glycolysis and reduce the production of total ATP. c-MYC has been confirmed to be involved in the regulation of aerobic glycolysis in cervical cancer [44]. The USP25/KIFC1/MYCBP signal axis detected by RT-PCR did not change the expression level of c-MYC mRNA(Supplementary Fig. 9A, B), but affected the expression levels of c-MYC transcription targets CDK4, CDK2, cyclin D1 and cyclin E (Supplementary Fig. 9C) which these have been reported to be regulated by c-MYC transcription in cervical cancer [45–47]. It is undeniable that MYCBP is involved in the malignant progression of CCa. In addition, the USP25/KIFC1/MYCBP had an obvious interaction by Co-Immunoprecipitation, and the expression level of KIFC1 was maintained by USP25 deubiquitination, and KIFC1 could regulate the expression level of downstream MYCBP. This regulatory relationship has also been verified in vivo xenotransplantation experiments.

Our findings elucidate KIFC1’s oncogenic potential in CCa. The deubiquitination of KIFC1 by USP25 stabilizes KIFC1 protein levels, which in turn modulates MYCBP expression, impacting CCa’s malignant progression and energy metabolism, but how to use KIFC1 in the diagnosis and treatment of CCa requires further study. Undeniable, high KIFC1 expression correlates with poor patient prognosis, positioning KIFC1 as a promising biomarker and therapeutic target for CCa.

## Supplementary information

Supplementary material legends

Supplementary Figure 1

Supplementary Figure 2

Supplementary Figure 3

Supplementary Figure 4

Supplementary Figure 5

Supplementary Figure 6

Supplementary Figure 7

Supplementary Figure 8

Supplementary Figure 9

Original WB images

IP_MS

## Data Availability

The data that support the findings of this study are available from the corresponding author upon reasonable request.
