# Supplementary material for: USP25-driven KIFC1 regulates MYCBP expression and promotes the progression of cervical cancer
Source: Cell Death Dis. 2025 May 16;16(1):390. doi: 10.1038/s41419-025-07713-x (PMC12084419; doi:10.1038/s41419-025-07713-x)
Supplement: Supplementary file 1 — Supplementary material legends [file 41419_2025_7713_MOESM1_ESM.docx]

**Supplementary figures and figure legends**

**Supplementary Figure 1. Expression of KIFC1 protein was detected by microarray in cervical cancer during survival.**

The survival time of 99 cases of cervical cancer, 111 points, mainly squamous cell carcinoma: 99 points of cancer/12 points paracancerous, red box near cancer, other tumor tissue.

**Supplementary Figure 2. Expression of KIFC1 in different cervical cancer cells.**

The expression of KIFC1 protein and mRNA in 7 cervical cancer cells was detected by RT-qPCR (A) and immunoblotting (B), and normal cervical epithelial cells HCerEpiC as the control. *p<0.05, **p<0.001, ***p<0.001, ****p<0.0001 by Student’s unpaired two-sided t-test.

**Supplementary Figure 3. Overexpression of KIFC1 by CCa cells after KIFC1 knockout promotes malignant progression of the tumor.**

1. Immunoblotting detects the delivery of exogenous overexpression of KIFC1 by pLVX vector in HeLa*^KIFC1-/-^* and SiHa*^KIFC1-/-^* cells. (B-C) CCK-8 (B) and colony formation (C) assays were performed using KIFC1-overexpressing in HeLa*^KIFC1-/-^* and SiHa*^KIFC1-/-^* cells. Representative images of colonies and relevant quantification are displayed in C, respectively. (D-E) KIFC1-overexpressing in HeLa*^KIFC1-/-^* and SiHa*^KIFC1-/-^* cells，the cell cycle (D) and apoptosis (E) were detected by flow cytometry. (F-G) Transwell migration and invasion assays were conducted with KIFC1-overexpressing in HeLa*^KIFC1-/-^* and SiHa*^KIFC1-/-^* cells. Representative images and the quantitative results of migrated or invaded cells are displayed in (F) and (G), respectively. Scale bar, 200 μm. *p<0.05,**p<0.001,***p<0.001,

****p<0.0001 by Student’s unpaired two-sided t-test.

**Supplementary Figure 4. Histogram of fluorescence intensity of KIFC1 combined with USPs**

**Supplementary Figure 5. Knocking down USP25 inhibited the invasion and metastasis of CCa cells i*n vitro* and tumor formation *in vivo*.**

1. B) Transwell migration and invasion assays were conducted with USP25-limited CCa cells. Representative images and the quantitative results of migrated or invaded cells are displayed in (A) and (B), respectively. Scale bar, 200 μm. (C-E) HeLa cells with stable expression of shNC and shUSP25 were implanted subcutaneously in the back of nude mice (n=5), and the tumor volume was measured every 3 days when the tumor reached a certain size (about 7 days). On the 19th day, all mice were killed and underwent immunohistochemical staining. The size of the tumor was shown in (C), and the growth time of the xenograft was shown in (D). Immunohistochemical detection was performed to detect USP25 and ki-67 protein expression in the tumor tissue (E). Scale bar, 75 μm. *p<0.05, **p<0.001, ***p<0.001, ****p<0.0001 by Student’s unpaired two-sided t-test.

**Supplementary Figure 6. After SiHa cells inhibited USP25, overexpression of KIFC1 rescued malignant progression of cells**

1. B) CCK-8 (A) and colony formation (B) assays were performed using KIFC1-overexpressing SiHa*^shUSP25^* cells. Representative images of colonies and relevant quantification are displayed in B, respectively. (C-D) The cell cycle (C) and apoptosis (D) of KIFC1-overexpressing SiHa*^shUSP25^* cells were detected by flow cytometry, the corresponding data statistics are shown in the figure.(E) Transwell migration and invasion assays were conducted with KIFC1-overexpressing SiHa*^shUSP25^* cells. Representative images and the quantitative results of migrated or invaded cells are displayed in E, respectively. Scale bar, 200 μm. (F-G) Total ATP production of KIFC1-overexpressing SiHa*^shUSP25^* cells (F) and extracellular acidification rate (ECAR) of aerobic glycolysis cells were measured by energy metabolism (G). (H-I) SiHa*^shUSP25 +Vector^* and SiHa*^shUSP25 +KIFC1 OE^* cells were subjected to various analyses to measure the expression levels of GLUT1, HK2 and LDH-A involved in glucose metabolism by Immunoblotting (H) and real-time PCR (I). *p<0.05, **p<0.001, ***p<0.001, ****p<0.0001 by Student’s unpaired two-sided t-test.

**Supplementary Figure 7. Inhibition of MYCBP led to apoptosis and cycle arrest of CCa cells and impaired invasion and metastasis ability.**

(A-B) The cell cycle (A) and apoptosis (B) of MYCBP-depleted CCa cells were detected by flow cytometry, the corresponding data statistics are shown in the figure. (C-D) Transwell migration and invasion assays were conducted with KIFC1-depleted CCa cells. Representative images and the quantitative results of migrated or invaded cells are displayed in (C) and (D), respectively. Scale bar, 200μm.*p<0.05, **p<0.001, ***p<0.001, ****p<0.0001 by Student’s unpaired two-sided t-test.

**Supplementary Figure 8. USP25/KIFC1/MYCBP cascade regulates CCa tumor growth in vivo.**

1. B) Hela*^kIFC1-/-+Vector^* and HeLa*^KIFC1-/-+MYCBP OE^* were used to conduct tumor formation experiments in nude mice. Tumor tissue (A) was taken from the mice on the 19th day.Tumor growth size (B) was recorded every two days. (C-D) Hela*^shUSP5+Vector^* and HeLa*^shUSP5+KIFC1 OE^* and HeLa*^shUSP5+MYCBP OE^* cells were used to conduct tumor formation experiments in nude mice. Tumor tissue (C) was taken from the mice on the 15th day, and tumor growth size (D) was recorded every two days. (E) The expression of KIFC1, ki-67 and MYCBP proteins in Hela*^kIFC1-/-+Vector^* and HeLa*^KIFC1-/-+MYCBP OE^* tumor tissues was detected, and the expression differences were analyzed. Scale bar, 75μm. (F) The expression of KIFC1, ki-67 and MYCBP proteins in Hela*^shUSP5+Vector^* and HeLa*^shUSP5+KIFC1 OE^* and HeLa*^shUSP5+MYCBP OE^* tumor tissues was detected, and the expression differences were analyzed. Scale bar, 75 μm. *p<0.05, **p<0.001, ***p<0.001, ****p<0.0001 by Student’s unpaired two-sided t-test.

**Supplementary Figure 9. USP25/KIFC1/MYCBP axis affect the expression of c-MYC transcriptional targets.**

1. KIFC1 and MYCBP were overexpressed in USP25-limited CCa cells, and MYCBP was overexpressed in KifC1-deletion CCa cells by RT-PCR to identify gene expression. (B) The expression difference of KIFC1 and MYCBP did not affect the expression level of c-MYC mRNA.(C) The expression difference between KIFC1 and MYCBP affects the mRNA expression levels of CDK4, CDK2, cyclin D1, cyclin E which regulated by c-MYC transcription.*p<0.05,**p<0.001,***p<0.001,****p<0.0001 by Student’s unpaired two-sided t-test.

Table S1. KIFC1 sgRNA sequence

| Gene Name | | Targeting sequences (5`-3`) |
| --- | --- | --- |
| KIFC1 sgRNA 1 | Forward | caccGAACTAAAACGGTGCCGTGAG |
|  | Reverse | aaacCTCACGGCACCGTTTTAGTTC |
| KIFC1 sgRNA 2 | Forward | caccGGAACTTGCGTGCTTGTGTCC |
|  | Reverse | aaacGGACACAAGCACGCAAGTTCC |

Table S2. MYCBP and USP25 shRNA sequence

| siRNA/shRNA | Targeting sequences (5`-3`) |
| --- | --- |
| MYCBP#1 | ATGGCCCATTACAAAGCCGCC |
| MYCBP#2 | TAGAACTGGCCGAAATGAAAG |
| MYCBP#3 | TAGAACTGGCCGAAATGAAAG |
| USP25#1 | TCCATCGAACAATTGAATTAA |
| USP25#2 | TCCATCGAACAATTGAATTAA |
| USP25#3 | CAAGCCTTGAAGGATAGTAAT |

Table S3. KIFC1 knockout genomic RT-PCR validation primer

| Primer | Targeting sequences (5`-3`) |
| --- | --- |
| Forward | AGAAGCCAGTTCCTGCTGTTCCTGT |
| Reverse | AGAAGCCAGTTCCTGCTGTTCCTGT |

Table S4. RT-qPCR series primers

| Gene Name | | Targeting sequences (5`-3`) |
| --- | --- | --- |
| KIFC1 | Forward | GGTGCAACGACCAAAATTACC |
|  | Reverse | GGGTCCTGTCTTCTTGGAAAC |
| USP25 | Forward | GCACCAGCAGACGTTTTTGAA |
|  | Reverse | AGCATTCTTCGCAGTAAGGAAA |
| MYCBP | Forward | ATGGCCCATTACAAAGCCG |
|  | Reverse | TTTCTGGAGTAGCAGCTCCTAA |
| GAPDH | Forward | GGAGCGAGATCCCTCCAAAAT |
|  | Reverse | GGCTGTTGTCATACTTCTCATGG |
| GLUT1 | Forward | TCTGGCATCAACGCTGTCTT |
|  | Reverse | CCGTGTTGACGATACCGGAG |
| LDHA | Forward | GATTCAGCCCGATTCCGTTAC |
|  | Reverse | AGAGACACCAGCAACATTCATTC |
| HK2 | Forward | TGAGGTCCTGATGCGGTTGG |
|  | Reverse | TCGCCTTTGTTCTCCTTGATGC |
| CDK4 | Forward | AGACCAGGACCTAAGGACATATC |
|  | Reverse | GCTTGACTGTTCCACCACTT |
| CDK2 | Forward | ATGGACGGAGCTTGTTATCG |
|  | Reverse | TACTGGCTTGGTCACATCCT |
| Cyclin D1 | Forward | CAGACCTTCGTTGCCCTCTG |
|  | Reverse | CAGTCCGGGTCACACTTGAT |
| Cyclin E | Forward | CTGGATGTTGACTGCCTTGAAT |
|  | Reverse | TCTCTATGTCGCACCACTGAT |
