# Supplementary figures and images for: USP25-driven KIFC1 regulates MYCBP expression and promotes the progression of cervical cancer
Source: Cell Death Dis. 2025 May 16;16(1):390. doi: 10.1038/s41419-025-07713-x (PMC12084419; doi:10.1038/s41419-025-07713-x)

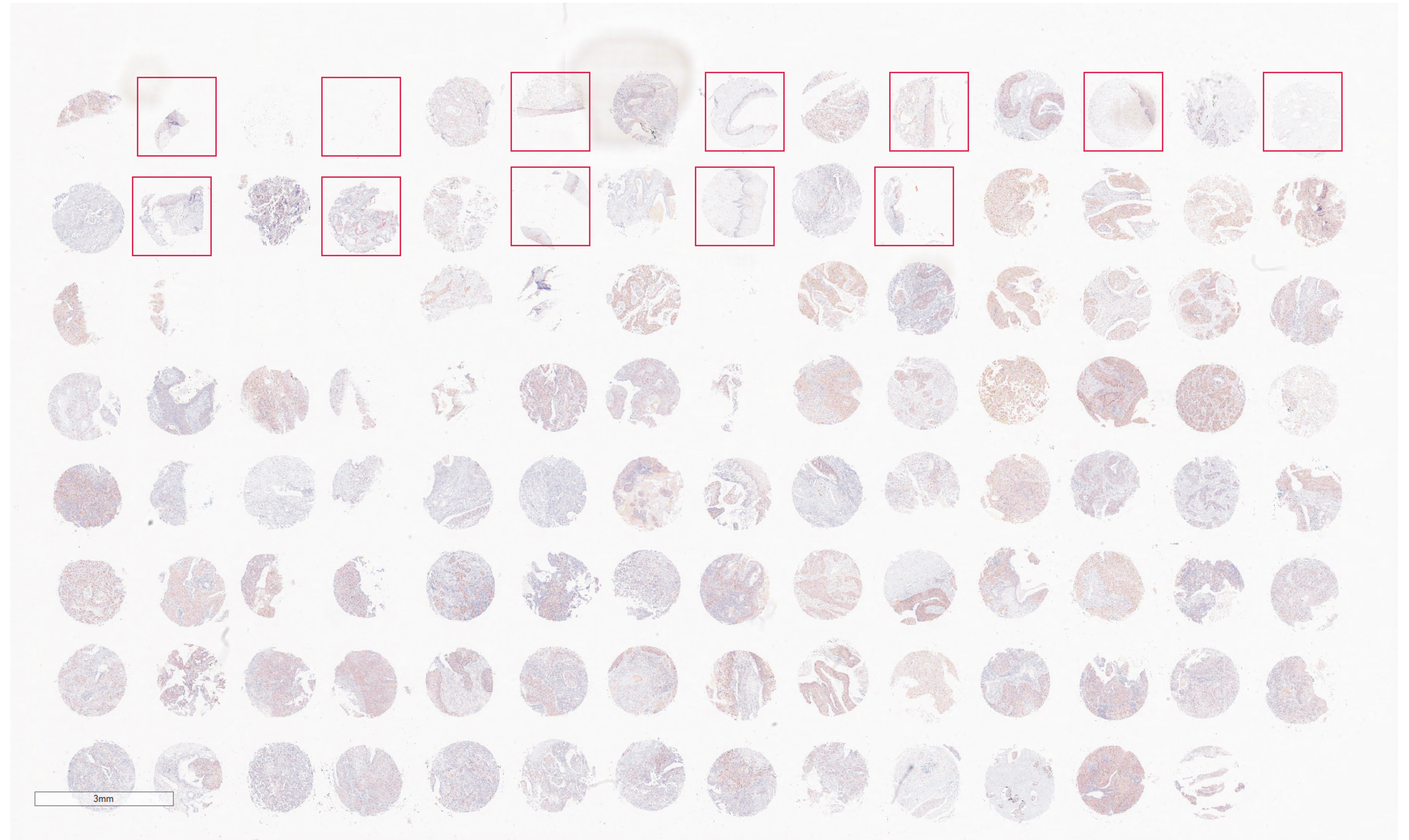

Supplement: Supplementary file 2 — Supplementary Figure 1 [file 41419_2025_7713_MOESM2_ESM.tif]

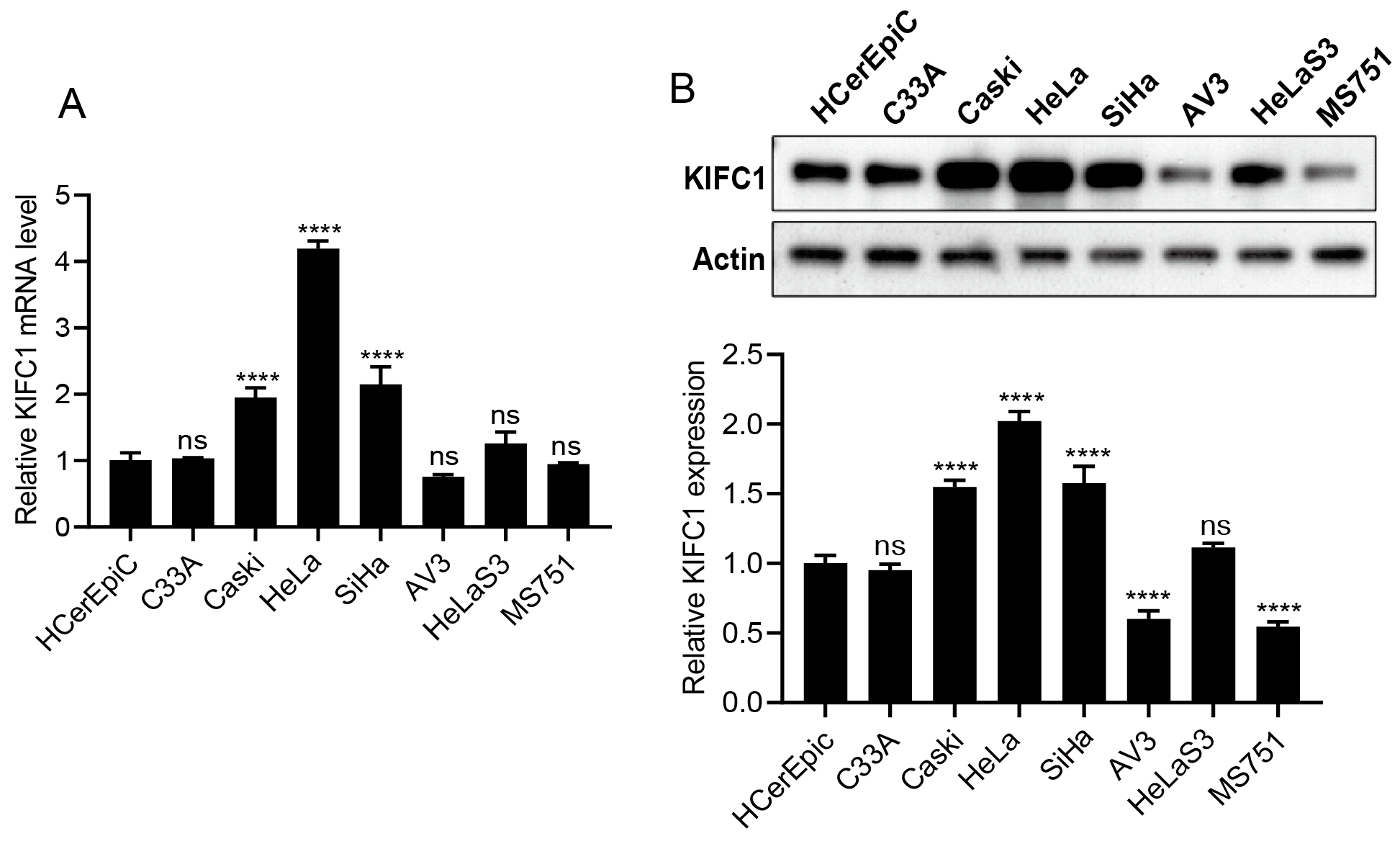

Supplement: Supplementary file 3 — Supplementary Figure 2 [file 41419_2025_7713_MOESM3_ESM.png]

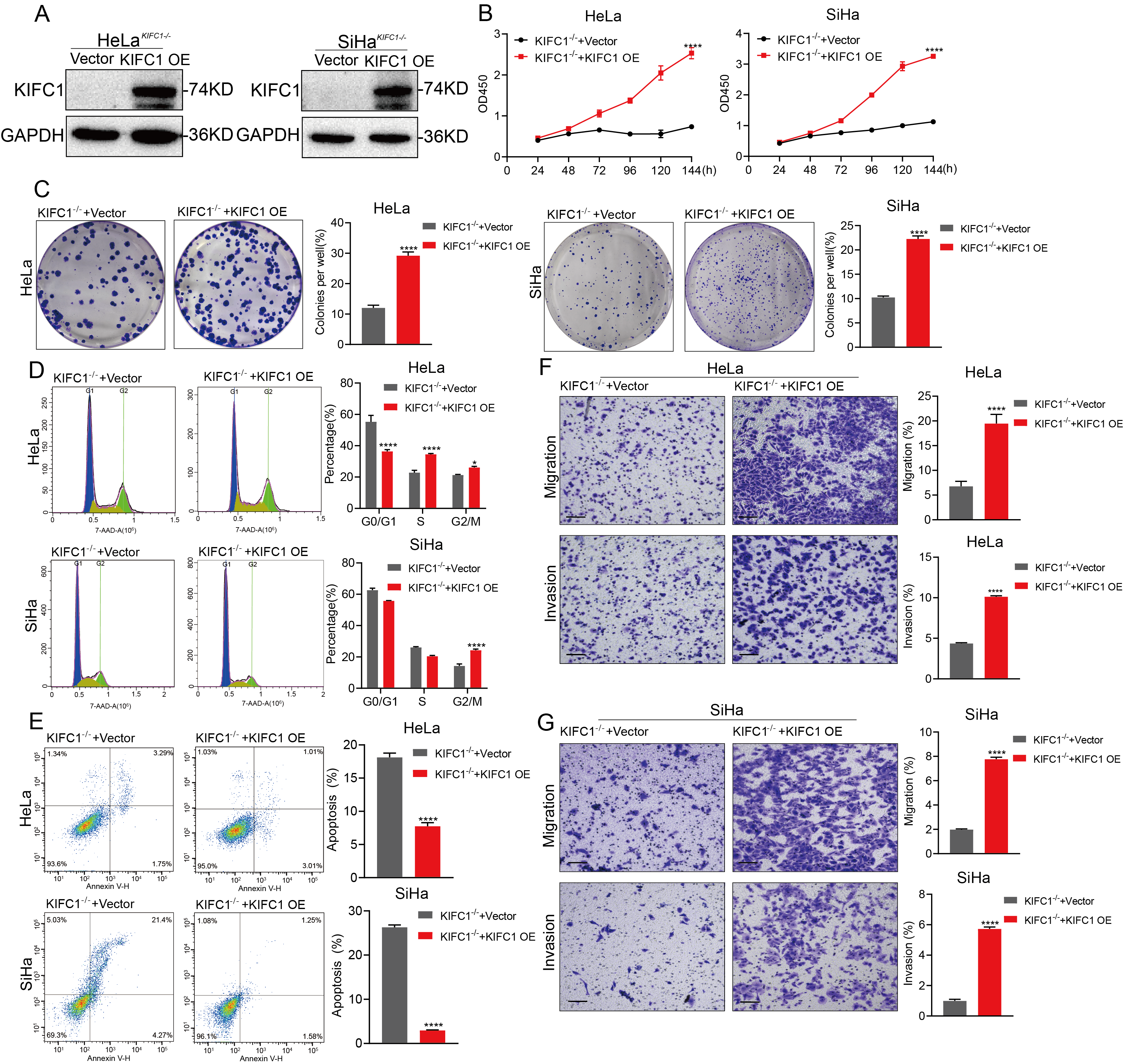

Supplement: Supplementary file 4 — Supplementary Figure 3 [file 41419_2025_7713_MOESM4_ESM.png]

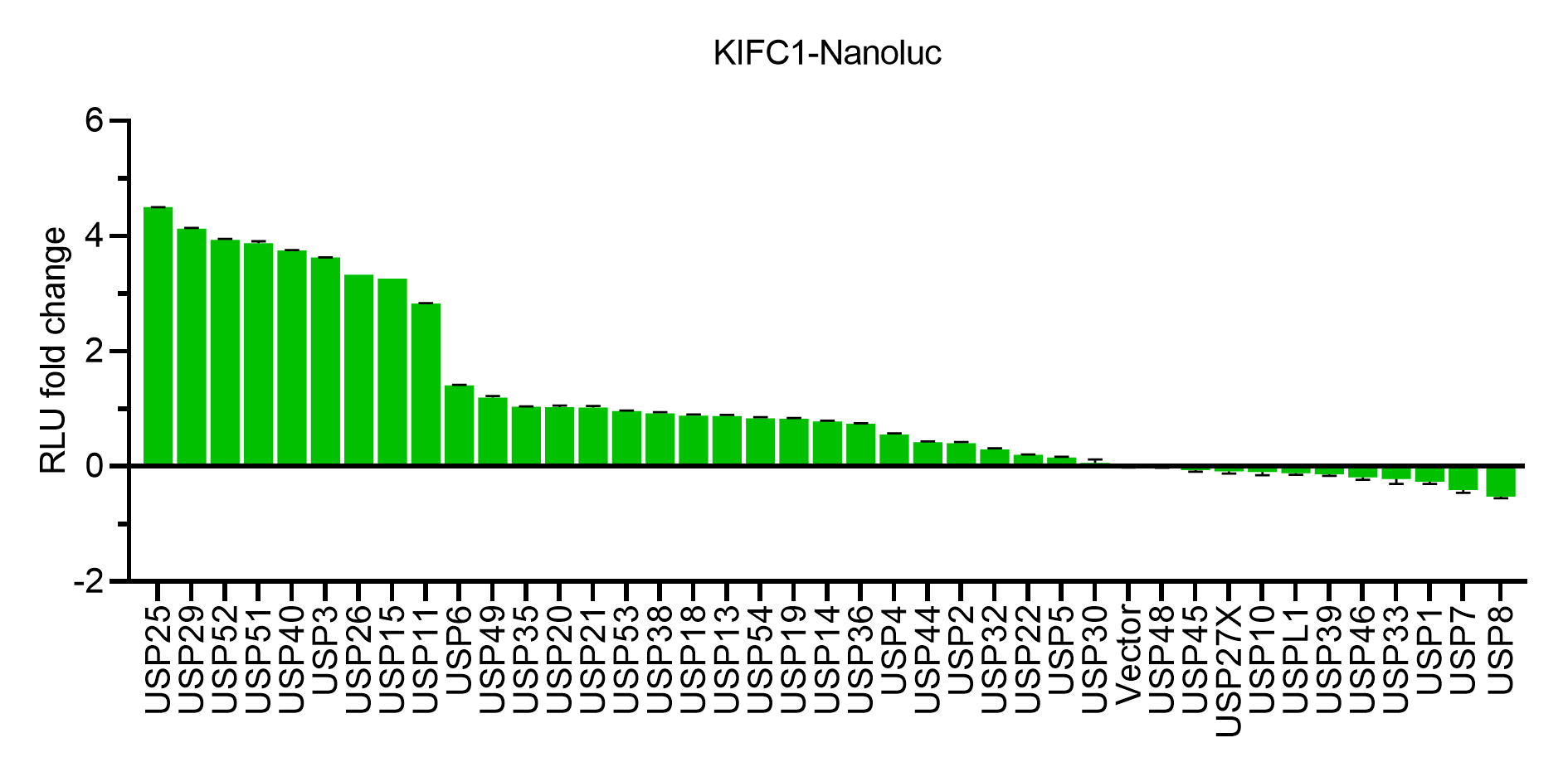

Supplement: Supplementary file 5 — Supplementary Figure 4 [file 41419_2025_7713_MOESM5_ESM.tif]

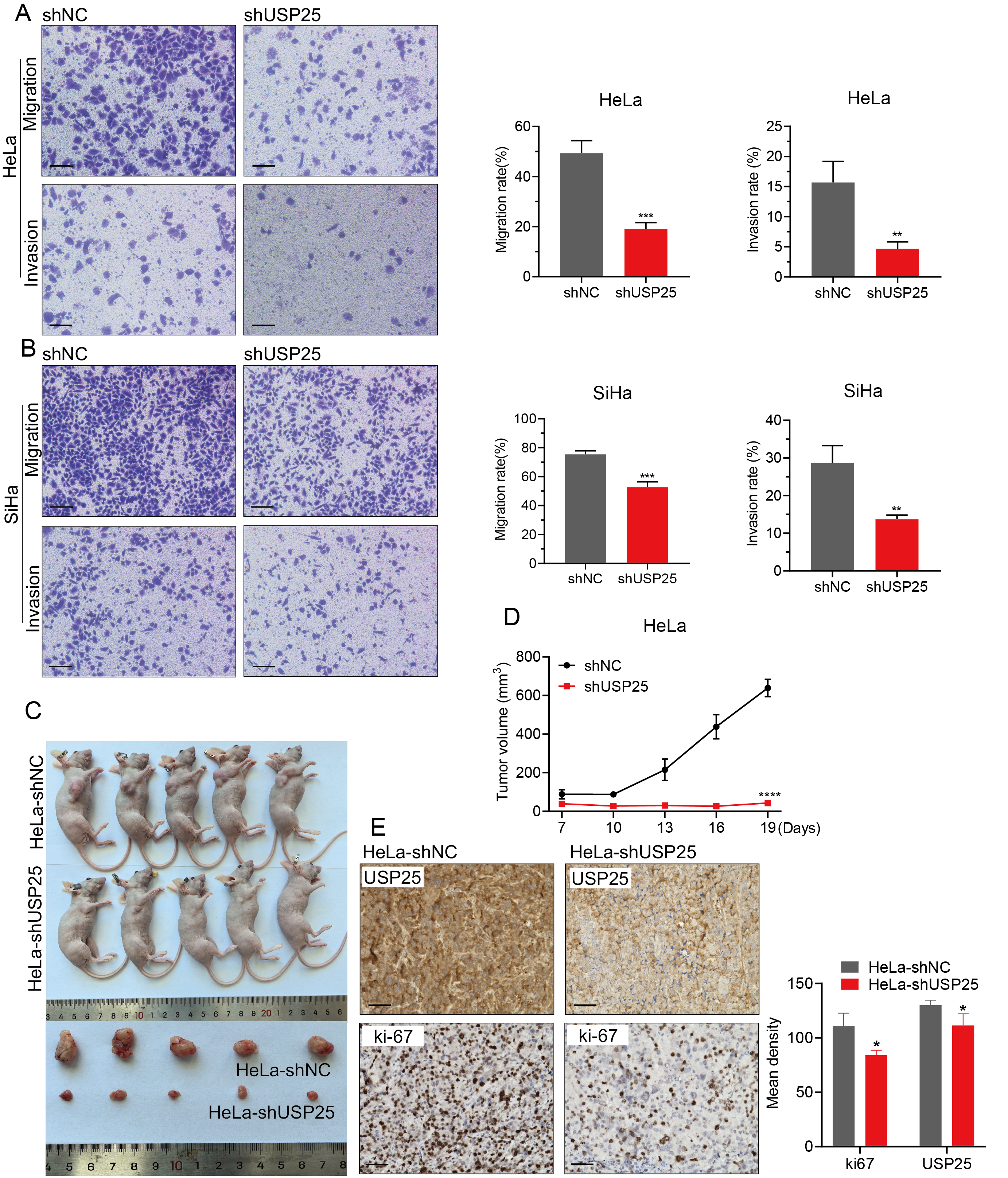

Supplement: Supplementary file 6 — Supplementary Figure 5 [file 41419_2025_7713_MOESM6_ESM.png]

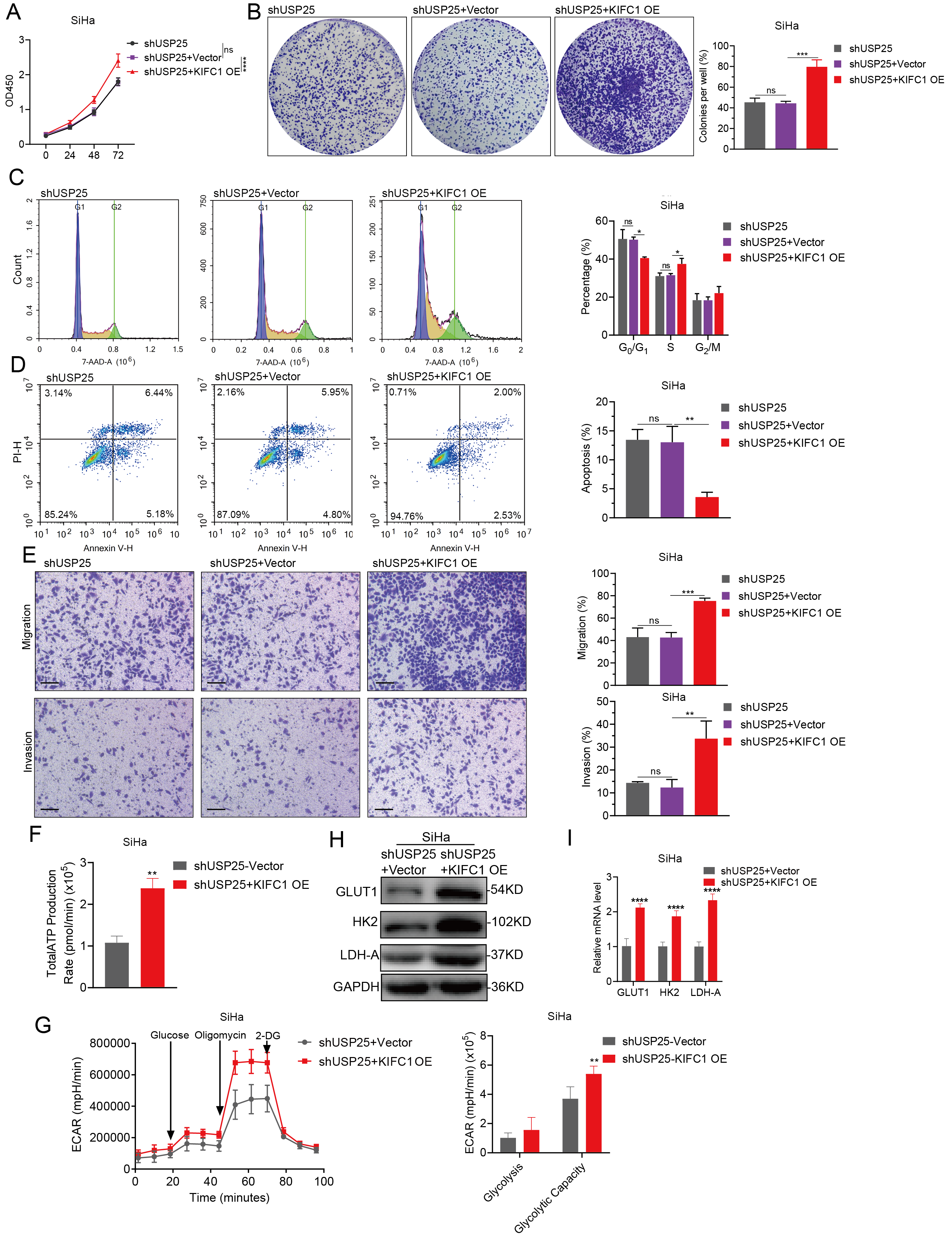

Supplement: Supplementary file 7 — Supplementary Figure 6 [file 41419_2025_7713_MOESM7_ESM.png]

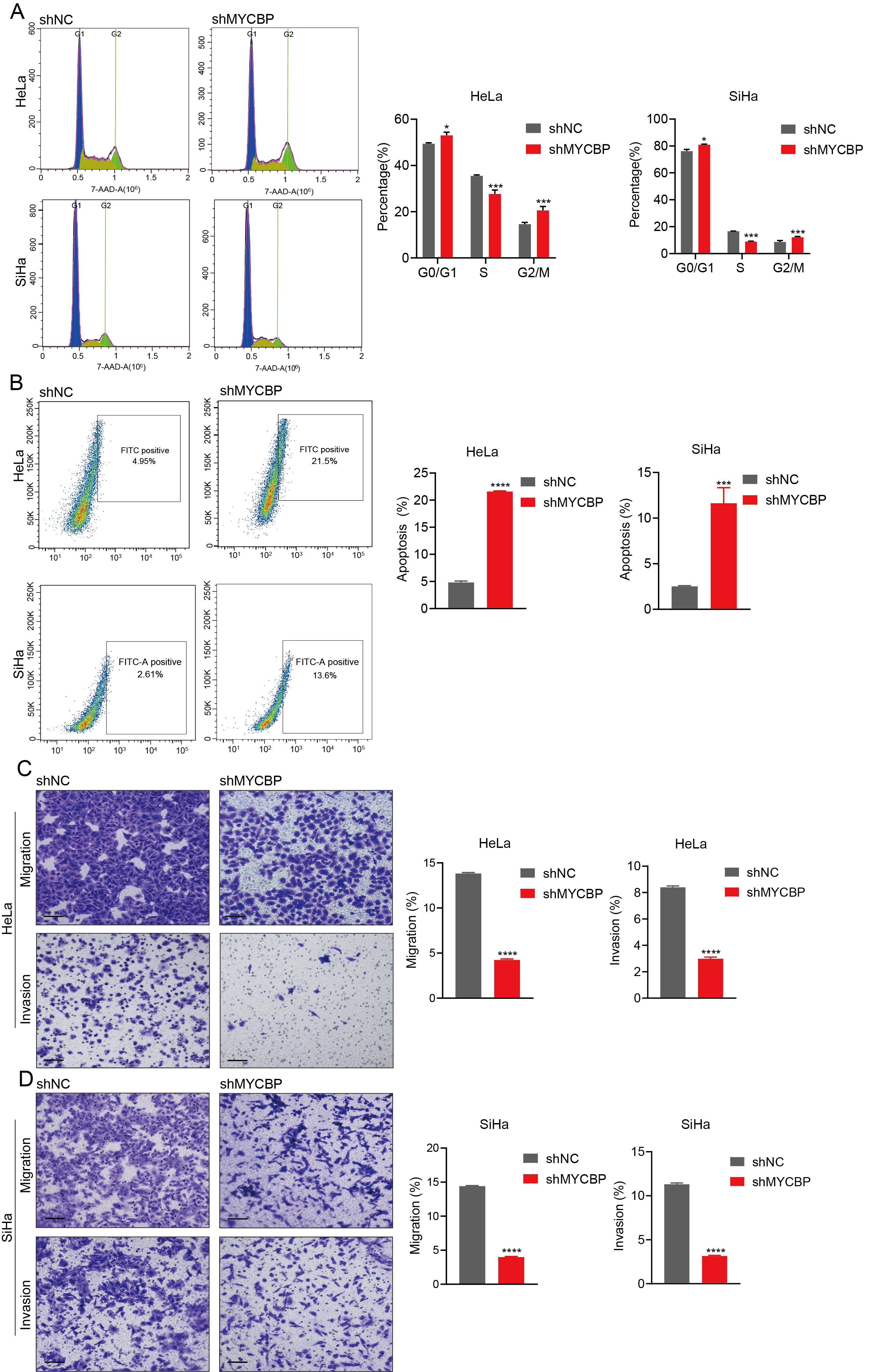

Supplement: Supplementary file 8 — Supplementary Figure 7 [file 41419_2025_7713_MOESM8_ESM.png]

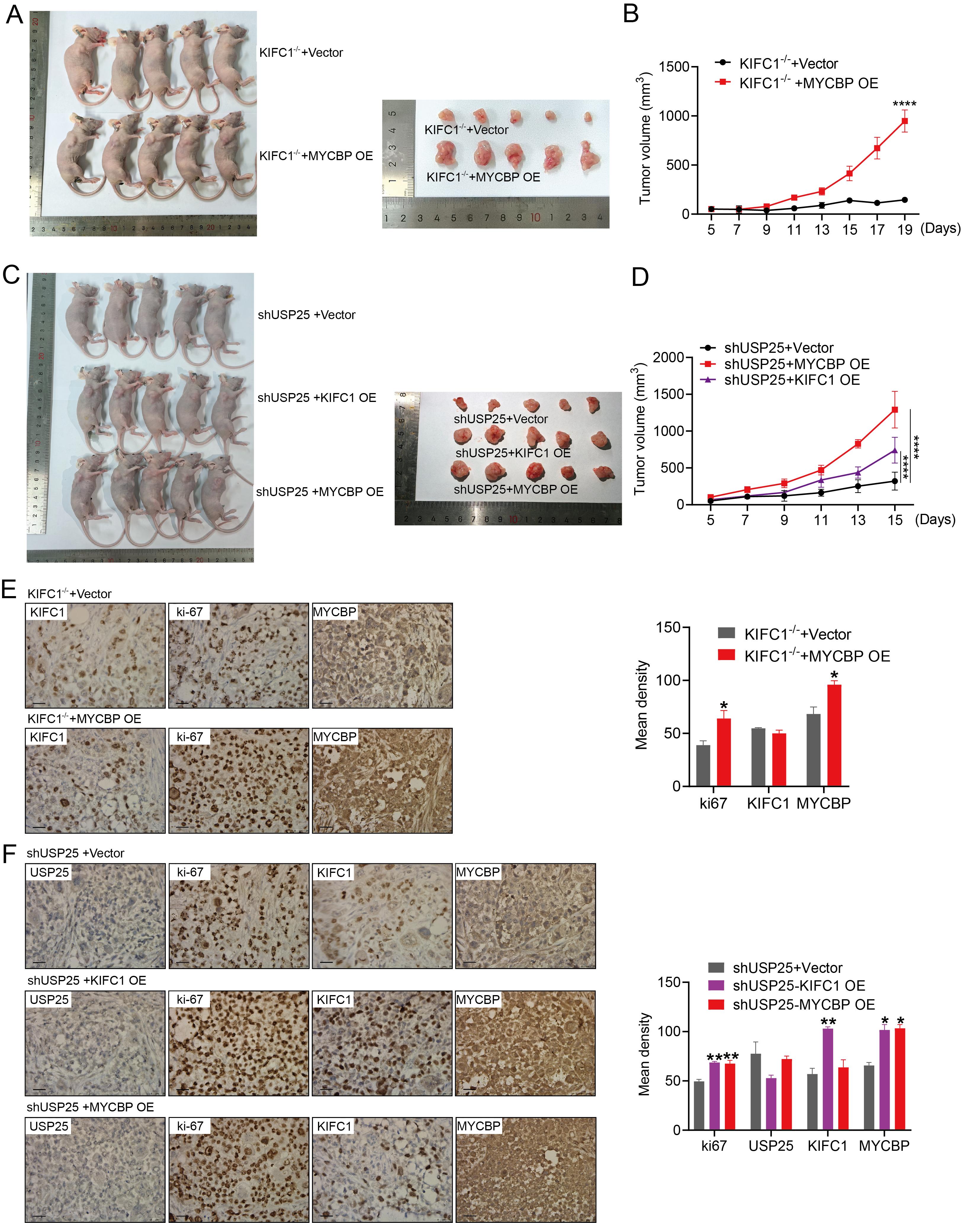

Supplement: Supplementary file 9 — Supplementary Figure 8 [file 41419_2025_7713_MOESM9_ESM.png]

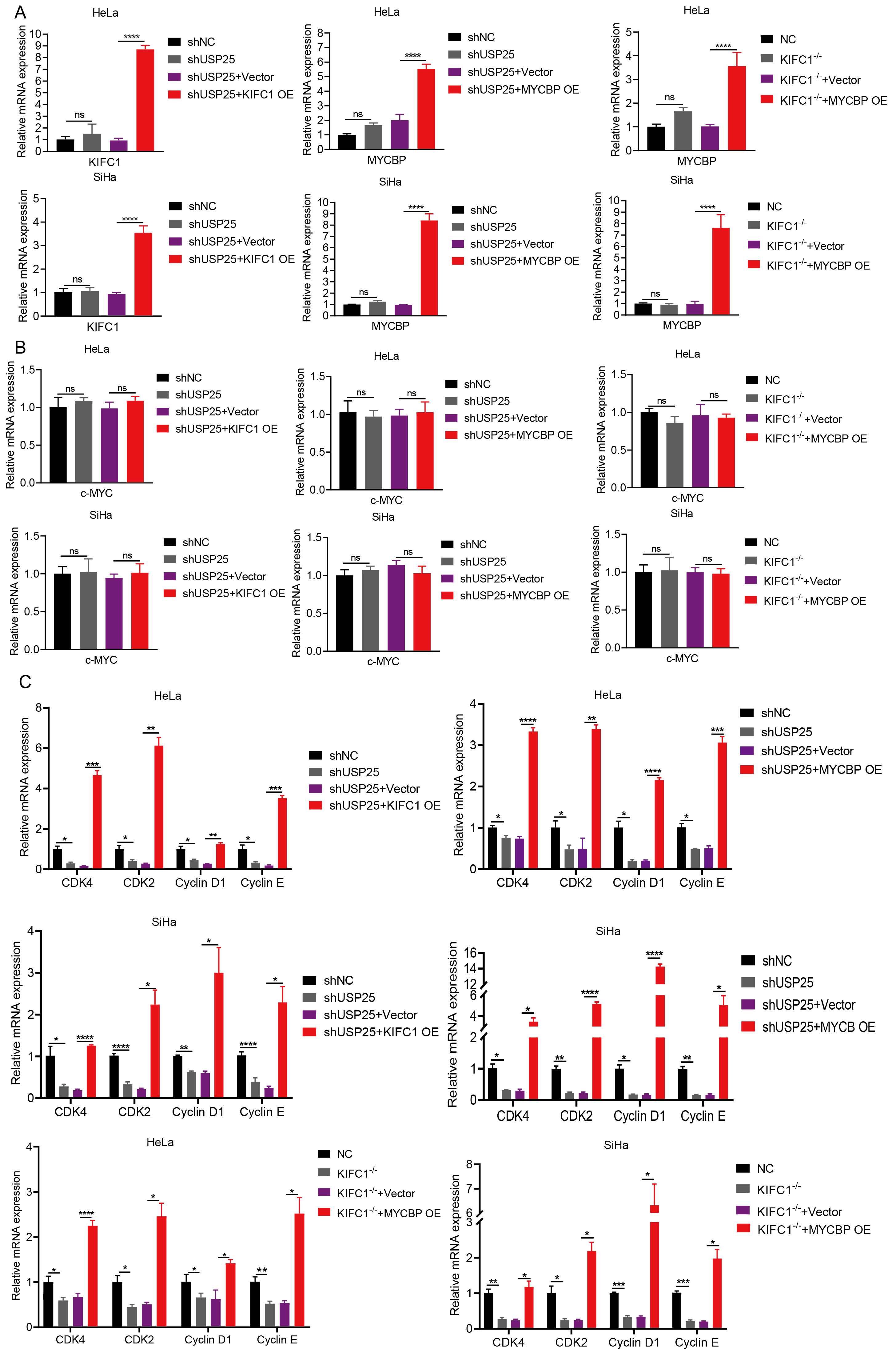

Supplement: Supplementary file 10 — Supplementary Figure 9 [file 41419_2025_7713_MOESM10_ESM.png]
