# Supplementary material for: USP25-driven KIFC1 regulates MYCBP expression and promotes the progression of cervical cancer
Source: Cell Death Dis. 2025 May 16;16(1):390. doi: 10.1038/s41419-025-07713-x (PMC12084419; doi:10.1038/s41419-025-07713-x)

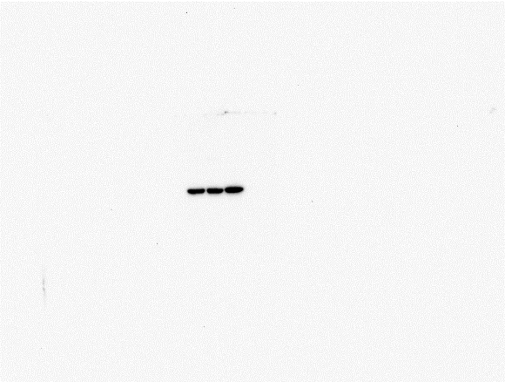

Fig 2 A HeLa GAPDH

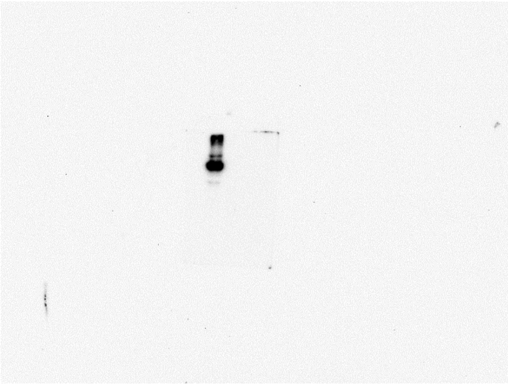

Fig2 A HeLa KIFC1

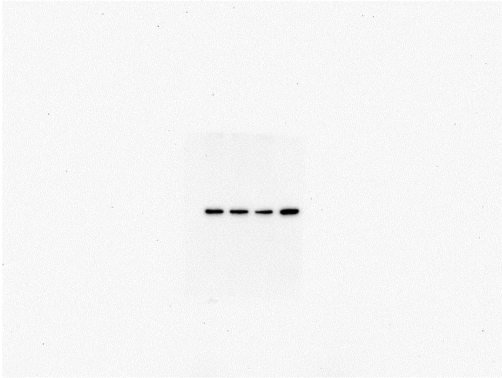

Fig2 A SiHa GAPDH

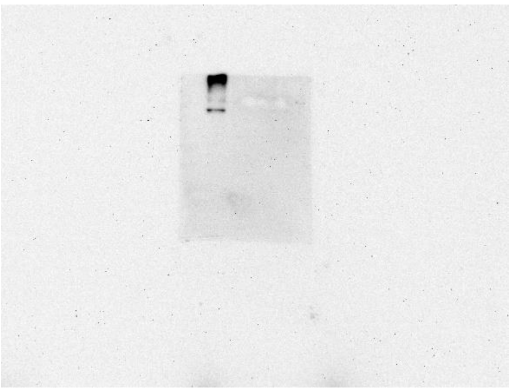

Fig2 A SiHa KIFC1

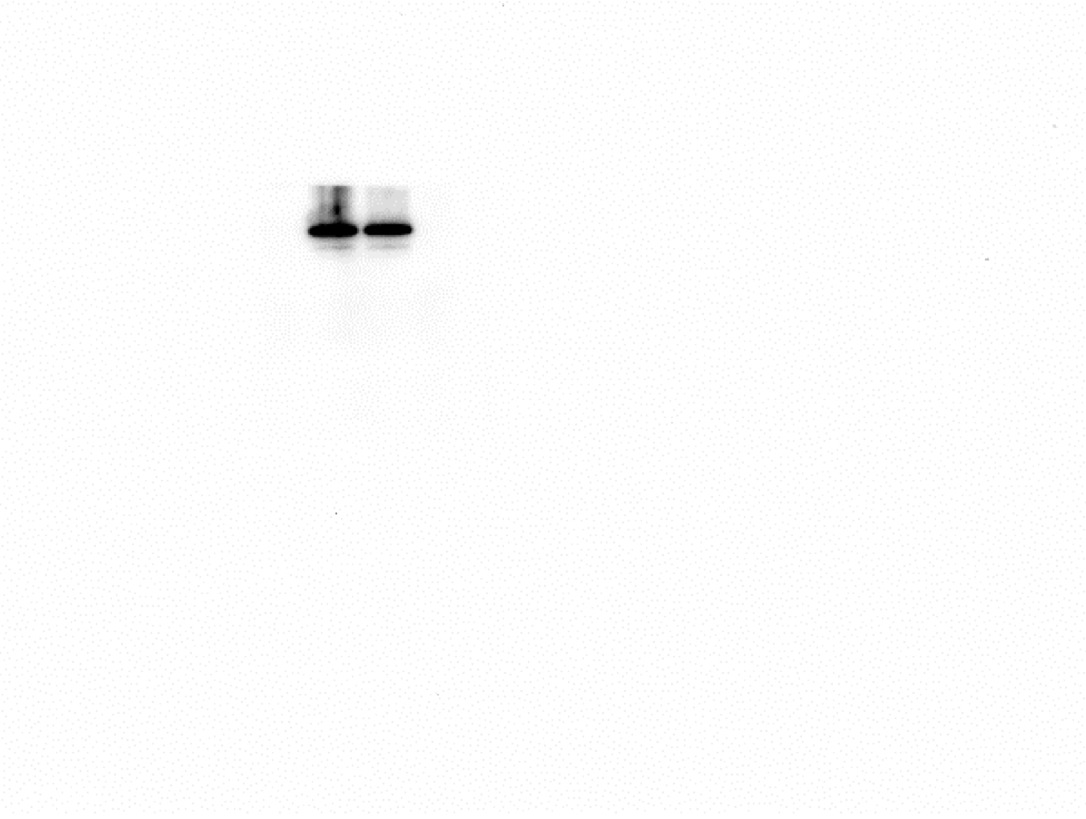

Fig4 C HeLa GAPDH

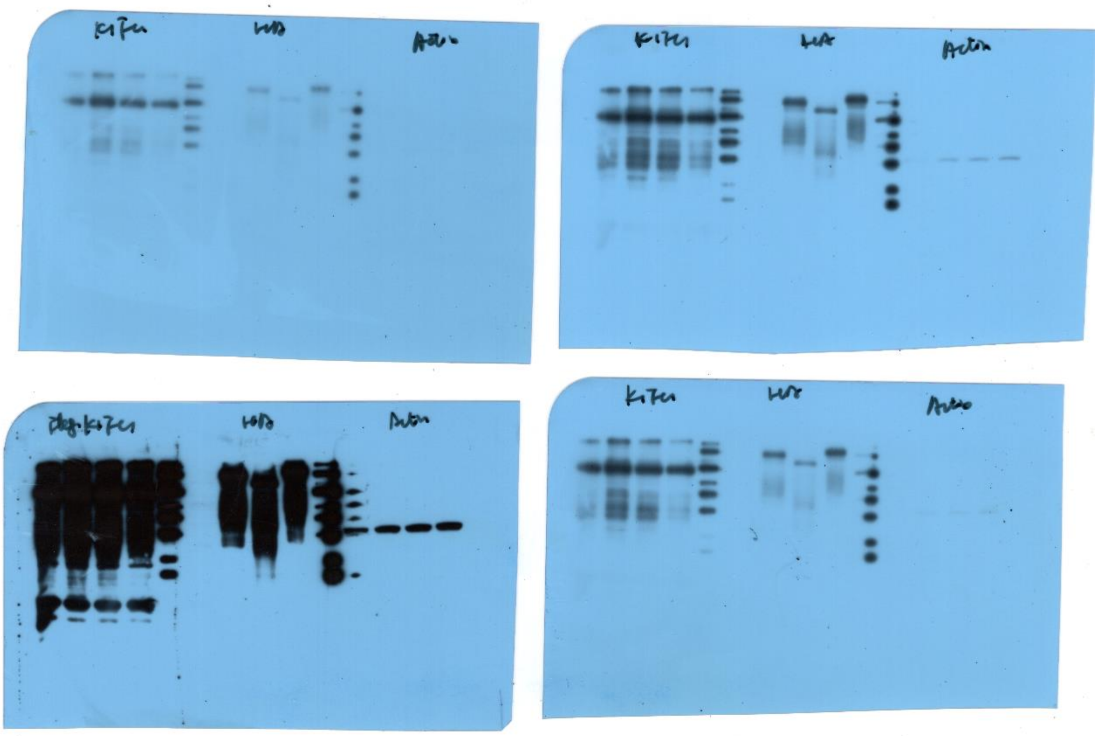

Fig4 B

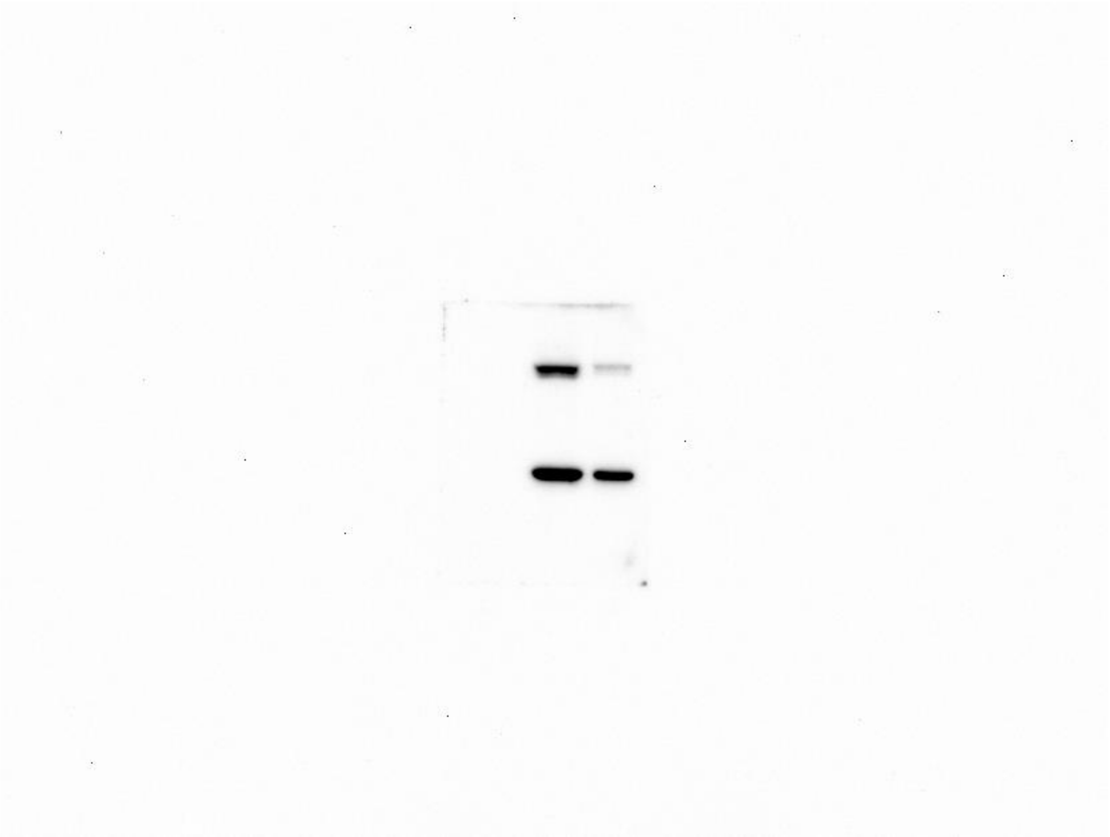

Fig4 C HeLa KIFC1

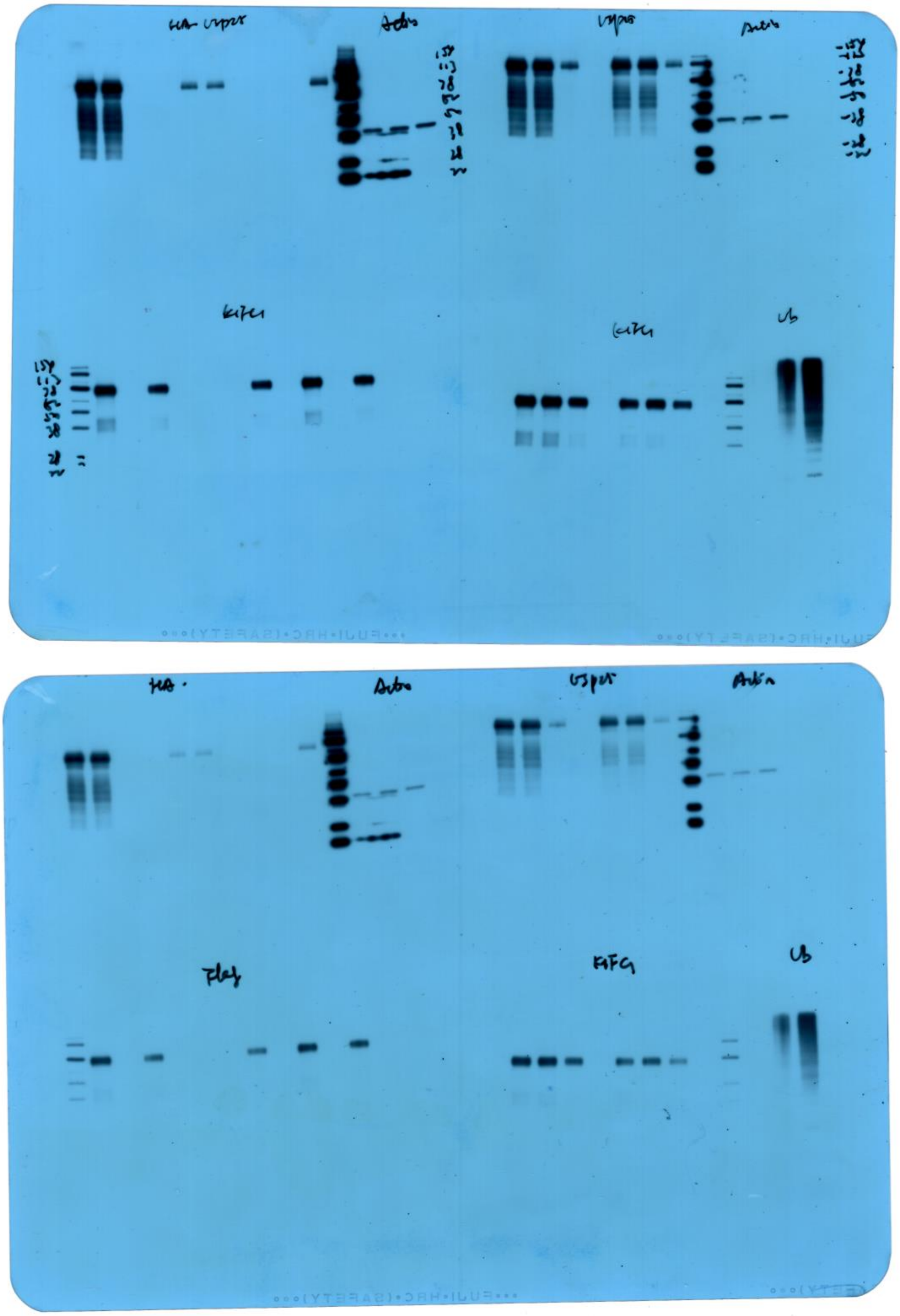

Fig4 D-E-1

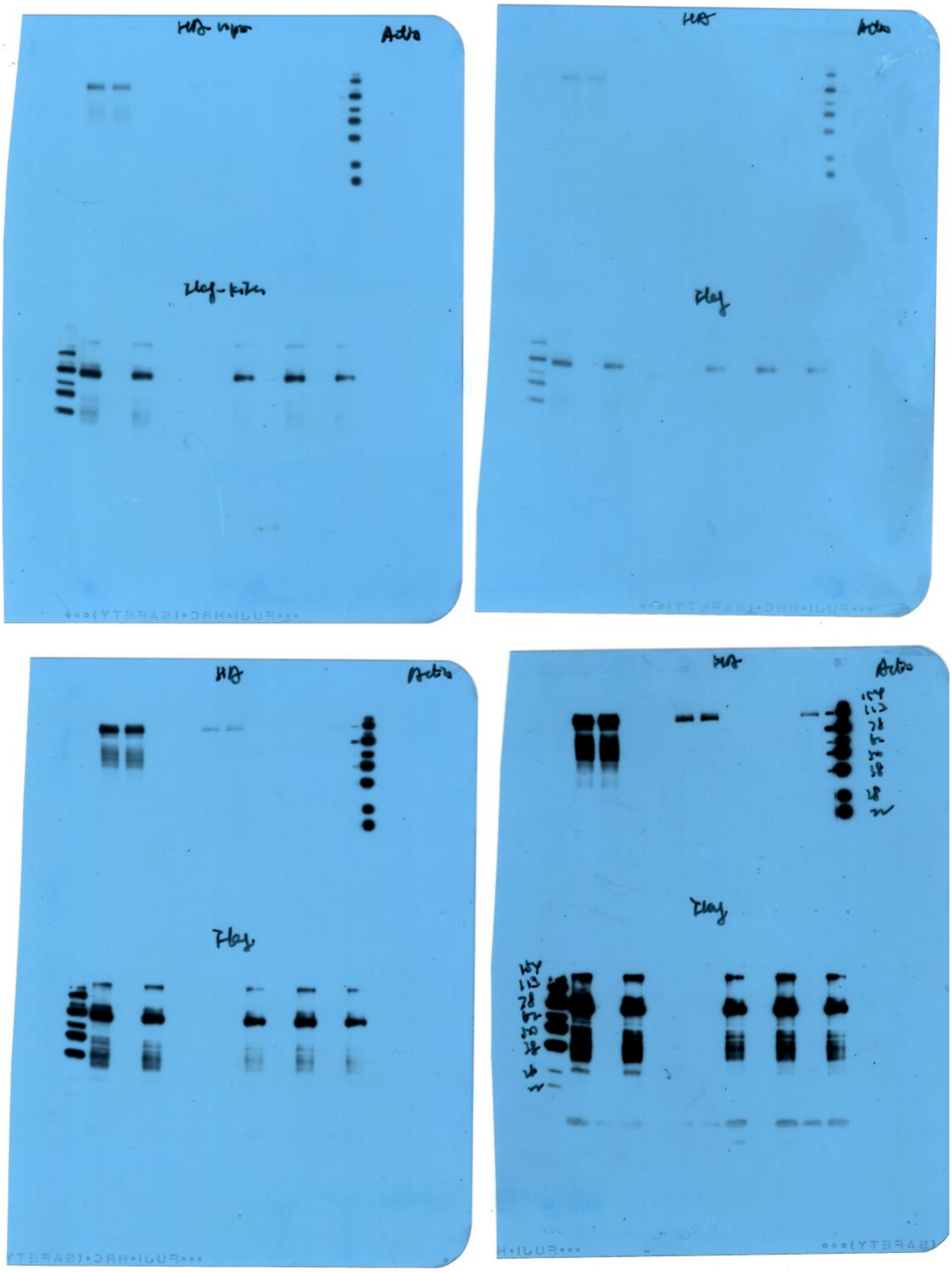

Fig4 D-E-2

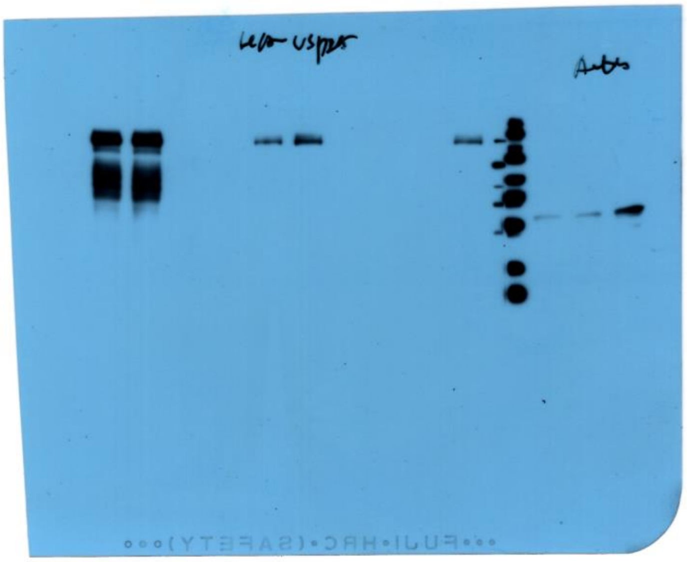

Fig4 D-E-3

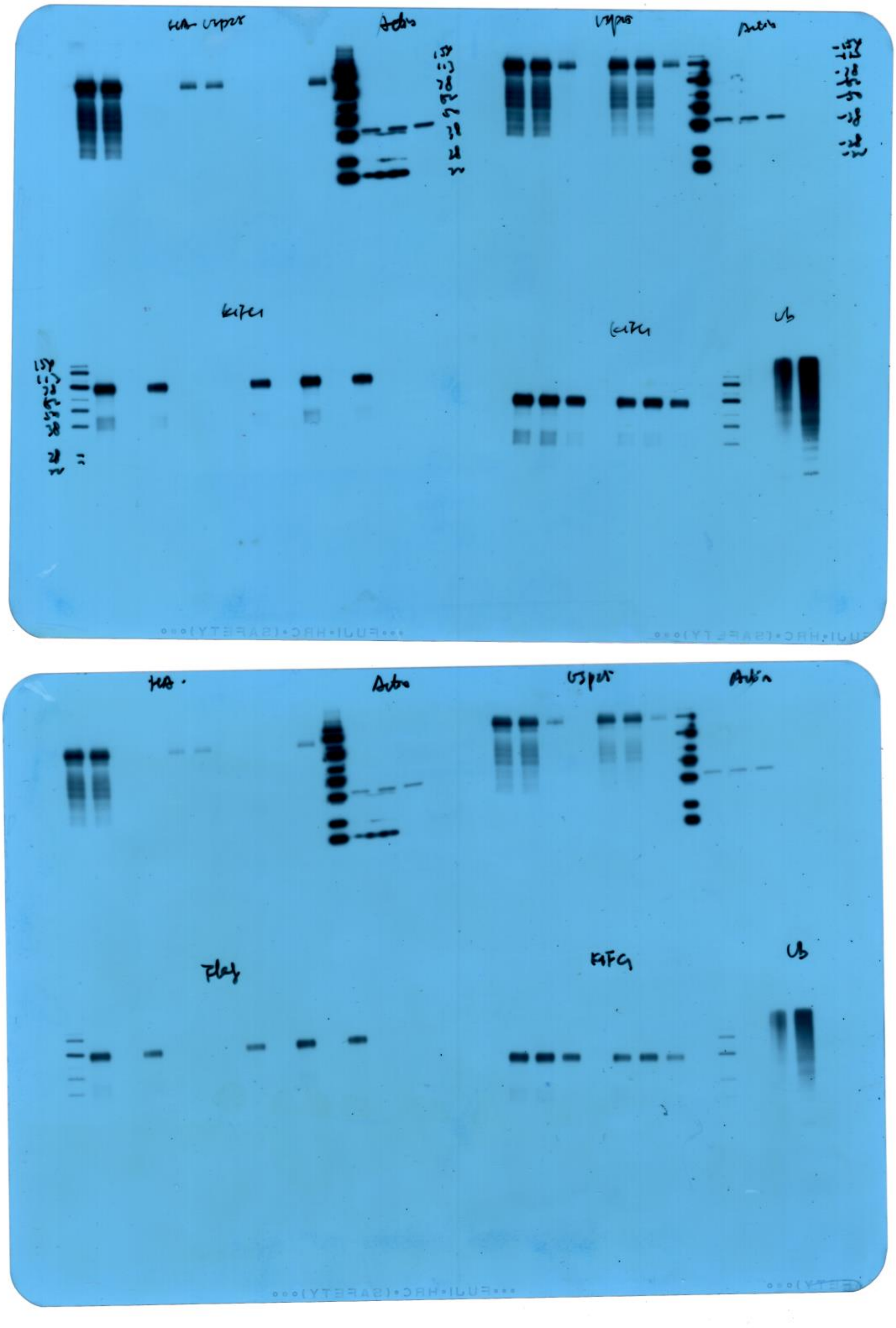

Fig4F-I-1

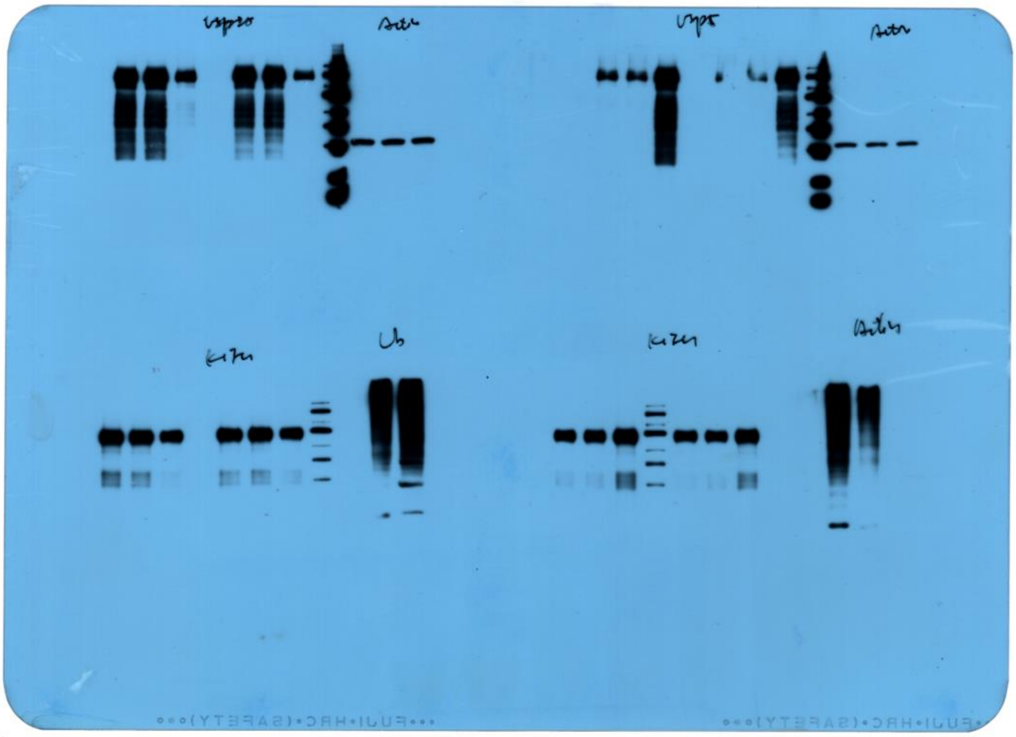

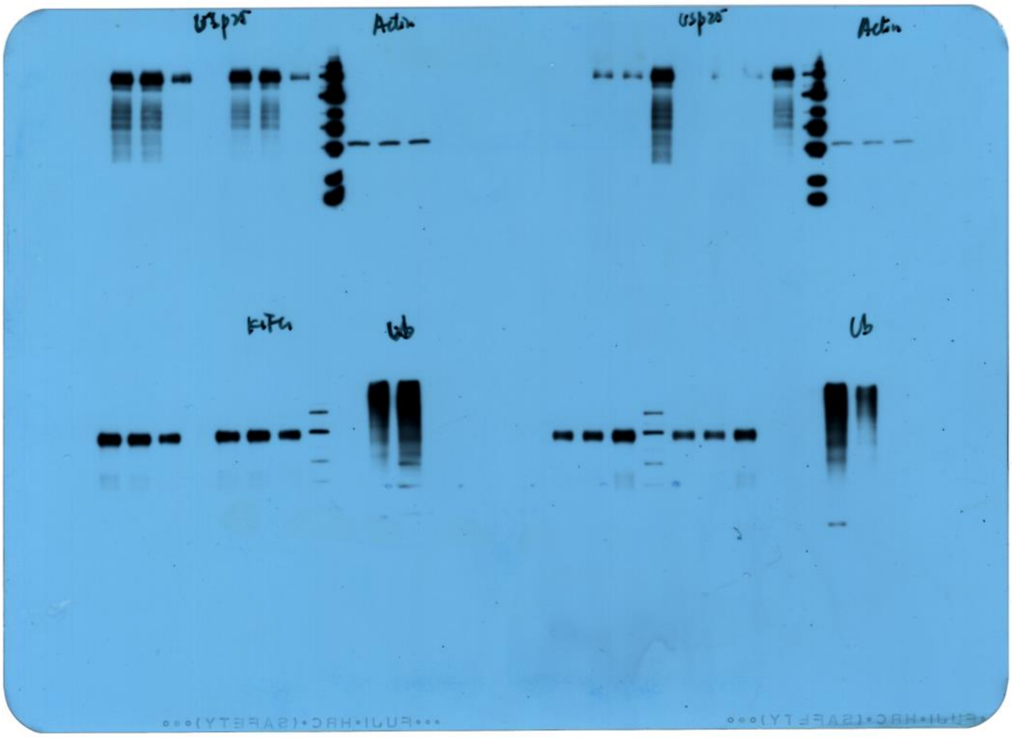

Fig4F-I-1-2

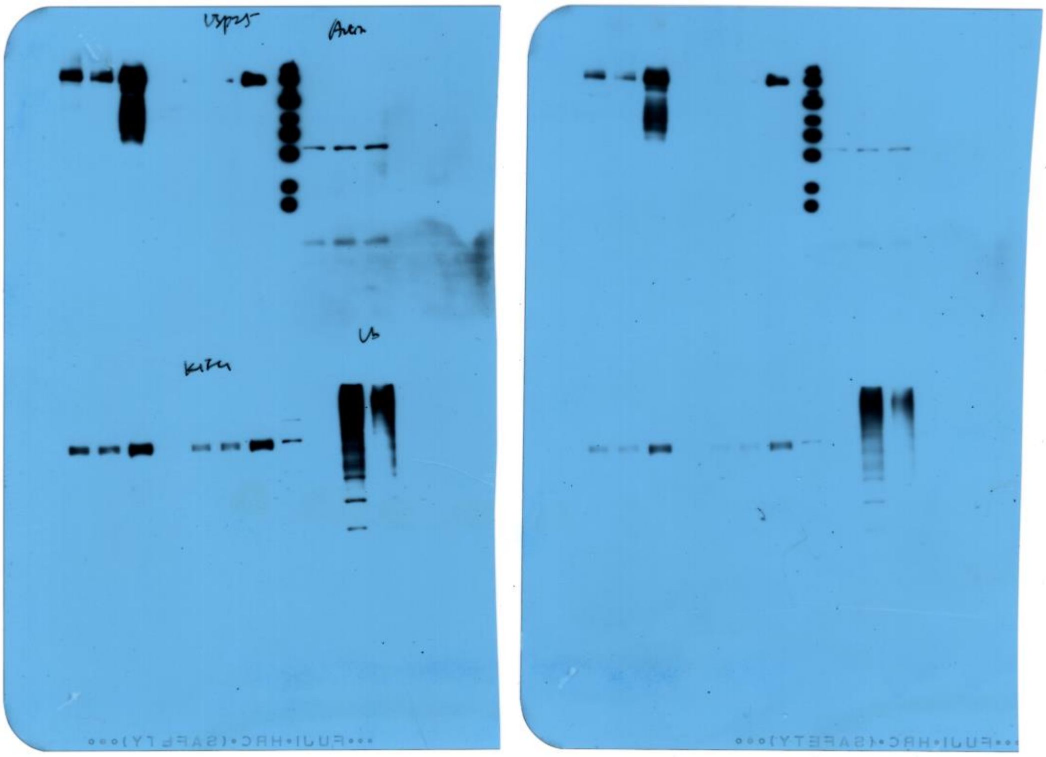

Fig4F-I-1-3

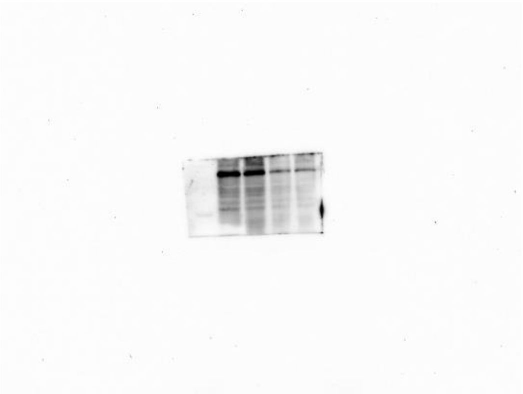

Fig5 A HeLa USP25

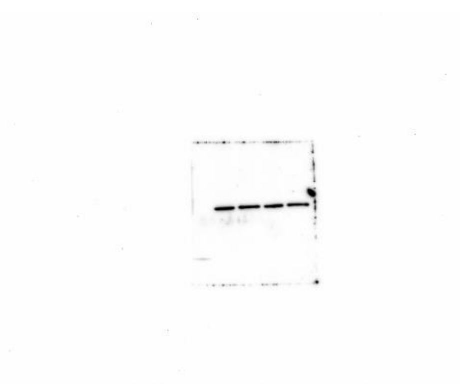

Fig5 A SiHa GAPDH

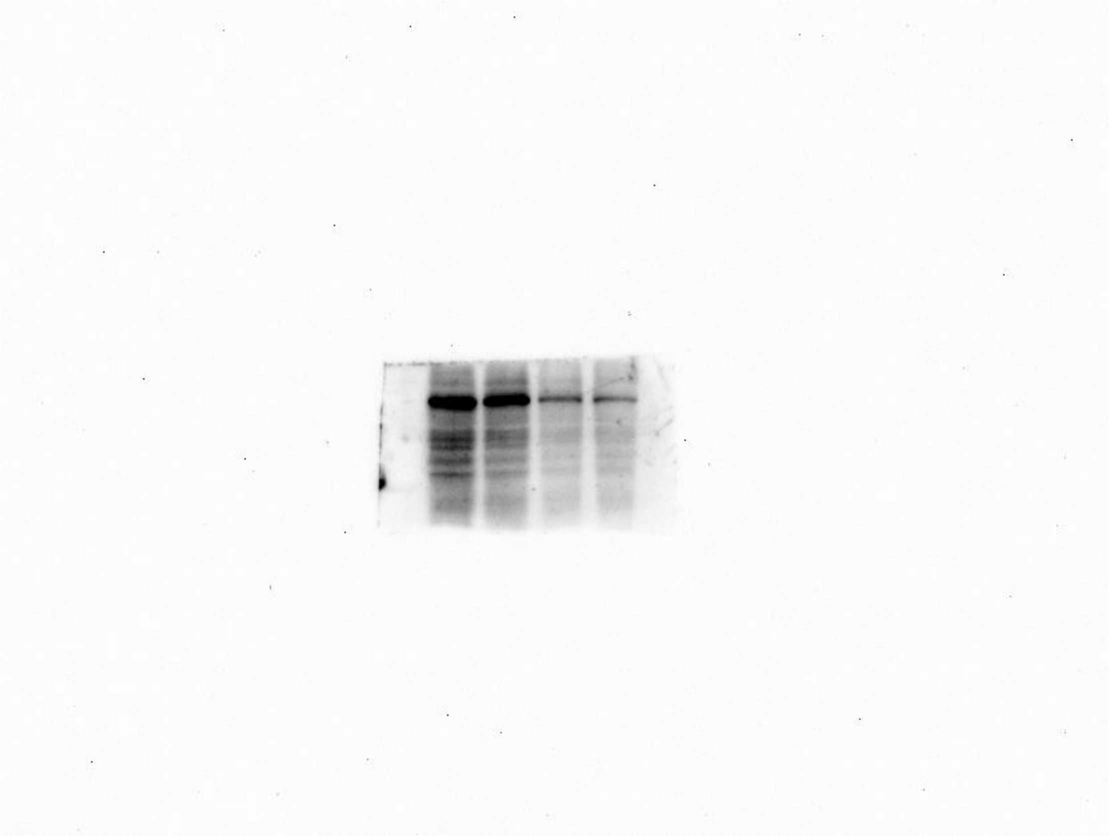

Fig5 A SiHa USP25

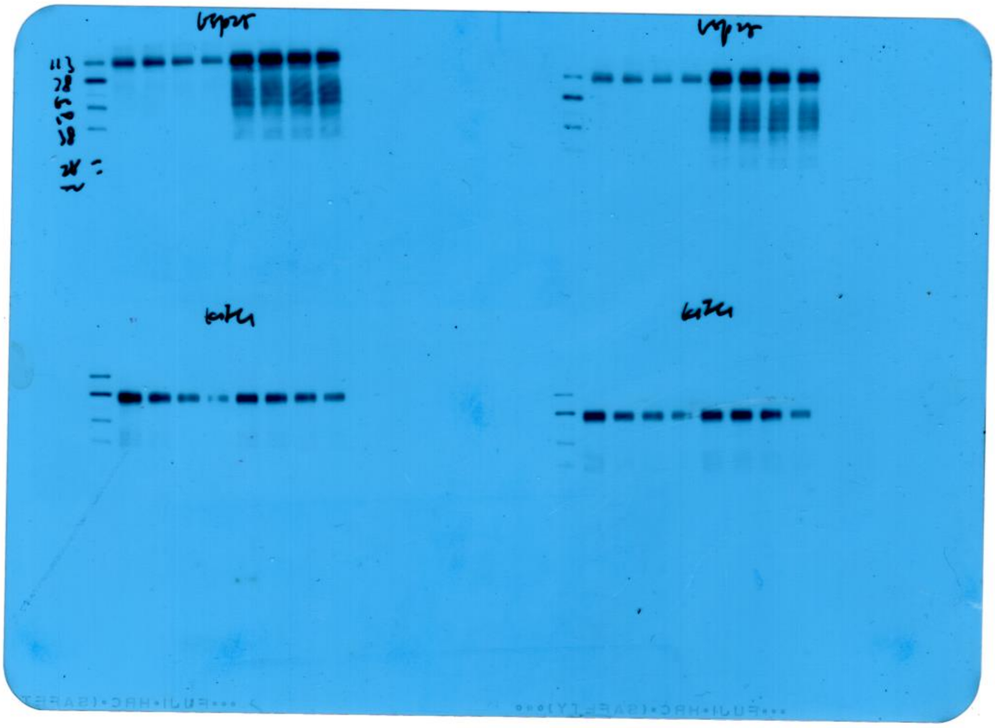

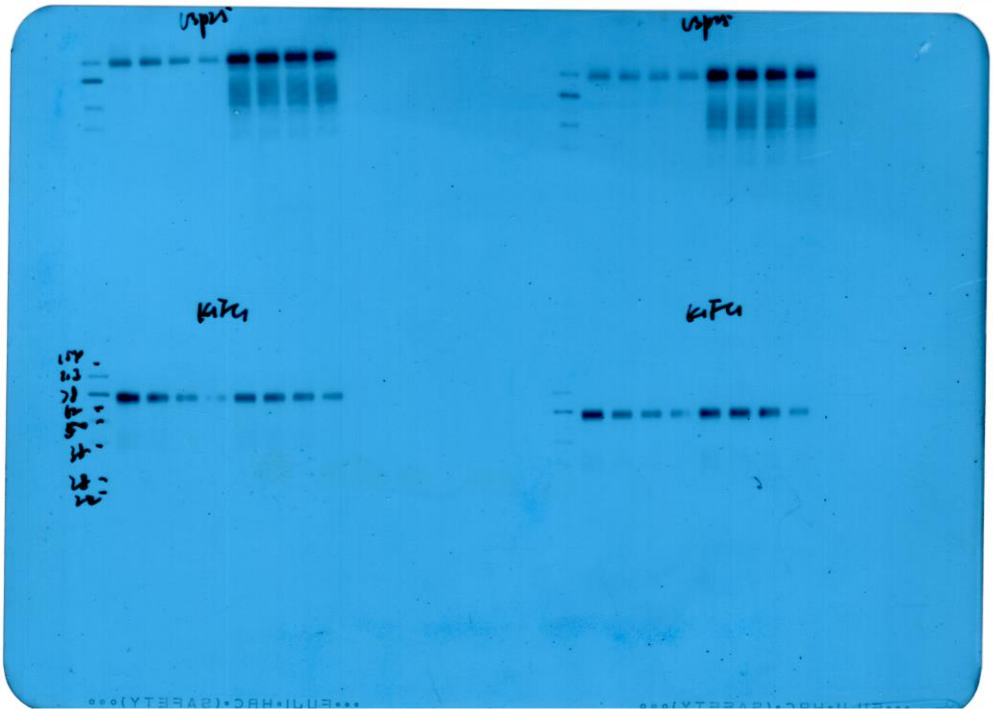

Fig5J-K

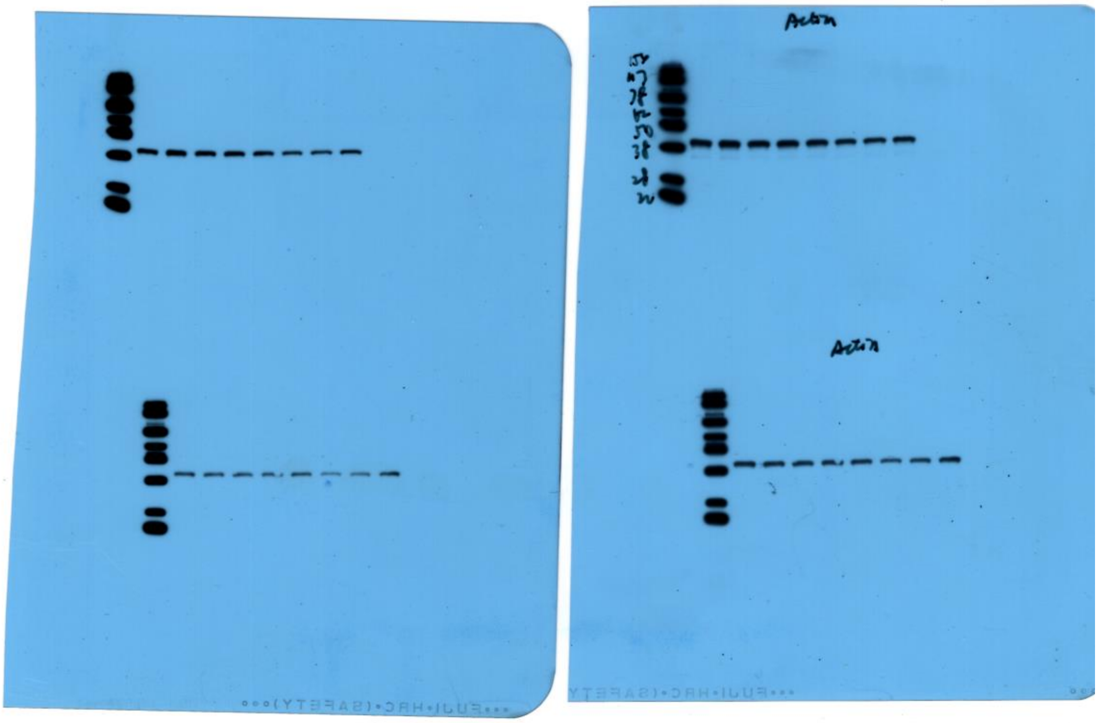

Fig5J-K-2

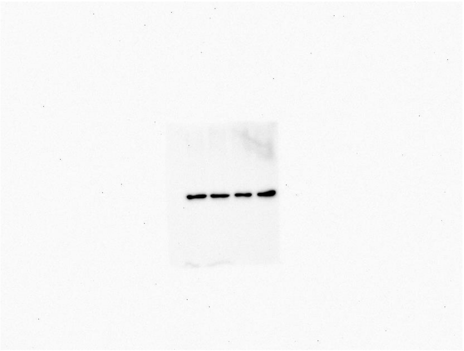

Fig7 E HaLa GAPDH

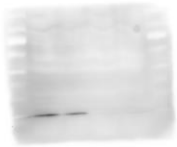

Fig7 E HaLa MYCBP

Fig7 E SiHa GAPDH

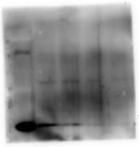

Fig7 E SiHa MYCBP

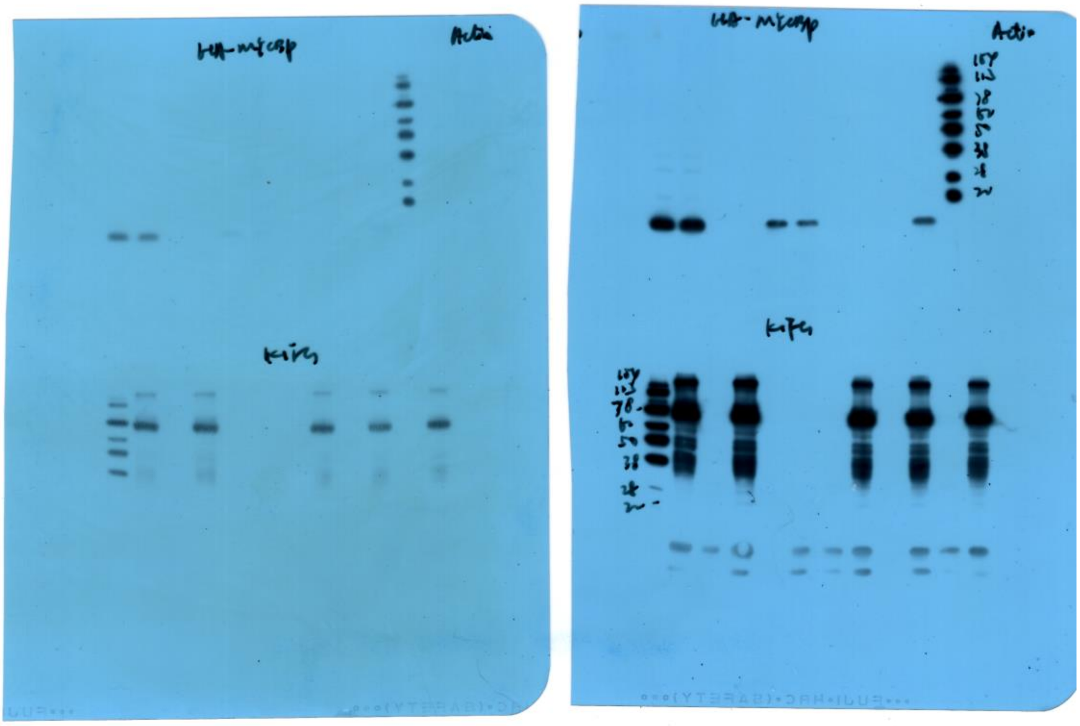

Fig7C-1

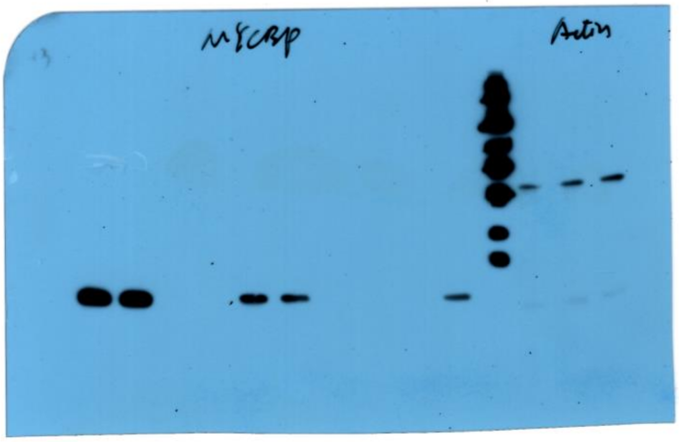

fig7C-2

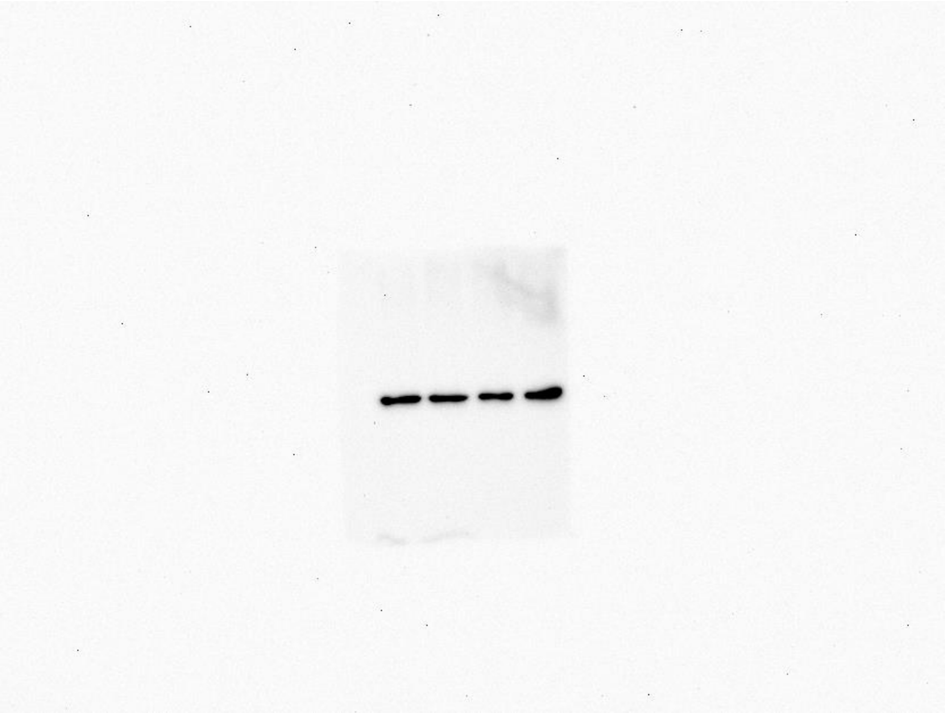

Fig7E GAPDH

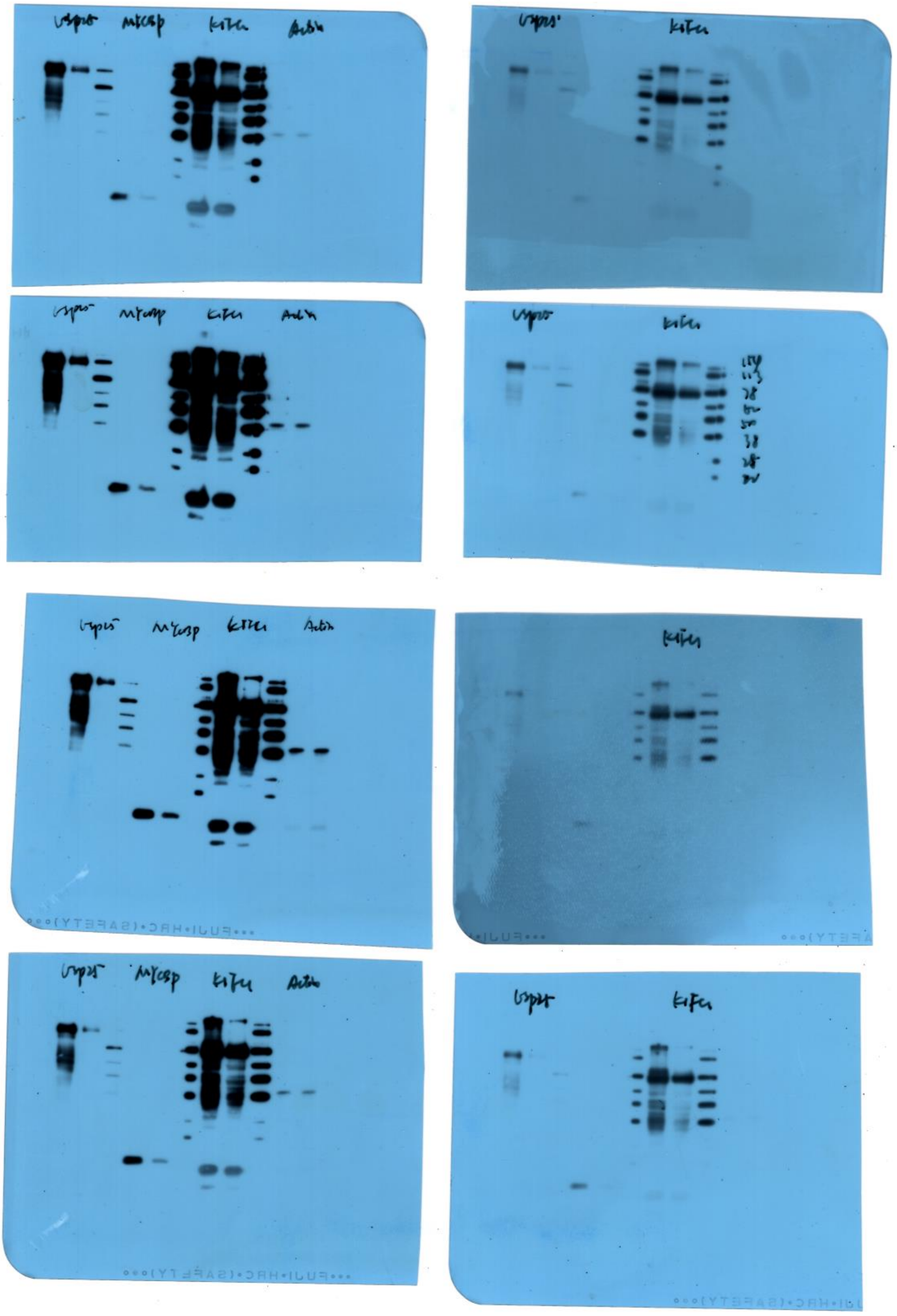

Fig8 C-D

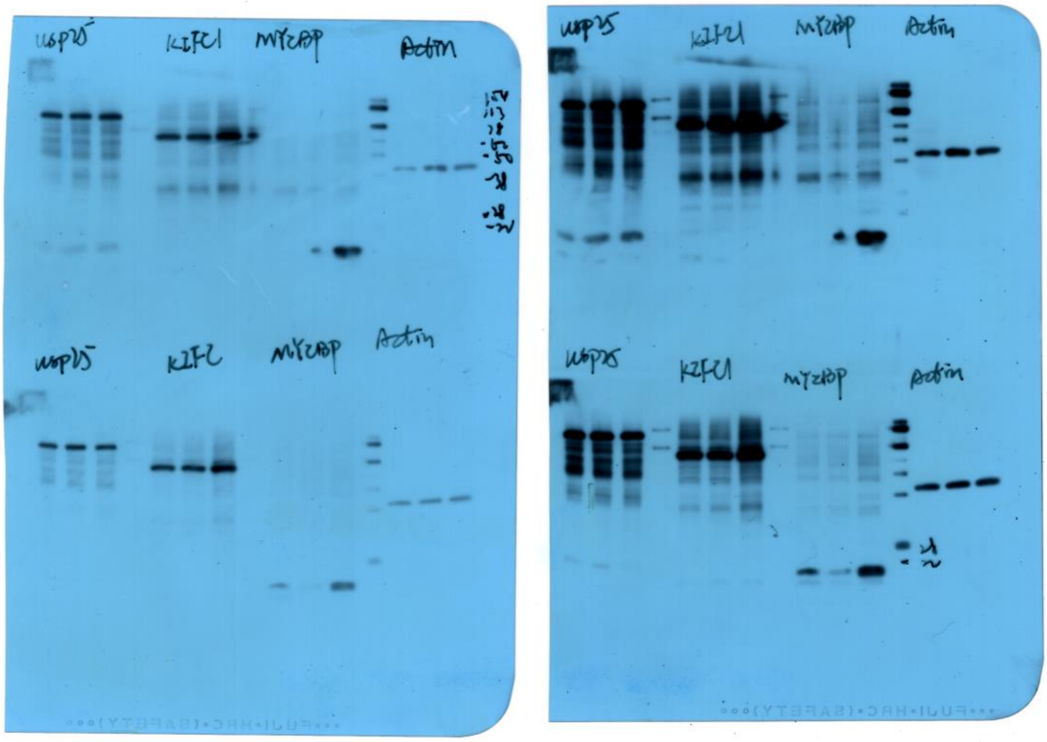

Fig8E-F

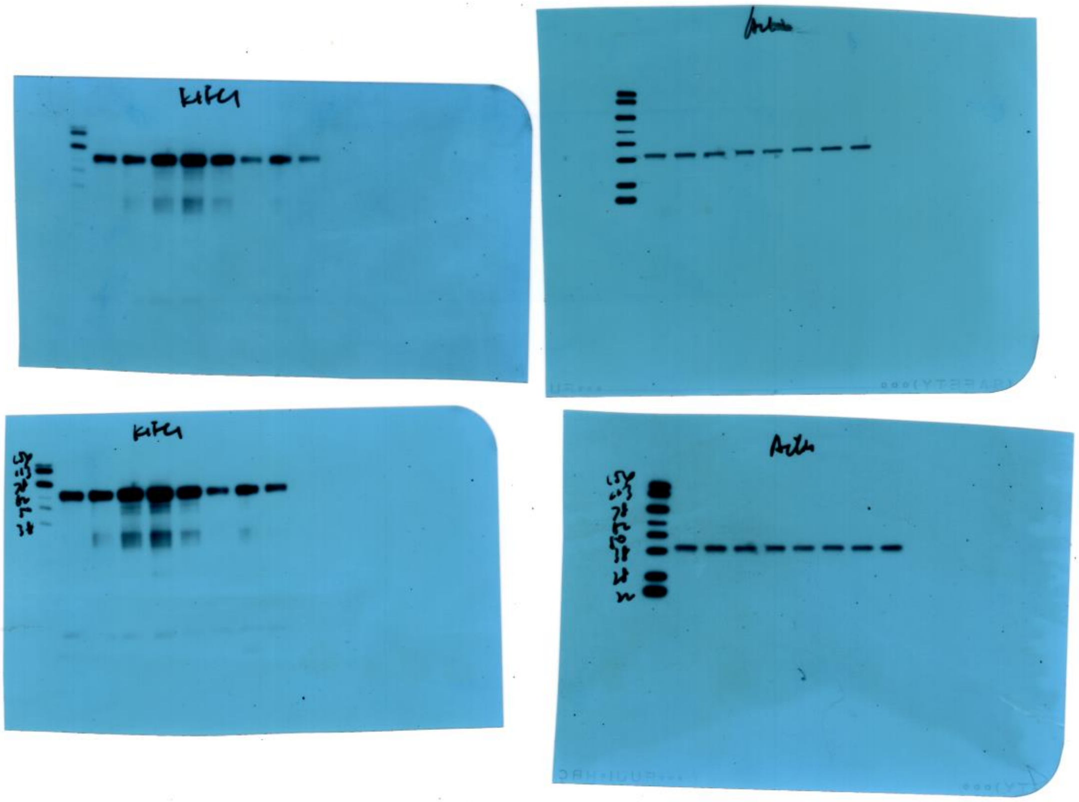

FigS2B

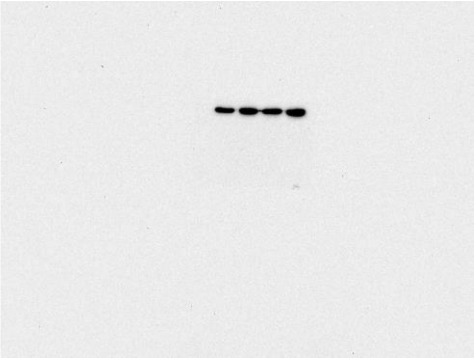

FigS3 A HeLa GAPDH

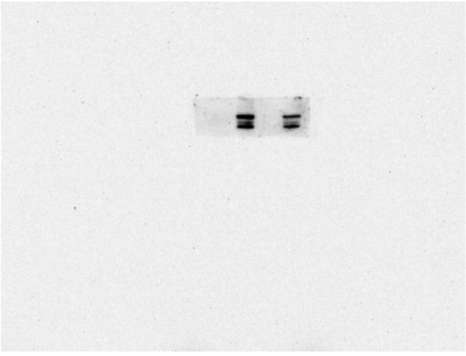

FigS3 A HeLa kifc1

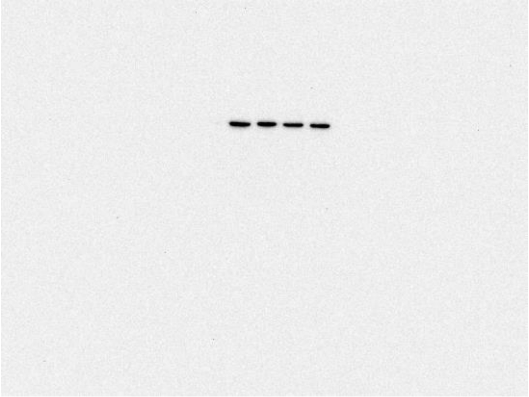

FigS3 A SIHA GAPDH

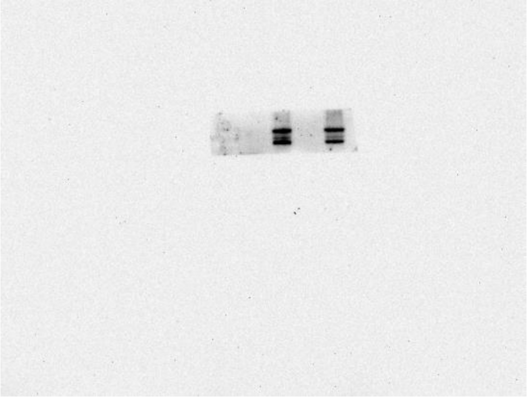

FigS3 A SIHA kifc1

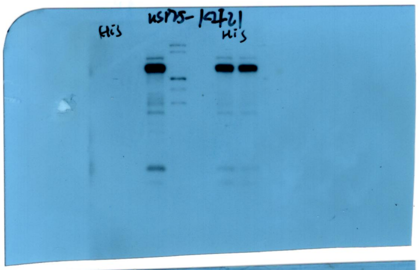
His
His

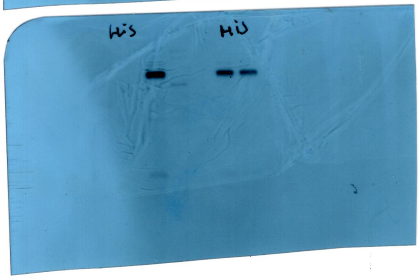
His
His

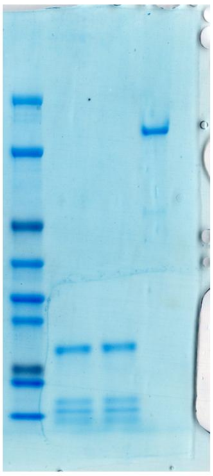

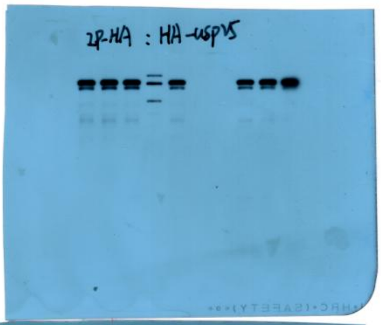
IP-HA : HA-usp75

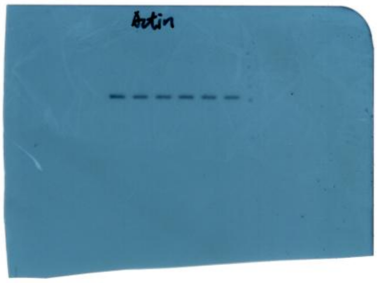
Actin

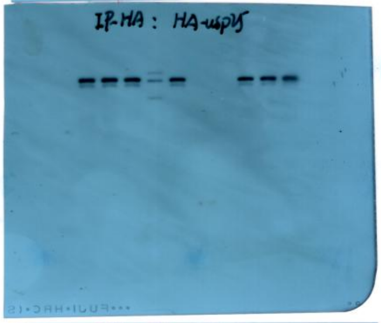
IP-HA : HA-usp75

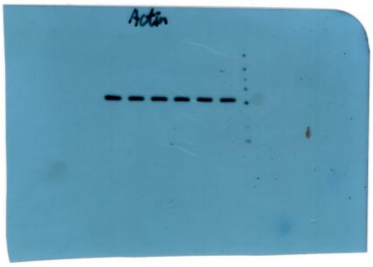
Actin

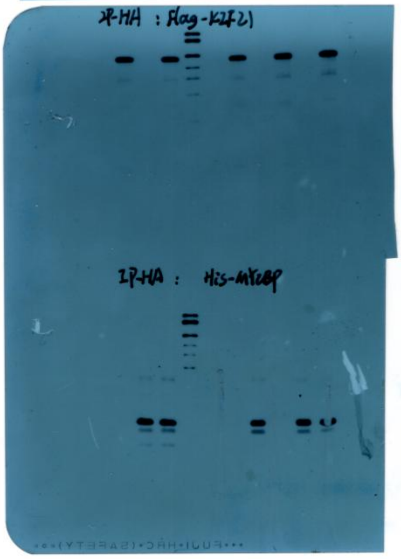
IP-HA : Flag-kzf21
IP-HA : His-MYCBP

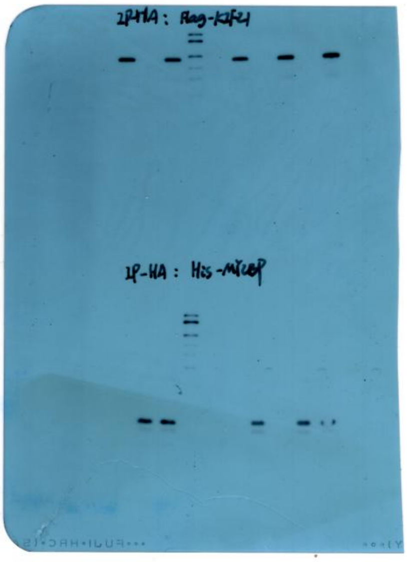
IP-HA : Flag-kzf21
IP-HA : His-MYCBP

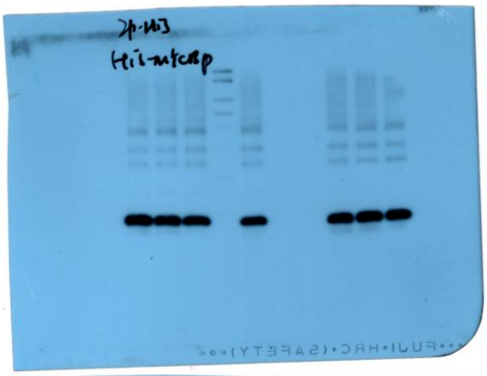

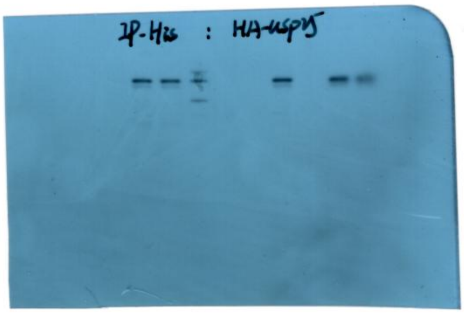

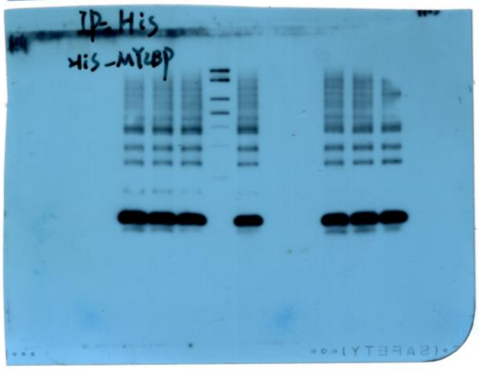
IP-His

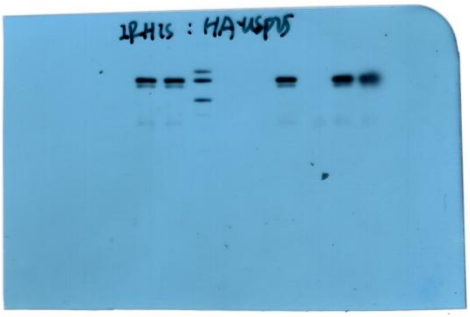

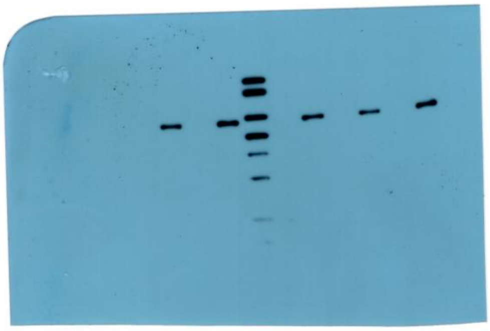

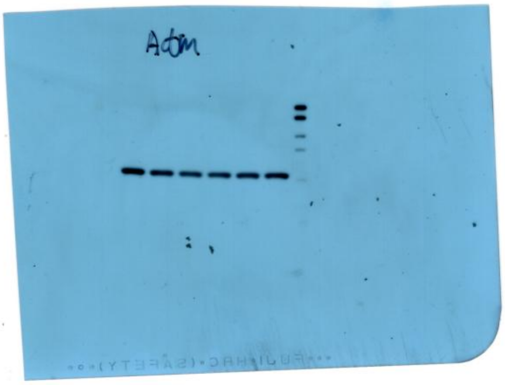
Actin

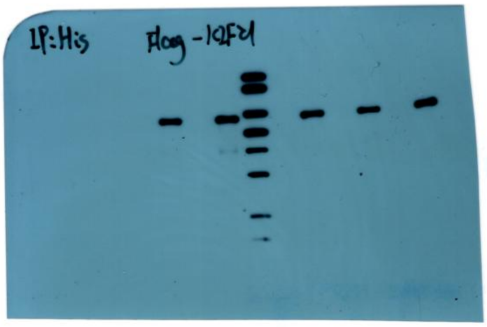
IP:His

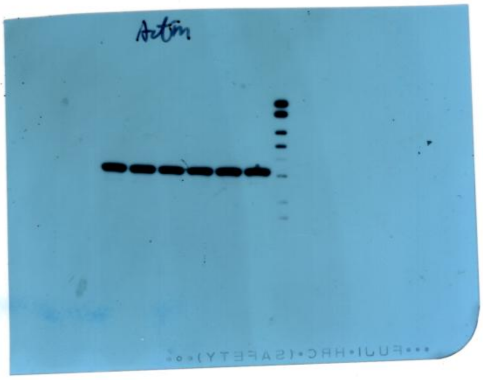
Actin

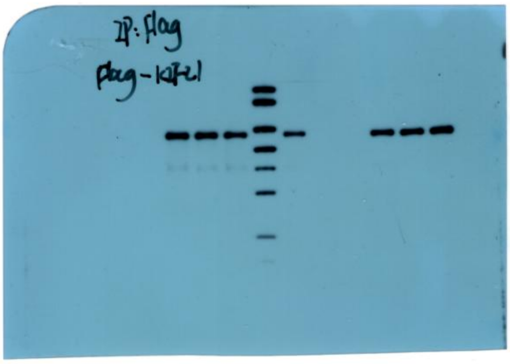
IP: Flag
Flag-KIF21

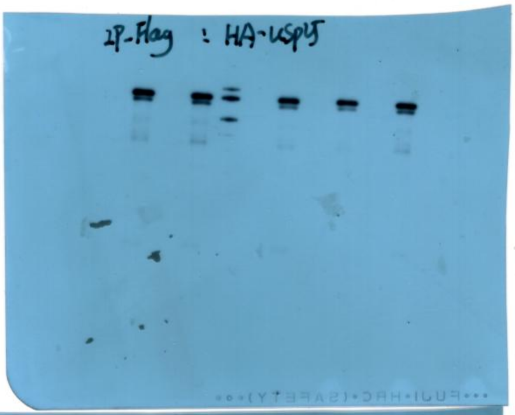
IP-Flag : HA-USP15

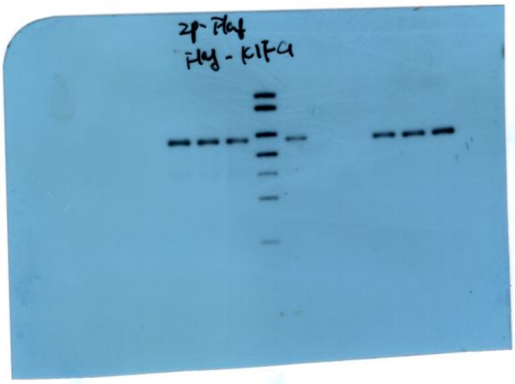
IP-Flag
Flag-KIFC1

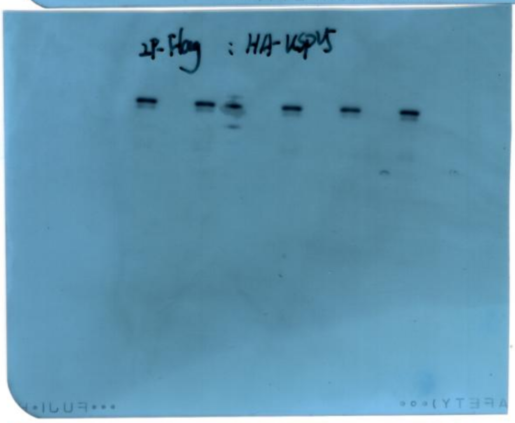
IP-Flag : HA-USP15

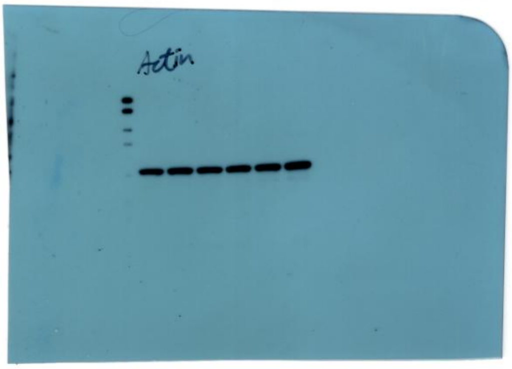
Actin

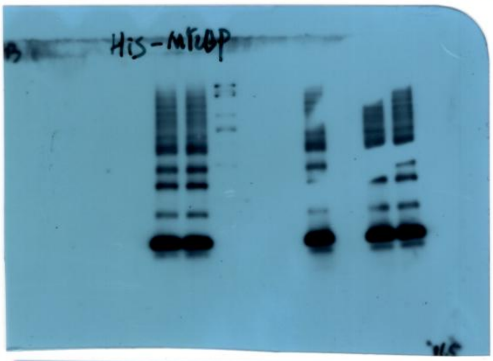
His-MYCBP

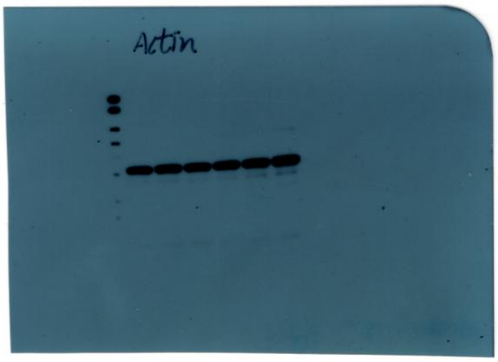
Actin

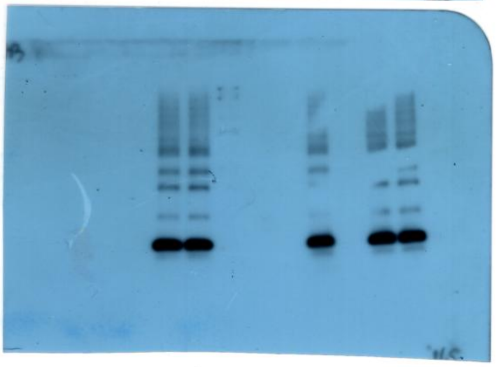

GLUT1 54kd SiHa^{shUSP25},SiHa$^{shUSP25+KIFC1\ OE}$

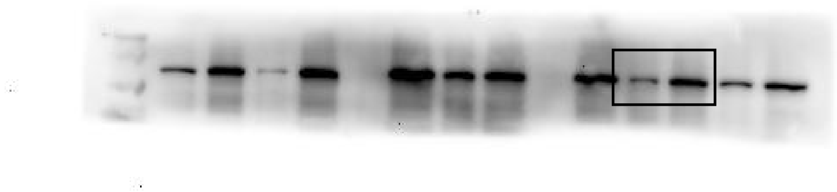

GLUT1 54kd HeLa^{shUSP25},HeLa$^{shUSP25+KIFC1\ OE}$

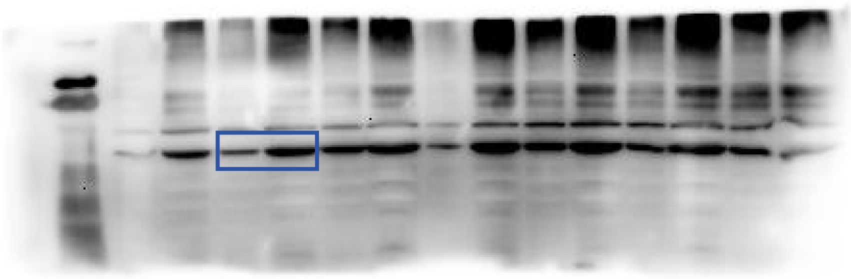

HK-2 102kd HeLa^{shUSP25},HeLa$^{shUSP25+KIFC1\ OE}$, SiHa^{shUSP25},SiHa$^{shUSP25+KIFC1\ OE}$

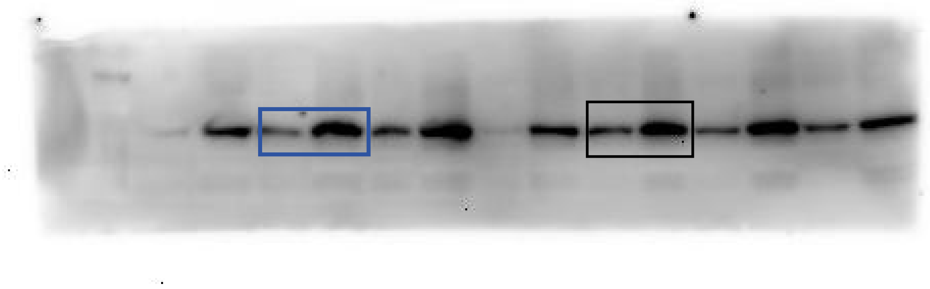

GAPDH 37KD HeLa^{shUSP25},HeLa$^{shUSP25+KIFC1\ OE}$, SiHa^{shUSP25},SiHa$^{shUSP25+KIFC1\ OE}$

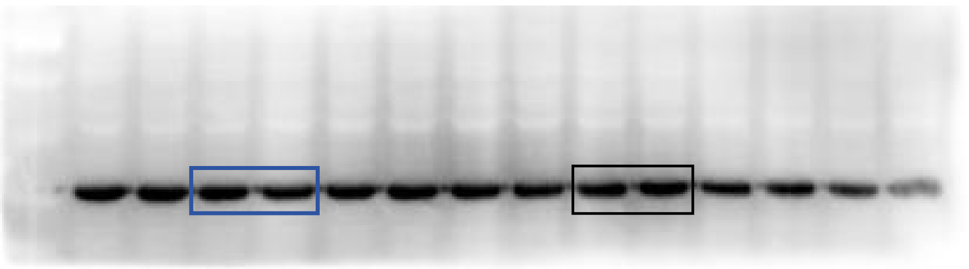

LDH-A 37kd SiHa^{shUSP25},SiHa$^{shUSP25+KIFC1\ OE}$

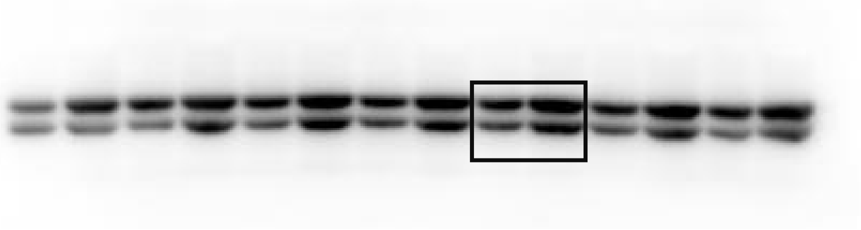

LDH-A 37kd HeLa^{shUSP25},HeLa$^{shUSP25+KIFC1\ OE}$

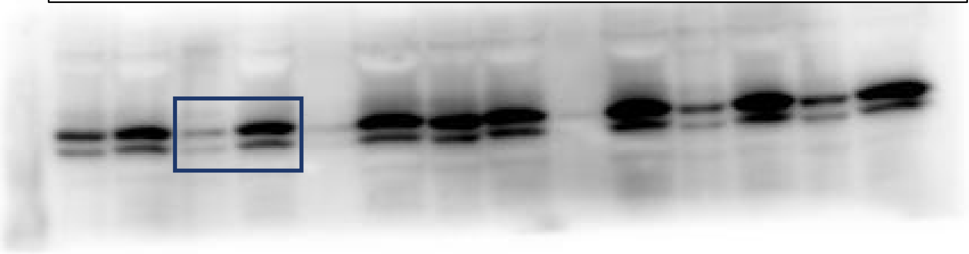

HeLa SiHa

WT kifc1-ko shNC shUSP25 shNC shMYCBP WT kifc1-ko shNC shUSP25 shNC shMYCBP

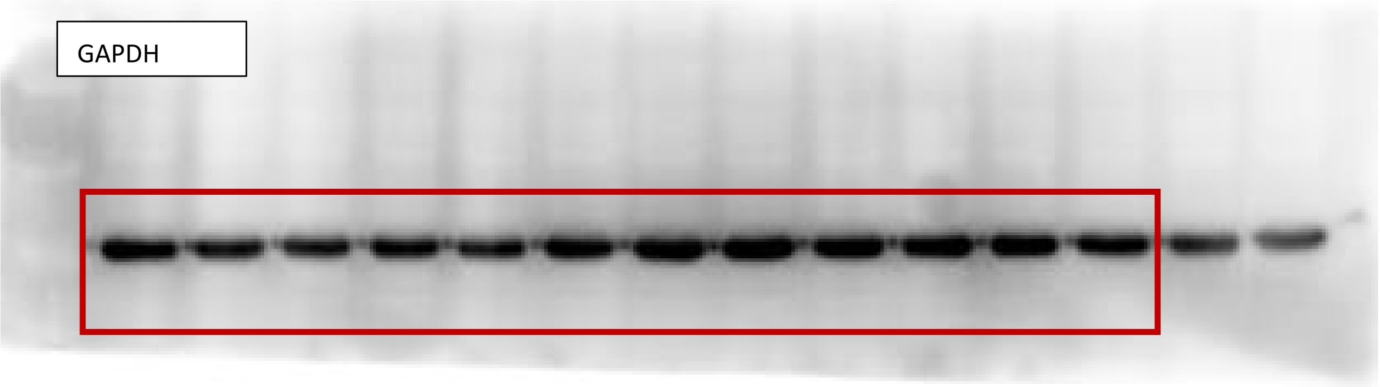

HeLa SiHa

WT kifc1-ko shNC shUSP25 shNC shMYCBP WT kifc1-ko shNC shUSP25 shNC shMYCBP

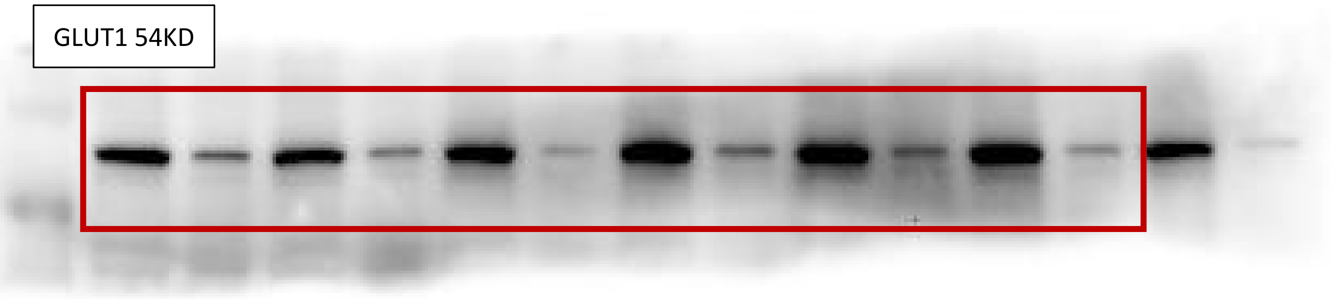

HeLa SiHa

WT kifc1-ko shNC shUSP25 shNC shMYCBP WT kifc1-ko shNC shUSP25 shNC shMYCBP

LDH-A 37KD

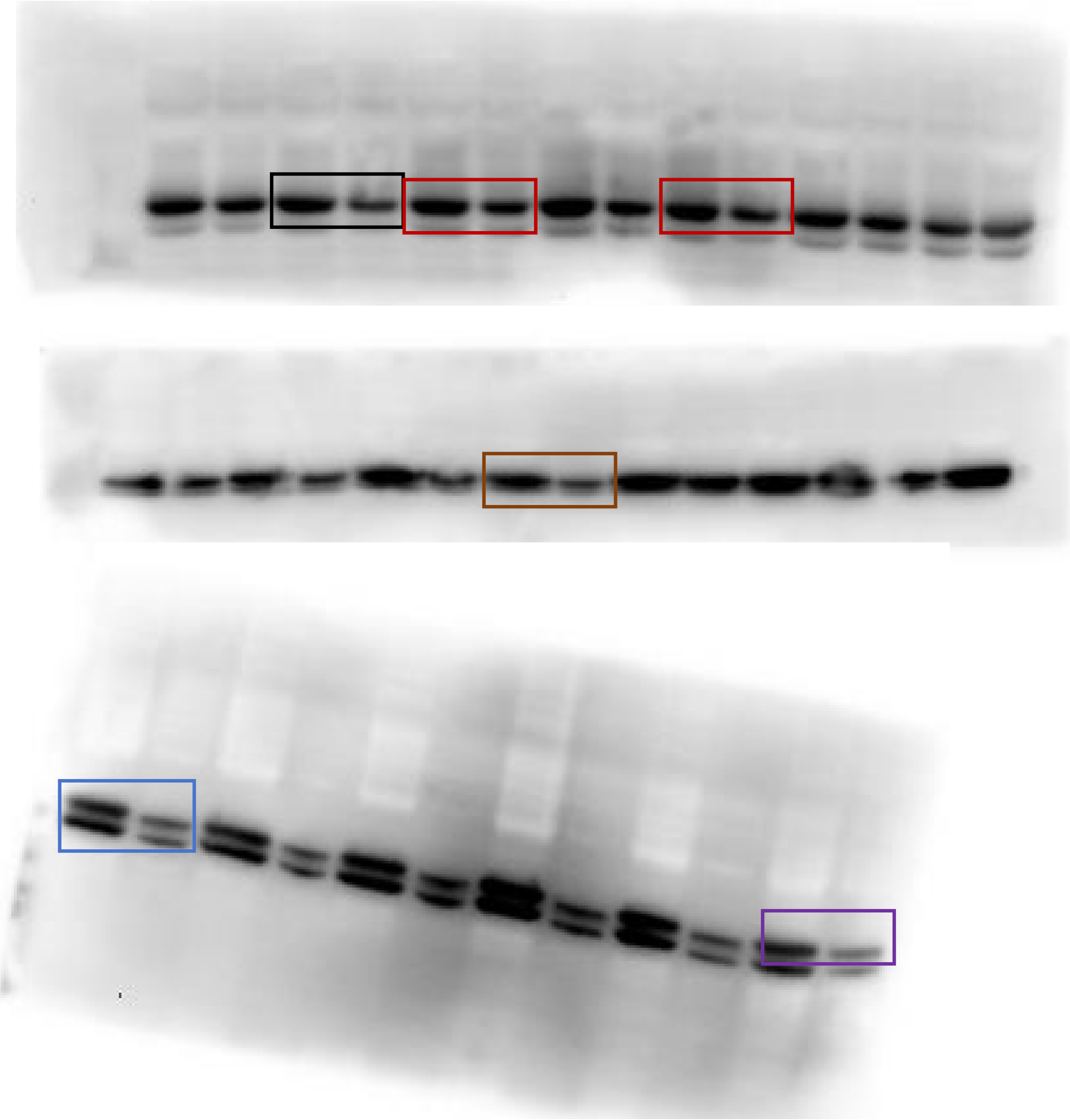

HeLa SiHa

WT kifc1-ko shNC shUSP25 shNC shMYCBP WT kifc1-ko shNC shUSP25 shNC shMYCBP

HK-2 102KD

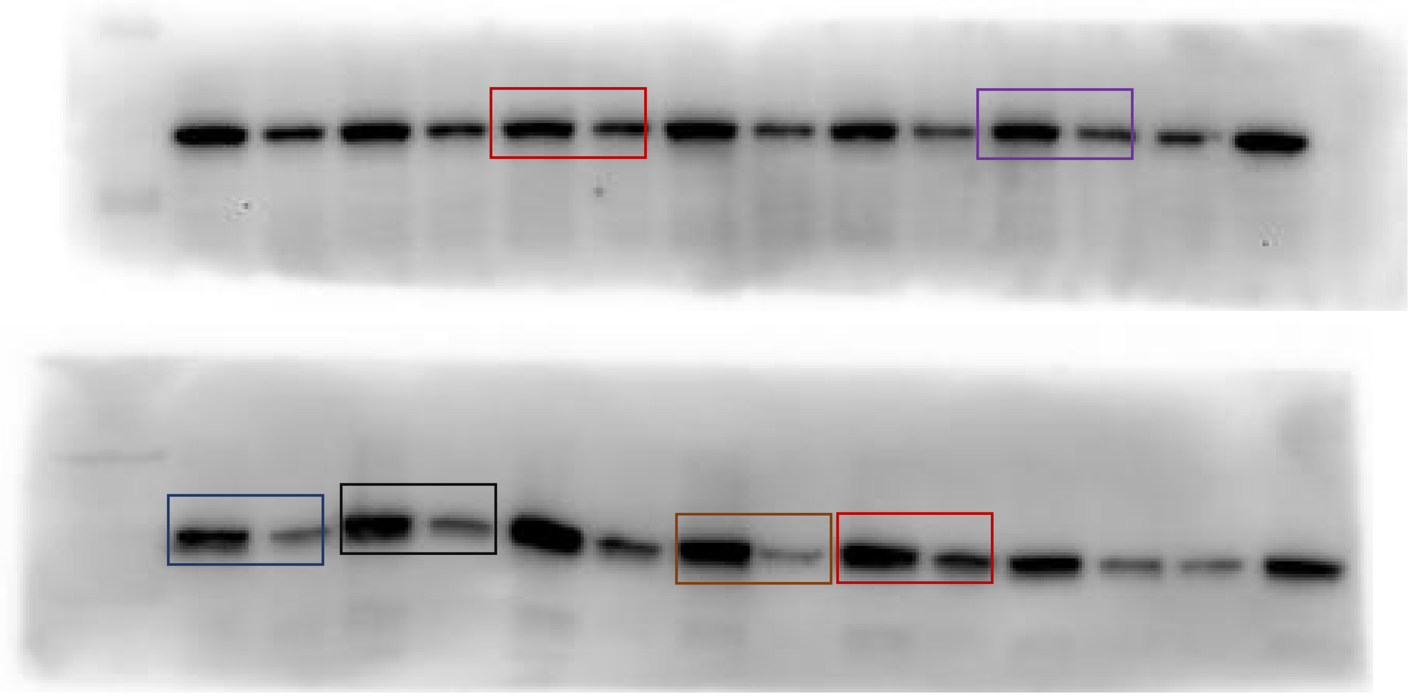

Supplement: Supplementary file 11 — Original WB images [file 41419_2025_7713_MOESM11_ESM.pdf]
