# Supplementary material for: USP25-driven KIFC1 regulates MYCBP expression and promotes the progression of cervical cancer
Source: Cell Death Dis. 2025 May 16;16(1):390. doi: 10.1038/s41419-025-07713-x (PMC12084419; doi:10.1038/s41419-025-07713-x)
Supplement: Supplementary file 12 — IP_MS [file 41419_2025_7713_MOESM12_ESM.pdf]

Accession	Gene	Descriptio	Mw(kDa)	Length	Hela-IP/He	log2 Hela-	Diff Sig	Count	Hela-IP	Hela-IgG
A6NHR9	SMCHD1	Structural r	226.374	2005	NA	NA	NA	0	8.927623	9.432231
A8MW99	MEI4	Meiosis-sp	44.024	385	NA	NA	NA	0	10.06384	10.00894
O15379	HDAC3	Histone de	48.848	428	NA	NA	NA	0	10.17426	11.16262
O60260	PRKN	E3 ubiquitii	51.641	465	NA	NA	NA	0	9.036972	9.745395
O95336	PGLS	6-phospho	27.547	258	NA	NA	NA	0	10.38353	11.69961
P16144	ITGB4	Integrin bet	202.167	1822	NA	NA	NA	0	10.23473	10.23378
P43034	PAFAH1B1	Platelet-act	46.638	410	NA	NA	NA	0	9.85984	10.63701
P60880	SNAP25	Synaptosoı	23.315	206	NA	NA	NA	0	10.10167	11.69741
Q12983	BNIP3	BCL2/aden	27.832	259	NA	NA	NA	0	9.476754	11.62749
Q14181	POLA2	DNA polym	65.948	598	NA	NA	NA	0	8.73671	10.70753
Q5JTH9	RRP12	RRP12-like	143.702	1297	NA	NA	NA	0	8.272722	10.60124
Q86SX6	GLRX5	Glutaredox	16.628	157	NA	NA	NA	0	10.17707	10.00675
Q8NEZ5	FBXO22	F-box only	44.508	403	NA	NA	NA	0	8.95291	9.926328
Q8WUA2	PPIL4	Peptidyl-pr	57.225	492	NA	NA	NA	0	10.09064	12.08402
Q8WXH0	SYNE2	Nesprin-2 (	796.442	6885	NA	NA	NA	0	8.692947	11.36519
Q92599	SEPTIN8	Septin-8 O	55.756	483	NA	NA	NA	0	9.277143	10.31246
Q92879	CELF1	CUGBP El	52.063	486	NA	NA	NA	0	9.54644	10.19191
Q96FJ2	DYNLL2	Dynein ligh	10.35	89	NA	NA	NA	0	8.905261	11.39823
Q96GG9	DCUN1D1	DCN1-like	30.124	259	NA	NA	NA	0	10.84732	10.17403
Q96JY6	PDLIM2	PDZ and L	37.459	352	NA	NA	NA	0	10.31289	10.81027
Q99426	TBCB	Tubulin-fol	27.326	244	NA	NA	NA	0	10.47468	9.549976
Q9H4B6	SAV1	Protein sal	44.634	383	NA	NA	NA	0	8.871861	10.84454
Q9H9Q2	COPS7B	COP9 sign	29.622	264	NA	NA	NA	0	9.946414	11.02326
Q9UI09	NDUFA12	NADH deh	17.114	145	NA	NA	NA	0	10.55353	9.913793
Q8ND04	SMG8	Nonsense-	109.684	991	3821.338	11.89986	+	1	21.80396	9.904102
Q9BW19	KIFC1	Kinesin-like	73.748	673	2877.906	11.4908	+	1	20.32323	8.832422
Q99417	MYCBP	c-Myc-bind	11.967	103	1418.995	10.47065	+	1	20.41214	9.94149
Q00587	CDC42EP1	Cdc42 effe	40.295	391	1332.544	10.37997	+	1	18.7693	8.389334
Q9NPI6	DCP1A	mRNA-dec	63.278	582	1165.071	10.1862	+	1	19.40736	9.221157
Q9NRG9	AAAS	Aladin OS=	59.574	546	907.79	9.826215	+	1	20.52736	10.70114
Q9BSA4	TTYH2	Protein twe	58.772	534	672.0601	9.392446	+	1	19.96466	10.57221
Q9UJA5	TRMT6	tRNA (ader	55.799	497	423.4699	8.726116	+	1	18.61324	9.887127
Q6PJG2	MIDEAS	Mitotic dea	114.989	1045	402.5749	8.653114	+	1	18.89826	10.24515
Q8WZ42	TTN	Titin OS=H	3816.03	34350	372.8324	8.542383	+	1	18.32917	9.786791
Q01804	OTUD4	OTU doma	124.045	1114	345.318	8.431782	+	2	19.51526	11.08348
Q9H098	FAM107B	Protein FAI	15.558	131	303.2547	8.244386	+	1	17.62214	9.377752
Q9UBL3	ASH2L	Set1/Ash2	68.723	628	279.9009	8.128773	+	1	17.6933	9.564524
Q9BT78	COPS4	COP9 sign	46.269	406	272.5553	8.090405	+	1	18.08654	9.996135
Q5TA89	HES5	Transcripti	18.226	166	219.5141	7.77817	+	1	18.44435	10.66618
Q9NVM6	DNAJC17	DnaJ homo	34.687	304	218.8038	7.773494	+	1	18.30977	10.53628
Q6WKZ4	RAB11FIP1	Rab11 fam	137.167	1283	215.0158	7.748299	+	2	22.49497	14.74667
Q96F86	EDC3	Enhancer c	56.078	508	203.1121	7.666133	+	1	18.9416	11.27547
Q9H223	EHD4	EH domain	61.175	541	179.7383	7.489754	+	2	17.79011	10.30035
P61964	WDR5	WD repeat	36.588	334	177.0921	7.468356	+	2	18.21722	10.74886
Q14686	NCOA6	Nuclear rec	219.145	2063	168.7337	7.398604	+	2	18.6145	11.2159
Q15291	RBBP5	Retinoblast	59.153	538	164.7459	7.364099	+	1	17.50711	10.14301
Q9UPT5	EXOC7	Exocyst co	83.382	735	159.0652	7.313474	+	1	18.9846	11.67112
Q6S8J3	POTEE	POTE anky	121.363	1075	154.7019	7.273348	+	1	17.37135	10.098
Q8N554	ZNF276	Zinc finger	67.219	614	121.8241	6.928656	+	1	17.22938	10.30073
Q9UJW0	DCTN4	Dynactin su	52.337	460	116.4192	6.863186	+	1	16.74202	9.878834
Q16401	PSMD5	26S protea	56.196	504	110.1047	6.782732	+	1	16.89036	10.10763
Q9C005	DPY30	Protein dpy	11.25	99	105.7707	6.724797	+	1	17.37814	10.65335
P55735	SEC13	Protein SE	35.541	322	102.211	6.675407	+	1	17.5998	10.92439
A0FGR8	ESYT2	Extended s	102.357	921	101.8156	6.669815	+	1	15.21512	8.545301
Q92738	USP6NL	USP6 N-te	94.104	828	93.46163	6.546302	+	2	17.53108	10.98477
P11234	RALB	Ras-relatec	23.409	206	89.91131	6.490431	+	1	16.49021	9.999778
O15047	SETD1A	Histone-lys	186.034	1707	89.35666	6.481503	+	1	16.6952	10.2137
Q6IBS0	TWF2	Twinfilin-2	39.548	349	84.00834	6.392461	+	1	15.91833	9.525867

P30049	ATP5F1D	ATP syntha	17.49	168	82.58916	6.367881	+	1	16.02382	9.655939
Q9P0U4	CXXC1	CXXC-type	75.712	656	80.37071	6.328598	+	1	16.71116	10.38256
Q15436	SEC23A	Protein trar	86.161	765	80.03552	6.322568	+	1	15.60342	9.280855
Q8NFC6	BOD1L1	Biorientatic	330.466	3051	79.65137	6.315627	+	1	16.45292	10.13729
O43290	SART1	U4/U6.U5 t	90.255	800	79.22089	6.307809	+	1	16.36436	10.05655
O43422	THAP12	52 kDa rep	87.704	761	75.67225	6.241692	+	1	16.3689	10.12721
Q7L2E3	DHX30	ATP-deper	133.938	1194	75.13781	6.231467	+	1	15.82187	9.590406
Q9BYX2	TBC1D2	TBC1 dom	105.414	928	74.87714	6.226453	+	1	16.88238	10.65593
Q9H147	DNTTIP1	Deoxynucle	37.013	329	74.42345	6.217685	+	2	17.80171	11.58402
Q8N3R9	PALS1	Protein PAI	77.294	675	72.06743	6.171275	+	1	15.49007	9.318792
Q96FS4	SIPA1	Signal-indu	112.149	1042	71.98939	6.169712	+	2	16.53549	10.36578
P19971	TYMP	Thymidine	49.955	482	71.93319	6.168586	+	1	16.31006	10.14148
P61163	ACTR1A	Alpha-cent	42.614	376	71.42609	6.158379	+	1	16.81065	10.65227
O00151	PDLIM1	PDZ and L	36.072	329	69.74403	6.123998	+	1	15.84703	9.723035
Q99878	H2AC14	Histone H2	13.936	128	68.95185	6.107517	+	2	16.95481	10.84729
P51610	HCFC1	Host cell fa	208.732	2035	67.44167	6.075568	+	1	16.86036	10.78479
Q13509	TUBB3	Tubulin bet	50.433	450	63.12548	5.980151	+	1	15.95878	9.978629
Q10570	CPSF1	Cleavage a	160.884	1443	61.54008	5.943454	+	1	16.61281	10.66936
Q9UNK0	STX8	Syntaxin-8	26.907	236	61.1206	5.933587	+	1	15.38822	9.454632
O75182	SIN3B	Paired amp	133.066	1162	60.99126	5.930531	+	2	15.57946	9.648933
P49757	NUMB	Protein nur	70.804	651	60.10289	5.909363	+	1	15.95948	10.05012
Q9BQA1	WDR77	Methylosor	36.724	342	59.69104	5.899443	+	1	15.35638	9.456941
P18615	NELFE	Negative el	43.24	380	58.40936	5.868128	+	1	16.62085	10.75272
O75475	PSIP1	PC4 and S	60.103	530	58.29763	5.865365	+	1	16.44595	10.58059
Q9Y520	PRRC2C	Protein PR	316.911	2896	58.1033	5.860548	+	1	15.48022	9.619674
O00193	SMAP	Small acidi	20.333	183	58.09473	5.860335	+	1	16.30953	10.44919
Q8NDX5	PHC3	Polyhomec	106.162	983	57.63219	5.848803	+	1	15.89344	10.04464
P42025	ACTR1B	Beta-centra	42.293	376	56.19704	5.812422	+	1	14.88417	9.071748
A6NHL2	TUBAL3	Tubulin alp	49.909	446	56.19042	5.812252	+	1	15.65329	9.841041
Q16527	CSRP2	Cysteine ai	20.954	193	55.26135	5.788199	+	1	14.87181	9.08361
Q9Y2W1	THRAP3	Thyroid hoi	108.666	955	54.7882	5.775793	+	1	15.75167	9.975881
O43169	CYB5B	Cytochrom	16.695	150	54.30625	5.763046	+	1	14.49922	8.736177
Q09028	RBBP4	Histone-bir	47.656	425	53.74605	5.748087	+	1	16.57949	10.83141
P01116	KRAS	GTPase KI	21.656	189	53.35268	5.737489	+	1	16.64597	10.90848
Q9H307	PNN	Pinin OS=I	81.628	717	52.98437	5.727495	+	1	15.19052	9.463024
Q14112	NID2	Nidogen-2	151.254	1375	52.81259	5.72281	+	1	15.77443	10.05162
Q9NSY1	BMP2K	BMP-2-indu	129.172	1161	52.20968	5.706245	+	2	16.88715	11.1809
P61421	ATP6V0D1	V-type prot	40.329	351	51.63216	5.690198	+	1	15.89955	10.20935
Q9BY89	KIAA1671	Uncharacte	196.711	1806	51.45104	5.685128	+	2	16.20535	10.52023
Q8NE71	ABCF1	ATP-bindin	95.926	845	51.10903	5.675506	+	2	16.41495	10.73944
O60841	EIF5B	Eukaryotic	138.827	1220	51.01983	5.672986	+	1	15.04243	9.369443
P51665	PSMD7	26S protea	37.025	324	50.83453	5.667737	+	1	15.26821	9.600476
A4D1S0	KLRG2	Killer cell le	42.851	409	50.62714	5.661839	+	1	17.06645	11.40461
Q9P2I0	CPSF2	Cleavage a	88.487	782	50.20956	5.64989	+	1	17.25183	11.60194
Q14011	CIRBP	Cold-induci	18.648	172	49.99277	5.643648	+	1	14.6696	9.025957
Q93052	LPP	Lipoma-pre	65.746	612	49.35379	5.625089	+	1	15.09556	9.470473
O15027	SEC16A	Protein trar	251.894	2357	48.83076	5.609718	+	1	15.93781	10.32809
Q00537	CDK17	Cyclin-depe	59.582	523	48.71156	5.606192	+	1	15.45163	9.845437
Q06210	GFPT1	Glutamine-	78.806	699	48.63944	5.604055	+	1	14.69746	9.093402
Q709C8	VPS13C	Vacuolar pi	422.39	3753	48.46868	5.598981	+	1	17.24804	11.64906
Q9BVA1	TUBB2B	Tubulin bet	49.953	445	47.19869	5.560675	+	1	16.11789	10.55721
Q9UKN8	GTF3C4	General tra	91.982	822	47.19124	5.560447	+	1	15.04827	9.487827
A1L390	PLEKHG3	Pleckstrin I	134.412	1219	46.66746	5.544345	+	1	16.32145	10.77711
O75821	EIF3G	Eukaryotic	35.611	320	46.36949	5.535104	+	1	15.5864	10.0513
Q9BZR6	RTN4R	Reticulon-4	50.708	473	45.6234	5.511702	+	1	15.23657	9.724873
Q96FF7	MISP3	Uncharacte	24.028	219	45.61927	5.511571	+	1	15.91614	10.40456
Q9Y5K6	CD2AP	CD2-assoc	71.451	639	45.58001	5.510329	+	1	14.65525	9.144922
Q12797	ASPH	Aspartyl/as	85.863	758	45.38912	5.504275	+	1	15.54629	10.04202
Q6P1M3	LLGL2	LLGL scrib	113.448	1020	45.38565	5.504164	+	1	14.85092	9.346755

Q8TF72	SHROOM3	Protein Shr	216.857	1996	45.04899	5.493423	+	1	15.82846	10.33504
P24666	ACP1	Low molec	18.042	158	44.93345	5.489718	+	1	15.11126	9.62154
P52298	NCBP2	Nuclear ca	18.001	156	44.29678	5.46913	+	1	15.76264	10.29351
Q96HC4	PDLIM5	PDZ and L	63.945	596	44.03812	5.460681	+	2	15.23212	9.77144
Q96SZ6	CDK5RAP	Mitochondr	67.689	601	43.64017	5.447585	+	1	16.94695	11.49937
Q8TAD7	OCC1	Overexpres	6.407	63	42.78243	5.418947	+	1	15.28352	9.864576
Q9BSD7	NTPCR	Cancer-rel	20.713	190	42.55942	5.411407	+	1	14.54327	9.131867
P43487	RANBP1	Ran-specif	23.31	201	41.76478	5.384215	+	1	15.16746	9.783242
P16104	H2AX	Histone H2	15.145	143	41.69831	5.381917	+	1	15.49457	10.11266
P26196	DDX6	Probable A	54.417	483	41.68649	5.381508	+	2	17.09713	11.71562
Q96FQ6	S100A16	Protein S10	11.801	103	41.60871	5.378814	+	1	14.24882	8.870003
Q9Y580	RBM7	RNA-bindir	30.504	266	41.55015	5.376782	+	1	14.16223	8.785452
Q9H6R4	NOL6	Nucleolar p	127.593	1146	41.32287	5.368869	+	1	13.95301	8.584146
Q14160	SCRIB	Protein scr	174.915	1630	41.13217	5.362195	+	2	16.86786	11.50566
Q96GI7	FAM89A	Protein FAI	19.569	184	41.06598	5.359872	+	1	14.81718	9.457312
O60749	SNX2	Sorting nex	58.471	519	40.3099	5.333062	+	1	14.67733	9.344272
O15427	SLC16A3	Monocarbo	49.469	465	40.1701	5.32805	+	1	14.83047	9.502416
P35659	DEK	Protein DE	42.674	375	39.49734	5.303684	+	1	14.60536	9.30168
P35221	CTNNA1	Catenin alp	100.071	906	39.25169	5.294683	+	2	16.39948	11.1048
P61962	DCAF7	DDB1- and	38.926	342	39.24283	5.294357	+	1	15.14832	9.85396
O00399	DCTN6	Dynactin su	20.747	190	38.89381	5.281469	+	1	16.49193	11.21046
Q9HB71	CACYBP	Calcyclin-b	26.21	228	38.58632	5.270018	+	1	14.62765	9.35763
Q3ZCM7	TUBB8	Tubulin bet	49.776	444	38.45219	5.264994	+	1	16.10278	10.83778
Q99714	HSD17B10	3-hydroxya	26.923	261	38.35894	5.261491	+	1	16.36475	11.10326
Q9UKX7	NUP50	Nuclear po	50.144	468	38.11386	5.252244	+	1	15.27798	10.02573
Q06587	RING1	E3 ubiquitir	42.429	406	37.52143	5.229643	+	1	14.89406	9.664417
P32320	CDA	Cytidine de	16.185	146	36.77465	5.20064	+	1	16.03021	10.82957
Q9HCD5	NCOA5	Nuclear rec	65.536	579	36.77417	5.200621	+	2	17.46714	12.26652
O14513	NCKAP5	Nck-associ	208.537	1909	36.32877	5.183041	+	2	18.69479	13.51175
P13995	MTHFD2	Bifunctiona	37.895	350	36.29931	5.18187	+	1	15.43648	10.25461
Q96SB4	SRPK1	SRSF prote	74.325	655	36.03621	5.171376	+	1	15.35779	10.18642
Q8ND76	CCNY	Cyclin-Y O	39.337	341	35.76676	5.160548	+	1	14.81453	9.653985
Q52LJ0	FAM98B	Protein FAI	45.547	433	35.47311	5.148654	+	1	14.99307	9.844417
Q96QT6	PHF12	PHD finger	109.698	1004	35.28788	5.141101	+	1	16.14326	11.00216
Q99496	RNF2	E3 ubiquitir	37.655	336	35.26267	5.14007	+	1	16.46596	11.32589
Q9UNE7	STUB1	E3 ubiquitir	34.856	303	35.24757	5.139452	+	1	14.97446	9.835008
P04350	TUBB4A	Tubulin bet	49.586	444	35.17222	5.136364	+	1	15.4516	10.31523
P55196	AFDN	Afadin OS=	206.804	1824	35.14501	5.135248	+	1	15.44187	10.30663
Q8NEY1	NAV1	Neuron nav	202.472	1877	34.83153	5.122322	+	2	16.88524	11.76292
Q15185	PTGES3	Prostaglan	18.697	160	34.72653	5.117966	+	1	14.38033	9.262359
Q5TZA2	CROCC	Rootletin O	228.424	2017	34.36574	5.102899	+	1	14.88527	9.782368
Q9NSB8	HOMER2	Homer prot	40.627	354	34.35349	5.102385	+	1	15.36379	10.26141
Q16718	NDUFA5	NADH deh	13.459	116	34.30189	5.100216	+	1	15.40251	10.3023
Q9Y333	LSM2	U6 snRNA-	10.835	95	34.07567	5.09067	+	1	14.32453	9.233862
P61326	MAGOH	Protein ma	17.164	146	33.82966	5.080217	+	1	14.54061	9.460396
O94973	AP2A2	AP-2 comp	103.96	939	33.74242	5.076491	+	2	17.36743	12.29094
Q9UQB8	BAIAP2	Brain-spec	60.868	552	33.6854	5.074052	+	2	16.36965	11.2956
Q14974	KPNB1	Importin su	97.17	876	33.52053	5.066973	+	2	17.592	12.52503
Q9NUL3	STAU2	Double-stra	62.608	570	33.46591	5.06462	+	1	15.03699	9.972369
Q7Z5L9	IRF2BP2	Interferon r	61.025	587	33.24129	5.054905	+	1	14.49467	9.439764
Q0JRZ9	FCHO2	F-BAR don	88.924	810	33.13743	5.05039	+	1	14.76736	9.716967
O75832	PSMD10	26S protea	24.428	226	32.88002	5.039139	+	1	14.89997	9.860831
Q96FX8	PERP	p53 apopto	21.386	193	32.86517	5.038487	+	1	15.64732	10.60883
P15336	ATF2	Cyclic AMF	54.537	505	32.2798	5.01256	+	1	14.15133	9.138774
Q06323	PSME1	Proteasom	28.723	249	32.27563	5.012373	+	1	14.45565	9.443275
Q6YHK3	CD109	CD109 anti	161.689	1445	31.98808	4.999463	+	2	16.98715	11.98769
Q12849	GRSF1	G-rich sequ	53.126	480	31.98342	4.999252	+	1	15.87289	10.87364
Q96SB3	PPP1R9B	Neurabin-2	89.334	817	31.96207	4.998289	+	1	15.25706	10.25877
Q96IK1	BOD1	Biorientatio	19.196	185	31.90833	4.995861	+	1	15.75377	10.7579

O15372	EIF3H	Eukaryotic	39.93	352	31.86551	4.993924	+	1	14.46741	9.473491
Q7KZF4	SND1	Staphyloco	101.997	910	31.07755	4.957801	+	1	13.48596	8.528154
O60658	PDE8A	High affinit	93.304	829	31.06062	4.957015	+	1	16.14277	11.18575
O43447	PPIH	Peptidyl-pr	19.208	177	30.92355	4.950634	+	1	15.33919	10.38855
Q9NXR1	NDE1	Nuclear dis	37.721	335	30.8327	4.946389	+	2	16.50342	11.55703
Q6F5E8	CARMIL2	Capping pr	154.689	1435	30.69913	4.940126	+	1	14.29519	9.355069
Q6AI12	ANKRD40	Ankyrin rep	41.088	368	30.60163	4.935536	+	1	13.95329	9.017751
P13797	PLS3	Plastin-3 O	70.811	630	30.57864	4.934452	+	2	16.9117	11.97724
Q7L2H7	EIF3M	Eukaryotic	42.503	374	30.54994	4.933098	+	1	14.33497	9.401874
Q8IWE4	DCUN1D3	DCN1-like	34.291	304	30.45439	4.928578	+	1	14.52025	9.591672
Q12929	EPS8	Epidermal	91.882	822	30.16018	4.914573	+	1	15.74162	10.82705
Q15293	RCN1	Reticulocal	38.89	331	30.02598	4.908139	+	1	14.74341	9.835275
Q13200	PSMD2	26S protea	100.2	908	30.00621	4.907189	+	2	16.23783	11.33064
Q9H910	JPT2	Jupiter mic	20.063	190	29.9927	4.90654	+	1	14.61563	9.70909
Q969V6	MRTFA	Myocardin-	98.919	931	29.71558	4.893147	+	1	13.28711	8.393959
Q53GL0	PLEKHO1	Pleckstrin h	46.237	409	29.4171	4.878583	+	1	13.75697	8.87839
P62070	RRAS2	Ras-related	23.4	204	29.39996	4.877742	+	1	16.08549	11.20775
O00410	IPO5	Importin-5	123.63	1097	29.38899	4.877204	+	1	13.59456	8.717354
Q8TD31	CCHCR1	Coiled-coil	88.671	782	29.35154	4.875365	+	1	15.38613	10.51077
Q9UBS4	DNAJB11	DnaJ homo	40.514	358	29.2243	4.869097	+	1	14.49523	9.626134
P29992	GNA11	Guanine nu	42.123	359	29.18816	4.867311	+	1	15.85099	10.98368
P63162	SNRPN	Small nucle	24.614	240	29.14176	4.865016	+	1	13.95828	9.093265
Q8IZD4	DCP1B	mRNA-dec	67.723	617	28.66202	4.841068	+	1	16.40001	11.55894
Q8IU81	IRF2BP1	Interferon r	61.688	584	28.5067	4.833229	+	1	13.66445	8.831218
Q92615	LARP4B	La-related	80.552	738	28.43412	4.829551	+	1	14.30464	9.475085
Q10567	AP1B1	AP-1 comp	104.607	949	28.30684	4.823079	+	1	14.74189	9.91881
P26992	CNTFR	Ciliary neu	40.633	372	28.26586	4.820989	+	1	15.52154	10.70055
Q5TC63	GRTP1	Growth hor	38.554	336	28.23114	4.819216	+	2	15.86252	11.0433
Q9UBP9	GULP1	PTB domai	34.49	304	28.04279	4.809558	+	1	14.74798	9.938425
Q8WU90	ZC3H15	Zinc finger	48.602	426	27.97406	4.806018	+	1	14.13867	9.332654
Q9BV40	VAMP8	Vesicle-ass	11.438	100	27.8337	4.798761	+	1	15.13611	10.33735
P62633	CNBP	CCHC-type	19.463	177	27.72356	4.79304	+	1	13.32221	8.529169
O60256	PRPSAP2	Phosphorib	40.926	369	27.68396	4.790978	+	1	14.51453	9.723551
Q9NXV2	KCTD5	BTB/POZ d	26.093	234	27.45269	4.778875	+	1	15.28605	10.50718
Q9UIV1	CNOT7	CCR4-NOT	32.745	285	27.05053	4.757585	+	1	15.54951	10.79193
Q15388	TOMM20	Mitochondr	16.298	145	26.99338	4.754534	+	1	14.99276	10.23823
P46013	MKI67	Proliferatio	358.694	3256	26.81389	4.744909	+	1	13.84931	9.104399
P43686	PSMC4	26S protea	47.366	418	26.7994	4.744129	+	1	15.68253	10.9384
Q15311	RALBP1	RalA-bindir	76.063	655	26.75964	4.741987	+	1	14.85077	10.10879
Q9H0S4	DDX47	Probable A	50.647	455	26.74577	4.741239	+	1	14.42404	9.682796
Q9UDY2	TJP2	Tight juncti	133.958	1190	26.68941	4.738196	+	2	17.38642	12.64822
O00505	KPNA3	Importin su	57.811	521	26.5684	4.731639	+	1	16.62414	11.8925
Q9UHD8	SEPTIN9	Septin-9 O	65.401	586	26.54329	4.730275	+	1	14.32404	9.593765
P55072	VCP	Transitiona	89.322	806	26.48957	4.727353	+	2	16.22006	11.4927
P62875	POLR2L	DNA-direct	7.645	67	26.40165	4.722556	+	1	14.39252	9.669963
O00154	ACOT7	Cytosolic a	41.796	380	26.39898	4.72241	+	1	13.77633	9.05392
O75489	NDUFS3	NADH deh	30.242	264	26.22038	4.712617	+	1	15.35101	10.63839
Q6UXN9	WDR82	WD repeat	35.079	313	26.11441	4.706774	+	1	16.10427	11.3975
O60437	PPL	Periplakin	204.747	1756	26.03089	4.702153	+	1	15.047	10.34484
Q13616	CUL1	Cullin-1 OS	89.679	776	25.83608	4.691315	+	1	14.03738	9.34606
Q8TEH3	DENND1A	DENN dom	110.577	1009	25.81347	4.690052	+	1	14.72056	10.03051
Q96C24	SYTL4	Synaptotag	76.024	671	25.66352	4.681647	+	1	14.92495	10.2433
O95817	BAG3	BAG family	61.595	575	25.5095	4.672963	+	1	15.40005	10.72708
P49750	YLPM1	YLP motif-	241.645	2146	25.3044	4.661316	+	1	14.65626	9.994941
Q6IQ23	PLEKHA7	Pleckstrin h	127.135	1121	25.16625	4.653418	+	1	15.22931	10.57589
P78362	SRPK2	SRSF prote	77.527	688	25.12631	4.651127	+	1	14.64853	9.997399
Q96DV4	MRPL38	39S riboso	44.597	380	25.04377	4.64638	+	1	13.67298	9.026598
P46087	NOP2	Probable 2	89.302	812	25.01509	4.644727	+	2	14.78274	10.13802
Q15417	CNN3	Calponin-3	36.414	329	25.00611	4.644209	+	1	14.76674	10.12253

Q12962	TAF10	Transcripti	21.711	218	24.91047	4.638681	+	1	14.47129	9.832614
Q9UBQ5	EIF3K	Eukaryotic	25.06	218	24.88732	4.637339	+	1	15.10115	10.46382
O60262	GNG7	Guanine nu	7.522	68	24.74502	4.629066	+	1	14.61707	9.988
P67870	CSNK2B	Casein kina	24.942	215	24.63571	4.622679	+	1	15.34547	10.7228
O15020	SPTBN2	Spectrin be	271.325	2390	24.62766	4.622208	+	1	14.62497	10.00276
Q9Y6W5	WASF2	Wiskott-Alc	54.284	498	24.5849	4.6197	+	1	14.28836	9.668661
P49795	RGS19	Regulator c	24.636	217	24.33625	4.605035	+	1	14.85219	10.24715
Q96EY7	PTCD3	Pentatricop	78.55	689	24.32714	4.604495	+	1	14.11285	9.508352
Q9Y446	PKP3	Plakophilin	87.082	797	24.30803	4.603361	+	1	15.63988	11.03652
P50151	GNG10	Guanine nu	7.205	68	24.30452	4.603153	+	1	14.94045	10.3373
P54136	RARS1	Arginine--tF	75.379	660	24.12979	4.592744	+	2	17.31061	12.71787
Q9UMS4	PRPF19	Pre-mRNA	55.181	504	23.61608	4.561698	+	1	15.60305	11.04135
Q7Z2W9	MRPL21	39S riboso	22.815	205	23.52209	4.555944	+	1	13.17365	8.617702
O15446	POLR1G	DNA-direct	54.986	510	23.48175	4.553468	+	1	14.512	9.958532
Q9NR12	PDLIM7	PDZ and L	49.845	457	23.39441	4.548092	+	2	16.8589	12.31081
Q9UBU8	MORF4L1	Mortality fa	41.474	362	23.39342	4.548031	+	2	16.14655	11.59852
Q6P161	MRPL54	39S riboso	15.819	138	23.32403	4.543745	+	1	14.22724	9.683495
Q14156	EFR3A	Protein EFI	92.924	821	23.23914	4.538485	+	1	14.76446	10.22597
O14976	GAK	Cyclin-G-as	143.191	1311	23.23444	4.538193	+	1	14.49198	9.953786
P82650	MRPS22	28S riboso	41.28	360	22.99535	4.52327	+	1	15.13443	10.61116
Q9Y2R5	MRPS17	28S riboso	14.502	130	22.80884	4.511521	+	1	14.81122	10.2997
P50552	VASP	Vasodilator	39.83	380	22.68072	4.503394	+	2	15.41141	10.90802
Q9H4A3	WNK1	Serine/thre	250.794	2382	22.53978	4.494401	+	1	12.82851	8.334112
Q9ULJ8	PPP1R9A	Neurabin-1	123.342	1098	22.53472	4.494078	+	2	15.27281	10.77873
Q96DX4	RSPRY1	RING finge	64.18	576	22.52794	4.493643	+	1	14.56605	10.07241
Q8NEJ9	NGDN	Neuroguidi	35.894	315	22.46662	4.489711	+	1	13.78862	9.298905
P82930	MRPS34	28S riboso	25.65	218	22.41106	4.486139	+	2	15.14203	10.65589
Q9NZN8	CNOT2	CCR4-NOT	59.738	540	22.345	4.48188	+	1	14.63288	10.151
O15231	ZNF185	Zinc finger	73.525	689	22.32262	4.480434	+	1	15.5137	11.03326
O43251	RBFOX2	RNA bindir	41.374	390	22.29575	4.478697	+	1	14.06095	9.582253
Q14694	USP10	Ubiquitin ca	87.134	798	22.18985	4.471828	+	1	14.59991	10.12808
Q9UJU6	DBNL	Drebrin-like	48.207	430	22.18826	4.471725	+	1	14.61902	10.14729
Q9BTE1	DCTN5	Dynactin su	20.127	182	22.18635	4.471601	+	1	14.11097	9.639372
Q9UPS6	SETD1B	Histone-lys	212.803	1966	22.18252	4.471352	+	1	14.46646	9.995107
Q9Y2S7	POLDIP2	Polymerase	42.033	368	22.15681	4.469678	+	1	14.59863	10.12896
O15484	CAPN5	Calpain-5 (	73.169	640	22.00248	4.459594	+	1	13.78913	9.329532
Q04637	EIF4G1	Eukaryotic	175.491	1599	21.94027	4.455509	+	2	15.14446	10.68895
P53355	DAPK1	Death-asso	160.046	1430	21.91253	4.453684	+	1	14.57436	10.12067
P34932	HSPA4	Heat shock	94.331	840	21.89462	4.452504	+	1	15.38899	10.93649
Q9H9J2	MRPL44	39S riboso	37.535	332	21.74602	4.44268	+	1	14.07965	9.636972
P52797	EFNA3	Ephrin-A3 (	26.35	238	21.63211	4.435103	+	1	15.32411	10.88901
P61289	PSME3	Proteasom	29.506	254	21.55825	4.430168	+	1	14.13218	9.70201
O60701	UGDH	UDP-gluco	55.024	494	21.48125	4.425006	+	2	15.42282	10.99781
Q9NZ32	ACTR10	Actin-relate	46.307	417	21.39161	4.418973	+	2	16.88107	12.46209
P50542	PEX5	Peroxisoma	70.865	639	21.36896	4.417445	+	1	15.16357	10.74613
P13073	COX4I1	Cytochrom	19.577	169	21.29532	4.412465	+	1	13.40394	8.991479
Q8WVV9	HNRNPLL	Heterogene	60.083	542	21.28957	4.412075	+	1	14.85316	10.44109
Q9UKF6	CPSF3	Cleavage a	77.486	684	21.15191	4.402716	+	1	14.21082	9.808107
Q8IV63	VRK3	Inactive se	52.881	474	21.13312	4.401434	+	1	14.69534	10.2939
Q92769	HDAC2	Histone de	55.364	488	21.09842	4.399063	+	1	14.71118	10.31212
P82673	MRPS35	28S riboso	36.844	323	21.07836	4.397691	+	1	14.37001	9.972315
Q15631	TSN	Translin OS	26.183	228	20.98851	4.391528	+	1	13.5411	9.149569
O95433	AHSA1	Activator of	38.274	338	20.93814	4.388061	+	1	15.10349	10.71543
Q8ND56	LSM14A	Protein LSI	50.53	463	20.85569	4.382369	+	1	13.75217	9.369802
Q5T6F2	UBAP2	Ubiquitin-as	117.116	1119	20.78918	4.377761	+	1	14.20945	9.831693
Q9Y3A4	RRP7A	Ribosomal	32.334	280	20.70932	4.372208	+	1	13.21837	8.846158
O60341	KDM1A	Lysine-spe	92.903	852	20.59123	4.363958	+	1	15.26942	10.90546
O00165	HAX1	HCLS1-ass	31.621	279	20.56251	4.361944	+	1	14.43665	10.0747
Q9NPA8	ENY2	Transcripti	11.529	101	20.52473	4.359291	+	1	14.45398	10.09469

P60059	SEC61G	Protein trar	7.741	68	20.35214	4.347109	+	1	14.48878	10.14167
Q9UFW8	CGGBP1	CGG triplet	18.82	167	20.30596	4.343831	+	1	14.52246	10.17863
Q05048	CSTF1	Cleavage s	48.358	431	20.30204	4.343553	+	1	14.56659	10.22304
Q96D71	REPS1	RalBP1-as	86.662	796	20.25467	4.340183	+	2	17.24275	12.90256
Q9Y5Q8	GTF3C5	General tra	59.571	519	20.22847	4.338315	+	1	13.01048	8.672161
Q9Y3D6	FIS1	Mitochondr	16.938	152	20.17416	4.334436	+	1	13.66089	9.326451
Q9P1Z2	CALCOCO	Calcium-bi	77.336	691	20.11608	4.330277	+	1	14.975	10.64472
Q16181	SEPTIN7	Septin-7 O	50.68	437	20.11379	4.330113	+	1	14.89576	10.56565
Q6ZN55	ZNF574	Zinc finger	98.9	896	20.10326	4.329358	+	2	15.74452	11.41516
Q92945	KHSRP	Far upstrea	73.115	711	20.06146	4.326354	+	1	15.4664	11.14004
Q96BR5	COA7	Cytochrom	25.709	231	20.05853	4.326144	+	1	13.24496	8.918815
Q9HC52	CBX8	Chromobox	43.396	389	20.05481	4.325876	+	1	15.58722	11.26134
P58546	MTPN	Myotrophin	12.895	118	20.02351	4.323623	+	1	15.591	11.26737
Q9H6S3	EPS8L2	Epidermal	80.621	715	20.00942	4.322607	+	1	15.31819	10.99558
Q9HCC0	MCCC2	Methylcroto	61.333	563	20.0033	4.322166	+	2	15.37337	11.05121
O60237	PPP1R12B	Protein pho	110.404	982	20.00328	4.322165	+	2	15.89529	11.57313
Q9P2B4	CTTNBP2N	CTTNBP2	70.158	639	19.89296	4.314186	+	1	14.85316	10.53898
Q99460	PSMD1	26S protea	105.836	953	19.73849	4.30294	+	1	15.25218	10.94925
Q99733	NAP1L4	Nucleosom	42.823	375	19.57865	4.29121	+	1	13.57163	9.280425
Q86YV9	HPS6	Hermansky	82.975	775	19.45283	4.281908	+	2	17.1894	12.90749
Q8WX93	PALLD	Palladin OS	150.564	1383	19.43425	4.28053	+	2	16.96183	12.6813
O96008	TOMM40	Mitochondr	37.893	361	19.42714	4.280001	+	1	13.87094	9.590941
P29966	MARCKS	Myristoylat	31.555	332	19.30234	4.270704	+	1	14.11764	9.846939
Q86VM9	ZC3H18	Zinc finger	106.378	953	19.28854	4.269672	+	1	13.69425	9.424577
P63172	DYNLT1	Dynein ligh	12.452	113	19.19638	4.262762	+	1	14.58267	10.31991
Q9P2N5	RBM27	RNA-bindir	118.718	1060	19.13211	4.257924	+	1	13.95283	9.694908
Q6ZSR9		Uncharacte	37.976	355	19.11516	4.256646	+	1	14.69843	10.44179
Q8IY63	AMOTL1	Angiomotin	106.574	956	19.08946	4.254704	+	2	15.07903	10.82432
P62993	GRB2	Growth fac	25.206	217	19.06903	4.25316	+	1	14.89955	10.64639
A0AV96	RBM47	RNA-bindir	64.099	593	19.06129	4.252574	+	1	14.06752	9.814945
Q7LGA3	HS2ST1	Heparan su	41.881	356	19.06002	4.252477	+	1	13.52368	9.271207
Q9BRJ6	C7orf50	Uncharacte	22.083	194	18.92923	4.242544	+	2	16.02845	11.7859
Q14134	TRIM29	Tripartite m	65.835	588	18.91114	4.241165	+	1	15.03708	10.79591
Q9Y294	ASF1A	Histone cha	22.969	204	18.89879	4.240222	+	1	14.72526	10.48504
Q9C0H5	ARHGAP3	Rho GTPas	121.286	1083	18.89317	4.239793	+	1	13.72409	9.484295
Q7LBR1	CHMP1B	Charged m	22.109	199	18.88447	4.239128	+	1	13.96217	9.723045
Q8IV08	PLD3	5'-3' exonu	54.705	490	18.61203	4.218163	+	1	12.34829	8.130122
P30153	PPP2R1A	Serine/thre	65.309	589	18.54872	4.213248	+	2	16.25052	12.03727
Q99816	TSG101	Tumor sus	43.944	390	18.53863	4.212463	+	1	14.54647	10.33401
Q9HBH0	RHOF	Rho-related	23.625	211	18.52821	4.211652	+	1	14.6277	10.41605
Q9BVK6	TMED9	Transmem	27.277	235	18.50431	4.20979	+	1	13.09253	8.882736
Q9UGT4	SUSD2	Sushi dom	90.208	822	18.39161	4.200976	+	1	14.41607	10.2151
Q14344	GNA13	Guanine nu	44.05	377	18.3902	4.200865	+	1	14.54019	10.33932
Q9Y508	RNF114	E3 ubiquitir	25.694	228	18.34325	4.197178	+	1	13.67276	9.475579
Q9Y448	KNSTRN	Small kinet	35.438	316	18.32645	4.195856	+	1	13.77592	9.580063
O75569	PRKRA	Interferon-i	34.404	313	18.28073	4.192251	+	1	13.61402	9.421768
P05023	ATP1A1	Sodium/po	112.896	1023	18.22764	4.188056	+	1	15.01297	10.82492
O15050	TRANK1	TPR and a	336.221	2925	18.21247	4.186855	+	1	14.70531	10.51845
Q86Y79	PTRH1	Probable p	22.937	214	18.17288	4.183715	+	1	13.84529	9.661579
Q5T8I3	FAM102B	Protein FAI	39.308	360	18.17095	4.183562	+	1	13.36892	9.185354
Q9HBM6	TAF9B	Transcripti	27.622	251	18.16456	4.183055	+	1	14.2799	10.09685
A0MZ66	SHTN1	Shootin-1 (	71.64	631	18.14962	4.181867	+	2	17.35879	13.17692
Q9BWF3	RBM4	RNA-bindir	40.314	364	18.03815	4.172979	+	1	14.74226	10.56928
O00231	PSMD11	26S protea	47.464	422	17.97642	4.168034	+	1	14.71032	10.54229
Q15758	SLC1A5	Neutral am	56.598	541	17.93246	4.164502	+	1	14.35913	10.19463
Q9NY35	CLDND1	Claudin do	28.603	253	17.91612	4.163186	+	1	14.32341	10.16022
Q8IYB5	SMAP1	Stromal me	50.386	467	17.91166	4.162827	+	1	14.51742	10.3546
Q9NTK5	OLA1	Obg-like AT	44.744	396	17.90732	4.162478	+	1	14.72962	10.56714
Q99618	CDCA3	Cell divisio	28.998	268	17.87817	4.160127	+	1	14.7719	10.61178

O75381	PEX14	Peroxisom	41.237	377	17.84187	4.157195	+	2	16.14083	11.98363
Q9H4M9	EHD1	EH domain	60.627	534	17.72597	4.147793	+	2	16.39652	12.24873
P55884	EIF3B	Eukaryotic	92.482	814	17.70581	4.146151	+	2	15.76774	11.62159
Q13404	UBE2V1	Ubiquitin-c	16.495	147	17.69256	4.145071	+	1	12.96613	8.821056
Q9UPX8	SHANK2	SH3 and m	158.822	1470	17.64659	4.141318	+	2	16.28486	12.14354
P52292	KPNA2	Importin su	57.862	529	17.57629	4.135559	+	2	19.45173	15.31618
Q9BYJ9	YTHDF1	YTH doma	60.874	559	17.4659	4.126469	+	1	13.69849	9.572018
Q92922	SMARCC1	SWI/SNF c	122.867	1105	17.38792	4.120013	+	1	13.69555	9.575541
O43504	LAMTOR5	Ragulator c	9.614	91	17.35968	4.117668	+	1	15.0858	10.96814
P30876	POLR2B	DNA-direct	133.897	1174	17.33077	4.115264	+	1	13.7628	9.647533
P11274	BCR	Breakpoint	142.819	1271	17.31602	4.114035	+	1	12.62726	8.513225
Q9Y5M8	SRPRB	Signal recc	29.702	271	17.27072	4.110256	+	1	14.84107	10.73082
O95274	LYPD3	Ly6/PLAUF	35.971	346	17.26922	4.110131	+	1	15.06643	10.95629
Q8WUM4	PDCD6IP	Programm	96.023	868	17.22219	4.106197	+	2	14.8267	10.7205
Q13426	XRCC4	DNA repair	38.287	336	17.20521	4.104774	+	1	12.30905	8.204276
P51608	MECP2	Methyl-CpC	52.441	486	17.20348	4.104628	+	1	14.25827	10.15364
P51153	RAB13	Ras-relatec	22.774	203	17.01518	4.08875	+	1	13.3479	9.259148
Q2M2I8	AAK1	AP2-assoc	103.885	961	17.01176	4.088461	+	1	13.64993	9.561469
Q9GZS3	WDR61	WD repeat	33.581	305	16.98687	4.086348	+	1	14.35156	10.26521
Q9UNF0	PACSIN2	Protein kin	55.739	486	16.92281	4.080898	+	1	15.42993	11.34903
Q9H0D6	XRN2	5'-3' exorib	108.582	950	16.90364	4.079262	+	1	14.79822	10.71896
P54652	HSPA2	Heat shock	70.021	639	16.79042	4.069566	+	1	13.9571	9.887536
Q969F1	GTF3C6	General tra	24.049	213	16.73572	4.064858	+	1	13.38613	9.321274
Q9P270	SLAIN2	SLAIN mot	62.543	581	16.63393	4.056057	+	1	13.662	9.605944
Q9NPA2	MMP25	Matrix meta	62.554	562	16.60503	4.053549	+	1	14.84882	10.79527
Q6P4F7	ARHGAP1	Rho GTPa	113.866	1023	16.5166	4.045845	+	1	14.61827	10.57243
O43823	AKAP8	A-kinase a	76.108	692	16.46965	4.041738	+	1	13.68814	9.646403
O95373	IPO7	Importin-7	119.517	1038	16.44486	4.039565	+	2	15.44103	11.40147
Q9BTK6	PAGR1	PAXIP1-as	27.716	254	16.44482	4.039561	+	1	15.16816	11.1286
Q8N556	AFAP1	Actin filam	80.725	730	16.44116	4.039241	+	1	16.42108	12.38184
P40222	TXLNA	Alpha-taxili	61.891	546	16.3344	4.029842	+	1	14.00825	9.978412
Q9Y6G9	DYNC1LI1	Cytoplasmi	56.579	523	16.32363	4.02889	+	1	13.9619	9.933012
O60306	AQR	RNA helica	171.295	1485	16.3209	4.028649	+	1	13.70704	9.678387
Q9BZK7	TBL1XR1	F-box-like/	55.595	514	16.24368	4.021807	+	1	13.83851	9.816708
Q9UBC2	EPS15L1	Epidermal	94.255	864	16.18149	4.016272	+	2	16.5145	12.49823
Q14116	IL18	Interleukin-	22.326	193	16.161	4.014444	+	1	14.30235	10.28791
Q9Y5A9	YTHDF2	YTH doma	62.334	579	16.15302	4.013732	+	1	14.88984	10.8761
Q01664	TFAP4	Transcripti	38.726	338	16.11281	4.010136	+	1	15.01437	11.00423
Q9HAN9	NMNAT1	Nicotinami	31.932	279	16.06921	4.006227	+	1	14.19083	10.1846
Q8TF65	GIPC2	PDZ doma	34.354	315	16.05688	4.00512	+	1	13.26498	9.259865
O14713	ITGB1BP1	Integrin be	21.782	200	16.05314	4.004783	+	1	13.95465	9.949867
Q9NZR1	TMOD2	Tropomodu	39.595	351	15.9879	3.998909	+	2	16.01787	12.01897
P54819	AK2	Adenylate	26.478	239	15.98366	3.998526	+	1	13.48306	9.484534
O75083	WDR1	WD repeat	66.194	606	15.93066	3.993734	+	2	15.1329	11.13917
P61011	SRP54	Signal recc	55.705	504	15.90979	3.991842	+	1	13.94123	9.949389
Q14558	PRPSAP1	Phosphorib	39.394	356	15.89323	3.99034	+	1	13.85701	9.866669
Q9P2E9	RRBP1	Ribosome-	152.456	1410	15.87439	3.98863	+	2	13.763	9.774375
O60232	ZNRD2	Protein ZN	21.474	199	15.80505	3.982314	+	1	15.36663	11.38432
P61604	HSPE1	10 kDa hea	10.932	102	15.80464	3.982276	+	1	14.34478	10.36251
Q15599	SLC9A3R2	Na(+)/H(+)	37.414	337	15.78355	3.98035	+	1	14.79264	10.81229
Q9Y399	MRPS2	28S ribosо	33.249	296	15.77402	3.979479	+	1	13.57033	9.590851
Q15836	VAMP3	Vesicle-ass	11.309	100	15.749	3.977189	+	1	14.38445	10.40726
Q92538	GBF1	Golgi-spec	206.446	1859	15.53778	3.957709	+	1	15.05947	11.10176
B1AK53	ESPN	Espin OS=	91.733	854	15.48826	3.953103	+	1	14.62845	10.67534
P49419	ALDH7A1	Alpha-amir	58.487	539	15.43245	3.947895	+	1	13.73217	9.784272
Q9H5V8	CDCP1	CUB doma	92.932	836	15.43207	3.947859	+	1	13.45764	9.509778
Q12996	CSTF3	Cleavage s	82.922	717	15.4272	3.947404	+	1	14.21265	10.26524
Q15046	KARS1	Lysine--tRI	68.048	597	15.42668	3.947356	+	2	15.49748	11.55012
O95425	SVIL	Supervillin	247.746	2214	15.42454	3.947155	+	2	17.80776	13.8606

P33176	KIF5B	Kinesin-1 h	109.685	963	15.37595	3.942604	+	2	14.72249	10.77988
P54709	ATP1B3	Sodium/po	31.513	279	15.3592	3.941031	+	1	14.39238	10.45135
Q15024	EXOSC7	Exosome c	31.821	291	15.35148	3.940306	+	1	14.77195	10.83165
Q9H4P4	RNF41	E3 ubiquitin	35.905	317	15.34768	3.939949	+	1	14.19921	10.25926
P24752	ACAT1	Acetyl-CoA	45.2	427	15.3354	3.938794	+	1	14.04951	10.11071
P51532	SMARCA4	Transcriptio	184.646	1647	15.2993	3.935393	+	1	13.71992	9.78453
Q8IVF2	AHNAK2	Protein AH	616.629	5795	15.29572	3.935056	+	1	13.91017	9.975118
Q8TDN6	BRIX1	Ribosome	41.401	353	15.2914	3.934649	+	1	14.4399	10.50525
Q96B70	LENG9	Leukocyte	53.167	501	15.26561	3.932213	+	1	15.5279	11.59569
Q96EL3	MRPL53	39S riboso	12.107	112	15.25443	3.931157	+	1	13.69043	9.759278
Q9H4F8	SMOC1	SPARC-rel	48.163	434	15.24494	3.930259	+	1	13.53211	9.601854
Q53EZ4	CEP55	Centrosom	54.178	464	15.23855	3.929654	+	1	13.56021	9.63056
Q8NFF5	FLAD1	FAD syntha	65.266	587	15.1333	3.919655	+	1	12.89349	8.973837
Q9NQW6	ANLN	Anillin OS=	124.199	1124	15.13117	3.919452	+	1	15.70968	11.79022
O00629	KPNA4	Importin su	57.887	521	15.13051	3.919389	+	1	14.624	10.70461
Q9UN37	VPS4A	Vacuolar p	48.898	437	15.09721	3.91621	+	1	15.26543	11.34922
O94875	SORBS2	Sorbin and	124.108	1100	15.07541	3.914125	+	2	19.59822	15.68409
Q96SI9	STRBP	Spermatid	73.653	672	15.03311	3.910071	+	1	14.48665	10.57658
O75533	SF3B1	Splicing fac	145.83	1304	15.02788	3.909569	+	2	15.24418	11.33461
P00390	GSR	Glutathione	56.257	522	14.94347	3.901443	+	1	14.37273	10.47129
Q9NZM1	MYOF	Myoferlin C	234.709	2061	14.90935	3.898146	+	1	15.73013	11.83198
Q8WUU5	GATAD1	GATA zinc	28.69	269	14.90566	3.897789	+	1	15.83837	11.94058
Q9NZI7	UBP1	Upstream-l	60.491	540	14.85893	3.893258	+	1	15.53044	11.63718
Q9UPQ9	TNRC6B	Trinucleotic	194.002	1833	14.82743	3.890197	+	1	14.22724	10.33704
A6NKD9	CCDC85C	Coiled-coil	45.21	419	14.79075	3.886623	+	1	13.41244	9.525814
O00512	BCL9	B-cell CLL/	149.29	1426	14.78185	3.885755	+	1	13.19708	9.311323
P62330	ARF6	ADP-ribosy	20.082	175	14.77885	3.885463	+	2	15.40225	11.51678
Q9NXV6	CDKN2AIP	CDKN2A-in	61.125	580	14.77799	3.885378	+	1	13.32305	9.437677
Q15382	RHEB	GTP-bindin	20.497	184	14.76236	3.883851	+	1	14.84186	10.95801
Q9P0L0	VAPA	Vesicle-ass	27.893	249	14.75822	3.883447	+	2	17.65235	13.76891
O00560	SDCBP	Syntenin-1	32.444	298	14.74084	3.881746	+	1	13.73767	9.855923
P63151	PPP2R2A	Serine/thre	51.692	447	14.71113	3.878836	+	1	15.40138	11.52254
Q6ZW49	PAXIP1	PAX-intera	121.341	1069	14.70537	3.878271	+	1	14.77582	10.89754
Q9Y3B9	RRP15	RRP15-like	31.484	282	14.68642	3.876411	+	1	15.18084	11.30443
Q9UKD1	GMEB2	Glucocortic	56.421	530	14.68409	3.876182	+	1	14.81458	10.9384
O95197	RTN3	Reticulon-3	112.611	1032	14.63738	3.871586	+	1	13.661	9.789413
P19784	CSNK2A2	Casein kina	41.213	350	14.63655	3.871504	+	1	13.79411	9.922607
O00233	PSMD9	26S protea	24.682	223	14.59871	3.867769	+	1	14.66861	10.80084
Q5T3I0	GPATCH4	G patch do	50.381	446	14.57932	3.865851	+	1	14.77535	10.9095
Q92572	AP3S1	AP-3 comp	21.732	193	14.5387	3.861826	+	1	14.0135	10.15167
Q9BTE3	MCMBP	Mini-chrom	72.98	642	14.5135	3.859323	+	1	13.26624	9.406922
O60716	CTNND1	Catenin de	108.17	968	14.48556	3.856544	+	1	14.25886	10.40232
Q0VDF9	HSPA14	Heat shock	54.794	509	14.43198	3.851197	+	1	13.81508	9.963886
Q96T37	RBM15	RNA-bindin	107.189	977	14.41804	3.849803	+	1	13.04707	9.197269
Q9Y266	NUDC	Nuclear mi	38.243	331	14.31611	3.839568	+	1	14.18975	10.35018
Q9UK22	FBXO2	F-box only	33.328	296	14.27392	3.83531	+	1	13.53976	9.704455
O75940	SMNDC1	Survival of	26.711	238	14.24621	3.832507	+	1	14.9359	11.1034
Q16540	MRPL23	39S riboso	17.781	153	14.2185	3.829698	+	1	14.09655	10.26685
Q16543	CDC37	Hsp90 co-c	44.468	378	14.21008	3.828842	+	2	16.76513	12.93629
Q99613	EIF3C	Eukaryotic	105.344	913	14.14535	3.822256	+	1	15.04093	11.21868
P62191	PSMC1	26S protea	49.185	440	14.12905	3.820593	+	2	15.3966	11.57601
Q15286	RAB35	Ras-related	23.025	201	14.03827	3.811294	+	1	14.54551	10.73421
O95757	HSPA4L	Heat shock	94.512	839	14.00755	3.808133	+	1	14.036	10.22787
Q9P206	KIAA1522	Uncharacte	107.095	1035	13.9987	3.807221	+	1	14.50426	10.69704
Q16658	FSCN1	Fascin OS=	54.53	493	13.92859	3.799977	+	2	19.26497	15.46499
P78527	PRKDC	DNA-deper	469.089	4128	13.90021	3.797034	+	2	13.4543	9.657265
Q86UE4	MTDH	Protein LYI	63.837	582	13.88984	3.795958	+	1	15.37833	11.58237
Q9BXS5	AP1M1	AP-1 comp	48.587	423	13.8766	3.794582	+	1	13.55267	9.758087
P10606	COX5B	Cytochrom	13.696	129	13.86558	3.793436	+	1	13.62913	9.835692

Q9NYK5	MRPL39	39S ribosо	38.712	338	13.79655	3.786236	+	1	13.34748	9.561247
Q9NRR3	CDC42SE2	CDC42 sm	9.223	84	13.73397	3.779677	+	1	13.60641	9.826729
Q9C0B5	ZDHHC5	Palmitoyltr	77.545	715	13.72319	3.778544	+	1	14.81513	11.03659
Q5VT25	CDC42BPA	Serine/thre	197.307	1732	13.66353	3.772259	+	2	14.0655	10.29324
Q9Y512	SAMM50	Sorting and	51.976	469	13.65453	3.771308	+	2	15.40035	11.62904
Q9NQ84	GPRC5C	G-protein c	48.193	441	13.62092	3.767752	+	1	14.27976	10.512
O00443	PIK3C2A	Phosphatid	190.68	1686	13.60743	3.766323	+	2	15.01437	11.24805
Q14152	EIF3A	Eukaryotic	166.569	1382	13.58447	3.763886	+	2	14.82883	11.06494
Q8WXE1	ATRIP	ATR-intera	85.838	791	13.54431	3.759615	+	1	13.6086	9.848986
O60684	KPNA6	Importin su	60.03	536	13.48764	3.753566	+	1	14.37579	10.62222
Q9Y6I3	EPN1	Epsin-1 OS	60.293	576	13.45848	3.750444	+	2	16.32615	12.5757
P26640	VARS1	Valine--tRN	140.476	1264	13.45111	3.749653	+	1	14.09581	10.34616
Q13011	ECH1	Delta(3,5)-I	35.816	328	13.39945	3.744102	+	1	12.4213	8.6772
P21912	SDHB	Succinate d	31.63	280	13.39291	3.743398	+	1	14.66217	10.91877
O76021	RSL1D1	Ribosomal	54.973	490	13.36784	3.740694	+	1	14.11073	10.37003
Q68D85	NCR3LG1	Natural cyt	50.827	454	13.36143	3.740003	+	1	13.67342	9.933417
P68036	UBE2L3	Ubiquitin-co	17.862	154	13.34975	3.738741	+	1	14.44825	10.7095
Q8WVC0	LEO1	RNA polym	75.404	666	13.33733	3.737398	+	1	13.60189	9.864489
Q01130	SRSF2	Serine/argi	25.476	221	13.31694	3.73519	+	1	13.25476	9.519573
Q9NSD9	FARSB	Phenylalan	66.116	589	13.3017	3.733539	+	1	13.58049	9.846956
Q15008	PSMD6	26S protea	45.531	389	13.30109	3.733473	+	1	14.47959	10.74612
Q9ULX6	AKAP8L	A-kinase a	71.64	646	13.28428	3.731648	+	1	14.55081	10.81916
P18887	XRCC1	DNA repair	69.498	633	13.17407	3.71963	+	1	14.56474	10.84512
Q9UEY8	ADD3	Gamma-ad	79.155	706	13.17294	3.719505	+	1	12.80136	9.08186
P05549	TFAP2A	Transcripti	48.062	437	13.16398	3.718524	+	1	14.39084	10.67232
O75127	PTCD1	Pentatricop	78.856	700	13.14892	3.716872	+	1	13.40647	9.689599
P60903	S100A10	Protein S1(	11.203	97	13.14433	3.716368	+	2	19.38528	15.66891
Q7L9B9	EEPD1	Endonucle	62.403	569	13.12908	3.714693	+	1	14.58485	10.87015
P39748	FEN1	Flap endon	42.593	380	13.11116	3.712724	+	1	13.43045	9.717729
Q06787	FMR1	Synaptic fu	71.174	632	13.11027	3.712625	+	1	14.35128	10.63866
Q8N158	GPC2	Glypican-2	62.83	579	13.08889	3.710271	+	1	13.59047	9.880199
Q8IWZ3	ANKHD1	Ankyrin rep	269.458	2542	13.08608	3.709961	+	1	14.5354	10.82544
Q15014	MORF4L2	Mortality fa	32.308	288	13.07616	3.708867	+	1	14.92296	11.21409
Q6XZF7	DNMBP	Dynamin-b	177.347	1577	13.02763	3.703502	+	1	14.26099	10.55749
O43242	PSMD3	26S protea	60.978	534	12.99606	3.700002	+	1	14.3592	10.6592
Q8N3V7	SYNPO	Synaptopo	99.463	929	12.95832	3.695807	+	2	18.03698	14.34117
P54577	YARS1	Tyrosine--t	59.143	528	12.91333	3.69079	+	1	13.04827	9.357476
Q14258	TRIM25	E3 ubiquitir	70.973	630	12.8979	3.689064	+	1	13.44411	9.755045
Q9BQE3	TUBA1C	Tubulin alp	49.895	449	12.88401	3.68751	+	1	14.85151	11.16399
P61224	RAP1B	Ras-related	20.825	184	12.83287	3.681772	+	1	13.67783	9.996057
Q9UQN3	CHMP2B	Charged m	23.907	213	12.82794	3.681218	+	1	14.42522	10.744
Q9C0J8	WDR33	pre-mRNA	145.891	1336	12.81915	3.680229	+	1	14.16373	10.4835
Q86XJ1	GAS2L3	GAS2-like	75.214	694	12.74441	3.671793	+	1	14.70022	11.02843
P35556	FBN2	Fibrillin-2 C	314.775	2912	12.72421	3.669505	+	1	12.87137	9.201862
Q96EY5	MVB12A	Multivesicu	28.783	273	12.68659	3.665233	+	1	14.40627	10.74104
Q9BYD6	MRPL1	39S riboso	36.909	325	12.68035	3.664523	+	1	14.52577	10.86124
P10909	CLU	Clusterin C	52.495	449	12.67728	3.664174	+	2	14.31487	10.65069
P49903	SEPHS1	Selenide, w	42.911	392	12.67306	3.663693	+	1	12.4797	8.816012
Q96KB5	PBK	Lymphokin	36.085	322	12.6692	3.663253	+	1	13.78423	10.12097
O75380	NDUFS6	NADH deh	13.712	124	12.62659	3.658393	+	1	13.6356	9.977212
Q9H0L4	CSTF2T	Cleavage s	64.437	616	12.61619	3.657205	+	1	13.00866	9.351452
O00148	DDX39A	ATP-deper	49.13	427	12.61192	3.656716	+	1	14.50457	10.84785
Q9UEU0	VTI1B	Vesicle tra	26.688	232	12.58481	3.653611	+	2	16.36429	12.71068
Q8NC96	NECAP1	Adaptin ea	29.737	275	12.5828	3.653381	+	2	17.32105	13.66767
Q08495	DMTN	Dematin O	45.514	405	12.56042	3.650813	+	1	14.82352	11.1727
Q14203	DCTN1	Dynactin su	141.695	1278	12.54825	3.649415	+	1	14.51342	10.864
O43148	RNMT	mRNA cap	54.844	476	12.50289	3.644189	+	1	14.10918	10.46499
Q9H6X2	ANTXR1	Anthrax tox	62.789	564	12.48505	3.642129	+	1	14.06609	10.42396
Q7Z4G1	COMMD6	COMM dor	9.638	85	12.45799	3.638999	+	1	13.14534	9.506344

P48444	ARCN1	Coatomer s	57.21	511	12.41458	3.633964	+	1	14.27234	10.63837
Q13610	PWP1	Periodic try	55.828	501	12.4044	3.63278	+	1	13.74452	10.11174
Q3ZCQ8	TIMM50	Mitochondr	39.646	353	12.40059	3.632336	+	1	14.50965	10.87731
Q15633	TARBP2	RISC-loadi	39.039	366	12.39952	3.632212	+	1	14.71848	11.08627
Q969F2	NKD2	Protein nak	50.055	451	12.39314	3.63147	+	1	14.3172	10.68573
Q9Y295	DRG1	Developme	40.542	367	12.3924	3.631384	+	2	16.10679	12.4754
Q9HAV0	GNB4	Guanine nu	37.567	340	12.38476	3.630493	+	2	16.08088	12.45039
Q8N8S7	ENAH	Protein ena	66.51	591	12.38036	3.629982	+	1	15.13214	11.50216
Q9P0M9	MRPL27	39S riboso	16.073	148	12.37519	3.629379	+	1	12.68538	9.056005
O43660	PLRG1	Pleiotropic	57.194	514	12.32656	3.623698	+	1	13.88579	10.26209
Q9BTD8	RBM42	RNA-bindir	50.414	480	12.27088	3.617166	+	1	14.0121	10.39493
Q9BY43	CHMP4A	Charged m	25.098	222	12.26223	3.616149	+	1	14.80957	11.19342
Q03252	LMNB2	Lamin-B2 (	69.948	620	12.19206	3.60787	+	1	14.21136	10.60349
P35611	ADD1	Alpha-addu	80.955	737	12.15296	3.603235	+	1	13.57152	9.96828
P26599	PTBP1	Polypyrimic	57.221	531	12.14308	3.602063	+	2	17.54155	13.93949
Q9BY76	ANGPTL4	Angiopoieti	45.214	406	12.13322	3.600891	+	1	13.4921	9.891213
Q92804	TAF15	TATA-bind	61.83	592	12.11815	3.599097	+	2	16.15981	12.56071
Q96RT1	ERBIN	Erbin OS=I	158.298	1412	12.09074	3.595831	+	1	12.77304	9.177205
Q08209	PPP3CA	Serine/thre	58.688	521	12.05697	3.591795	+	1	13.73174	10.13995
P98179	RBM3	RNA-bindir	17.17	157	12.05494	3.591553	+	1	13.47244	9.880884
O95630	STAMBP	STAM-binc	48.077	424	12.04428	3.590276	+	1	14.05274	10.46246
Q9P2M7	CGN	Cingulin OS	137.057	1203	12.03986	3.589747	+	2	16.64808	13.05833
Q9Y4P3	TBL2	Transducin	49.798	447	12.02306	3.587732	+	1	13.97683	10.3891
Q6PJ61	FBXO46	F-box only	64.631	603	12.01818	3.587147	+	1	13.34541	9.758258
Q13547	HDAC1	Histone de	55.103	482	12.01168	3.586366	+	2	17.43274	13.84637
Q01518	CAP1	Adenylyl cy	51.901	475	11.99541	3.58441	+	1	13.23071	9.646301
P01889	HLA-B	HLA class	40.46	362	11.98652	3.583341	+	1	14.91336	11.33002
O00268	TAF4	Transcripti	110.114	1085	11.98442	3.583088	+	1	14.37972	10.79663
Q14192	FHL2	Four and a	32.193	279	11.96928	3.581265	+	1	14.87498	11.29372
O15173	PGRMC2	Membrane	23.818	223	11.91854	3.575136	+	1	13.38559	9.810457
O43237	DYNC1LI2	Cytoplasmi	54.099	492	11.90916	3.574	+	1	14.58555	11.01155
Q9H0B6	KLC2	Kinesin ligh	68.935	622	11.90533	3.573536	+	2	14.12024	10.5467
Q9H6F5	CCDC86	Coiled-coil	40.236	360	11.89035	3.57172	+	1	13.29949	9.727775
Q7Z6M4	MTERF4	Transcripti	43.958	381	11.81287	3.562288	+	1	13.88912	10.32684
Q9Y4L1	HYOU1	Hypoxia up	111.335	999	11.76175	3.556031	+	1	13.67706	10.12103
P31949	S100A11	Protein S1(	11.74	105	11.75387	3.555063	+	1	13.83723	10.28217
Q8NHG7	SVIP	Small VCP	8.443	77	11.68691	3.546822	+	1	14.27823	10.73141
Q6KC79	NIPBL	Nipped-B-li	316.051	2804	11.67976	3.545939	+	1	13.832	10.28606
Q155Q3	DIXDC1	Dixin OS=H	77.478	683	11.65496	3.542872	+	1	14.99603	11.45316
Q9BQG0	MYBBP1A	Myb-bindin	148.855	1328	11.62277	3.538882	+	2	14.52166	10.98278
Q8WU20	FRS2	Fibroblast (	57.029	508	11.61605	3.538048	+	1	14.19944	10.66139
O60884	DNAJA2	DnaJ homo	45.746	412	11.5903	3.534846	+	2	17.51222	13.97737
Q15029	EFTUD2	116 kDa U	109.436	972	11.57669	3.533151	+	2	14.71821	11.18506
Q9H074	PAIP1	Polyadenyl	53.525	479	11.568	3.532067	+	1	15.50069	11.96862
Q96A33	CCDC47	PAT compl	55.874	483	11.56715	3.531961	+	1	14.03652	10.50456
Q8IWU2	LMTK2	Serine/thre	164.9	1503	11.52924	3.527226	+	1	14.77992	11.2527
Q9BRK5	SDF4	45 kDa calc	41.807	362	11.52705	3.526951	+	1	15.65832	12.13137
O00487	PSMD14	26S protea	34.577	310	11.52438	3.526617	+	1	14.22679	10.70017
Q9GZP0	PDGFD	Platelet-de	42.848	370	11.52087	3.526178	+	1	15.08846	11.56228
Q96DH6	MSI2	RNA-bindir	35.197	328	11.51779	3.525792	+	1	14.02911	10.50332
Q9Y676	MRPS18B	28S riboso	29.396	258	11.4649	3.519152	+	1	14.07448	10.55532
Q8NEZ4	KMT2C	Histone-lys	541.37	4911	11.45941	3.518461	+	1	13.8745	10.35604
Q9NRX2	MRPL17	39S riboso	20.05	175	11.42523	3.514152	+	1	14.17866	10.66451
Q8NDI1	EHBP1	EH domain	140.017	1231	11.39596	3.510451	+	1	12.45054	8.940091
Q13136	PPFIA1	Liprin-alpha	135.779	1202	11.38019	3.508453	+	1	13.23095	9.722498
Q86TG7	PEG10	Retrotrans	80.173	708	11.36675	3.506748	+	1	14.33113	10.82438
Q6ZU35	CRACD	Capping pr	136.76	1233	11.35269	3.504962	+	2	16.25525	12.75029
P0CG39	POTEJ	POTE anky	117.39	1038	11.33131	3.502243	+	1	14.48822	10.98597
Q14197	MRPL58	Peptidyl-tR	23.63	206	11.31527	3.500199	+	1	13.53916	10.03896

Q9BUF5	TUBB6	Tubulin bet	49.857	446	11.30114	3.498397	+	2	15.56338	12.06498
Q9NPJ6	MED4	Mediator of	29.745	270	11.28821	3.496744	+	1	13.67408	10.17734
Q8NHZ8	CDC26	Anaphase-	9.777	85	11.28779	3.496691	+	1	13.87498	10.37829
P08559	PDHA1	Pyruvate de	43.296	390	11.26696	3.494026	+	1	14.46199	10.96796
Q8IXM2	BAP18	Chromatin	17.9	172	11.2544	3.492417	+	1	14.41277	10.92035
Q92908	GATA6	Transcripti	60.033	595	11.19798	3.485166	+	1	13.73312	10.24796
P04632	CAPNS1	Calpain sm	28.316	268	11.1771	3.482473	+	1	13.53223	10.04976
Q92614	MYO18A	Unconventi	233.115	2054	11.16098	3.480391	+	2	16.40076	12.92037
Q9NPD3	EXOSC4	Exosome c	26.383	245	11.15496	3.479613	+	1	14.65329	11.17368
O75396	SEC22B	Vesicle-tra	24.741	215	11.14898	3.47884	+	1	12.77037	9.291535
P28482	MAPK1	Mitogen-ac	41.39	360	11.11846	3.474884	+	1	14.93526	11.46037
P36969	GPX4	Phospholip	22.175	197	11.11308	3.474187	+	1	13.53795	10.06376
Q13112	CHAF1B	Chromatin	61.493	559	11.10442	3.473063	+	1	13.59887	10.1258
O60573	EIF4E2	Eukaryotic	28.362	245	11.0994	3.472409	+	1	14.15181	10.6794
O96019	ACTL6A	Actin-like p	47.461	429	11.09856	3.4723	+	2	15.55767	12.08537
Q8TB52	FBXO30	F-box only	82.304	745	11.09822	3.472257	+	1	13.16933	9.697073
Q93008	USP9X	Probable u	290.463	2554	11.04219	3.464955	+	1	12.96096	9.496006
Q13123	IK	Protein Re	65.602	557	11.03932	3.46458	+	1	14.54774	11.08316
O75348	ATP6V1G1	V-type prot	13.758	118	11.02163	3.462266	+	2	14.17874	10.71648
Q92600	CNOT9	CCR4-NOT	33.631	299	10.9986	3.459248	+	1	14.41396	10.95471
Q9BW61	DDA1	DET1- and	11.835	102	10.95312	3.45327	+	1	14.73344	11.28017
Q70SY1	CREB3L2	Cyclic AMF	57.415	520	10.90303	3.446657	+	1	12.59756	9.150906
Q8NDC0	MAPK1IP1	MAPK-inte	24.269	245	10.88817	3.444689	+	1	14.25333	10.80864
Q13868	EXOSC2	Exosome c	32.789	293	10.86391	3.441472	+	1	13.61574	10.17427
P30837	ALDH1B1	Aldehyde d	57.249	517	10.84273	3.438657	+	1	13.61114	10.17248
Q99567	NUP88	Nuclear po	83.542	741	10.81287	3.434678	+	1	13.0821	9.647421
Q6UN15	FIP1L1	Pre-mRNA	66.526	594	10.78167	3.430508	+	2	17.88917	14.45866
O75794	CDC123	Cell divisio	39.135	336	10.77256	3.42929	+	1	14.06836	10.63907
Q12788	TBL3	Transducin	89.035	808	10.73679	3.42449	+	1	14.12008	10.69559
P16422	EPCAM	Epithelial c	34.932	314	10.72229	3.422541	+	1	13.40461	9.982069
Q13098	GPS1	COP9 sign	55.537	491	10.71811	3.421978	+	1	13.28034	9.858358
P35226	BMI1	Polycomb c	36.949	326	10.68869	3.418013	+	1	13.19151	9.773493
P49792	RANBP2	E3 SUMO-	358.199	3224	10.66388	3.414661	+	1	13.18925	9.774593
Q9Y6V7	DDX49	Probable A	54.226	483	10.61957	3.408653	+	1	13.14582	9.737167
P42704	LRPPRC	Leucine-ric	157.905	1394	10.60451	3.406606	+	2	17.26527	13.85866
Q96IJ6	GMPPA	Mannose-1	46.291	420	10.53942	3.397723	+	1	12.90471	9.506987
Q9Y262	EIF3L	Eukaryotic	66.727	564	10.53206	3.396715	+	1	14.75906	11.36234
O43318	MAP3K7	Mitogen-ac	67.196	606	10.51967	3.395017	+	1	14.14983	10.75481
P35680	HNF1B	Hepatocyte	61.324	557	10.48506	3.390263	+	1	13.64374	10.25348
P20827	EFNA1	Ephrin-A1 (	23.787	205	10.47361	3.388687	+	1	13.38195	9.993262
O75128	COBL	Protein cor	135.617	1261	10.40093	3.37864	+	2	16.93802	13.55938
Q8WXE9	STON2	Stonin-2 O	101.165	905	10.39109	3.377275	+	1	14.156	10.77873
P05161	ISG15	Ubiquitin-lik	17.888	165	10.38925	3.37702	+	1	14.04311	10.66609
Q13409	DYNC1I2	Cytoplasmi	71.457	638	10.38436	3.376341	+	1	14.53004	11.1537
O00541	PES1	Pescadillo	68.003	588	10.3819	3.375998	+	1	13.77787	10.40187
P50570	DNM2	Dynamin-2	98.064	870	10.36804	3.374071	+	1	12.61137	9.237299
Q9UKB1	FBXW11	F-box/WD	62.091	542	10.35776	3.37264	+	1	13.44954	10.0769
Q9UBV8	PEF1	Peflin OS=	30.381	284	10.35695	3.372527	+	1	12.64024	9.267718
Q3SXY8	ARL13B	ADP-ribosy	48.643	428	10.33884	3.370003	+	1	13.7012	10.3312
Q96NB1	CEP20	Centrosom	19.778	174	10.32953	3.368702	+	1	14.27707	10.90837
O15550	KDM6A	Lysine-spe	154.177	1401	10.29832	3.364337	+	1	14.01463	10.65029
Q9Y2A7	NCKAP1	Nck-associ	128.79	1128	10.28022	3.3618	+	1	13.37341	10.01161
P28347	TEAD1	Transcripti	47.946	426	10.27677	3.361316	+	1	13.78392	10.4226
Q9UNS2	COPS3	COP9 sign	47.873	423	10.27024	3.360398	+	1	12.89858	9.538185
Q9C0C2	TNKS1BP1	182 kDa ta	181.796	1729	10.22986	3.354715	+	2	17.18582	13.83111
Q9NX55	HYPK	Huntingtin-	13.651	121	10.22787	3.354434	+	1	12.57573	9.221294
O15504	NUP42	Nucleopori	44.872	423	10.22302	3.353749	+	1	13.65161	10.29786
Q15691	MAPRE1	Microtubule	29.999	268	10.18949	3.34901	+	1	14.83556	11.48655
Q99418	CYTH2	Cytohesin-2	46.546	400	10.18192	3.347938	+	1	13.97522	10.62728

Q9H0V9	LMAN2L	VIP36-like	39.711	348	10.17619	3.347125	+	1	15.33469	11.98757
P35240	NF2	Merlin OS=	69.69	595	10.1646	3.345482	+	2	16.32129	12.97581
Q99569	PKP4	Plakophilin	131.868	1192	10.1632	3.345283	+	1	13.40674	10.06145
Q8NHU0	CT45A3	Cancer/tes	21.331	189	10.15992	3.344818	+	1	14.40095	11.05613
P08133	ANXA6	Annexin A6	75.873	673	10.14852	3.343198	+	1	12.09299	8.749791
Q9Y639	NPTN	Neuroplast	44.387	398	10.14689	3.342966	+	1	13.09077	9.747808
P07814	EPRS1	Bifunctiona	170.591	1512	10.12224	3.339457	+	1	14.10901	10.76956
Q9H900	ZWILCH	Protein zwi	67.214	591	10.11256	3.338077	+	2	15.97107	12.633
P52294	KPNA1	Importin su	60.222	538	10.09399	3.335425	+	1	15.05036	11.71493
O00232	PSMD12	26S protea	52.904	456	10.09386	3.335406	+	1	13.69816	10.36276
Q7Z589	EMSY	BRCA2-inte	141.468	1322	10.08248	3.333779	+	2	15.84945	12.51568
Q9H0G5	NSRP1	Nuclear sp	66.39	558	10.06946	3.331914	+	1	13.01275	9.680833
Q8IY33	MICALL2	MICAL-like	97.502	904	10.06832	3.331752	+	1	13.55926	10.22751
Q9BR76	CORO1B	Coronin-1E	54.235	489	10.06792	3.331694	+	1	15.26419	11.93249
Q10471	GALNT2	Polypeptide	64.733	571	10.06629	3.33146	+	1	12.2788	8.947338
Q9UBB4	ATXN10	Ataxin-10 (	53.489	475	10.05716	3.330151	+	1	13.69392	10.36377
O15234	CASC3	Protein CA	76.278	703	10.05001	3.329125	+	1	13.10115	9.77203
Q9BTL3	RAMAC	RNA guani	14.381	118	10.04085	3.32781	+	1	14.13178	10.80397
P61244	MAX	Protein ma	18.275	160	9.980316	3.319086	+	1	13.81087	10.49179
P26232	CTNNA2	Catenin alp	105.313	953	9.968237	3.317338	+	1	14.50631	11.18897
Q5T5U3	ARHGAP2	Rho GTPa:	217.462	1958	9.953265	3.31517	+	1	14.13467	10.8195
Q8IZP9	ADGRG2	Adhesion (	111.593	1017	9.937933	3.312946	+	1	13.7276	10.41466
Q8NFH3	NUP43	Nucleopori	42.151	380	9.921928	3.31062	+	1	13.02034	9.709717
Q9BYD3	MRPL4	39S riboso	34.919	311	9.884778	3.305209	+	1	13.77582	10.47061
Q9BQE5	APOL2	Apolipoprot	37.092	337	9.858992	3.30144	+	1	13.58285	10.28141
Q15785	TOMM34	Mitochondr	34.559	309	9.850063	3.300133	+	1	13.47256	10.17243
Q8WUP2	FBLIM1	Filamin-bin	40.67	373	9.826246	3.29664	+	1	13.76342	10.46678
Q9GZP4	PITHD1	PITH doma	24.178	211	9.826144	3.296625	+	1	13.62822	10.33159
P53985	SLC16A1	Monocarbo	53.944	500	9.822977	3.29616	+	1	14.16428	10.86812
P19022	CDH2	Cadherin-2	99.809	906	9.822829	3.296139	+	1	14.09094	10.7948
Q8N5N7	MRPL50	39S riboso	18.325	158	9.818266	3.295468	+	1	14.46135	11.16588
Q05682	CALD1	Caldesmor	93.231	793	9.796256	3.29223	+	2	18.99281	15.70058
Q96CP2	FLYWCH2	FLYWCH f	14.564	140	9.793033	3.291756	+	1	13.46608	10.17432
P49643	PRIM2	DNA prima	58.806	509	9.752786	3.285814	+	1	13.56546	10.27964
Q99543	DNAJC2	DnaJ homo	71.996	621	9.739451	3.28384	+	1	13.76176	10.47792
Q9BTT0	ANP32E	Acidic leuc	30.692	268	9.73338	3.282941	+	1	12.75904	9.476094
Q12874	SF3A3	Splicing fac	58.849	501	9.732895	3.282869	+	2	15.32822	12.04535
O15155	BET1	BET1 hom	13.289	118	9.728007	3.282144	+	1	14.37904	11.0969
Q9Y2D5	AKAP2	A-kinase ar	94.661	859	9.720614	3.281047	+	2	15.53117	12.25012
Q07157	TJP1	Tight juncti	195.459	1748	9.718428	3.280723	+	2	17.49717	14.21644
P51398	DAP3	28S riboso	45.566	398	9.713822	3.280039	+	2	15.08365	11.80361
O60610	DIAPH1	Protein dia	141.347	1272	9.70278	3.278398	+	1	13.70369	10.42529
P28072	PSMB6	Proteasom	25.358	239	9.689187	3.276376	+	1	12.83206	9.555684
P07203	GPX1	Glutathione	22.088	203	9.681281	3.275198	+	1	12.95057	9.675376
P60900	PSMA6	Proteasom	27.399	246	9.674904	3.274247	+	2	14.43058	11.15634
Q71DI3	H3C15	Histone H3	15.388	136	9.66417	3.272646	+	1	14.0991	10.82646
O15212	PFDN6	Prefoldin su	14.583	129	9.661671	3.272273	+	1	14.34027	11.06799
Q5XKP0	MICOS13	MICOS cor	13.087	118	9.654784	3.271244	+	1	13.55579	10.28454
Q12931	TRAP1	Heat shock	80.11	704	9.654758	3.27124	+	1	13.46148	10.19024
O15355	PPM1G	Protein pho	59.272	546	9.654113	3.271144	+	2	15.29563	12.02448
P20618	PSMB1	Proteasom	26.489	241	9.64546	3.26985	+	1	13.69849	10.42864
Q9NP97	DYNLRB1	Dynein ligh	10.922	96	9.615754	3.2654	+	1	14.54466	11.27926
P08582	MELTF	Melanotran	80.215	738	9.608139	3.264257	+	1	13.73524	10.47098
Q86YM7	HOMER1	Homer prot	40.277	354	9.604284	3.263678	+	2	15.90035	12.63667
P19404	NDUFV2	NADH deh	27.392	249	9.596821	3.262557	+	1	13.45288	10.19033
P46109	CRKL	Crk-like pro	33.777	303	9.596715	3.262541	+	1	14.40188	11.13934
Q9P015	MRPL15	39S riboso	33.42	296	9.587586	3.261168	+	1	12.98661	9.725439
Q02487	DSC2	Desmocolli	99.962	901	9.58056	3.26011	+	1	14.54454	11.28443
Q15019	SEPTIN2	Septin-2 O	41.487	361	9.579729	3.259985	+	2	17.39858	14.13859

P82933	MRPS9	28S riboso	45.835	396	9.576287	3.259466	+	1	15.18909	11.92962
P07741	APRT	Adenine ph	19.608	180	9.529523	3.252404	+	1	14.09589	10.84349
O60825	PFKFB2	6-phosphof	58.477	505	9.470984	3.243514	+	2	15.80914	12.56563
P46379	BAG6	Large proli	119.409	1132	9.46112	3.242011	+	1	12.67194	9.429928
Q9Y314	NOSIP	Nitric oxide	33.172	301	9.441	3.23894	+	2	17.5947	14.35576
Q9C0B1	FTO	Alpha-keto	58.282	505	9.439092	3.238648	+	1	12.56129	9.32264
P10114	RAP2A	Ras-relatec	20.615	183	9.427443	3.236867	+	1	13.37599	10.13912
O00291	HIP1	Huntingtin-	116.221	1037	9.393304	3.231633	+	2	16.2595	13.02787
P05556	ITGB1	Integrin bet	88.415	798	9.391554	3.231364	+	1	13.89311	10.66175
Q96QC0	PPP1R10	Serine/thre	99.058	940	9.386127	3.23053	+	1	13.01324	9.782706
Q99873	PRMT1	Protein arg	42.462	371	9.365103	3.227295	+	2	16.96285	13.73556
Q9Y3Y2	CHTOP	Chromatin	26.397	248	9.361983	3.226814	+	1	14.10877	10.88196
P53597	SUCLG1	Succinate--	36.25	346	9.355948	3.225884	+	1	11.67627	8.450382
Q96CF2	CHMP4C	Charged m	26.411	233	9.351966	3.22527	+	1	13.5243	10.29903
P45877	PPIC	Peptidyl-pr	22.763	212	9.32976	3.22184	+	1	14.66489	11.44305
Q9NP66	HMG20A	High mobili	40.144	347	9.314272	3.219443	+	1	13.5265	10.30706
Q99627	COPS8	COP9 sign	23.226	209	9.274601	3.213285	+	1	14.54557	11.33228
Q9NVP1	DDX18	ATP-deper	75.407	670	9.271322	3.212775	+	1	13.74997	10.5372
Q96BK5	PINX1	PIN2/TERF	37.035	328	9.254075	3.210089	+	1	12.90015	9.690061
Q9NQT5	EXOSC3	Exosome c	29.572	275	9.242351	3.20826	+	1	13.57033	10.36207
Q96G74	OTUD5	OTU doma	60.626	571	9.223209	3.205269	+	1	13.71864	10.51337
Q9H299	SH3BGRL3	SH3 domai	10.438	93	9.211225	3.203393	+	1	12.83875	9.635358
Q9BQ39	DDX50	ATP-deper	82.565	737	9.206994	3.20273	+	1	13.56296	10.36023
Q9UG63	ABCF2	ATP-bindin	71.29	623	9.18138	3.198711	+	2	15.72704	12.52833
P48960	ADGRE5	Adhesion C	91.869	835	9.179615	3.198434	+	1	13.3833	10.18487
O14545	TRAFD1	TRAF-type	64.841	582	9.15293	3.194234	+	1	13.21902	10.02478
Q6FI81	CIAPIN1	Anamorsin	33.582	312	9.148516	3.193538	+	1	12.28581	9.092269
Q9H2U2	PPA2	Inorganic p	37.92	334	9.147131	3.193319	+	1	13.8535	10.66019
P53567	CEBPG	CCAAT/en	16.408	150	9.142001	3.19251	+	1	13.84784	10.65533
Q9ULW0	TPX2	Targeting p	85.653	747	9.13124	3.190811	+	1	13.79065	10.59984
O14579	COPE	Coatomer s	34.482	308	9.130462	3.190688	+	2	15.82908	12.63839
P00403	MT-CO2	Cytochrom	25.565	227	9.119017	3.188878	+	1	15.30028	12.1114
Q9NZL4	HSPBP1	Hsp70-binc	39.303	359	9.099604	3.185804	+	1	13.74956	10.56375
Q7L576	CYFIP1	Cytoplasmi	145.182	1253	9.095926	3.18522	+	1	11.42862	8.243401
Q96DE5	ANAPC16	Anaphase-	11.667	110	9.082053	3.183018	+	1	13.09102	9.908004
Q969S3	ZNF622	Zinc finger	54.272	477	9.059814	3.179481	+	1	14.27081	11.09132
Q9NZB2	FAM120A	Constitutive	121.888	1118	9.05343	3.178465	+	2	14.99112	11.81266
Q96PU8	QKI	Protein qua	37.671	341	9.053	3.178396	+	1	13.47269	10.29429
P98175	RBM10	RNA-bindir	103.533	930	9.038705	3.176116	+	1	13.91681	10.7407
O75935	DCTN3	Dynactin su	21.119	186	9.00294	3.170396	+	1	14.56695	11.39655
P29375	KDM5A	Lysine-spe	192.095	1690	8.991364	3.16854	+	1	14.98081	11.81227
P52926	HMGA2	High mobili	11.832	109	8.990964	3.168476	+	1	14.03557	10.8671
P41252	IARS1	Isoleucine-	144.498	1262	8.976506	3.166154	+	2	14.19537	11.02922
P78347	GTF2I	General tra	112.416	998	8.96819	3.164817	+	2	15.67758	12.51277
P62314	SNRPD1	Small nucle	13.282	119	8.94574	3.161201	+	1	14.96104	11.79984
P40424	PBX1	Pre-B-cell l	46.626	430	8.938638	3.160055	+	1	12.89186	9.731805
P55010	EIF5	Eukaryotic	49.223	431	8.932651	3.159088	+	1	12.57745	9.418364
Q13492	PICALM	Phosphatid	70.755	652	8.891441	3.152417	+	2	16.67328	13.52086
Q7L5N1	COPS6	COP9 sign	36.163	327	8.844756	3.144822	+	1	13.17975	10.03493
Q9NY61	AATF	Protein AA	63.133	560	8.838674	3.14383	+	1	14.28273	11.1389
Q8IYD1	GSPT2	Eukaryotic	68.883	628	8.826589	3.141856	+	1	14.44191	11.30005
Q96B26	EXOSC8	Exosome c	30.04	276	8.81569	3.140074	+	1	14.33518	11.19511
Q9NUL5	SHFL	Shiftless ar	33.11	291	8.786846	3.135345	+	1	12.56339	9.428041
P62333	PSMC6	26S protea	44.173	389	8.778729	3.134012	+	2	15.55342	12.41941
O75351	VPS4B	Vacuolar p	49.302	444	8.762879	3.131405	+	1	12.80748	9.676071
Q9UNL2	SSR3	Translocon	21.081	185	8.755558	3.130199	+	1	13.99841	10.86822
P62906	RPL10A	60S riboso	24.831	217	8.750659	3.129392	+	2	18.34082	15.21143
O15294	OGT	UDP-N-ace	116.925	1046	8.740844	3.127773	+	1	14.73392	11.60614
Q9BXS6	NUSAP1	Nucleolar a	49.452	441	8.709148	3.122532	+	1	14.75744	11.63491

P49366	DHPS	Deoxyhypu	40.971	369	8.691749	3.119647	+	1	14.20694	11.08729
Q9UNS1	TIMELESS	Protein tim	138.658	1208	8.681	3.117861	+	1	13.29002	10.17216
P11586	MTHFD1	C-1-tetrahy	101.531	935	8.667955	3.115692	+	2	14.83165	11.71596
Q8N7R7	CCNYL1	Cyclin-Y-lik	40.705	359	8.643218	3.111569	+	1	13.41996	10.30839
Q8IYL3	C1orf174	UPF0688 p	25.977	243	8.634524	3.110117	+	1	13.38343	10.27332
Q15434	RBMS2	RNA-bindir	43.959	407	8.630411	3.109429	+	1	13.14003	10.0306
Q9BV38	WDR18	WD repeat	47.405	432	8.614308	3.106735	+	1	13.46301	10.35628
P19086	GNAZ	Guanine nu	40.924	355	8.611184	3.106212	+	1	14.01088	10.90467
Q9NYU2	UGGT1	UDP-gluco	177.19	1555	8.599152	3.104194	+	1	13.3372	10.23301
Q9Y5Y2	NUBP2	Cytosolic F	28.825	271	8.586152	3.102012	+	1	12.86525	9.763238
Q6ZT12	UBR3	E3 ubiquitir	212.433	1888	8.584093	3.101666	+	1	12.41424	9.31257
Q9NWH9	SLTM	SAFB-like t	117.148	1034	8.578673	3.100754	+	1	13.73386	10.63311
P07948	LYN	Tyrosine-pı	58.574	512	8.565861	3.098598	+	2	16.85672	13.75812
Q9BY77	POLDIP3	Polymerase	46.089	421	8.563397	3.098183	+	1	13.37096	10.27278
Q9Y4Z0	LSM4	U6 snRNA-	15.35	139	8.561715	3.0979	+	1	12.96672	9.868822
P85037	FOXK1	Forkhead b	75.457	733	8.542383	3.094639	+	1	13.68891	10.59427
Q99959	PKP2	Plakophilin	97.415	881	8.506433	3.088554	+	1	13.75895	10.6704
O15182	CETN3	Centrin-3 C	19.55	167	8.504446	3.088217	+	1	12.72377	9.63555
Q9ULL1	PLEKHG1	Pleckstrin h	155.44	1385	8.493217	3.086311	+	1	14.16137	11.07506
P22695	UQCRC2	Cytochrom	48.443	453	8.479858	3.08404	+	1	13.39473	10.31069
O43395	PRPF3	U4/U6 sma	77.529	683	8.439951	3.077235	+	1	14.03729	10.96005
P22314	UBA1	Ubiquitin-lik	117.849	1058	8.439191	3.077105	+	2	16.21576	13.13866
Q86SQ0	PHLDB2	Pleckstrin h	142.158	1253	8.432502	3.075961	+	1	14.4611	11.38513
Q6P6C2	ALKBH5	RNA deme	44.256	394	8.425064	3.074688	+	1	13.40075	10.32606
Q69YN2	CWF19L1	CWF19-like	60.619	538	8.418275	3.073525	+	1	11.81686	8.743339
Q96EV2	RBM33	RNA-bindir	129.986	1170	8.38827	3.068373	+	1	13.2619	10.19353
Q92552	MRPS27	28S riboso	47.611	414	8.384914	3.067796	+	2	15.57923	12.51143
Q16352	INA	Alpha-inter	55.391	499	8.376282	3.06631	+	1	14.16867	11.10236
Q96BJ3	AIDA	Axin intera	35.023	306	8.366469	3.064619	+	1	14.02219	10.95758
P51452	DUSP3	Dual specif	20.478	185	8.366061	3.064548	+	1	12.91113	9.846581
P08579	SNRPB2	U2 small nu	25.486	225	8.351216	3.061986	+	1	13.73217	10.67018
O00425	IGF2BP3	Insulin-like	63.705	579	8.343887	3.06072	+	2	15.8905	12.82978
P36383	GJC1	Gap junctio	45.47	396	8.343217	3.060604	+	1	12.54673	9.486122
Q8WXI9	GATAD2B	Transcriptio	65.261	593	8.335766	3.059315	+	1	13.47624	10.41693
O75643	SNRNP200	U5 small nu	244.508	2136	8.31101	3.055024	+	1	14.43202	11.377
Q8TBX8	PIP4K2C	Phosphatid	47.3	421	8.273561	3.048508	+	1	13.75405	10.70554
Q13895	BYSL	Bystin OS=	49.601	437	8.272281	3.048285	+	1	13.61517	10.56688
P49721	PSMB2	Proteasom	22.836	201	8.269259	3.047758	+	1	13.87152	10.82376
Q9BZL4	PPP1R12C	Protein pho	84.881	782	8.268397	3.047608	+	1	14.90115	11.85354
P30260	CDC27	Cell divisio	91.867	824	8.265113	3.047034	+	1	13.74578	10.69874
Q96ST3	SIN3A	Paired amp	145.175	1273	8.264738	3.046969	+	1	12.90588	9.858907
O60832	DKC1	H/ACA ribo	57.674	514	8.264173	3.04687	+	1	14.22106	11.17419
Q08431	MFGE8	Lactadherin	43.105	387	8.244698	3.043467	+	2	14.61626	11.57279
Q53H12	AGK	Acylglycero	47.137	422	8.243907	3.043328	+	1	12.62393	9.580599
P28290	ITPRID2	Protein ITP	138.386	1259	8.239972	3.042639	+	2	14.89969	11.85705
O75330	HMMR	Hyaluronar	84.1	724	8.222171	3.039519	+	1	13.8876	10.84808
P48507	GCLM	Glutamate-	30.727	274	8.210402	3.037453	+	1	13.68803	10.65058
Q13393	PLD1	Phospholip	124.184	1074	8.201273	3.035848	+	1	14.22957	11.19372
B2RUZ4	SMIM1	Small integ	8.749	78	8.186251	3.033203	+	1	13.45982	10.42661
Q9NP79	VTA1	Vacuolar p	33.879	307	8.176985	3.031569	+	1	13.73714	10.70557
O60502	OGA	Protein O-(	102.915	916	8.168971	3.030154	+	1	12.60003	9.569875
Q9Y6M4	CSNK1G3	Casein kina	51.389	447	8.162777	3.02906	+	2	16.20766	13.1786
Q13573	SNW1	SNW doma	61.494	536	8.158605	3.028323	+	2	17.14629	14.11797
Q8N6M0	OTUD6B	Deubiquitin	33.813	293	8.157172	3.028069	+	1	12.47112	9.443047
Q86WJ1	CHD1L	Chromodo	101	897	8.154162	3.027537	+	1	13.41032	10.38278
P28289	TMOD1	Tropomodu	40.569	359	8.144863	3.02589	+	2	15.30538	12.27949
P51570	GALK1	Galactokina	42.272	392	8.122203	3.021871	+	1	13.44243	10.42055
P11908	PRPS2	Ribose-pho	34.769	318	8.069793	3.012532	+	1	13.82496	10.81243
O00217	NDUFS8	NADH deh	23.705	210	8.064998	3.011674	+	1	13.68168	10.67

Q13907	IDI1	Isopenteny	26.319	227	8.037899	3.006818	+	1	13.31388	10.30706
P48729	CSNK1A1	Casein kin	38.915	337	8.036898	3.006639	+	1	14.81178	11.80514
Q5JTV8	TOR1AIP1	Torsin-1A-i	66.248	583	8.002801	3.000505	+	1	12.7174	9.716893
Q8TAQ2	SMARCC2	SWI/SNF c	132.879	1214	7.993589	2.998843	+	2	14.57684	11.578
P30419	NMT1	Glycylpepti	56.806	496	7.990478	2.998282	+	1	13.89879	10.90051
Q96KR1	ZFR	Zinc finger	117.012	1074	7.984363	2.997177	+	2	14.38842	11.39124
Q02809	PLOD1	Procollage	83.55	727	7.974417	2.995379	+	1	13.99081	10.99543
Q9Y2H5	PLEKHA6	Pleckstrin h	117.128	1048	7.966527	2.993951	+	1	13.26843	10.27447
P49257	LMAN1	Protein ER	57.549	510	7.96495	2.993665	+	1	12.04596	9.052299
P35998	PSMC2	26S protea	48.634	433	7.943883	2.989844	+	1	14.48822	11.49837
Q8WXF1	PSPC1	Paraspeckl	58.744	523	7.920274	2.98555	+	1	13.95292	10.96737
O60547	GMDS	GDP-manr	41.95	372	7.90748	2.983218	+	1	13.1483	10.16508
Q16531	DDB1	DNA dama	126.968	1140	7.901186	2.982069	+	2	15.55579	12.57372
P52943	CRIP2	Cysteine-ri	22.493	208	7.891137	2.980233	+	1	13.36208	10.38185
P54920	NAPA	Alpha-solul	33.233	295	7.887081	2.979491	+	1	12.87427	9.894779
Q9BXP5	SRRT	Serrate RN	100.666	876	7.881363	2.978445	+	2	14.25643	11.27798
Q9Y4W6	AFG3L2	AFG3-like	88.584	797	7.872571	2.976835	+	1	12.14851	9.171674
Q99575	POP1	Ribonuclea	114.709	1024	7.860725	2.974662	+	1	12.83678	9.862118
Q96E39	RBMXL1	RNA bindir	42.142	390	7.859333	2.974407	+	1	12.09981	9.125401
Q9P016	THYN1	Thymocyte	25.697	225	7.856726	2.973928	+	2	14.20335	11.22942
Q9Y230	RUVBL2	RuvB-like 2	51.157	463	7.851691	2.973003	+	2	16.31035	13.33734
Q86X55	CARM1	Histone-arg	65.854	608	7.851053	2.972886	+	1	13.29275	10.31987
P17931	LGALS3	Galectin-3	26.152	250	7.849624	2.972624	+	2	18.98123	16.0086
Q13015	MLLT11	Protein AF	10.061	90	7.81963	2.9671	+	1	13.94581	10.97871
Q9NXW2	DNAJB12	DnaJ homo	41.86	375	7.817742	2.966752	+	1	13.09564	10.12889
Q5RKV6	EXOSC6	Exosome c	28.235	272	7.815604	2.966357	+	2	14.25222	11.28586
Q8NF37	LPCAT1	Lysophosp	59.151	534	7.806847	2.96474	+	1	12.90695	9.942207
P12532	CKMT1A	Creatine ki	47.037	417	7.800322	2.963534	+	1	13.52613	10.5626
Q9UHB9	SRP68	Signal reco	70.73	627	7.788717	2.961386	+	1	13.6322	10.67081
Q9BY32	ITPA	Inosine trip	21.446	194	7.760451	2.95614	+	1	12.69701	9.740871
Q86UN2	RTN4RL1	Reticulon-4	49.065	441	7.709927	2.946717	+	1	13.85526	10.90854
Q3V6T2	CCDC88A	Girdin OS=	216.042	1871	7.688158	2.942638	+	1	12.61255	9.669908
P61966	AP1S1	AP-1 comp	18.733	158	7.684872	2.942021	+	1	13.52833	10.58631
O60925	PFDN1	Prefoldin s	14.21	122	7.682095	2.9415	+	1	13.2766	10.3351
Q5JVS0	HABP4	Intracellula	45.785	413	7.671776	2.939561	+	1	12.83006	9.890499
Q9NSE4	IARS2	Isoleucine-	113.792	1012	7.628296	2.931361	+	1	12.25343	9.322073
O95486	SEC24A	Protein trar	119.749	1093	7.625658	2.930862	+	1	13.84068	10.90982
P17544	ATF7	Cyclic AMF	51.757	483	7.614798	2.928806	+	1	13.04209	10.11328
O43663	PRC1	Protein reg	71.607	620	7.61223	2.928319	+	2	15.30417	12.37585
Q9BV86	NTMT1	N-terminal	25.387	223	7.60098	2.926186	+	1	14.0189	11.09271
O75874	IDH1	Isocitrate d	46.659	414	7.599591	2.925922	+	2	14.78612	11.86019
Q9H9H4	VPS37B	Vacuolar p	31.307	285	7.591948	2.92447	+	1	13.23326	10.30879
Q99961	SH3GL1	Endophilin-	41.49	368	7.590802	2.924252	+	2	15.34176	12.41751
O00566	MPHOSPH	U3 small nu	78.864	681	7.574784	2.921205	+	1	13.45546	10.53425
O75175	CNOT3	CCR4-NOT	81.872	753	7.552534	2.916961	+	1	14.83728	11.92032
O00330	PDHX	Pyruvate de	54.122	501	7.54839	2.916169	+	2	16.04437	13.1282
P02786	TFRC	Transferrin	84.871	760	7.542721	2.915085	+	1	13.52784	10.61276
P78318	IGBP1	Immunoglo	39.222	339	7.527957	2.912258	+	1	12.42259	9.510332
O15160	POLR1C	DNA-direct	39.25	346	7.503851	2.907631	+	2	15.68316	12.77553
Q9H201	EPN3	Epsin-3 OS	68.222	632	7.496077	2.906136	+	1	12.6446	9.738464
O75326	SEMA7A	Semaphori	74.824	666	7.485909	2.904178	+	1	12.96694	10.06276
Q9UM00	TMCO1	Calcium loa	27.079	239	7.471773	2.901451	+	1	13.16604	10.26459
Q5SSJ5	HP1BP3	Heterochro	61.207	553	7.470484	2.901202	+	1	14.02401	11.12281
Q9UBW8	COPS7A	COP9 sign	30.277	275	7.461338	2.899434	+	1	13.1532	10.25377
Q9Y6M1	IGF2BP2	Insulin-like	66.121	599	7.449773	2.897197	+	2	14.79568	11.89849
Q6P582	MZT2A	Mitotic-spir	16.221	158	7.434657	2.894266	+	1	14.09606	11.20179
O60784	TOM1	Target of M	53.818	492	7.416036	2.890648	+	1	12.88364	9.992988
P52434	POLR2H	DNA-direct	17.143	150	7.39741	2.88702	+	2	14.79045	11.90343
Q9UPN4	CEP131	Centrosom	122.149	1083	7.391162	2.885801	+	2	15.23085	12.34504

Q15165	PON2	Serum para	39.381	354	7.369783	2.881622	+	1	13.58226	10.70064
O75663	TIPRL	TIP41-like	31.444	272	7.36813	2.881299	+	1	12.82159	9.940296
P61158	ACTR3	Actin-relate	47.371	418	7.363768	2.880444	+	2	17.3532	14.47275
Q14204	DYNC1H1	Cytoplasmi	532.408	4646	7.363211	2.880335	+	2	13.07853	10.1982
Q6H8Q1	ABLIM2	Actin-bindi	67.812	611	7.362272	2.880151	+	1	12.90253	10.02237
P61758	VBP1	Prefoldin s	22.626	197	7.352681	2.87827	+	1	13.60142	10.72315
P50579	METAP2	Methionine	52.892	478	7.347794	2.877311	+	1	12.6223	9.744992
Q96ST2	IWS1	Protein IW	91.955	819	7.34198	2.876169	+	1	13.75196	10.87579
Q9NSK0	KLC4	Kinesin ligh	68.64	619	7.324558	2.872742	+	1	12.60701	9.734265
P62837	UBE2D2	Ubiquitin-c	16.735	147	7.322471	2.872331	+	1	15.02367	12.15134
P51553	IDH3G	Isocitrate d	42.794	393	7.318835	2.871614	+	1	11.97302	9.101402
Q9HC36	MRM3	rRNA meth	47.02	420	7.309664	2.869805	+	1	12.70967	9.83986
Q9Y2X7	GIT1	ARF GTPa	84.341	761	7.299161	2.867731	+	1	12.70557	9.837837
Q00341	HDLBP	Vigilin OS=	141.456	1268	7.296577	2.86722	+	2	15.41881	12.55159
Q14527	HLTF	Helicase-lik	113.929	1009	7.288078	2.865538	+	1	12.98575	10.12021
Q4KMP7	TBC1D10E	TBC1 dom	87.199	808	7.282101	2.864355	+	1	14.32811	11.46376
O95785	WIZ	Protein Wiz	178.674	1651	7.270861	2.862126	+	1	12.03218	9.170057
Q92665	MRPS31	28S riboso	45.318	395	7.270564	2.862067	+	1	14.04542	11.18335
Q96AE4	FUBP1	Far upstrea	67.56	644	7.251916	2.858362	+	1	14.35032	11.49196
Q9NY47	CACNA2D	Voltage-de	129.817	1150	7.248365	2.857656	+	1	13.52833	10.67068
Q53GG5	PDLIM3	PDZ and L	39.232	364	7.235332	2.855059	+	1	13.46722	10.61216
P14735	IDE	Insulin-deg	117.969	1019	7.206035	2.849206	+	1	13.39312	10.54392
Q96S97	MYADM	Myeloid-as	35.274	322	7.204556	2.848909	+	1	12.6986	9.849687
Q9BVW5	TIPIN	TIMELESS	34.555	301	7.173494	2.842676	+	1	13.68135	10.83867
Q9UI08	EVL	Ena/VASP-	44.62	416	7.165627	2.841093	+	1	12.94136	10.10027
Q9Y6E0	STK24	Serine/thre	49.308	443	7.14648	2.837233	+	1	12.86473	10.02749
Q15904	ATP6AP1	V-type prot	52.026	470	7.145864	2.837108	+	1	13.55039	10.71328
Q9BUL8	PDCD10	Programm	24.702	212	7.14502	2.836938	+	1	13.81328	10.97634
Q13155	AIMP2	Aminoacyl	35.349	320	7.133482	2.834606	+	1	14.98646	12.15186
P61020	RAB5B	Ras-relatec	23.707	215	7.125064	2.832903	+	1	12.85378	10.02087
P25685	DNAJB1	DnaJ homc	38.044	340	7.124932	2.832876	+	2	15.06584	12.23296
P13164	IFITM1	Interferon-i	13.964	125	7.122559	2.832396	+	1	12.7324	9.900005
P13693	TPT1	Translation	19.595	172	7.115507	2.830966	+	1	13.82118	10.99021
P35249	RFC4	Replication	39.682	363	7.111074	2.830068	+	1	14.10509	11.27502
Q15542	TAF5	Transcripti	86.83	800	7.109498	2.829748	+	1	13.04043	10.21068
P30044	PRDX5	Peroxiredo	22.086	214	7.106877	2.829216	+	1	14.05545	11.22624
P63167	DYNLL1	Dynein ligh	10.366	89	7.080966	2.823946	+	2	17.6757	14.85175
O75052	NOS1AP	Carboxyl-te	56.15	506	7.078747	2.823494	+	1	12.76084	9.937351
P42566	EPS15	Epidermal	98.656	896	7.040357	2.815649	+	2	15.56926	12.75361
P48643	CCT5	T-complex	59.671	541	7.039679	2.81551	+	2	16.0552	13.23969
Q8NFP9	NBEA	Neurobeac	327.822	2946	7.016049	2.810659	+	1	13.24243	10.43177
P11177	PDHB	Pyruvate d	39.233	359	6.999805	2.807315	+	1	14.71398	11.90666
Q8IVF7	FMNL3	Formin-like	117.213	1028	6.996647	2.806664	+	1	13.74147	10.9348
P18754	RCC1	Regulator c	44.969	421	6.976558	2.802515	+	1	12.64885	9.846336
P78563	ADARB1	Double-stra	80.763	741	6.947751	2.796546	+	1	13.51249	10.71595
O94929	ABLIM3	Actin-bindi	77.802	683	6.924971	2.791808	+	1	12.66425	9.872439
P14859	POU2F1	POU doma	76.472	743	6.921521	2.791089	+	1	13.7346	10.94351
P40121	CAPG	Macrophag	38.499	348	6.921435	2.791071	+	1	13.2798	10.48873
Q08945	SSRP1	FACT com	81.075	709	6.908272	2.788325	+	1	12.60587	9.817548
Q9P258	RCC2	Protein RC	56.085	522	6.90524	2.787692	+	2	15.80087	13.01318
O14929	HAT1	Histone ac	49.541	419	6.904955	2.787632	+	1	13.23283	10.44519
P10636	MAPT	Microtubule	78.928	758	6.90005	2.786607	+	1	13.039	10.2524
Q92973	TNPO1	Transportir	102.355	898	6.891564	2.784831	+	1	12.54209	9.757257
Q9HAW4	CLSPN	Claspin OS	151.094	1339	6.883253	2.783091	+	1	13.49436	10.71127
Q13625	TP53BP2	Apoptosis-	125.616	1128	6.883041	2.783046	+	1	14.44268	11.65964
O60568	PLOD3	Multifunctic	84.785	738	6.882581	2.78295	+	2	14.20143	11.41849
Q8N4Q1	CHCHD4	Mitochondr	15.996	142	6.880754	2.782567	+	1	13.50891	10.72634
P25490	YY1	Transcripti	44.713	414	6.871669	2.780661	+	1	13.72824	10.94758
P09110	ACAA1	3-ketoacyl-	44.292	424	6.87044	2.780402	+	1	13.02585	10.24545

Q9Y6R9	CCDC61	Centrosom	57.368	512	6.851864	2.776496	+	1	13.2538	10.47731
Q9NPF5	DMAP1	DNA methy	52.993	467	6.84664	2.775396	+	1	12.66309	9.887695
Q9BZI7	UPF3B	Regulator c	57.762	483	6.838956	2.773776	+	1	13.23489	10.46112
Q9P035	HACD3	Very-long-c	43.16	362	6.834737	2.772886	+	1	14.38505	11.61217
O43633	CHMP2A	Charged m	25.104	222	6.829874	2.771859	+	1	13.47117	10.69931
P19388	POLR2E	DNA-direct	24.551	210	6.819035	2.769568	+	1	13.99859	11.22902
Q14839	CHD4	Chromodoı	218.005	1912	6.810247	2.767707	+	1	12.28898	9.521274
O75909	CCNK	Cyclin-K O:	64.24	580	6.789423	2.763289	+	1	12.90927	10.14599
P63218	GNG5	Guanine nu	7.318	68	6.765893	2.75828	+	1	12.65262	9.894341
Q5HYI8	RABL3	Rab-like pr	26.423	236	6.748832	2.754638	+	1	12.17789	9.423249
Q13503	MED21	Mediator ot	15.564	144	6.744554	2.753723	+	1	13.29031	10.53658
P35613	BSG	Basigin OS	42.2	385	6.733356	2.751326	+	2	16.87448	14.12315
Q8NEY8	PPHLN1	Periphilin-1	52.737	458	6.732463	2.751134	+	1	14.12129	11.37016
Q96RF0	SNX18	Sorting nex	68.894	628	6.715219	2.747434	+	1	12.80371	10.05627
Q9NS69	TOMM22	Mitochondı	15.522	142	6.714873	2.74736	+	1	12.90757	10.16021
Q9BSC4	NOL10	Nucleolar p	80.302	688	6.7037	2.744958	+	1	13.21664	10.47168
P28340	POLD1	DNA polym	123.631	1107	6.702983	2.744803	+	1	11.8179	9.0731
Q8N684	CPSF7	Cleavage a	52.05	471	6.697009	2.743517	+	1	14.29455	11.55103
P52756	RBM5	RNA-bindir	92.154	815	6.691174	2.742259	+	1	13.12239	10.38013
Q16342	PDCD2	Programm	38.592	344	6.66045	2.73562	+	1	13.58355	10.84793
P41567	EIF1	Eukaryotic	12.732	113	6.659215	2.735352	+	1	13.58167	10.84632
Q93009	USP7	Ubiquitin ca	128.302	1102	6.638413	2.730838	+	1	14.01332	11.28248
P61106	RAB14	Ras-relatec	23.897	215	6.623111	2.727509	+	1	13.38923	10.66172
P49959	MRE11	Double-stra	80.593	708	6.616347	2.726035	+	1	13.78841	11.06238
Q13263	TRIM28	Transcriptic	88.55	835	6.615755	2.725906	+	2	16.67162	13.94572
Q12846	STX4	Syntaxin-4	34.18	297	6.604343	2.723415	+	1	13.3696	10.64618
Q13951	CBFB	Core-bindir	21.508	182	6.603249	2.723176	+	1	13.55878	10.8356
Q96GA3	LTV1	Protein LT\	54.855	475	6.596091	2.721611	+	1	14.35576	11.63415
Q15390	MTFR1	Mitochondı	37.001	333	6.567635	2.715374	+	1	13.78841	11.07304
P19387	POLR2C	DNA-direct	31.441	275	6.564104	2.714598	+	2	15.17329	12.45869
P35606	COPB2	Coatomer s	102.487	906	6.543936	2.710159	+	1	14.18642	11.47627
Q9BRX5	GINS3	DNA replica	24.535	216	6.535463	2.70829	+	1	11.73043	9.022138
O14745	SLC9A3R1	Na(+)/H(+)	38.868	358	6.499707	2.700375	+	1	13.00543	10.30506
P32322	PYCR1	Pyrroline-5	33.361	319	6.49305	2.698896	+	1	13.57258	10.87369
Q86UY5	FAM83A	Protein FAI	47.458	434	6.48214	2.69647	+	1	12.89357	10.1971
Q9NX08	COMMD8	COMM dor	21.09	183	6.475577	2.695009	+	1	12.75104	10.05603
Q9Y3C6	PPIL1	Peptidyl-pro	18.237	166	6.474337	2.694733	+	2	16.29406	13.59933
O75439	PMPCB	Mitochondı	54.366	489	6.471775	2.694162	+	1	12.00958	9.315422
O75436	VPS26A	Vacuolar p	38.17	327	6.451849	2.689713	+	1	13.05394	10.36423
Q8N884	CGAS	Cyclic GMI	58.814	522	6.444185	2.687998	+	1	13.7292	11.0412
Q15750	TAB1	TGF-beta-a	54.644	504	6.439408	2.686928	+	1	14.51404	11.82711
O95793	STAU1	Double-stra	63.182	577	6.422706	2.683181	+	1	12.92719	10.244
Q99471	PFDN5	Prefoldin su	17.328	154	6.416708	2.681833	+	1	13.43359	10.75175
Q9UFC0	LRWD1	Leucine-ric	70.861	647	6.411209	2.680596	+	1	12.97382	10.29323
Q9NYB0	TERF2IP	Telomeric r	44.26	399	6.408602	2.68001	+	1	12.27973	9.599717
Q9NWS0	PIH1D1	PIH1 doma	32.363	290	6.406798	2.679604	+	1	13.93599	11.25639
Q9NRH3	TUBG2	Tubulin gar	51.092	451	6.387623	2.675279	+	1	13.46735	10.79207
Q5UIP0	RIF1	Telomere-a	274.466	2472	6.383397	2.674324	+	1	13.03983	10.3655
Q9Y5X2	SNX8	Sorting nex	52.569	465	6.36821	2.670888	+	1	12.78649	10.11561
O75427	LRCH4	Leucine-ric	73.45	683	6.366718	2.67055	+	1	12.52809	9.857538
P23677	ITPKA	Inositol-tris	51.009	461	6.365023	2.670166	+	1	12.91012	10.23995
Q96B97	SH3KBP1	SH3 domai	73.126	665	6.362185	2.669522	+	1	13.33915	10.66963
Q96EY8	MMAB	Corrinoid a	27.388	250	6.353459	2.667542	+	1	12.21669	9.549143
Q9NR30	DDX21	Nucleolar F	87.344	783	6.349792	2.666709	+	2	17.07012	14.40341
Q9NQT4	EXOSC5	Exosome c	25.249	235	6.343653	2.665314	+	1	13.925	11.25968
Q02978	SLC25A11	Mitochondı	34.062	314	6.333848	2.663082	+	1	13.52148	10.8584
Q9H3U1	UNC45A	Protein unc	103.077	944	6.320221	2.659975	+	1	13.02273	10.36276
Q13564	NAE1	NEDD8-ac	60.246	534	6.315334	2.658859	+	1	12.1095	9.450645
Q71RC2	LARP4	La-related	80.596	724	6.308899	2.657388	+	2	15.53172	12.87433

P51149	RAB7A	Ras-relate(	23.49	207	6.292082	2.653538	+	1	13.34693	10.69339
P53814	SMTN	Smoothelir	99.059	917	6.291951	2.653507	+	2	16.47717	13.82367
P16615	ATP2A2	Sarcoplasn	114.757	1042	6.280675	2.65092	+	1	13.83111	11.18019
P49790	NUP153	Nuclear po	153.938	1475	6.269511	2.648353	+	2	16.87867	14.23032
Q27J81	INF2	Inverted foi	135.624	1249	6.260082	2.646182	+	1	12.94171	10.29553
Q9H7E9	C8orf33	UPF0488 p	24.993	229	6.255738	2.64518	+	1	13.07424	10.42906
Q96M96	FGD4	FYVE, Rhc	86.626	766	6.235621	2.640533	+	1	13.30606	10.66553
O00264	PGRMC1	Membrane	21.671	195	6.235568	2.640521	+	1	15.23025	12.58972
Q99459	CDC5L	Cell divisio	92.251	802	6.23255	2.639823	+	2	15.26836	12.62854
O15269	SPTLC1	Serine palr	52.744	473	6.229574	2.639134	+	1	12.28306	9.643926
Q9UBQ0	VPS29	Vacuolar pi	20.506	182	6.223648	2.63776	+	1	13.30521	10.66745
O00567	NOP56	Nucleolar p	66.05	594	6.220255	2.636974	+	1	12.34155	9.704573
O43707	ACTN4	Alpha-actir	104.854	911	6.205704	2.633595	+	2	19.23928	16.60568
Q8NFX7	STXBP6	Syntaxin-bi	23.554	210	6.201867	2.632703	+	1	13.31274	10.68004
O76031	CLPX	ATP-deper	69.224	633	6.198166	2.631841	+	1	13.55459	10.92275
Q8IY21	DDX60	Probable A	197.853	1712	6.196785	2.63152	+	1	12.7266	10.09508
Q9UNX4	WDR3	WD repeat	106.099	943	6.195646	2.631255	+	1	12.49783	9.866572
O94906	PRPF6	Pre-mRNA	106.925	941	6.17451	2.626325	+	1	12.71167	10.08534
Q9H9Y6	POLR1B	DNA-direct	128.229	1135	6.161591	2.623303	+	1	12.36178	9.738476
Q9H0E2	TOLLIP	Toll-interac	30.282	274	6.156803	2.622181	+	1	13.42771	10.80552
Q13761	RUNX3	Runt-relate	44.356	415	6.128718	2.615585	+	1	12.44053	9.824946
Q9UNN8	PROCR	Endothelial	26.671	238	6.110867	2.611377	+	2	18.01269	15.40131
O60499	STX10	Syntaxin-1(	28.114	249	6.108955	2.610926	+	1	13.68562	11.0747
Q9H773	DCTPP1	dCTP pyro|	18.681	170	6.106632	2.610377	+	1	12.72998	10.1196
Q5VZK9	CARMIL1	F-actin-unc	151.557	1371	6.101904	2.60926	+	1	12.39626	9.786997
A6ZKI3	RTL8C	Retrotrans|	13.171	113	6.098273	2.608401	+	1	13.18843	10.58003
Q14241	ELOA	Elongin-A (	89.909	798	6.089654	2.60636	+	1	13.48003	10.87367
A1L170	C1orf226	Uncharacte	29.057	272	6.08537	2.605345	+	1	13.75457	11.14923
Q96L92	SNX27	Sorting ne›	61.265	541	6.082379	2.604636	+	1	12.69647	10.09183
Q5SW79	CEP170	Centrosom	175.293	1584	6.07849	2.603713	+	1	13.98469	11.38097
P10321	HLA-C	HLA class	40.649	366	6.06957	2.601594	+	2	16.00541	13.40381
Q96H35	RBM18	Probable R	21.649	190	6.060605	2.599462	+	1	14.22822	11.62876
O60231	DHX16	Pre-mRNA	119.264	1041	6.046782	2.596167	+	1	14.22408	11.62791
Q9BZM4	ULBP3	UL16-bindi	27.949	244	6.041037	2.594796	+	2	15.63052	13.03573
P12270	TPR	Nucleoprot	267.293	2363	6.038177	2.594113	+	2	15.8629	13.26879
P13489	RNH1	Ribonuclea	49.973	461	6.037791	2.594021	+	2	14.01472	11.4207
Q01780	EXOSC10	Exosome c	100.831	885	6.026425	2.591302	+	1	12.79853	10.20723
Q8TAP8	PPP1R35	Protein phc	27.953	253	6.026256	2.591262	+	1	13.38424	10.79298
P62745	RHOB	Rho-relate(	22.123	196	6.007808	2.586839	+	2	16.48251	13.89567
P25208	NFYB	Nuclear tra	22.831	207	6.002092	2.585465	+	1	13.82645	11.24098
Q9NQP4	PFDN4	Prefoldin su	15.314	134	5.99365	2.583435	+	1	13.22673	10.64329
Q9BPZ3	PAIP2	Polyadenyl	14.984	127	5.989929	2.582539	+	1	13.74725	11.16471
P36776	LONP1	Lon protea:	106.489	959	5.984274	2.581176	+	1	12.9691	10.38792
Q96G23	CERS2	Ceramide s	44.876	380	5.983828	2.581069	+	1	12.23643	9.655364
Q4KMQ1	TPRN	Taperin OS	75.556	711	5.972823	2.578413	+	2	17.15926	14.58085
P35251	RFC1	Replication	128.255	1148	5.969431	2.577593	+	1	13.13955	10.56196
Q8IZV5	RDH10	Retinol deh	38.087	341	5.958666	2.57499	+	1	12.28326	9.708273
Q92828	CORO2A	Coronin-2A	59.763	525	5.958401	2.574925	+	2	15.11411	12.53918
P00846	MT-ATP6	ATP syntha	24.817	226	5.957862	2.574795	+	1	12.4187	9.843901
P23193	TCEA1	Transcriptio	33.97	301	5.950442	2.572997	+	1	13.85389	11.2809
Q8WXW3	PIBF1	Progestero	89.805	757	5.936832	2.569693	+	1	12.5832	10.01351
Q86TC9	MYPN	Myopalladi	145.257	1320	5.936795	2.569684	+	1	14.04192	11.47223
Q68CQ7	GLT8D1	Glycosyltra	41.935	371	5.9365	2.569613	+	1	12.37737	9.807761
Q07065	CKAP4	Cytoskeleto	66.022	602	5.925413	2.566916	+	2	15.58611	13.01919
P62316	SNRPD2	Small nucle	13.527	118	5.918547	2.565243	+	2	15.39647	12.83123
Q9NYJ8	TAB2	TGF-beta-a	76.494	693	5.910737	2.563338	+	1	13.57341	11.01007
Q92598	HSPH1	Heat shock	96.865	858	5.896126	2.559767	+	2	14.92699	12.36722
Q9NX05	FAM120C	Constitutive	120.588	1096	5.895002	2.559492	+	1	11.68541	9.125913
O15230	LAMA5	Laminin su	399.737	3695	5.891433	2.558619	+	1	11.38084	8.822221

O60675	MAFK	Transcripti	17.523	156	5.862632	2.551549	+	1	13.11182	10.56027
O14773	TPP1	Tripeptidyl-	61.248	563	5.859666	2.550818	+	1	11.70956	9.158739
P20020	ATP2B1	Plasma me	134.685	1220	5.854217	2.549476	+	1	13.84274	11.29327
Q9BT25	HAUS8	HAUS augi	44.857	410	5.853357	2.549264	+	1	13.70325	11.15399
Q8IV48	ERI1	3'-5' exorib	40.064	349	5.851584	2.548827	+	1	13.67739	11.12856
Q9BVJ7	DUSP23	Dual specit	16.588	150	5.844867	2.54717	+	1	13.49411	10.94694
Q9P021	CRIPT	Cysteine-ri	11.216	101	5.842766	2.546651	+	1	13.40952	10.86287
Q9NYB9	ABI2	Abl interact	55.663	513	5.833493	2.54436	+	1	13.29978	10.75542
Q9UDW1	UQCR10	Cytochrom	7.308	63	5.826377	2.542599	+	1	12.87115	10.32856
P41250	GARS1	Glycine--tR	83.166	739	5.817413	2.540378	+	2	14.15963	11.61926
P06748	NPM1	Nucleopho	32.575	294	5.809537	2.538423	+	2	19.64723	17.10881
Q99622	C12orf57	Protein C1	13.178	126	5.806239	2.537604	+	1	14.03351	11.49591
Q5T280	SPOUT1	Putative m	42.009	376	5.805307	2.537372	+	1	13.4991	10.96173
Q9BT09	CNPY3	Protein car	30.748	278	5.782565	2.53171	+	1	12.92743	10.39572
Q9NNW5	WDR6	WD repeat	121.725	1121	5.771437	2.52893	+	1	12.57637	10.04744
Q8WUF5	PPP1R13L	RelA-asso	89.091	828	5.766715	2.52775	+	1	14.0897	11.56195
Q96I24	FUBP3	Far upstrea	61.64	572	5.762676	2.526739	+	1	14.07205	11.54531
Q9BRQ0	PYGO2	Pygopus h	41.244	406	5.754068	2.524582	+	1	12.29872	9.774139
Q16576	RBBP7	Histone-bir	47.82	425	5.753565	2.524456	+	2	17.73562	15.21117
P54289	CACNA2D	Voltage-de	124.568	1103	5.752745	2.524251	+	2	17.30919	14.78494
Q14566	MCM6	DNA replic	92.889	821	5.74236	2.521644	+	2	14.84475	12.32311
O95996	APC2	Adenomat	243.949	2303	5.741912	2.521531	+	1	14.72083	12.1993
Q5M775	SPECC1	Cytospin-B	118.585	1068	5.733395	2.51939	+	2	17.29813	14.77874
Q9BRK4	LZTS2	Leucine zip	72.759	669	5.718723	2.515693	+	1	12.63866	10.12297
P63096	GNAI1	Guanine nu	40.361	354	5.715811	2.514958	+	2	17.81209	15.29713
Q9P032	NDUFAF4	NADH deh	20.266	175	5.708934	2.513221	+	1	13.67937	11.16615
Q9BZG1	RAB34	Ras-relate	29.044	259	5.705745	2.512415	+	1	12.53072	10.01831
P51808	DYNLT3	Dynein ligh	13.062	116	5.705708	2.512406	+	1	13.00538	10.49297
Q96HR3	MED30	Mediator o	20.277	178	5.702547	2.511607	+	1	12.11087	9.599268
P31040	SDHA	Succinate	72.692	664	5.691069	2.5087	+	2	15.16624	12.65754
Q14493	SLBP	Histone RN	31.286	270	5.690211	2.508482	+	1	13.43098	10.92249
O75391	SPAG7	Sperm-ass	26.034	227	5.689064	2.508191	+	1	11.30754	8.799351
Q9GZT9	EGLN1	Egl nine ho	46.021	426	5.684341	2.506993	+	1	11.97882	9.471824
Q6UUV7	CRTC3	CREB-regu	66.959	619	5.683121	2.506683	+	1	13.07942	10.57273
Q5SRD1	TIMM23B	Mitochondr	19.67	188	5.677602	2.505282	+	2	14.42954	11.92426
Q5F1R6	DNAJC21	DnaJ homo	62.028	531	5.670106	2.503376	+	1	14.03531	11.53194
P29558	RBMS1	RNA-bindir	44.505	406	5.664434	2.501932	+	1	13.92147	11.41954
Q8N7H5	PAF1	RNA polym	59.976	531	5.653643	2.499181	+	1	12.54185	10.04267
Q14681	KCTD2	BTB/POZ d	28.527	263	5.641221	2.496008	+	1	12.181	9.684989
Q9Y2D8	SSX2IP	Afadin- and	71.236	614	5.6283	2.492699	+	1	12.52356	10.03086
Q9H6S0	YTHDC2	3'-5' RNA h	160.248	1430	5.625962	2.4921	+	1	12.60784	10.11574
Q96CS3	FAF2	FAS-assoc	52.623	445	5.621437	2.490939	+	1	12.91126	10.42032
Q8IZL8	PELP1	Proline-, gl	119.7	1130	5.616069	2.489561	+	1	13.18697	10.6974
P62310	LSM3	U6 snRNA-	11.845	102	5.615843	2.489502	+	1	13.71188	11.22238
P51970	NDUFA8	NADH deh	20.105	172	5.613617	2.488931	+	1	13.65027	11.16134
O43813	LANCL1	Glutathione	45.283	399	5.610616	2.488159	+	1	13.65609	11.16793
P04844	RPN2	Dolichyl-dip	69.284	631	5.606504	2.487101	+	1	13.60374	11.11664
Q9Y6R0	NUMBL	Numb-like	64.891	609	5.606089	2.486995	+	1	15.14414	12.65715
Q01433	AMPD2	AMP deam	100.688	879	5.597839	2.48487	+	1	10.853	8.368128
Q14651	PLS1	Plastin-1 O	70.253	629	5.584446	2.481414	+	2	16.25486	13.77345
Q8WUB8	PHF10	PHD finger	56.051	498	5.57593	2.479212	+	1	12.6055	10.12629
Q9Y692	GMEB1	Glucocortic	62.591	573	5.569645	2.477585	+	1	11.80723	9.329649
P12814	ACTN1	Alpha-actin	103.058	892	5.55583	2.474002	+	2	20.36335	17.88935
Q15424	SAFB	Scaffold at	102.642	915	5.550301	2.472566	+	2	15.19225	12.71969
Q9P000	COMMD9	COMM dor	21.819	198	5.548267	2.472037	+	1	13.22598	10.75394
Q8N954	GPATCH1	G patch do	33.277	285	5.543488	2.470794	+	1	13.00056	10.52977
Q9UER7	DAXX	Death dom	81.373	740	5.524383	2.465813	+	1	13.62776	11.16195
Q8N163	CCAR2	Cell cycle a	102.902	923	5.516584	2.463775	+	2	14.10844	11.64467
P53701	HCCS	Holocytoch	30.602	268	5.501552	2.459839	+	1	13.42141	10.96157

P06241	FYN	Tyrosine-pı	60.762	537	5.500284	2.459506	+	1	12.63422	10.17471
Q5HYK7	SH3D19	SH3 domai	86.525	790	5.483597	2.455122	+	1	12.94427	10.48915
Q96PY5	FMNL2	Formin-like	123.321	1086	5.475414	2.452968	+	2	18.10621	15.65324
A5YKK6	CNOT1	CCR4-NOT	266.939	2376	5.470276	2.451614	+	1	12.15924	9.707627
O00178	GTPBP1	GTP-bindir	72.454	669	5.465879	2.450453	+	1	13.44863	10.99818
Q9NQT8	KIF13B	Kinesin-like	202.789	1826	5.448613	2.445889	+	1	12.53821	10.09232
Q15418	RPS6KA1	Ribosomal	82.723	735	5.443866	2.444632	+	1	13.77438	11.32974
O75534	CSDE1	Cold shock	88.885	798	5.430528	2.441092	+	2	14.86026	12.41917
P28070	PSMB4	Proteasom	29.204	264	5.429336	2.440776	+	1	13.6185	11.17772
P08243	ASNS	Asparagine	64.37	561	5.427459	2.440277	+	1	13.8896	11.44932
Q5VWG9	TAF3	Transcripti	103.582	929	5.418914	2.438004	+	1	13.39727	10.95927
P40616	ARL1	ADP-ribosy	20.418	181	5.407093	2.434853	+	1	12.2035	9.768648
P50213	IDH3A	Isocitrate d	39.592	366	5.406026	2.434569	+	1	13.09644	10.66187
Q9P0V9	SEPTIN10	Septin-10 (	52.593	454	5.393847	2.431315	+	1	13.30021	10.86889
Q9BTC0	DIDO1	Death-indu	243.873	2240	5.393241	2.431153	+	1	11.70066	9.269504
P33992	MCM5	DNA replic	82.286	734	5.387915	2.429727	+	2	16.10226	13.67254
O43159	RRP8	Ribosomal	50.715	456	5.380608	2.427769	+	1	11.67834	9.250567
Q99584	S100A13	Protein S1(	11.471	98	5.362508	2.422908	+	1	12.96127	10.53836
Q9NW82	WDR70	WD repeat	73.201	654	5.358338	2.421785	+	1	13.12783	10.70605
Q9BWG6	SCNM1	Sodium cha	25.949	230	5.358176	2.421742	+	1	13.08408	10.66234
Q9NSC5	HOMER3	Homer prot	39.836	361	5.353507	2.420484	+	2	18.21091	15.79042
Q9NP81	SARS2	Serine--tRI	58.283	518	5.351573	2.419963	+	1	12.71583	10.29587
O14639	ABLIM1	Actin-bindi	87.688	778	5.350664	2.419718	+	2	17.14679	14.72707
P78380	OLR1	Oxidized lo	30.959	273	5.344788	2.418133	+	1	12.17143	9.753294
P78344	EIF4G2	Eukaryotic	102.362	907	5.338114	2.41633	+	1	13.28171	10.86538
Q8N4C6	NIN	Ninein OS=	243.249	2090	5.337751	2.416232	+	2	18.26847	15.85224
Q9UPZ3	HPS5	Hermansky	127.449	1129	5.334	2.415218	+	1	13.34568	10.93046
P40261	NNMT	Nicotinami	29.574	264	5.32971	2.414057	+	1	12.15238	9.738323
O14617	AP3D1	AP-3 comp	130.158	1153	5.329666	2.414045	+	1	11.88142	9.467375
Q9Y2L1	DIS3	Exosome c	109.003	958	5.329604	2.414028	+	1	12.85349	10.43946
Q9H2U1	DHX36	ATP-deper	114.76	1008	5.32224	2.412033	+	1	14.29182	11.87978
P53396	ACLY	ATP-citrate	120.839	1101	5.319879	2.411393	+	2	13.70325	11.29186
P07858	CTSB	Cathepsin	37.822	339	5.306003	2.407625	+	1	12.40205	9.994427
Q6P1X5	TAF2	Transcripti	136.971	1199	5.272029	2.398358	+	1	11.90828	9.509922
P11047	LAMC1	Laminin su	177.603	1609	5.271482	2.398209	+	1	11.98068	9.582467
O94776	MTA2	Metastasis	75.023	668	5.261689	2.395526	+	1	13.21879	10.82326
Q8IXT5	RBM12B	RNA-bindir	118.103	1001	5.259151	2.39483	+	1	12.54219	10.14736
O75131	CPNE3	Copine-3 C	60.131	537	5.254611	2.393584	+	1	13.1027	10.70911
Q9UNM6	PSMD13	26S protea	42.945	376	5.246153	2.39126	+	1	13.65195	11.26069
P17275	JUNB	Transcripti	35.879	347	5.232528	2.387508	+	1	13.10326	10.71575
Q9UIL1	SCOC	Short coile	18.045	159	5.227546	2.386134	+	1	13.135	10.74887
Q6PKG0	LARP1	La-related	123.51	1096	5.211442	2.381683	+	2	16.68168	14.29999
Q99661	KIF2C	Kinesin-like	81.313	725	5.198888	2.378203	+	1	13.5626	11.1844
Q12860	CNTN1	Contactin-1	113.32	1018	5.184129	2.374102	+	2	17.52848	15.15438
Q04323	UBXN1	UBX doma	33.325	297	5.172881	2.370968	+	2	15.01946	12.64849
Q15637	SF1	Splicing fac	68.33	639	5.171745	2.370651	+	2	16.40416	14.03351
O43172	PRPF4	U4/U6 sma	58.449	522	5.164439	2.368612	+	1	14.3526	11.98398
P49848	TAF6	Transcripti	72.668	677	5.158599	2.366979	+	1	13.82416	11.45718
Q969T9	WBP2	WW doma	28.087	261	5.154081	2.365715	+	1	12.9208	10.55508
P04843	RPN1	Dolichyl-dip	68.569	607	5.144782	2.36311	+	2	14.92017	12.55706
Q9Y5B6	PAXBP1	PAX3- and	104.804	917	5.142983	2.362605	+	1	13.0989	10.7363
Q9UL15	BAG5	BAG family	51.2	447	5.137526	2.361074	+	1	13.72142	11.36035
Q9Y3A5	SBDS	Ribosome	28.764	250	5.119363	2.355964	+	1	12.11832	9.76236
Q8NHQ9	DDX55	ATP-deper	68.547	600	5.102544	2.351217	+	1	12.27871	9.927494
Q9NV31	IMP3	U3 small nı	21.85	184	5.097377	2.349755	+	1	12.19927	9.849519
P60510	PPP4C	Serine/thre	35.08	307	5.097351	2.349748	+	1	12.37854	10.02879
Q9NUP9	LIN7C	Protein lin-7	21.834	197	5.095473	2.349216	+	2	16.37098	14.02176
Q9BZX2	UCK2	Uridine-cyti	29.299	261	5.084747	2.346176	+	1	13.83635	11.49017
Q13889	GTF2H3	General tra	34.378	308	5.078366	2.344364	+	1	13.01566	10.67129

Q5JWF2	GNAS	Guanine nu	111.025	1037	5.060956	2.33941	+	2	17.51761	15.1782
Q9Y5S2	CDC42BPI	Serine/thre	194.315	1711	5.059377	2.33896	+	1	12.86838	10.52942
O95456	PSMG1	Proteasom	32.854	288	5.059319	2.338943	+	2	14.14561	11.80667
Q9Y450	HBS1L	HBS1-like	75.473	684	5.057146	2.338324	+	1	12.55349	10.21516
P18031	PTPN1	Tyrosine-pr	49.967	435	5.051971	2.336846	+	1	12.01137	9.674521
Q9Y3F4	STRAP	Serine-thre	38.438	350	5.04499	2.334851	+	2	16.37326	14.0384
P27816	MAP4	Microtubule	121.005	1152	5.038929	2.333117	+	2	15.63818	13.30506
P49005	POLD2	DNA polym	51.289	469	5.031975	2.331125	+	1	12.95225	10.62113
Q9BSV6	TSEN34	tRNA-splici	33.652	310	5.019358	2.327503	+	1	12.60703	10.27953
P28838	LAP3	Cytosol am	56.166	519	5.012973	2.325666	+	1	11.41611	9.090445
Q9BZH6	WDR11	WD repeat	136.685	1224	5.008332	2.32433	+	1	12.91948	10.59515
Q9BRQ6	CHCHD6	MICOS cor	26.458	235	5.003537	2.322948	+	1	12.7946	10.47165
Q9NR33	POLE4	DNA polym	12.209	117	5.001493	2.322359	+	1	12.00282	9.680456
Q8WUZ0	BCL7C	B-cell CLL/	23.468	217	4.996975	2.321055	+	1	14.48363	12.16257
Q9Y2Q5	LAMTOR2	Ragulator c	13.508	125	4.995946	2.320758	+	2	15.26704	12.94628
Q53GS9	USP39	U4/U6.U5 t	65.381	565	4.985247	2.317665	+	1	11.89773	9.580067
Q9BQ61	TRIR	Telomeras	18.419	176	4.984506	2.317451	+	1	13.88732	11.56987
Q96E29	MTERF3	Transcripti	47.971	417	4.979165	2.315904	+	1	13.11091	10.795
O95470	SGPL1	Sphingosin	63.524	568	4.972388	2.313939	+	1	13.43032	11.11638
Q9H479	FN3K	Fructosami	35.171	309	4.970368	2.313353	+	2	14.49336	12.18
Q9ULD2	MTUS1	Microtubule	141.397	1270	4.959862	2.3103	+	1	13.53296	11.22266
O15067	PFAS	Phosphorib	144.734	1338	4.954409	2.308713	+	1	13.09782	10.78911
P05026	ATP1B1	Sodium/po	35.061	303	4.95305	2.308317	+	1	13.96596	11.65765
Q14642	INPP5A	Inositol pol	47.82	412	4.95062	2.307609	+	1	13.68759	11.37999
P20810	CAST	Calpastatin	76.573	708	4.945269	2.306049	+	1	12.97093	10.66488
O75525	KHDRBS3	KH domain	38.8	346	4.943118	2.305421	+	1	14.13162	11.82619
P56270	MAZ	Myc-assoc	48.608	477	4.938952	2.304205	+	1	11.45327	9.149066
P13639	EEF2	Elongation	95.338	858	4.934393	2.302873	+	2	17.39716	15.09428
Q96PE2	ARHGEF1	Rho guanir	221.673	2063	4.92598	2.300411	+	1	13.09579	10.79538
Q9BRA0	NAA38	N-alpha-ac	13.514	125	4.918279	2.298154	+	1	12.21493	9.916772
Q8WV92	MITD1	MIT domai	29.314	249	4.915504	2.297339	+	1	12.43538	10.13804
Q9H6D7	HAUS4	HAUS augi	42.4	363	4.91182	2.296258	+	1	11.7868	9.490543
O95684	CEP43	Centrosom	43.065	399	4.892158	2.290471	+	1	12.74155	10.45108
P40938	RFC3	Replication	40.556	356	4.888953	2.289526	+	1	13.33385	11.04433
Q12899	TRIM26	Tripartite m	62.166	539	4.882521	2.287626	+	1	13.75572	11.4681
Q8N3X1	FNBP4	Formin-bin	110.266	1017	4.879945	2.286865	+	1	13.01365	10.72679
Q96P70	IPO9	Importin-9	115.963	1041	4.866613	2.282918	+	1	13.61344	11.33053
Q9NP92	MRPS30	39S riboso	50.365	439	4.858628	2.280549	+	1	11.81678	9.536235
P11413	G6PD	Glucose-6-	59.257	515	4.837703	2.274322	+	2	14.3312	12.05687
Q00535	CDK5	Cyclin-depe	33.304	292	4.837367	2.274222	+	1	13.32305	11.04883
Q9H246	C1orf21	Uncharacte	13.865	121	4.833959	2.273205	+	1	12.87048	10.59728
Q9UKD2	MRTO4	mRNA turn	27.56	239	4.832377	2.272733	+	1	13.47485	11.20211
Q9BW92	TARS2	Threonine-	81.036	718	4.824675	2.270432	+	1	12.2159	9.945465
Q9NVS9	PNPO	Pyridoxine-	29.988	261	4.822233	2.269701	+	1	12.91728	10.64758
Q9H2D6	TRIOBP	TRIO and F	261.376	2365	4.817375	2.268247	+	1	14.22393	11.95568
Q8N5C8	TAB3	TGF-beta-a	78.653	712	4.815821	2.267782	+	1	12.82532	10.55753
P68400	CSNK2A1	Casein kina	45.144	391	4.800637	2.263226	+	2	14.8359	12.57268
P49916	LIG3	DNA ligase	112.907	1009	4.798941	2.262716	+	2	13.54569	11.28297
Q9BUJ2	HNRNPUL	Heterogene	95.739	856	4.79812	2.262469	+	2	16.16569	13.90322
Q13601	KRR1	KRR1 sma	43.665	381	4.798043	2.262446	+	1	13.57317	11.31073
P52948	NUP98	Nuclear po	197.579	1817	4.793078	2.260952	+	1	13.71457	11.45362
Q9NYD6	HOXC10	Homeobox	38.073	342	4.785789	2.258757	+	1	12.69905	10.4403
P53350	PLK1	Serine/thre	68.255	603	4.77583	2.255751	+	1	13.12641	10.87066
Q03111	MLLT1	Protein EN	62.056	559	4.76935	2.253793	+	1	12.84257	10.58877
Q9Y4E1	WASHC2C	WASH con	144.911	1320	4.768549	2.25355	+	1	11.72426	9.470708
P35222	CTNNB1	Catenin be	85.497	781	4.768289	2.253472	+	1	13.80796	11.55449
Q9NWT1	PAK1IP1	p21-activat	43.964	392	4.757508	2.250206	+	1	12.73568	10.48548
Q29983	MICA	MHC class	42.915	383	4.755784	2.249683	+	1	14.82347	12.57378
P11233	RALA	Ras-related	23.567	206	4.745442	2.246542	+	2	18.07379	15.82724

Q96A26	FAM162A	Protein FAI	17.342	154	4.744556	2.246273	+	1	12.43564	10.18937
Q8N5A5	ZGPAT	Zinc finger	57.359	531	4.744059	2.246122	+	1	11.11348	8.86736
Q9NZT2	OGFR	Opioid grov	73.325	677	4.743341	2.245904	+	1	11.92615	9.680244
Q2TAY7	SMU1	WD40 repe	57.544	513	4.740083	2.244912	+	2	15.28879	13.04388
P61457	PCBD1	Pterin-4-alp	12	104	4.739875	2.244849	+	1	12.71873	10.47388
Q8TED9	AFAP1L1	Actin filame	86.432	768	4.727334	2.241027	+	1	12.22255	9.981527
P50148	GNAQ	Guanine nu	42.142	359	4.723677	2.23991	+	1	13.87872	11.63881
P40227	CCT6A	T-complex	58.024	531	4.715197	2.237318	+	2	16.38746	14.15014
Q5JSZ5	PRRC2B	Protein PR	242.967	2229	4.714979	2.237251	+	1	13.88941	11.65216
Q9H9A5	CNOT10	CCR4-NOT	82.31	744	4.712405	2.236463	+	1	12.86248	10.62602
P62877	RBX1	E3 ubiquitir	12.274	108	4.710306	2.235821	+	1	13.36153	11.12571
Q9H7D7	WDR26	WD repeat	72.124	661	4.708217	2.235181	+	1	12.67556	10.44038
Q15646	OASL	2'-5'-oligoa	59.226	514	4.707048	2.234823	+	2	15.95774	13.72291
Q9H814	PHAX	Phosphoryl	44.403	394	4.705438	2.234329	+	2	14.77371	12.53938
Q96J84	KIRREL1	Kin of IRRE	83.536	757	4.701877	2.233237	+	1	13.37558	11.14235
Q96CT7	CCDC124	Coiled-coil	25.835	223	4.692305	2.230297	+	1	13.07307	10.84277
P41743	PRKCI	Protein kina	68.262	596	4.674122	2.224695	+	1	11.78668	9.561983
Q16649	NFIL3	Nuclear fac	51.472	462	4.662753	2.221182	+	1	13.2229	11.00172
P60604	UBE2G2	Ubiquitin-co	18.566	165	4.661552	2.22081	+	1	13.87815	11.65734
Q9BXL6	CARD14	Caspase re	113.27	1004	4.660932	2.220618	+	1	13.16743	10.94682
P35241	RDX	Radixin OS	68.564	583	4.656189	2.219149	+	1	13.96434	11.74519
Q96GK7	FAHD2A	Fumarylace	34.596	314	4.655682	2.218993	+	1	12.764	10.54501
P09471	GNAO1	Guanine nu	40.051	354	4.649559	2.217094	+	1	14.1956	11.97851
Q92896	GLG1	Golgi appa	134.552	1179	4.648747	2.216842	+	2	14.71032	12.49348
Q9Y490	TLN1	Talin-1 OS	269.767	2541	4.644628	2.215563	+	2	16.88262	14.66706
Q9Y6M0	PRSS21	Testisin OS	34.884	314	4.638825	2.21376	+	1	12.43199	10.21823
Q9Y3E5	PTRH2	Peptidyl-tR	19.194	179	4.625989	2.209762	+	1	12.18741	9.977652
Q9Y4E8	USP15	Ubiquitin ca	112.419	981	4.623595	2.209015	+	1	13.24384	11.03483
P68032	ACTC1	Actin, alpha	42.019	377	4.615594	2.206516	+	2	16.18666	13.98014
Q9H0C8	ILKAP	Integrin-link	42.907	392	4.60359	2.202759	+	1	12.85028	10.64753
P14923	JUP	Junction pla	81.745	745	4.602311	2.202359	+	2	18.61633	16.41397
Q9BTC8	MTA3	Metastasis	67.504	594	4.597542	2.200863	+	1	12.68299	10.48213
P06730	EIF4E	Eukaryotic	25.097	217	4.597002	2.200693	+	2	15.67833	13.47763
Q14574	DSC3	Desmocolli	99.969	896	4.586252	2.197315	+	1	12.00401	9.806694
Q9ULR0	ISY1	Pre-mRNA	32.992	285	4.58024	2.195423	+	1	13.3333	11.13787
Q15369	ELOC	Elongin-C (	12.473	112	4.574102	2.193488	+	2	16.16216	13.96867
Q9NW64	RBM22	Pre-mRNA	46.896	420	4.563545	2.190155	+	1	13.1801	10.98994
O95400	CD2BP2	CD2 antige	37.646	341	4.563519	2.190147	+	1	13.42587	11.23572
Q96GQ7	DDX27	Probable A	89.835	796	4.559998	2.189033	+	1	13.1444	10.95537
P36405	ARL3	ADP-ribosy	20.456	182	4.55833	2.188505	+	1	13.05408	10.86557
O14776	TCERG1	Transcripti	123.901	1098	4.557964	2.18839	+	2	14.20457	12.01618
P62195	PSMC5	26S protea	45.626	406	4.554806	2.18739	+	2	14.54533	12.35794
P09923	ALPI	Intestinal-ty	56.812	528	4.549026	2.185558	+	2	13.9427	11.75714
O75116	ROCK2	Rho-assoc	160.9	1388	4.543038	2.183657	+	1	11.69949	9.515828
Q08AG7	MZT1	Mitotic-spir	8.479	82	4.540155	2.182742	+	1	12.4996	10.31686
Q9NW61	PLEKHJ1	Pleckstrin h	17.551	149	4.513568	2.174268	+	1	12.02174	9.847475
Q99848	EBNA1BP2	Probable rR	34.852	306	4.509762	2.173051	+	2	16.5451	14.37205
Q96D46	NMD3	60S riboso	57.603	503	4.506412	2.171979	+	1	11.84478	9.672805
Q9H0J9	PARP12	Protein mo	79.064	701	4.501794	2.1705	+	1	12.94011	10.76961
Q15746	MYLK	Myosin ligh	210.715	1914	4.497157	2.169013	+	1	14.15893	11.98991
O60264	SMARCA5	SWI/SNF-r	121.905	1052	4.495562	2.168501	+	1	12.82126	10.65275
Q9UPY8	MAPRE3	Microtubule	31.982	281	4.492924	2.167655	+	1	13.22373	11.05608
Q14318	FKBP8	Peptidyl-pro	44.562	412	4.478267	2.16294	+	1	12.62205	10.45911
Q13111	CHAF1A	Chromatin	106.91	956	4.477823	2.162797	+	1	13.57566	11.41286
P50150	GNG4	Guanine nu	8.389	75	4.472226	2.160993	+	1	13.34305	11.18205
Q9Y6E2	BZW2	eIF5-mimic	48.162	419	4.471649	2.160807	+	1	12.64678	10.48598
O95777	LSM8	U6 snRNA-	10.403	96	4.464888	2.158624	+	1	12.78326	10.62464
Q9BWJ5	SF3B5	Splicing fac	10.135	86	4.451716	2.154362	+	2	14.6425	12.48814
P78371	CCT2	T-complex	57.488	535	4.450938	2.154109	+	2	16.31713	14.16302

Q9BRX2	PELO	Protein pel	43.359	385	4.449606	2.153678	+	1	11.70118	9.547499
P21860	ERBB3	Receptor ty	148.098	1342	4.449534	2.153654	+	2	17.08379	14.93014
Q9H078	CLPB	Caseinolyti	78.729	707	4.431911	2.147929	+	1	13.1004	10.95247
Q9Y2Q9	MRPS28	28S riboso	20.843	187	4.431867	2.147915	+	2	14.95283	12.80492
P12931	SRC	Proto-onco	59.835	536	4.431716	2.147865	+	1	12.02701	9.879142
Q9NVJ2	ARL8B	ADP-ribosy	21.539	186	4.421905	2.144668	+	1	11.82114	9.676468
P62879	GNB2	Guanine nu	37.331	340	4.420467	2.144199	+	2	20.16521	18.02101
P15408	FOSL2	Fos-relatec	35.193	326	4.40888	2.140412	+	1	12.43858	10.29817
Q9UKL0	RCOR1	REST core	53.327	485	4.408013	2.140129	+	2	15.40218	13.26205
Q658Y4	FAM91A1	Protein FAI	93.909	838	4.404347	2.138928	+	1	12.88956	10.75063
Q9NP74	PALMD	Palmdelphi	62.758	551	4.399103	2.137209	+	1	12.74542	10.60821
O75781	PALM	Paralemmi	42.076	387	4.395925	2.136167	+	2	17.43973	15.30357
Q9P0V3	SH3BP4	SH3 domai	107.496	963	4.393319	2.135311	+	1	11.3403	9.204984
Q96EY1	DNAJA3	DnaJ homo	52.489	480	4.39066	2.134438	+	2	16.38148	14.24704
Q9BQI0	AIF1L	Allograft int	17.068	150	4.389638	2.134102	+	2	15.19979	13.06569
P78346	RPP30	Ribonuclea	29.321	268	4.386619	2.13311	+	1	12.69113	10.55802
O75306	NDUFS2	NADH deh	52.546	463	4.385874	2.132864	+	1	13.88684	11.75398
Q9UBK9	UXT	Protein UX	18.246	157	4.380676	2.131154	+	1	12.81702	10.68587
P50750	CDK9	Cyclin-depe	42.778	372	4.370768	2.127887	+	1	13.18789	11.06001
Q7Z3B4	NUP54	Nucleopori	55.435	507	4.366136	2.126357	+	1	12.25352	10.12717
P63010	AP2B1	AP-2 comp	104.553	937	4.361883	2.124951	+	2	19.55646	17.43151
Q7Z3J3	RGPD4	RanBP2-lik	197.289	1758	4.360309	2.12443	+	1	12.78204	10.65761
P46777	RPL5	60S riboso	34.363	297	4.352928	2.121986	+	2	15.80476	13.68278
P25788	PSMA3	Proteasom	28.433	255	4.349975	2.121007	+	1	13.68738	11.56637
Q9NR31	SAR1A	GTP-bindir	22.367	198	4.347006	2.120022	+	1	13.93369	11.81367
Q969X6	UTP4	U3 small nu	76.89	686	4.344014	2.119029	+	1	13.09956	10.98053
P84095	RHOG	Rho-relatec	21.309	191	4.343356	2.11881	+	1	14.39934	12.28053
P68402	PAFAH1B2	Platelet-act	25.569	229	4.342944	2.118673	+	1	12.47895	10.36027
P30740	SERPINB1	Leukocyte	42.742	379	4.340358	2.117814	+	2	12.64968	10.53187
O00170	AIP	AH recepto	37.664	330	4.335909	2.116334	+	1	13.23524	11.1189
Q9UK59	DBR1	Lariat debra	61.555	544	4.319782	2.110959	+	1	12.23131	10.12035
P28331	NDUFS1	NADH-ubic	79.468	727	4.315454	2.109512	+	1	12.09833	9.988816
Q9HCP0	CSNK1G1	Casein kina	48.511	422	4.31113	2.108066	+	1	13.24151	11.13344
P09874	PARP1	Poly [ADP-	113.084	1014	4.309283	2.107448	+	2	16.96567	14.85822
Q8NBQ5	HSD17B11	Estradiol 17	32.936	300	4.287552	2.100154	+	1	13.50333	11.40317
Q8TEQ6	GEMIN5	Gem-assoc	168.589	1508	4.274122	2.095628	+	1	13.26942	11.17379
Q86UP2	KTN1	Kinectin OS	156.275	1357	4.270176	2.094295	+	2	13.76663	11.67234
Q53LP3	SOWAHC	Ankyrin rep	55.672	525	4.26739	2.093354	+	1	13.32853	11.23518
Q9BX40	LSM14B	Protein LSI	42.071	385	4.261065	2.091214	+	1	11.45327	9.362056
O15371	EIF3D	Eukaryotic	63.973	548	4.256498	2.089667	+	2	14.85613	12.76647
P33991	MCM4	DNA replica	96.558	863	4.25506	2.089179	+	2	15.17376	13.08458
O00442	RTCA	RNA 3'-terr	39.337	366	4.253936	2.088798	+	1	12.41864	10.32984
Q00577	PURA	Transcriptio	34.911	322	4.25299	2.088477	+	2	15.82287	13.73439
Q9BXF6	RAB11FIP5	Rab11 fam	70.415	653	4.250117	2.087503	+	1	12.86293	10.77543
Q9UBS0	RPS6KB2	Ribosomal	53.455	482	4.248727	2.08703	+	1	12.51385	10.42682
Q96G25	MED8	Mediator of	29.08	268	4.232687	2.081574	+	1	12.70846	10.62688
O75694	NUP155	Nuclear po	155.199	1391	4.231464	2.081157	+	1	13.46148	11.38032
Q99829	CPNE1	Copine-1 C	59.059	537	4.23082	2.080937	+	1	12.1064	10.02546
Q15773	MLF2	Myeloid leu	28.147	248	4.222542	2.078112	+	1	13.85224	11.77413
O15049	N4BP3	NEDD4-bir	60.47	544	4.221349	2.077704	+	1	13.01573	10.93802
Q9P1F3	ABRACL	Costars far	9.057	81	4.214871	2.075489	+	1	11.77462	9.699134
Q9Y605	MRFAP1	MORF4 far	14.65	127	4.20991	2.073789	+	1	11.81478	9.740993
Q8NFJ5	GPRC5A	Retinoic ac	40.251	357	4.209056	2.073497	+	2	19.02524	16.95174
O43852	CALU	Calumenin	37.107	315	4.198929	2.070021	+	2	15.54499	13.47497
O75718	CRTAP	Cartilage-a	46.562	401	4.196356	2.069137	+	1	11.59353	9.524394
Q15154	PCM1	Pericentriol	228.544	2024	4.19166	2.067522	+	2	16.95174	14.88422
O60890	OPHN1	Oligophren	91.641	802	4.190224	2.067027	+	1	13.06444	10.99741
Q99501	GAS2L1	GAS2-like	72.717	681	4.183826	2.064823	+	1	12.715	10.65017
P46977	STT3A	Dolichyl-dip	80.53	705	4.181837	2.064137	+	1	11.87214	9.807999

Q9NRY2	INIP	SOSS com	11.425	104	4.179686	2.063395	+	1	12.03239	9.968996
Q9Y5S9	RBM8A	RNA-bindir	19.889	174	4.176971	2.062457	+	1	14.30585	12.24339
P51116	FXR2	Fragile X m	74.223	673	4.176721	2.062371	+	2	14.62325	12.56088
O14802	POLR3A	DNA-direct	155.641	1390	4.168226	2.059434	+	1	10.78676	8.727326
Q96CS2	HAUS1	HAUS augi	31.863	278	4.167257	2.059098	+	1	12.69729	10.6382
Q15393	SF3B3	Splicing fac	135.577	1217	4.164512	2.058147	+	2	16.7407	14.68256
O75431	MTX2	Metaxin-2 (	29.763	263	4.16073	2.056837	+	2	16.33347	14.27663
Q6NYC8	PPP1R18	Phostensin	67.943	613	4.159653	2.056463	+	2	19.39607	17.33961
P07947	YES1	Tyrosine-pi	60.801	543	4.159201	2.056306	+	2	18.08758	16.03127
Q68D10	SPTY2D1	Protein SP	75.599	685	4.158757	2.056152	+	1	13.38775	11.3316
P38117	ETFB	Electron tra	27.844	255	4.155994	2.055194	+	1	13.33274	11.27754
Q6DD88	ATL3	Atlastin-3 (	60.542	541	4.153251	2.054241	+	1	12.39296	10.33872
Q53SF7	COBLL1	Cordon-ble	123.868	1128	4.145505	2.051548	+	1	13.1433	11.09176
Q9Y265	RUVBL1	RuvB-like 1	50.228	456	4.144456	2.051183	+	2	16.59319	14.542
P51151	RAB9A	Ras-relatec	22.838	201	4.141976	2.050319	+	1	13.81348	11.76316
Q9HCE1	MOV10	Helicase M	113.671	1003	4.141616	2.050194	+	2	14.96975	12.91955
P82912	MRPS11	28S ribosol	20.616	194	4.139262	2.049373	+	1	12.18453	10.13516
O95466	FMNL1	Formin-like	121.854	1100	4.128308	2.045551	+	1	14.59053	12.54498
Q9HAU5	UPF2	Regulator c	147.81	1272	4.117029	2.041604	+	1	12.26512	10.22351
Q5T7V8	GORAB	RAB6-inter	42.266	369	4.113926	2.040516	+	1	11.72703	9.686511
Q9Y2G0	EFR3B	Protein EFI	92.487	817	4.113528	2.040376	+	1	11.57554	9.535163
Q9NP77	SSU72	RNA polym	22.574	194	4.111549	2.039682	+	1	12.80522	10.76554
P12081	HARS1	Histidine--t	57.411	509	4.110107	2.039176	+	1	12.70511	10.66594
Q9NXR7	BABAM2	BRISC anc	43.552	383	4.108091	2.038468	+	1	11.96676	9.92829
Q9BPX3	NCAPG	Condensin	114.334	1015	4.104713	2.037281	+	1	12.14236	10.10508
Q12904	AIMP1	Aminoacyl	34.353	312	4.100796	2.035904	+	2	16.05376	14.01785
Q15067	ACOX1	Peroxisom	74.424	660	4.10019	2.035691	+	1	11.89387	9.85818
Q13444	ADAM15	Disintegrin	92.959	863	4.098692	2.035164	+	1	11.35601	9.320848
Q96T88	UHRF1	E3 ubiquitir	89.814	793	4.091876	2.032762	+	1	13.96452	11.93176
Q03701	CEBPZ	CCAAT/en	120.974	1054	4.076661	2.027388	+	1	12.46888	10.44149
A6NDG6	PGP	Glycerol-3-	34.006	321	4.074225	2.026526	+	1	12.6439	10.61738
O94763	URI1	Unconventi	59.832	535	4.072411	2.025883	+	1	12.75046	10.72457
O60763	USO1	General ve	107.895	962	4.067217	2.024042	+	1	12.47477	10.45073
Q12959	DLG1	Disks large	100.455	904	4.055883	2.020016	+	1	12.85621	10.8362
Q9UFG5	C19orf25	UPF0449 p	12.878	118	4.05522	2.01978	+	1	11.37813	9.358352
Q16513	PKN2	Serine/thre	112.035	984	4.052779	2.018912	+	1	12.13028	10.11137
Q9UNF1	MAGED2	Melanoma-	64.954	606	4.052433	2.018788	+	1	12.1326	10.11381
Q8WV41	SNX33	Sorting nex	65.265	574	4.04776	2.017124	+	1	12.15494	10.13782
Q9NTZ6	RBM12	RNA-bindir	97.395	932	4.041034	2.014724	+	1	13.08755	11.07282
Q9UGI8	TES	Testin OS=	47.996	421	4.02498	2.008982	+	2	14.02972	12.02074
Q9NW13	RBM28	RNA-bindir	85.738	759	4.02366	2.008508	+	1	13.08165	11.07314
Q86Y07	VRK2	Serine/thre	58.141	508	4.023631	2.008498	+	1	13.8574	11.8489
Q8IZ69	TRMT2A	tRNA (urac	68.726	625	4.014631	2.005268	+	1	12.68709	10.68182
P55265	ADAR	Double-stra	136.066	1226	4.01164	2.004192	+	2	14.60687	12.60268
P14373	TRIM27	Zinc finger	58.49	513	4.003977	2.001434	+	1	12.76008	10.75864
Q6QNY1	BLOC1S2	Biogenesis	15.961	142	3.999941	1.999979	+	1	12.61972	10.61974
Q8NHQ8	RASSF8	Ras associ	48.327	419	3.997005	1.998919	+	1	13.79391	11.79499
Q05655	PRKCD	Protein kin	77.505	676	3.995794	1.998482	+	1	13.51508	11.5166
P24539	ATP5PB	ATP syntha	28.909	256	3.995401	1.99834	+	1	13.4732	11.47486
Q8WUR7	C15orf40	UPF0235 p	16.353	153	3.994693	1.998085	+	1	12.75989	10.7618
Q53EL6	PDCD4	Programm	51.735	469	3.982896	1.993818	+	1	13.85185	11.85803
Q15070	OXA1L	Mitochondr	48.548	435	3.977403	1.991827	+	1	12.56677	10.57494
P32929	CTH	Cystathioni	44.508	405	3.974605	1.990812	+	1	12.92472	10.93391
Q13363	CTBP1	C-terminal-	47.535	440	3.971687	1.989752	+	1	11.05643	9.066683
O60762	DPM1	Dolichol-ph	29.634	260	3.971131	1.98955	+	1	13.65866	11.66911
Q14807	KIF22	Kinesin-like	73.262	665	3.968166	1.988472	+	1	12.59061	10.60214
P29372	MPG	DNA-3-met	32.869	298	3.966443	1.987846	+	1	13.09484	11.10699
Q9UII4	HERC5	E3 ISG15--	116.852	1024	3.955191	1.983747	+	1	13.38721	11.40346
P12235	SLC25A4	ADP/ATP t	33.065	298	3.953306	1.98306	+	1	12.48298	10.49992

Q14966	ZNF638	Zinc finger	220.625	1978	3.951759	1.982495	+	1	13.01432	11.03182
Q9NQY0	BIN3	Bridging int	29.665	253	3.942512	1.979115	+	2	14.925	12.94588
P51948	MNAT1	CDK-activa	35.823	309	3.937449	1.977261	+	1	12.69122	10.71396
P30566	ADSL	Adenylosuc	54.889	484	3.936138	1.976781	+	1	12.209	10.23222
Q15648	MED1	Mediator of	168.478	1581	3.91952	1.970677	+	1	11.65468	9.684004
Q9H7E2	TDRD3	Tudor dom	73.185	651	3.914593	1.968862	+	1	12.01332	10.04446
O43175	PHGDH	D-3-phospl	56.651	533	3.906682	1.965944	+	2	15.67535	13.70941
Q15428	SF3A2	Splicing fac	49.256	464	3.895826	1.961929	+	1	13.49548	11.53355
Q8TDX7	NEK7	Serine/thre	34.551	302	3.89123	1.960226	+	1	12.58815	10.62793
P42356	PI4KA	Phosphatid	236.83	2102	3.872922	1.953422	+	1	10.94141	8.987992
P09913	IFIT2	Interferon-i	54.632	472	3.870313	1.95245	+	1	12.08557	10.13312
A2A3N6	PIPSL	Putative PI	95.048	862	3.870234	1.952421	+	1	13.13305	11.18063
Q14166	TTLL12	Tubulin--tyı	74.404	644	3.860262	1.948699	+	1	12.87771	10.92901
Q9NQ55	PPAN	Suppresso	53.194	473	3.857969	1.947842	+	1	13.64971	11.70186
Q9NQC3	RTN4	Reticulon-4	129.931	1192	3.85504	1.946746	+	1	12.85781	10.91106
P53990	IST1	IST1 homo	39.751	364	3.853245	1.946074	+	1	13.19846	11.25239
Q6PK04	CCDC137	Coiled-coil	33.231	289	3.851776	1.945524	+	1	13.10524	11.15971
Q9BTT4	MED10	Mediator of	15.688	135	3.847219	1.943816	+	1	12.64437	10.70056
P05388	RPLP0	60S acidic	34.274	317	3.844935	1.942959	+	2	18.05197	16.10901
P53007	SLC25A1	Tricarboxyl	34.013	311	3.822758	1.934614	+	1	12.7039	10.76929
P49023	PXN	Paxillin OS	64.505	591	3.807651	1.928901	+	1	12.75343	10.82452
Q13596	SNX1	Sorting nex	59.07	522	3.800814	1.926309	+	1	11.56081	9.634502
P40855	PEX19	Peroxisom	32.807	299	3.797832	1.925176	+	1	12.57795	10.65277
Q15181	PPA1	Inorganic p	32.66	289	3.795863	1.924428	+	2	15.4912	13.56677
P05455	SSB	Lupus La p	46.837	408	3.793667	1.923593	+	2	16.0593	14.13571
Q9Y5X1	SNX9	Sorting nex	66.592	595	3.792226	1.923045	+	2	16.41245	14.48941
Q08AE8	SPIRE1	Protein spi	85.544	756	3.789183	1.921887	+	1	12.8079	10.88601
Q9NXH9	TRMT1	tRNA (guar	72.234	659	3.786535	1.920878	+	1	11.98702	10.06614
Q13838	DDX39B	Spliceosom	48.991	428	3.780081	1.918417	+	2	15.85072	13.93231
O15511	ARPC5	Actin-relate	16.32	151	3.77626	1.916958	+	2	15.74062	13.82367
Q86UW9	DTX2	Probable E	67.246	622	3.772614	1.915564	+	1	12.00783	10.09227
Q96QK1	VPS35	Vacuolar p	91.707	796	3.768022	1.913807	+	1	12.57466	10.66086
O60613	SELENOF	Selenoprot	18.092	165	3.767689	1.91368	+	1	12.00748	10.0938
Q8WTT2	NOC3L	Nucleolar c	92.548	800	3.766545	1.913242	+	1	12.07464	10.1614
P05091	ALDH2	Aldehyde d	56.381	517	3.756132	1.909248	+	1	11.764	9.854753
Q9H4G4	GLIPR2	Golgi-asso	17.218	154	3.750894	1.907235	+	2	17.0002	15.09296
P00558	PGK1	Phosphogly	44.615	417	3.747532	1.905941	+	2	13.97791	12.07197
Q9UBI6	GNG12	Guanine nu	8.006	72	3.74659	1.905578	+	2	19.83275	17.92718
Q16763	UBE2S	Ubiquitin-co	23.845	222	3.73893	1.902625	+	1	12.90398	11.00135
Q9UHR4	BAIAP2L1	Brain-spec	56.883	511	3.733537	1.900543	+	2	16.11517	14.21462
O95782	AP2A1	AP-2 comp	107.546	977	3.724795	1.897161	+	2	18.86351	16.96635
O94979	SEC31A	Protein trar	133.015	1220	3.714347	1.893109	+	1	12.42569	10.53258
P10646	TFPI	Tissue fact	35.015	304	3.713574	1.892808	+	1	12.93693	11.04412
P08621	SNRNP70	U1 small nu	51.557	437	3.712799	1.892507	+	1	13.26774	11.37523
P09543	CNP	2',3'-cyclic-	47.579	421	3.708101	1.890681	+	1	13.03909	11.14841
P61006	RAB8A	Ras-related	23.668	207	3.698268	1.88685	+	2	15.22976	13.34291
Q92541	RTF1	RNA polym	80.313	710	3.697962	1.88673	+	1	12.66973	10.783
Q96C86	DCPS	m7GpppX	38.609	337	3.697016	1.886361	+	1	12.86409	10.97773
O95573	ACSL3	Fatty acid (	80.42	720	3.695645	1.885826	+	2	14.49135	12.60553
O96007	MOCS2	Molybdopte	20.944	188	3.682247	1.880586	+	1	11.30047	9.419881
Q9BRP8	PYM1	Partner of '	22.656	204	3.677549	1.878745	+	1	12.86612	10.98738
Q9BQ04	RBM4B	RNA-bindir	40.15	359	3.677466	1.878712	+	1	12.5885	10.70979
Q8N9B5	JMY	Junction-m	111.445	988	3.674828	1.877677	+	1	13.0638	11.18612
P49642	PRIM1	DNA prima	49.902	420	3.669752	1.875683	+	1	12.5499	10.67422
Q99811	PRRX2	Paired mes	27.079	253	3.66868	1.875261	+	1	11.69139	9.816134
Q9NYF8	BCLAF1	Bcl-2-asso	106.122	920	3.665494	1.874008	+	2	16.02518	14.15118
Q9H0A0	NAT10	RNA cytidir	115.73	1025	3.663929	1.873391	+	1	12.92754	11.05415
P50995	ANXA11	Annexin A1	54.39	505	3.66302	1.873034	+	1	11.61356	9.740526
P39060	COL18A1	Collagen al	178.188	1754	3.662162	1.872696	+	1	13.12414	11.25144

P11802	CDK4	Cyclin-depe	33.73	303	3.659308	1.871571	+	1	13.58097	11.70939
P08240	SRPRA	Signal reco	69.811	638	3.649136	1.867555	+	1	12.47035	10.6028
P37235	HPCAL1	Hippocalcin	22.313	193	3.649105	1.867543	+	1	11.48704	9.619493
P55060	CSE1L	Exportin-2	110.417	971	3.646622	1.866561	+	1	13.77015	11.90359
Q86VP6	CAND1	Cullin-asso	136.376	1230	3.643169	1.865194	+	2	13.55327	11.68808
P08590	MYL3	Myosin ligh	21.932	195	3.63594	1.862328	+	2	21.05775	19.19542
Q4G0N4	NADK2	NAD kinase	49.433	442	3.630999	1.860367	+	1	12.6444	10.78403
Q96B23	C18orf25	Uncharacte	43.395	404	3.626841	1.858713	+	1	11.69401	9.835296
P51636	CAV2	Caveolin-2	18.291	162	3.624795	1.8579	+	1	11.8677	10.0098
Q68CQ4	UTP25	U3 small nu	87.055	756	3.622677	1.857056	+	1	12.22011	10.36305
Q9BVJ6	UTP14A	U3 small nu	87.978	771	3.617168	1.854861	+	1	13.04109	11.18623
Q9BUE0	MED18	Mediator of	23.663	208	3.605698	1.850279	+	1	12.31266	10.46238
P67775	PPP2CA	Serine/thre	35.594	309	3.605443	1.850176	+	2	15.25425	13.40408
Q9H3Q1	CDC42EP4	Cdc42 effe	37.98	356	3.597877	1.847146	+	1	13.23398	11.38683
Q96IU4	ABHD14B	Protein ABI	22.346	210	3.596136	1.846447	+	1	12.85146	11.00501
P56537	EIF6	Eukaryotic	26.599	245	3.593395	1.845348	+	2	13.73143	11.88608
Q9H4G0	EPB41L1	Band 4.1-lil	98.503	881	3.585953	1.842356	+	1	12.35604	10.51368
Q05639	EEF1A2	Elongation	50.47	463	3.582355	1.840908	+	2	15.61033	13.76942
Q16795	NDUFA9	NADH dehy	42.51	377	3.577844	1.83909	+	1	12.04258	10.20349
Q9NRG0	CHRAC1	Chromatin	14.711	131	3.577224	1.83884	+	1	12.38861	10.54977
P25787	PSMA2	Proteasom	25.899	234	3.576428	1.838519	+	1	12.64275	10.80423
P63220	RPS21	40S riboso	9.111	83	3.574666	1.837809	+	2	16.83269	14.99488
Q8NDV7	TNRC6A	Trinucleotic	210.297	1962	3.572202	1.836814	+	1	12.28794	10.45113
Q9BQ52	ELAC2	Zinc phosp	92.219	826	3.571154	1.83639	+	1	12.19112	10.35473
Q06265	EXOSC9	Exosome c	48.949	439	3.56982	1.835852	+	1	12.11293	10.27708
P47897	QARS1	Glutamine-	87.799	775	3.564878	1.833853	+	1	13.30107	11.46721
Q9BY42	RTF2	Replication	33.887	306	3.56452	1.833708	+	1	12.57738	10.74367
Q99497	PARK7	Parkinson (	19.891	189	3.562659	1.832954	+	1	12.93791	11.10495
O75312	ZPR1	Zinc finger	50.925	459	3.561793	1.832604	+	1	12.31871	10.48611
Q5BKZ1	ZNF326	DBIRD con	65.654	582	3.550929	1.828196	+	2	16.48278	14.65458
P19256	CD58	Lymphocyt	28.147	250	3.541567	1.824388	+	2	16.59335	14.76896
Q96J02	ITCH	E3 ubiquitin	102.803	903	3.537858	1.822876	+	1	11.23278	9.409905
Q9HC35	EML4	Echinoderr	108.916	981	3.536263	1.822226	+	1	13.30007	11.47784
Q9NX40	OCIAD1	OCIA doma	27.626	245	3.525745	1.817928	+	1	12.19927	10.38135
Q14690	PDCD11	Protein RR	208.701	1871	3.509763	1.811374	+	1	11.95902	10.14765
Q9NZL9	MAT2B	Methionine	37.552	334	3.509025	1.81107	+	1	13.05691	11.24584
Q9UJX5	ANAPC4	Anaphase-	92.116	808	3.5071	1.810279	+	1	12.81262	11.00234
O96013	PAK4	Serine/thre	64.072	591	3.506318	1.809957	+	1	12.50879	10.69883
Q9NZH0	GPRC5B	G-protein c	44.795	403	3.495111	1.805338	+	1	13.44424	11.6389
Q9Y5K8	ATP6V1D	V-type prot	28.263	247	3.483922	1.800712	+	1	12.14093	10.34021
O95363	FARS2	Phenylalan	52.357	451	3.479321	1.798806	+	1	11.37314	9.574331
Q9UNH7	SNX6	Sorting nex	46.649	406	3.474789	1.796925	+	1	12.28245	10.48553
Q9UH99	SUN2	SUN doma	80.311	717	3.473435	1.796363	+	1	12.71528	10.91891
O95297	MPZL1	Myelin prot	29.082	269	3.472022	1.795776	+	1	13.59339	11.79762
P46940	IQGAP1	Ras GTPas	189.252	1657	3.469278	1.794635	+	2	18.79934	17.0047
Q9NPA0	EMC7	ER membr	26.471	242	3.463604	1.792274	+	1	11.85502	10.06275
Q13049	TRIM32	E3 ubiquitin	71.989	653	3.460517	1.790988	+	1	12.39414	10.60315
P04156	PRNP	Major prior	27.661	253	3.458564	1.790173	+	2	17.33987	15.54969
O75695	RP2	Protein XR	39.641	350	3.454472	1.788465	+	2	17.45563	15.66717
O14939	PLD2	Phospholip	105.987	933	3.434961	1.780294	+	1	12.8024	11.0221
Q5VTB9	RNF220	E3 ubiquitin	62.765	566	3.431528	1.778851	+	1	11.80353	10.02467
P27361	MAPK3	Mitogen-ac	43.136	379	3.431142	1.778689	+	1	12.27543	10.49674
Q9UKK9	NUDT5	ADP-sugar	24.328	219	3.430766	1.778531	+	1	10.42459	8.646056
P08237	PFKM	ATP-deper	85.183	780	3.426333	1.776666	+	1	13.4397	11.66304
Q9Y3L5	RAP2C	Ras-relatec	20.745	183	3.423969	1.77567	+	2	16.18294	14.40727
Q29980	MICB	MHC class	42.575	383	3.421578	1.774662	+	1	12.75236	10.9777
P49184	DNASE1L1	Deoxyribor	33.893	302	3.417824	1.773078	+	2	16.99546	15.22238
L0R8F8	MIEF1	MIEF1 ups	8.445	70	3.415401	1.772055	+	1	11.51254	9.740488
O14818	PSMA7	Proteasom	27.887	248	3.413141	1.7711	+	2	16.04322	14.27212

Q86Y82	STX12	Syntaxin-1:	31.642	276	3.411375	1.770354	+	1	11.51713	9.746775
Q13813	SPTAN1	Spectrin al	284.539	2472	3.398522	1.764908	+	2	20.02513	18.26022
Q9Y2V2	CARHSP1	Calcium-re	15.892	147	3.390913	1.761674	+	1	12.63445	10.87277
Q8N5W9	RFLNB	Refilin-B O	22.882	214	3.387991	1.76043	+	2	16.65662	14.89619
Q6NXT1	ANKRD54	Ankyrin rep	32.505	300	3.38682	1.759931	+	1	10.99428	9.234351
Q09161	NCBP1	Nuclear ca	91.839	790	3.383539	1.758533	+	2	15.52506	13.76653
Q9NWV8	BABAM1	BRISC anc	36.56	329	3.380801	1.757365	+	1	11.82492	10.06755
Q9Y2T3	GDA	Guanine de	51.003	454	3.379857	1.756962	+	1	12.63347	10.87651
Q9UQE7	SMC3	Structural r	141.542	1217	3.377379	1.755904	+	1	13.05418	11.29828
O14980	XPO1	Exportin-1	123.386	1071	3.376051	1.755337	+	1	13.36687	11.61153
Q04917	YWHAH	14-3-3 prot	28.219	246	3.374955	1.754868	+	2	14.78156	13.0267
Q9UKV8	AGO2	Protein arg	97.208	859	3.372031	1.753618	+	1	11.91177	10.15815
O60678	PRMT3	Protein arg	59.903	531	3.371447	1.753368	+	1	12.614	10.86063
Q00059	TFAM	Transcriptio	29.097	246	3.370399	1.752919	+	2	15.80896	14.05604
Q9NQG5	RPRD1B	Regulation	36.9	326	3.36814	1.751952	+	1	12.43679	10.68484
P51114	FXR1	Fragile X m	69.721	621	3.362216	1.749412	+	2	16.61684	14.86742
O00203	AP3B1	AP-3 comp	121.32	1094	3.360334	1.748605	+	1	11.79576	10.04715
Q3KQU3	MAP7D1	MAP7 dom	92.82	841	3.351715	1.744899	+	1	11.63567	9.890773
O43678	NDUFA2	NADH deh	10.922	99	3.348032	1.743313	+	1	12.8082	11.06489
P15170	GSPT1	Eukaryotic	55.756	499	3.346616	1.742703	+	2	14.50575	12.76305
Q8IX12	CCAR1	Cell divisio	132.821	1150	3.341341	1.740427	+	1	13.23764	11.49722
O14965	AURKA	Aurora kina	45.823	403	3.340981	1.740272	+	1	13.93774	12.19747
O75369	FLNB	Filamin-B (	278.164	2602	3.328123	1.734709	+	2	20.13283	18.39812
P40926	MDH2	Malate deh	35.503	338	3.326465	1.73399	+	2	15.22972	13.49573
Q9Y4F1	FARP1	FERM, AR	118.633	1045	3.322308	1.732186	+	1	13.35645	11.62427
O75607	NPM3	Nucleoplas	19.344	178	3.321939	1.732025	+	2	15.68203	13.95001
P28074	PSMB5	Proteasom	28.48	263	3.321121	1.73167	+	1	12.91135	11.17968
Q9UJ70	NAGK	N-acetyl-D-	37.376	344	3.320431	1.731371	+	1	13.14671	11.41534
Q9UJZ1	STOML2	Stomatin-li	38.534	356	3.310862	1.727207	+	2	15.60669	13.87949
Q8NEZ2	VPS37A	Vacuolar p	44.314	397	3.309701	1.726701	+	1	11.6506	9.923902
P35520	CBS	Cystathioni	60.587	551	3.300937	1.722876	+	1	11.9416	10.21872
P52747	ZNF143	Zinc finger	68.896	638	3.299905	1.722424	+	1	12.02697	10.30455
Q9NTJ3	SMC4	Structural r	147.182	1288	3.294293	1.719969	+	2	12.4041	10.68413
Q8TAA9	VANGL1	Vang-like p	59.975	524	3.290012	1.718093	+	1	13.23855	11.52046
Q9UGN5	PARP2	Poly [ADP-	66.206	583	3.28718	1.71685	+	1	12.25175	10.5349
Q9UBX3	SLC25A10	Mitochondr	31.282	287	3.281865	1.714516	+	1	12.72091	11.00639
Q96D15	RCN3	Reticulocal	37.493	328	3.277697	1.712682	+	2	14.72286	13.01018
Q9ULX3	NOB1	RNA-bindir	46.675	412	3.275633	1.711774	+	1	13.20004	11.48827
Q53S08	RAB6D	Ras-relatec	28.242	254	3.273659	1.710904	+	1	12.79391	11.083
O75044	SRGAP2	SLIT-ROB(	120.871	1071	3.272421	1.710358	+	1	12.10836	10.398
Q15397	PUM3	Pumilio hoı	73.584	648	3.253142	1.701834	+	1	12.56298	10.86115
Q9Y285	FARSA	Phenylalan	57.564	508	3.24918	1.700076	+	2	16.58229	14.88221
Q96FZ2	HMCES	Abasic site	40.575	354	3.24786	1.69949	+	1	12.25715	10.55766
Q9Y2X3	NOP58	Nucleolar p	59.578	529	3.2335	1.693096	+	1	11.30132	9.608228
Q01082	SPTBN1	Spectrin be	274.609	2364	3.227677	1.690496	+	2	19.27665	17.58615
O43583	DENR	Density-reg	22.092	198	3.224756	1.68919	+	1	13.38262	11.69343
O43776	NARS1	Asparagine	62.943	548	3.221373	1.687676	+	1	12.64869	10.96102
Q9Y221	NIP7	60S riboso	20.463	180	3.218398	1.686343	+	1	11.31436	9.628014
P43246	MSH2	DNA mism	104.743	934	3.218002	1.686165	+	1	13.58989	11.90372
Q8NEF9	SRFBP1	Serum resp	48.634	429	3.213911	1.68433	+	1	12.43413	10.7498
Q14008	CKAP5	Cytoskeleto	225.495	2032	3.204477	1.680089	+	1	12.46202	10.78193
P09914	IFIT1	Interferon-i	55.36	478	3.204026	1.679886	+	1	13.07862	11.39873
O60927	PPP1R11	E3 ubiquitir	13.953	126	3.199892	1.678023	+	1	12.83325	11.15522
Q9Y2X9	ZNF281	Zinc finger	96.915	895	3.19696	1.676701	+	1	10.80114	9.124442
Q86TS9	MRPL52	39S riboso	13.664	123	3.183657	1.670685	+	1	12.29715	10.62646
Q15545	TAF7	Transcriptio	40.259	349	3.1836	1.670659	+	2	13.38842	11.71776
Q15404	RSU1	Ras suppre	31.54	277	3.182295	1.670068	+	1	12.74193	11.07186
Q96AX1	VPS33A	Vacuolar p	67.611	596	3.182099	1.669979	+	1	12.08972	10.41974
O95299	NDUFA10	NADH deh	40.751	355	3.180049	1.669049	+	1	11.89679	10.22774

Q9P289	STK26	Serine/thre	46.529	416	3.178746	1.668458	+	1	13.03392	11.36546
Q15654	TRIP6	Thyroid rec	50.288	476	3.178613	1.668398	+	2	14.99033	13.32193
Q9UBB9	TFIP11	Tuftelin-inte	96.82	837	3.175744	1.667095	+	1	11.82309	10.15599
Q9UQ13	SHOC2	Leucine-ric	64.888	582	3.16923	1.664132	+	1	12.92959	11.26546
P39880	CUX1	Homeobox	164.187	1505	3.16631	1.662802	+	2	15.23028	13.56748
Q96EE3	SEH1L	Nucleopori	39.649	360	3.163742	1.661632	+	1	12.47723	10.81559
Q9Y4Y9	LSM5	U6 snRNA-	9.937	91	3.163393	1.661473	+	1	13.46161	11.80013
O75367	MACROH2	Core histor	39.617	372	3.162675	1.661145	+	1	13.407	11.74586
Q99832	CCT7	T-complex	59.367	543	3.162015	1.660844	+	2	15.03819	13.37735
P48556	PSMD8	26S protea	39.612	350	3.15273	1.656602	+	1	13.65631	11.99971
Q7L1Q6	BZW1	eIF5-mimic	48.043	419	3.152592	1.656539	+	1	11.63159	9.975048
Q9HBM1	SPC25	Kinetochor	26.153	224	3.146463	1.653731	+	1	12.65919	11.00546
Q86XZ4	SPATS2	Spermatog	59.545	545	3.146344	1.653676	+	1	12.65808	11.0044
Q9BRZ2	TRIM56	E3 ubiquitir	81.488	755	3.135762	1.648816	+	1	11.09395	9.44513
O60563	CCNT1	Cyclin-T1 (	80.685	726	3.133928	1.647972	+	1	11.84549	10.19752
Q9NZM5	NOP53	Ribosome	54.389	478	3.129394	1.645883	+	2	14.53679	12.89091
Q86U70	LDB1	LIM domai	46.533	411	3.116108	1.639745	+	1	12.12519	10.48544
P49327	FASN	Fatty acid s	273.427	2511	3.113206	1.638401	+	2	15.26217	13.62377
Q9H788	SH2D4A	SH2 domai	52.727	454	3.107666	1.635831	+	1	12.16239	10.52656
Q13618	CUL3	Cullin-3 OS	88.93	768	3.1064	1.635244	+	1	11.95696	10.32171
Q9H2H8	PPIL3	Peptidyl-pr	18.155	161	3.105606	1.634875	+	1	13.26456	11.62968
P22087	FBL	rRNA 2'-O-	33.784	321	3.102735	1.633541	+	2	14.99068	13.35714
P78310	CXADR	Coxsackie\	40.03	365	3.096906	1.630828	+	1	12.19626	10.56544
Q8TCS8	PNPT1	Polyribonu	85.951	783	3.093455	1.629219	+	1	11.85716	10.22795
P61019	RAB2A	Ras-relate	23.546	212	3.090387	1.627787	+	1	11.63086	10.00307
Q96PZ0	PUS7	Pseudouric	75.035	661	3.082579	1.624138	+	1	11.57009	9.945955
Q5EBL8	PDZD11	PDZ doma	16.131	140	3.07787	1.621932	+	2	16.32291	14.70098
O75152	ZC3H11A	Zinc finger	89.131	810	3.064436	1.615622	+	1	11.92403	10.30841
P49585	PCYT1A	Choline-ph	41.731	367	3.064285	1.615551	+	1	12.6636	11.04805
Q9Y5Q9	GTF3C3	General tra	101.272	886	3.062428	1.614676	+	1	12.52999	10.91532
Q29RF7	PDS5A	Sister chro	150.83	1337	3.053258	1.61035	+	1	12.81332	11.20297
Q14980	NUMA1	Nuclear mi	238.26	2115	3.050894	1.609232	+	1	11.69414	10.08491
P30154	PPP2R1B	Serine/thre	66.214	601	3.049366	1.608509	+	1	12.40069	10.79218
Q9UNP9	PPIE	Peptidyl-pr	33.431	301	3.049062	1.608365	+	1	12.93574	11.32737
O75947	ATP5PD	ATP syntha	18.491	161	3.044685	1.606293	+	2	15.10439	13.4981
Q9NVX2	NLE1	Notchless p	53.32	485	3.041323	1.604699	+	1	12.18463	10.57993
Q16637	SMN1	Survival mo	31.849	294	3.040398	1.60426	+	1	11.79116	10.1869
Q5U5X0	LYRM7	Complex II	11.955	104	3.039432	1.603802	+	1	13.54714	11.94333
O95229	ZWINT	ZW10 inter	31.293	277	3.027973	1.598352	+	1	11.26168	9.663331
Q9P2S5	WRAP73	WD repeat	51.588	460	3.024129	1.596519	+	1	11.78631	10.18979
O43293	DAPK3	Death-asso	52.536	454	3.023847	1.596385	+	2	18.23318	16.63679
Q9P2R7	SUCLA2	Succinate--	50.317	463	3.021989	1.595498	+	1	11.90745	10.31196
Q14978	NOLC1	Nucleolar a	73.603	699	3.01545	1.592373	+	2	16.80335	15.21098
P60228	EIF3E	Eukaryotic	52.221	445	3.015002	1.592159	+	2	16.68702	15.09486
Q9BZE1	MRPL37	39S riboso	48.118	423	3.01231	1.59087	+	1	13.18676	11.59589
Q14315	FLNC	Filamin-C (	291.022	2725	3.01085	1.590171	+	2	16.91076	15.32059
Q15102	PAFAH1B3	Platelet-act	25.734	231	3.007648	1.588636	+	1	11.35821	9.769576
Q5VZ89	DENND4C	DENN dom	212.711	1909	3.007515	1.588572	+	1	11.77194	10.18337
Q96DI7	SNRNP40	U5 small nu	39.311	357	2.99991	1.584919	+	2	14.51286	12.92794
Q15645	TRIP13	Pachytene	48.551	432	2.999757	1.584846	+	1	11.96246	10.37762
Q86UN3	RTN4RL2	Reticulon-4	46.106	420	2.999491	1.584718	+	2	16.33186	14.74714
Q8IWX8	CHERP	Calcium hc	103.702	916	2.998258	1.584124	+	2	14.29749	12.71337
Q14244	MAP7	Ensconsin	84.052	749	2.995313	1.582707	+	2	19.31416	17.73145
Q9Y3B7	MRPL11	39S riboso	20.683	192	2.993302	1.581738	+	2	17.19604	15.61431
O95983	MBD3	Methyl-CpG	32.844	291	2.991999	1.58111	+	1	12.70154	11.12044
P60953	CDC42	Cell divisio	21.259	191	2.985796	1.578115	+	2	19.75525	18.17714
Q9NRF8	CTPS2	CTP syntha	65.678	586	2.980176	1.575397	+	1	12.38462	10.80922
Q96TA2	YME1L1	ATP-deper	86.455	773	2.978519	1.574595	+	2	14.57973	13.00513
Q86U38	NOP9	Nucleolar p	69.438	636	2.97839	1.574532	+	1	12.04234	10.46781

Q14683	SMC1A	Structural r	143.233	1233	2.963912	1.567502	+	2	12.39984	10.83234
Q16881	TXNRD1	Thioredoxi	70.906	649	2.962482	1.566806	+	2	15.77679	14.20999
Q13185	CBX3	Chromobox	20.811	183	2.96248	1.566805	+	2	16.13589	14.56908
Q9UBI1	COMMD3	COMM dor	22.151	195	2.960481	1.565832	+	1	11.78558	10.21974
P61221	ABCE1	ATP-bindin	67.314	599	2.959601	1.565403	+	1	13.36851	11.8031
Q9UQ80	PA2G4	Proliferatio	43.787	394	2.959313	1.565262	+	2	13.256	11.69074
Q15942	ZYX	Zyxin OS=I	61.277	572	2.959117	1.565167	+	2	14.28186	12.71669
Q01638	IL1RL1	Interleukin-	63.358	556	2.957969	1.564607	+	2	17.24488	15.68028
P82921	MRPS21	28S riboso	10.689	87	2.955319	1.563314	+	1	12.00731	10.44399
Q5T8D3	ACBD5	Acyl-CoA-b	60.092	534	2.954925	1.563122	+	1	11.53124	9.968114
Q9NZM3	ITSN2	Intersectin-	193.461	1697	2.951285	1.561343	+	2	13.93037	12.36902
P80723	BASP1	Brain acid s	22.693	227	2.950064	1.560746	+	2	21.62377	20.06303
P13804	ETFA	Electron tra	35.08	333	2.949491	1.560466	+	1	12.8027	11.24223
P46060	RANGAP1	Ran GTPas	63.542	587	2.947219	1.559354	+	1	13.79888	12.23952
Q8WW12	PCNP	PEST prote	18.925	178	2.942202	1.556896	+	1	12.59248	11.03558
P20290	BTF3	Transcripti	22.168	206	2.934691	1.553208	+	2	15.70652	14.15331
P30519	HMOX2	Heme oxyg	36.033	316	2.933381	1.552565	+	1	12.3807	10.82814
P05387	RPLP2	60S acidic	11.665	115	2.927483	1.549661	+	2	19.34713	17.79747
Q9Y2R9	MRPS7	28S riboso	28.134	242	2.922525	1.547216	+	2	15.28584	13.73862
Q86V81	ALYREF	THO comp	26.888	257	2.920303	1.546118	+	2	17.38093	15.83482
Q8WXD5	GEMIN6	Gem-asso	18.824	167	2.917837	1.544899	+	1	11.87556	10.33066
Q14684	RRP1B	Ribosomal	84.428	758	2.910778	1.541405	+	1	13.05036	11.50895
Q8IXK0	PHC2	Polyhomeo	90.713	858	2.906596	1.539331	+	1	13.12824	11.58891
P21266	GSTM3	Glutathione	26.56	225	2.903192	1.53764	+	2	14.61752	13.07988
P46937	YAP1	Transcripti	54.462	504	2.902783	1.537437	+	1	11.98306	10.44563
O14686	KMT2D	Histone-lys	593.389	5537	2.900575	1.536339	+	1	14.17532	12.63898
P48730	CSNK1D	Casein kin	47.33	415	2.898147	1.535131	+	1	12.58644	11.05131
P14384	CPM	Carboxype	50.514	443	2.895689	1.533906	+	2	19.39995	17.86605
Q9Y3E2	BOLA1	BolA-like p	14.289	137	2.892843	1.532488	+	1	12.69788	11.16539
Q9Y4F5	CEP170B	Centrosom	171.688	1589	2.875344	1.523735	+	1	13.13449	11.61076
O00273	DFFA	DNA fragm	36.522	331	2.872778	1.522446	+	1	12.97357	11.45113
P05386	RPLP1	60S acidic	11.514	114	2.867726	1.519907	+	2	18.88887	17.36897
O94916	NFAT5	Nuclear fac	165.763	1531	2.866915	1.519499	+	2	14.83847	13.31897
O00161	SNAP23	Synaptoso	23.354	211	2.865302	1.518687	+	2	18.64121	17.12252
Q9BVC5	C2orf49	Ashwin OS	25.858	232	2.865301	1.518687	+	1	12.43173	10.91305
P51572	BCAP31	B-cell rece	27.992	246	2.863407	1.517733	+	1	12.46633	10.9486
Q9H9L3	ISG20L2	Interferon-s	39.154	353	2.858955	1.515488	+	1	12.23056	10.71507
P04899	GNAI2	Guanine nu	40.451	355	2.855272	1.513628	+	2	17.99689	16.48326
P50990	CCT8	T-complex	59.621	548	2.851044	1.51149	+	2	15.8847	14.37321
P17858	PFKL	ATP-deper	85.018	780	2.850154	1.51104	+	1	11.56439	10.05335
P11717	IGF2R	Cation-inde	274.375	2491	2.848009	1.509954	+	1	11.84408	10.33412
Q9Y4W2	LAS1L	Ribosomal	83.065	734	2.84628	1.509078	+	1	12.72165	11.21258
Q9NZN4	EHD2	EH domain	61.161	543	2.837497	1.504619	+	1	11.6545	10.14988
Q07666	KHDRBS1	KH domain	48.227	443	2.835828	1.50377	+	2	18.64248	17.13871
Q01970	PLCB3	1-phosphat	138.799	1234	2.835404	1.503554	+	1	12.52351	11.01996
P49770	EIF2B2	Translation	38.99	351	2.823367	1.497417	+	1	11.60349	10.10607
P01112	HRAS	GTPase HI	21.298	189	2.823167	1.497314	+	2	16.28226	14.78494
Q01813	PFKP	ATP-deper	85.596	784	2.814371	1.492812	+	2	14.76937	13.27656
P82675	MRPS5	28S riboso	48.006	430	2.813833	1.492537	+	1	12.49386	11.00132
P78406	RAE1	mRNA exp	40.968	368	2.81004	1.490591	+	2	15.34009	13.8495
Q6P1N0	CC2D1A	Coiled-coil	104.062	951	2.807966	1.489526	+	1	12.38804	10.89852
P31153	MAT2A	S-adenosyl	43.661	395	2.80658	1.488813	+	2	15.12872	13.63991
Q9BRG1	VPS25	Vacuolar p	20.748	176	2.805916	1.488472	+	1	12.63234	11.14386
P50454	SERPINH1	Serpin H1 (	46.441	418	2.805421	1.488217	+	2	15.91991	14.43169
Q9H019	MTFR1L	Mitochondr	31.957	292	2.800662	1.485768	+	1	11.3609	9.875134
Q96CW1	AP2M1	AP-2 comp	49.655	435	2.797206	1.483986	+	2	18.13711	16.65312
Q96AZ6	ISG20	Interferon-s	20.363	181	2.796274	1.483506	+	1	11.52762	10.04412
Q9H3P7	ACBD3	Golgi resid	60.593	528	2.796095	1.483413	+	1	12.68126	11.19785
Q99700	ATXN2	Ataxin-2 O	140.283	1313	2.787935	1.479197	+	2	14.52687	13.04767

P54727	RAD23B	UV excisio	43.171	409	2.781761	1.475998	+	1	12.85748	11.38148
O75521	ECI2	Enoyl-CoA	43.585	394	2.780527	1.475358	+	1	12.41196	10.9366
Q86U42	PABPN1	Polyadenyl	32.749	306	2.775858	1.472934	+	2	15.41508	13.94215
Q32MZ4	LRRFIP1	Leucine-ric	89.253	808	2.769663	1.46971	+	2	17.28627	15.81656
Q7Z7C8	TAF8	Transcripti	34.262	310	2.768917	1.469322	+	1	13.29247	11.82314
Q7RTV0	PHF5A	PHD finger	12.405	110	2.767962	1.468824	+	1	12.51863	11.0498
O00459	PIK3R2	Phosphatid	81.545	728	2.767603	1.468637	+	1	11.38397	9.915337
Q9NXS2	QPCTL	Glutaminyl-	42.924	382	2.765294	1.467433	+	1	12.26036	10.79293
P49588	AARS1	Alanine--tR	106.81	968	2.760431	1.464894	+	1	11.72562	10.26073
O43847	NRDC	Nardilysin (	131.701	1151	2.755964	1.462557	+	1	11.27682	9.814265
Q13162	PRDX4	Peroxiredo	30.54	271	2.751847	1.4604	+	2	14.53442	13.07402
Q14847	LASP1	LIM and SH	29.717	261	2.750576	1.459734	+	2	15.39047	13.93074
P08651	NFIC	Nuclear fac	55.675	508	2.749983	1.459422	+	1	12.5636	11.10418
Q14254	FLOT2	Flotillin-2 C	47.064	428	2.747364	1.458048	+	2	17.47849	16.02044
Q9H840	GEMIN7	Gem-assoc	14.537	131	2.744037	1.4563	+	1	10.83162	9.375324
Q16851	UGP2	UTP--glucc	56.94	508	2.74268	1.455586	+	1	12.53715	11.08156
O00422	SAP18	Histone de	17.561	153	2.735413	1.451759	+	2	16.75486	15.3031
Q6ZRV2	FAM83H	Protein FAI	127.122	1179	2.729289	1.448525	+	1	13.42206	11.97354
P82663	MRPS25	28S riboso	20.116	173	2.727867	1.447773	+	2	15.02626	13.57849
Q99933	BAG1	BAG family	38.779	345	2.722312	1.444833	+	1	10.74542	9.30059
Q16644	MAPKAPK	MAP kinas	42.987	382	2.719815	1.443508	+	1	12.31419	10.87068
Q96MU7	YTHDC1	YTH doma	84.7	727	2.719798	1.4435	+	1	11.73674	10.29324
Q9NUU7	DDX19A	ATP-deper	53.975	478	2.719113	1.443136	+	1	13.32334	11.8802
P40939	HADHA	Trifunction	83	763	2.718448	1.442783	+	2	18.15595	16.71317
Q96H79	ZC3HAV1L	Zinc finger	32.962	300	2.716424	1.441709	+	1	11.86593	10.42422
Q9H6W3	RIOX1	Ribosomal	71.086	641	2.708662	1.437581	+	1	12.35436	10.91678
P21333	FLNA	Filamin-A (	280.739	2647	2.707124	1.436761	+	2	20.00086	18.5641
P49321	NASP	Nuclear au	85.238	788	2.701717	1.433877	+	1	13.05885	11.62498
P08047	SP1	Transcripti	80.693	785	2.700642	1.433302	+	1	11.78353	10.35023
Q13435	SF3B2	Splicing fac	100.228	895	2.700134	1.433031	+	2	16.15167	14.71864
P49761	CLK3	Dual specif	73.515	638	2.699309	1.43259	+	1	11.9158	10.48321
O75964	ATP5MG	ATP syntha	11.428	103	2.692709	1.429058	+	1	13.46531	12.03625
P11387	TOP1	DNA topois	90.726	765	2.688839	1.426983	+	2	16.20978	14.78279
O75190	DNAJB6	DnaJ homo	36.087	326	2.687668	1.426355	+	2	15.82309	14.39674
P25398	RPS12	40S riboso	14.515	132	2.682141	1.423385	+	2	18.40553	16.98215
Q7KZI7	MARK2	Serine/thre	87.911	788	2.676019	1.420088	+	1	11.23092	9.810833
Q9GZR2	REXO4	RNA exonu	46.672	422	2.667804	1.415653	+	1	12.32438	10.90872
Q9BQ67	GRWD1	Glutamate-	49.419	446	2.66572	1.414525	+	2	16.04422	14.6297
Q96N67	DOCK7	Dedicator c	242.561	2140	2.662416	1.412736	+	2	15.32787	13.91513
Q9Y4C8	RBM19	Probable R	107.332	960	2.65894	1.410851	+	1	12.10332	10.69247
O43615	TIMM44	Mitochondr	51.356	452	2.658092	1.410391	+	1	11.77079	10.3604
Q00653	NFKB2	Nuclear fac	96.749	900	2.6519	1.407026	+	1	12.53048	11.12345
Q86W42	THOC6	THO comp	37.535	341	2.651634	1.406882	+	1	12.68998	11.28309
Q92616	GCN1	eIF-2-alpha	292.71	2671	2.64539	1.403481	+	1	11.38354	9.980062
O60664	PLIN3	Perilipin-3 (	47.075	434	2.644023	1.402735	+	1	11.35678	9.954047
P10696	ALPG	Alkaline ph	57.377	532	2.639064	1.400027	+	2	16.19749	14.79746
Q9Y3D8	AK6	Adenylate I	20.061	172	2.635665	1.398167	+	1	11.85093	10.45276
Q969X5	ERGIC1	Endoplasm	32.592	290	2.634077	1.397298	+	2	13.93369	12.53639
Q6P158	DHX57	Putative AT	155.604	1386	2.626422	1.393099	+	1	11.41109	10.01799
O15145	ARPC3	Actin-relate	20.547	178	2.6258	1.392757	+	2	16.63991	15.24715
Q01650	SLC7A5	Large neutr	55.01	507	2.625161	1.392406	+	2	14.89685	13.50445
Q14697	GANAB	Neutral alp	106.874	944	2.62319	1.391322	+	2	13.63878	12.24745
P49406	MRPL19	39S riboso	33.535	292	2.618624	1.388809	+	1	11.8121	10.42329
O75822	EIF3J	Eukaryotic	29.062	258	2.613136	1.385782	+	1	12.39326	11.00747
O15031	PLXNB2	Plexin-B2 (	205.127	1838	2.598487	1.377672	+	1	11.93793	10.56025
Q13685	AAMP	Angio-asso	46.751	434	2.594882	1.375669	+	1	13.20811	11.83244
Q13356	PPIL2	RING-type	58.823	520	2.585226	1.37029	+	1	12.07129	10.701
P55795	HNRNPH2	Heterogene	49.264	449	2.584147	1.369688	+	2	17.06266	15.69297
Q01844	EWSR1	RNA-bindir	68.478	656	2.581472	1.368194	+	2	16.49082	15.12263

Q16864	ATP6V1F	V-type prot	13.37	119	2.578198	1.366363	+	1	12.90805	11.54169
P53618	COPB1	Coatomer s	107.142	953	2.571872	1.362819	+	2	13.8319	12.46908
Q8WWC4	MAIP1	m-AAA pro	32.545	291	2.566658	1.359891	+	1	12.53277	11.17288
Q9NR56	MBNL1	Muscleblin	41.817	388	2.565916	1.359474	+	1	11.91506	10.55558
P10301	RRAS	Ras-related	23.48	218	2.563848	1.358311	+	2	16.75929	15.40098
Q9Y678	COPG1	Coatomer s	97.718	874	2.563102	1.357891	+	2	14.1817	12.82381
P19338	NCL	Nucleolin C	76.614	710	2.559249	1.355721	+	2	19.58562	18.2299
P49591	SARS1	Serine--tRN	58.777	514	2.558016	1.355025	+	1	11.29949	9.944469
P41091	EIF2S3	Eukaryotic	51.11	472	2.555672	1.353702	+	2	17.16447	15.81077
P38919	EIF4A3	Eukaryotic	46.871	411	2.554651	1.353126	+	2	15.90153	14.5484
Q9Y4C2	TCAF1	TRPM8 cha	102.126	921	2.55231	1.351804	+	1	11.77919	10.42738
Q9NX58	LYAR	Cell growth	43.634	379	2.550706	1.350897	+	1	12.801	11.4501
Q6ZVM7	TOM1L2	TOM1-like	55.556	507	2.550585	1.350828	+	1	12.32246	10.97164
Q9UI10	EIF2B4	Translation	57.557	523	2.550223	1.350623	+	1	11.29732	9.946694
Q14699	RFTN1	Raftlin OS=	63.146	578	2.549814	1.350392	+	1	12.40605	11.05565
Q15459	SF3A1	Splicing fac	88.886	793	2.549748	1.350355	+	2	15.30709	13.95674
Q14669	TRIP12	E3 ubiquitin	220.434	1992	2.548852	1.349847	+	1	12.80242	11.45257
Q9NYZ3	GTSE1	G2 and S p	76.645	720	2.548389	1.349586	+	1	12.49461	11.14502
Q14247	CTTN	Src substra	61.586	550	2.546574	1.348558	+	2	17.48605	16.13749
P07919	UQCRH	Cytochrom	10.739	91	2.545859	1.348152	+	1	11.52292	10.17477
Q07866	KLC1	Kinesin ligh	65.31	573	2.541135	1.345473	+	2	13.92193	12.57646
Q9H8Y5	ANKZF1	Ankyrin rep	80.927	726	2.54073	1.345243	+	1	13.06918	11.72394
P48681	NES	Nestin OS=	177.439	1621	2.540345	1.345024	+	2	17.6113	16.26627
Q9HCN8	SDF2L1	Stromal ce	23.598	221	2.53917	1.344357	+	1	12.26603	10.92167
P23588	EIF4B	Eukaryotic	69.151	611	2.538545	1.344002	+	2	15.65603	14.31203
P27448	MARK3	MAP/micro	84.429	753	2.536723	1.342966	+	1	11.90106	10.55809
Q9BXI6	TBC1D10A	TBC1 dom	57.118	508	2.536275	1.342711	+	1	12.87065	11.52794
O95639	CPSF4	Cleavage a	30.255	269	2.536077	1.342599	+	2	15.75099	14.4084
Q86W92	PPFIBP1	Liprin-beta-	114.024	1011	2.531549	1.34002	+	1	12.69336	11.35334
P36543	ATP6V1E1	V-type prot	26.145	226	2.530006	1.339141	+	2	14.34755	13.00841
Q04741	EMX1	Homeobox	31.295	290	2.524411	1.335947	+	1	12.68534	11.34939
O60870	KIN	DNA/RNA-	45.374	393	2.522704	1.334971	+	1	13.20601	11.87104
O75558	STX11	Syntaxin-1	33.196	287	2.520982	1.333986	+	1	12.21735	10.88337
P23921	RRM1	Ribonucleo	90.07	792	2.519498	1.333136	+	1	11.89137	10.55823
P51659	HSD17B4	Peroxisom	79.686	736	2.513439	1.329663	+	2	14.63458	13.30492
P53041	PPP5C	Serine/thre	56.879	499	2.512849	1.329324	+	1	11.52547	10.19615
O94826	TOMM70	Mitochondr	67.455	608	2.511885	1.32877	+	1	11.77207	10.4433
Q13247	SRSF6	Serine/argi	39.587	344	2.511188	1.32837	+	1	13.05985	11.73148
P08195	SLC3A2	4F2 cell-su	67.994	630	2.505734	1.325233	+	2	16.60441	15.27918
P29084	GTF2E2	Transcriptio	33.044	291	2.503073	1.3237	+	1	12.82546	11.50176
Q9Y6M9	NDUFB9	NADH deh	21.831	179	2.500156	1.322018	+	1	13.08468	11.76266
P49915	GMPS	GMP synth	76.715	693	2.489865	1.316068	+	1	12.78335	11.46728
P21281	ATP6V1B2	V-type prot	56.501	511	2.482972	1.312068	+	1	11.66875	10.35668
Q6RFH5	WDR74	WD repeat	42.441	385	2.476088	1.308063	+	1	11.90959	10.60153
P52789	HK2	Hexokinase	102.38	917	2.468301	1.303518	+	1	12.35818	11.05467
P47756	CAPZB	F-actin-cap	31.35	277	2.4633	1.300592	+	2	20.17194	18.87135
Q9H0A8	COMMD4	COMM dor	21.764	199	2.462286	1.299999	+	1	11.94789	10.64789
Q9NUQ3	TXLNG	Gamma-ta	60.586	528	2.461518	1.299548	+	1	12.02538	10.72583
O76003	GLRX3	Glutaredox	37.432	335	2.457465	1.297171	+	1	13.42705	12.12988
O95816	BAG2	BAG family	23.772	211	2.449805	1.292667	+	2	18.37386	17.08119
Q9H269	VPS16	Vacuolar p	94.694	839	2.445277	1.289998	+	1	11.71467	10.42468
P04792	HSPB1	Heat shock	22.783	205	2.44074	1.287319	+	2	18.47314	17.18582
Q9Y6W3	CAPN7	Calpain-7 (	92.652	813	2.439309	1.286473	+	1	10.92214	9.635666
P46459	NSF	Vesicle-fus	82.594	744	2.437113	1.285173	+	1	11.29439	10.00922
Q9H4A6	GOLPH3	Golgi phos	33.811	298	2.435808	1.2844	+	1	12.95877	11.67437
O43719	HTATSF1	HIV Tat-sp	85.853	755	2.43376	1.283187	+	1	11.00253	9.719347
Q15007	WTAP	Pre-mRNA	44.244	396	2.432647	1.282527	+	2	13.90595	12.62342
Q96FZ7	CHMP6	Charged m	23.485	201	2.429719	1.28079	+	1	12.74937	11.46858
Q8TAE8	GADD45G	Growth arr	25.384	222	2.427465	1.27945	+	2	14.70125	13.4218

O15066	KIF3B	Kinesin-like	85.125	747	2.422039	1.276222	+	1	11.60052	10.3243
P56556	NDUFA6	NADH deh	15.137	128	2.41726	1.273373	+	1	11.84482	10.57145
P59768	GNG2	Guanine nu	7.85	71	2.414923	1.271977	+	1	11.76645	10.49447
Q8WXI7	MUC16	Mucin-16 C	1519.175	14507	2.414458	1.271699	+	1	13.24914	11.97744
Q9GZM8	NDEL1	Nuclear dis	38.375	345	2.412424	1.270484	+	1	12.90243	11.63195
Q96EP5	DAZAP1	DAZ-assoc	43.383	407	2.410355	1.269246	+	2	14.87623	13.60698
P32242	OTX1	Homeobox	37.327	354	2.409326	1.26863	+	1	12.1315	10.86287
Q8TF74	WIPF2	WAS/WAS	46.289	440	2.408835	1.268335	+	1	11.99499	10.72665
O75179	ANKRD17	Ankyrin rep	274.258	2603	2.408417	1.268085	+	1	12.63513	11.36704
P18859	ATP5PF	ATP syntha	12.588	108	2.40798	1.267824	+	1	13.48596	12.21813
P10644	PRKAR1A	cAMP-depe	42.982	381	2.40577	1.266499	+	1	12.25252	10.98602
P84077	ARF1	ADP-ribosy	20.697	181	2.405261	1.266194	+	2	16.65158	15.38539
P67809	YBX1	Y-box-bind	35.924	324	2.40453	1.265755	+	2	18.85951	17.59376
Q9BZF9	UACA	Uveal auto	162.505	1416	2.402892	1.264772	+	2	16.05609	14.79132
Q9NZZ3	CHMP5	Charged m	24.571	219	2.402675	1.264642	+	2	16.60244	15.3378
Q13829	TNFAIP1	BTB/POZ c	36.204	316	2.402525	1.264551	+	1	13.10527	11.84072
O95429	BAG4	BAG family	49.594	457	2.399861	1.262951	+	2	13.42757	12.16462
O43815	STRN	Striatin OS	86.132	780	2.399535	1.262755	+	1	11.48467	10.22192
P61313	RPL15	60S riboso	24.146	204	2.399176	1.262539	+	2	16.45317	15.19063
Q9NUG6	PDRG1	p53 and DI	15.511	133	2.397146	1.261318	+	1	12.63336	11.37204
Q96I51	RCC1L	RCC1-like	49.898	464	2.396951	1.2612	+	1	12.41191	11.15071
P54619	PRKAG1	5'-AMP-act	37.579	331	2.396536	1.26095	+	1	11.52635	10.2654
P20839	IMPDH1	Inosine-5'-I	55.406	514	2.391258	1.25777	+	2	16.29424	15.03647
Q8IXB1	DNAJC10	DnaJ homo	91.08	793	2.391247	1.257763	+	1	11.71939	10.46163
Q9UMS6	SYNPO2	Synaptopo	117.514	1093	2.386123	1.254668	+	1	11.664	10.40933
P05198	EIF2S1	Eukaryotic	36.112	315	2.386037	1.254617	+	2	15.34762	14.093
P11171	EPB41	Protein 4.1	97.017	864	2.375737	1.248375	+	1	13.01056	11.76219
O14561	NDUFAB1	Acyl carrier	17.417	156	2.373531	1.247035	+	2	14.27968	13.03265
Q12800	TFCP2	Alpha-globi	57.256	502	2.37085	1.245405	+	2	16.36037	15.11496
O94806	PRKD3	Serine/thre	100.471	890	2.37075	1.245343	+	1	11.24151	9.996163
O95980	RECK	Reversion-	106.457	971	2.369186	1.244391	+	1	12.73634	11.49195
Q92542	NCSTN	Nicastrin O	78.411	709	2.367418	1.243314	+	1	11.33349	10.09018
Q15032	R3HDM1	R3H doma	120.696	1099	2.365185	1.241953	+	1	12.51239	11.27044
P15311	EZR	Ezrin OS=I	69.413	586	2.365174	1.241946	+	2	14.92231	13.68036
O75716	STK16	Serine/thre	34.656	305	2.36502	1.241852	+	1	11.77746	10.53561
Q16555	DPYSL2	Dihydropyri	62.294	572	2.364864	1.241757	+	2	15.17333	13.93157
O00267	SUPT5H	Transcripti	121	1087	2.360774	1.23926	+	1	11.78721	10.54795
O43670	ZNF207	BUB3-inter	50.751	478	2.359985	1.238778	+	2	16.8964	15.65763
Q9Y5K5	UCHL5	Ubiquitin ca	37.607	329	2.350779	1.233139	+	1	12.75332	11.52018
P40763	STAT3	Signal tran	88.068	770	2.347615	1.231196	+	1	12.42438	11.19318
Q96FJ0	STAMBPL	AMSH-like	49.783	436	2.346338	1.230411	+	2	15.23665	14.00624
P17480	UBTF	Nucleolar t	89.406	764	2.339122	1.225967	+	1	12.76928	11.54331
Q8TCJ2	STT3B	Dolichyl-dip	93.674	826	2.336744	1.2245	+	1	12.39135	11.16685
Q9H3P2	NELFA	Negative el	57.277	528	2.334327	1.223007	+	1	12.31956	11.09655
Q9NRN7	AASDHPP	L-aminoadi	35.776	309	2.331068	1.220991	+	1	13.0195	11.79851
Q12789	GTF3C1	General tra	238.875	2109	2.330635	1.220723	+	1	11.45692	10.2362
Q15437	SEC23B	Protein tran	86.479	767	2.328949	1.219679	+	1	12.03827	10.81859
P98082	DAB2	Disabled h	82.448	770	2.325403	1.217481	+	2	17.56585	16.34837
O43837	IDH3B	Isocitrate d	42.184	385	2.322524	1.215694	+	1	12.43648	11.22078
Q9H9B4	SFXN1	Sideroflexi	35.619	322	2.321867	1.215286	+	1	11.57099	10.35571
Q9UL46	PSME2	Proteasom	27.402	239	2.321771	1.215225	+	1	12.60005	11.38483
P61160	ACTR2	Actin-relate	44.761	394	2.321014	1.214755	+	2	16.37577	15.16101
Q8NFW8	CMAS	N-acylneur	48.379	434	2.319994	1.214121	+	1	12.88232	11.6682
O15116	LSM1	U6 snRNA-	15.179	133	2.317022	1.212272	+	1	11.3564	10.14413
Q9P086	MED11	Mediator of	13.129	117	2.316057	1.21167	+	1	11.83158	10.61991
P11310	ACADM	Medium-ch	46.588	421	2.312336	1.209351	+	1	11.85436	10.64501
P49748	ACADVL	Very long-c	70.39	655	2.310519	1.208217	+	1	11.48512	10.27691
Q09666	AHNAK	Neuroblast	629.101	5890	2.308343	1.206858	+	2	20.09249	18.88563
Q96LI5	CNOT6L	CCR4-NOT	63.001	555	2.299046	1.201035	+	1	12.71894	11.5179

P63241	EIF5A	Eukaryotic	16.832	154	2.298753	1.200852	+	2	17.88184	16.68099
Q00610	CLTC	Clathrin he	191.615	1675	2.297971	1.200361	+	2	19.06731	17.86695
Q03393	PTS	6-pyruvoyl	16.386	145	2.295887	1.199052	+	1	12.25272	11.05367
O43396	TXNL1	Thioredoxi	32.251	289	2.291525	1.196308	+	1	12.46773	11.27143
Q14118	DAG1	Dystroglyca	97.441	895	2.2892	1.194844	+	1	11.834	10.63915
Q7Z2T5	TRMT1L	TRMT1-like	81.747	733	2.289064	1.194758	+	1	12.09024	10.89549
Q13144	EIF2B5	Translation	80.38	721	2.288415	1.194349	+	1	12.1458	10.95146
Q9ULC4	MCTS1	Malignant T	20.555	181	2.285574	1.192557	+	1	12.05399	10.86144
Q9GZU7	CTDSP1	Carboxy-te	29.203	261	2.285204	1.192323	+	1	10.85526	9.662935
Q9Y570	PPME1	Protein pho	42.315	386	2.284229	1.191707	+	1	12.39103	11.19932
Q14126	DSG2	Desmoglei	122.294	1118	2.280569	1.189394	+	2	19.69666	18.50727
Q5TAQ9	DCAF8	DDB1- and	66.852	597	2.280317	1.189234	+	1	10.97097	9.781734
Q9HAC8	UBTD1	Ubiquitin do	25.938	227	2.27999	1.189028	+	2	17.30519	16.11616
P61923	COPZ1	Coatomer s	20.198	177	2.27754	1.187477	+	2	13.06572	11.87824
P61225	RAP2B	Ras-related	20.504	183	2.272961	1.184573	+	2	15.46055	14.27598
Q9UHA4	LAMTOR3	Ragulator c	13.623	124	2.266585	1.18052	+	2	15.25182	14.07129
P07900	HSP90AA1	Heat shock	84.66	732	2.265531	1.179849	+	2	16.34082	15.16097
Q9H501	ESF1	ESF1 homo	98.796	851	2.260805	1.176837	+	1	12.61623	11.43939
Q9UKS6	PACSIN3	Protein kina	48.487	424	2.254711	1.172943	+	2	18.66331	17.49037
O14974	PPP1R12A	Protein pho	115.281	1030	2.250886	1.170493	+	2	19.45328	18.28279
P53365	ARFIP2	Arfaptin-2 (	37.856	341	2.250707	1.170378	+	1	12.07099	10.90061
O15400	STX7	Syntaxin-7	29.816	261	2.246451	1.167648	+	1	11.58651	10.41886
P26639	TARS1	Threonine-	83.435	723	2.244183	1.166191	+	2	14.31974	13.15355
Q9BXW9	FANCD2	Fanconi an	164.128	1451	2.23936	1.163086	+	2	18.4614	17.29831
P78345	RPP38	Ribonuclea	31.834	283	2.232747	1.15882	+	1	12.40785	11.24903
Q9UPN9	TRIM33	E3 ubiquitin	122.533	1127	2.231973	1.15832	+	1	11.03232	9.874001
P51571	SSR4	Translocon	18.999	173	2.230822	1.157575	+	2	15.92602	14.76844
O14908	GIPC1	PDZ doma	36.049	333	2.22764	1.155516	+	2	15.73052	14.57501
P45974	USP5	Ubiquitin ca	95.786	858	2.22681	1.154978	+	1	13.3126	12.15762
Q08379	GOLGA2	Golgin sub	113.086	1002	2.225088	1.153862	+	2	14.24459	13.09072
A6NDR6	MEIS3P1	Putative ho	30.204	274	2.222643	1.152277	+	1	12.98744	11.83517
P53621	COPA	Coatomer s	138.346	1224	2.222112	1.151931	+	2	13.93397	12.78204
Q92785	DPF2	Zinc finger	44.155	391	2.221081	1.151262	+	1	12.35309	11.20183
Q5PSV4	BRMS1L	Breast can	37.629	323	2.217645	1.149029	+	1	12.09358	10.94455
P86791	CCZ1	Vacuolar fu	55.866	482	2.216158	1.148061	+	1	10.68554	9.537476
P22234	PAICS	Multifunctio	47.079	425	2.213026	1.14602	+	2	16.28869	15.14267
O60828	PQBP1	Polyglutam	30.472	265	2.211825	1.145237	+	1	12.68486	11.53962
Q99828	CIB1	Calcium an	21.703	191	2.211631	1.145111	+	1	12.09744	10.95233
Q14677	CLINT1	Clathrin inte	68.259	625	2.208756	1.143234	+	2	20.11248	18.96925
P30085	CMPK1	UMP-CMP	22.222	196	2.208649	1.143164	+	2	13.88197	12.73881
Q9UKV3	ACIN1	Apoptotic c	151.862	1341	2.204475	1.140435	+	2	15.8752	14.73476
Q9ULV4	CORO1C	Coronin-1C	53.249	474	2.202244	1.138975	+	2	19.9042	18.76523
Q1KMD3	HNRNPUL	Heterogene	85.105	747	2.200354	1.137736	+	2	15.80252	14.66478
Q969V3	NCLN	Nicalin OS	62.974	563	2.194638	1.133983	+	1	11.4364	10.30242
P31948	STIP1	Stress-indu	62.639	543	2.194428	1.133845	+	2	14.42358	13.28973
P57088	TMEM33	Transmem	27.978	247	2.192482	1.132565	+	1	12.5039	11.37134
P48634	PRRC2A	Protein PR	228.863	2157	2.190305	1.131132	+	2	14.92616	13.79502
Q8TEX9	IPO4	Importin-4	118.715	1081	2.189407	1.13054	+	1	11.19937	10.06883
Q9H0H5	RACGAP1	Rac GTPas	71.027	632	2.187397	1.129215	+	2	17.22496	16.09575
Q9BZE4	GTPBP4	GTP-bindin	73.964	634	2.187299	1.12915	+	1	12.74309	11.61394
Q53F19	NCBP3	Nuclear ca	70.593	620	2.186708	1.12876	+	1	12.19525	11.06649
O60524	NEMF	Ribosome	122.954	1076	2.185991	1.128287	+	1	12.89773	11.76944
Q9BSJ8	ESYT1	Extended s	122.856	1104	2.182302	1.125851	+	2	14.20862	13.08276
Q53GQ0	HSD17B12	Very-long-c	34.324	312	2.178407	1.123273	+	1	11.31832	10.19504
Q9H0P0	NT5C3A	Cytosolic 5	37.948	336	2.176512	1.122018	+	1	12.51222	11.3902
Q9BRJ2	MRPL45	39S riboso	35.351	306	2.175819	1.121558	+	1	11.11647	9.994916
Q9NVA2	SEPTIN11	Septin-11 (	49.398	429	2.174048	1.120384	+	1	12.38646	11.26607
Q7Z7F7	MRPL55	39S riboso	15.128	128	2.169307	1.117234	+	1	12.72932	11.61209
Q8N0X7	SPART	Spartin OS	72.833	666	2.166193	1.115162	+	1	12.2643	11.14913

Q8WYL5	SSH1	Protein pho	115.511	1049	2.16433	1.11392	+	1	12.05542	10.9415
P11166	SLC2A1	Solute carr	54.084	492	2.161127	1.111784	+	1	13.63651	12.52473
Q05193	DNM1	Dynamin-1	97.408	864	2.161115	1.111776	+	1	10.25231	9.140535
Q9BTY7	HGH1	Protein HG	42.129	390	2.160951	1.111667	+	1	11.83356	10.7219
P52435	POLR2J	DNA-direct	13.293	117	2.160656	1.11147	+	1	13.19663	12.08516
Q8WVM8	SCFD1	Sec1 family	72.38	642	2.159466	1.110675	+	1	11.16786	10.05718
Q9NPQ8	RIC8A	Synembryn	59.71	531	2.158844	1.110259	+	1	11.56458	10.45432
O95985	TOP3B	DNA topois	96.662	862	2.14591	1.101589	+	1	11.58745	10.48586
Q13347	EIF3I	Eukaryotic	36.502	325	2.144357	1.100545	+	2	15.11744	14.0169
O00303	EIF3F	Eukaryotic	37.564	357	2.142565	1.099339	+	2	15.67866	14.57932
Q86V48	LUZP1	Leucine zip	120.275	1076	2.14234	1.099187	+	2	18.46132	17.36213
Q96MX6	DNAAF10	Dynein axo	39.74	357	2.136537	1.095275	+	1	11.75096	10.65568
Q8NI36	WDR36	WD repeat	105.322	951	2.134061	1.093602	+	1	11.44827	10.35467
Q9P227	ARHGAP2	Rho GTPa	162.192	1491	2.12972	1.090664	+	2	13.64858	12.55792
P55209	NAP1L1	Nucleosom	45.374	391	2.129273	1.090361	+	2	16.60029	15.50993
Q0ZGT2	NEXN	Nexilin OS	80.658	675	2.124475	1.087106	+	2	17.17699	16.08988
P60842	EIF4A1	Eukaryotic	46.154	406	2.122326	1.085646	+	2	16.19633	15.11069
P30040	ERP29	Endoplasm	28.993	261	2.120052	1.0841	+	1	11.27607	10.19197
Q9NRA8	EIF4ENIF1	Eukaryotic	108.201	985	2.109663	1.077012	+	1	11.68308	10.60607
P06396	GSN	Gelsolin O	85.698	782	2.107231	1.075349	+	2	18.54774	17.47239
Q9Y281	CFL2	Cofilin-2 O	18.737	166	2.102376	1.072021	+	2	16.30001	15.22799
Q96RL1	UIMC1	BRCA1-A c	79.727	719	2.101187	1.071205	+	1	13.14246	12.07125
Q9UJC5	SH3BGRL	SH3 domai	12.326	107	2.100223	1.070542	+	2	16.9138	15.84326
Q14320	FAM50A	Protein FA	40.242	339	2.096625	1.068069	+	1	12.29229	11.22422
P62873	GNB1	Guanine nu	37.377	340	2.095809	1.067507	+	2	19.25139	18.18389
Q12905	ILF2	Interleukin	43.062	390	2.09565	1.067398	+	2	18.17202	17.10462
P14868	DARS1	Aspartate--	57.136	501	2.095028	1.066969	+	2	15.60267	14.5357
Q6P1J9	CDC73	Parafibrom	60.577	531	2.094221	1.066413	+	1	12.21756	11.15115
Q93050	ATP6V0A1	V-type prot	96.413	837	2.093506	1.065921	+	1	12.03902	10.9731
P08754	GNAI3	Guanine nu	40.532	354	2.09314	1.065669	+	2	19.45545	18.38978
Q5T2T1	MPP7	MAGUK p5	65.524	576	2.092213	1.06503	+	1	12.06693	11.0019
P50991	CCT4	T-complex	57.924	539	2.085469	1.060372	+	2	16.05229	14.99192
Q8IU60	DCP2	m7GpppN-	48.423	420	2.083364	1.058915	+	1	12.2323	11.17339
Q96BH1	RNF25	E3 ubiquiti	51.219	459	2.082912	1.058602	+	2	15.67532	14.61672
Q9Y3D0	CIAO2B	Cytosolic ir	17.663	163	2.082103	1.058041	+	1	11.97499	10.91695
P21291	CSRP1	Cysteine a	20.567	193	2.077496	1.054846	+	2	17.47207	16.41723
Q6ZSJ8	C1orf122	Uncharacte	11.471	110	2.075453	1.053426	+	1	13.15217	12.09875
Q8N6T3	ARFGAP1	ADP-ribosy	44.668	406	2.069727	1.049441	+	1	12.55015	11.5007
Q96IZ0	PAWR	PRKC apop	36.568	340	2.069002	1.048935	+	2	17.94747	16.89853
O75937	DNAJC8	DnaJ homo	29.842	253	2.064389	1.045715	+	1	13.18305	12.13733
P47755	CAPZA2	F-actin-cap	32.949	286	2.06389	1.045366	+	2	19.62961	18.58424
P23378	GLDC	Glycine del	112.73	1020	2.063666	1.045209	+	1	11.90249	10.85728
Q92796	DLG3	Disks large	90.314	817	2.061961	1.044017	+	1	12.17149	11.12747
Q13362	PPP2R5C	Serine/thre	61.061	524	2.06145	1.043659	+	1	12.30175	11.25809
Q8IVT2	MISP	Mitotic inte	75.357	679	2.056402	1.040122	+	2	20.38974	19.34962
Q9BVL2	NUP58	Nucleopori	60.897	599	2.056279	1.040036	+	1	10.91931	9.879274
P41227	NAA10	N-alpha-ac	26.459	235	2.054052	1.038473	+	1	11.61393	10.57545
Q15018	ABRAXAS	BRISC con	46.901	415	2.052195	1.037167	+	1	11.86921	10.83204
Q92878	RAD50	DNA repair	153.892	1312	2.050974	1.036309	+	1	12.8008	11.76449
Q9BTT6	LRRC1	Leucine-ric	59.242	524	2.046785	1.03336	+	1	11.58548	10.55212
Q15843	NEDD8	NEDD8 OS	9.072	81	2.046132	1.032899	+	1	11.8892	10.8563
Q9ULV0	MYO5B	Unconventi	213.672	1848	2.045287	1.032303	+	1	10.09183	9.059529
Q5T8P6	RBM26	RNA-bindir	113.597	1007	2.044703	1.031891	+	1	12.11576	11.08387
Q8NC51	SERBP1	Plasminog	44.965	408	2.042426	1.030284	+	2	18.32474	17.29446
A6NI79	CCDC69	Coiled-coil	34.796	296	2.042351	1.030231	+	1	11.58909	10.55886
P00367	GLUD1	Glutamate	61.398	558	2.040937	1.029231	+	1	11.80397	10.77474
Q8IZ21	PHACTR4	Phosphata	78.211	702	2.04045	1.028888	+	1	11.77351	10.74462
Q9Y6U3	SCIN	Scinderin C	80.489	715	2.038821	1.027735	+	2	12.63363	11.6059
Q7Z6B7	SRGAP1	SLIT-ROB	124.264	1085	2.034872	1.024938	+	1	11.8689	10.84396

P31946	YWHAB	14-3-3 prot	28.082	246	2.034052	1.024357	+	2	16.06537	15.04102
P22102	GART	Trifunctiona	107.767	1010	2.033874	1.02423	+	1	13.3005	12.27627
Q8N5P1	ZC3H8	Zinc finger	33.576	291	2.032505	1.023259	+	1	12.66565	11.64239
Q63ZY3	KANK2	KN motif ai	91.174	851	2.031034	1.022215	+	1	12.03782	11.01561
Q96GM8	TOE1	Target of E	56.548	510	2.02982	1.021352	+	1	11.68014	10.65879
Q99439	CNN2	Calponin-2	33.697	309	2.027703	1.019846	+	1	13.18681	12.16696
Q7Z4Q2	HEATR3	HEAT repe	74.583	680	2.026133	1.018729	+	1	11.56043	10.5417
P42285	MTREX	Exosome F	117.805	1042	2.020815	1.014937	+	1	13.28959	12.27465
Q9H9T3	ELP3	Elongator c	62.259	547	2.020666	1.014831	+	1	12.89201	11.87718
Q12965	MYO1E	Unconventi	127.062	1108	2.01581	1.01136	+	2	18.79201	17.78066
Q6P2Q9	PRPF8	Pre-mRNA	273.6	2335	2.007122	1.005128	+	2	16.93307	15.92794
P68371	TUBB4B	Tubulin bet	49.831	445	2.006371	1.004588	+	2	19.70826	18.70367
P68366	TUBA4A	Tubulin alp	49.924	448	2.004796	1.003455	+	2	16.90713	15.90367
Q9BYD1	MRPL13	39S riboso	20.692	178	2.004247	1.00306	+	1	12.41148	11.40842
O14933	UBE2L6	Ubiquitin/IS	17.769	153	2.002049	1.001477	+	1	12.47879	11.47732
Q8TB72	PUM2	Pumilio hor	114.216	1066	2.001376	1.000992	+	1	11.8983	10.89731
Q8NBJ4	GOLM1	Golgi mem	45.333	401	1.999986	0.99999	+	1	11.59703	10.59704
O94832	MYO1D	Unconventi	116.202	1006	1.996947	0.997796	+	1	11.27158	10.27378
P21589	NT5E	5'-nucleotic	63.368	574	1.993977	0.995649	+	2	19.69285	18.69721
Q13033	STRN3	Striatin-3 C	87.209	797	1.990818	0.993361	+	1	12.09681	11.10345
P15941	MUC1	Mucin-1 OS	122.102	1255	1.989832	0.992647	+	1	12.53559	11.54294
O14744	PRMT5	Protein arg	72.684	637	1.98972	0.992565	+	2	15.44828	14.45571
Q8N8Y2	ATP6V0D2	V-type prot	40.426	350	1.988032	0.991341	+	2	14.42036	13.42901
P45880	VDAC2	Voltage-de	31.567	294	1.984461	0.988747	+	2	15.64417	14.65542
P10586	PTPRF	Receptor-ty	212.879	1907	1.984012	0.988421	+	1	11.50646	10.51804
O15091	PRORP	Mitochondr	67.315	583	1.981311	0.986455	+	1	11.73763	10.75117
Q15370	ELOB	Elongin-B (	13.133	118	1.972907	0.980323	+	2	15.15782	14.1775
O95487	SEC24B	Protein trar	137.418	1268	1.97282	0.980259	+	2	12.34346	11.3632
P60891	PRPS1	Ribose-pho	34.834	318	1.968445	0.977056	+	2	14.05155	13.07449
Q7L5D6	GET4	Golgi to ER	36.504	327	1.968157	0.976845	+	1	11.56114	10.5843
Q8WWY3	PRPF31	U4/U6 sma	55.456	499	1.967816	0.976596	+	1	10.979	10.0024
Q53TN4	CYBRD1	Plasma me	31.641	286	1.967557	0.976406	+	2	16.80348	15.82707
O95202	LETM1	Mitochondr	83.354	739	1.965847	0.975151	+	1	11.28013	10.30498
Q13188	STK3	Serine/thre	56.301	491	1.96471	0.974316	+	1	13.45866	12.48435
Q9BXW7	HDHD5	Haloacid de	46.321	423	1.964499	0.974161	+	1	11.59777	10.62361
P00533	EGFR	Epidermal	134.277	1210	1.962747	0.972874	+	2	13.85584	12.88297
Q15276	RABEP1	Rab GTPas	99.29	862	1.96212	0.972413	+	1	11.24174	10.26933
P25054	APC	Adenomato	311.646	2843	1.961753	0.972143	+	1	11.9709	10.99875
P63000	RAC1	Ras-relatec	21.45	192	1.959943	0.970811	+	2	17.98437	17.01356
Q8NBS9	TXNDC5	Thioredoxi	47.629	432	1.956609	0.968355	+	1	12.59355	11.6252
Q7L592	NDUFAF7	Protein arg	49.238	441	1.956478	0.968259	+	1	11.37346	10.4052
P53582	METAP1	Methionine	43.215	386	1.956472	0.968254	+	1	11.22966	10.26141
Q15650	TRIP4	Activating s	66.146	581	1.956091	0.967974	+	1	12.96728	11.99931
P30043	BLVRB	Flavin redu	22.119	206	1.953268	0.96589	+	1	11.83036	10.86447
Q6I9Y2	THOC7	THO comp	23.743	204	1.953221	0.965855	+	1	12.05117	11.08532
P42167	TMPO	Lamina-ass	50.67	454	1.95267	0.965448	+	2	17.63098	16.66553
Q12802	AKAP13	A-kinase ar	307.55	2813	1.952352	0.965213	+	1	11.7932	10.82798
Q7L5Y9	MAEA	E3 ubiquitir	45.287	396	1.951104	0.964291	+	1	10.78259	9.818298
P54105	CLNS1A	Methylosor	26.215	237	1.949431	0.963053	+	2	15.36273	14.39968
P47712	PLA2G4A	Cytosolic p	85.239	749	1.949007	0.962739	+	1	12.2927	11.32996
Q96C57	CUSTOS	Protein CU	28.171	262	1.948821	0.962602	+	1	12.90597	11.94337
Q9Y3X0	CCDC9	Coiled-coil	59.703	531	1.946717	0.961043	+	1	12.24231	11.28127
Q12792	TWF1	Twinfilin-1	40.283	350	1.944501	0.9594	+	2	19.30837	18.34897
P62834	RAP1A	Ras-relatec	20.987	184	1.943874	0.958935	+	2	17.95373	16.9948
Q86W56	PARG	Poly(ADP-r	111.11	976	1.943625	0.95875	+	1	11.77713	10.81838
P35237	SERPINB6	Serpin B6 (	42.622	376	1.943369	0.95856	+	2	15.29311	14.33455
Q96HR8	NAF1	H/ACA ribo	53.717	494	1.942358	0.957809	+	1	12.78611	11.8283
Q8IWC1	MAP7D3	MAP7 dom	98.429	876	1.940702	0.956578	+	1	12.51039	11.55382
Q13418	ILK	Integrin-linl	51.419	452	1.939538	0.955713	+	1	11.31509	10.35938

Q9HCN4	GPN1	GPN-loop (	41.74	374	1.936351	0.95334	+	1	11.50189	10.54855
Q9Y3Z3	SAMHD1	Deoxynucle	72.201	626	1.936134	0.953179	+	1	12.43786	11.48468
Q08211	DHX9	ATP-deper	140.958	1270	1.93558	0.952766	+	2	19.35063	18.39787
Q9P0J0	NDUFA13	NADH deh	16.698	144	1.935276	0.95254	+	1	12.41785	11.46531
P31327	CPS1	Carbamoyl	164.939	1500	1.933333	0.95109	+	2	18.4373	17.48621
Q9H3R2	MUC13	Mucin-13 C	54.604	512	1.933095	0.950913	+	2	19.35574	18.40483
Q8WUM0	NUP133	Nuclear po	128.979	1156	1.931085	0.949411	+	1	11.46913	10.51972
Q99729	HNRNPAB	Heterogene	36.225	332	1.930632	0.949074	+	2	17.5414	16.59233
Q14151	SAFB2	Scaffold at	107.473	953	1.925222	0.945025	+	2	14.35693	13.41191
Q9NZW5	PALS2	Protein PA	61.117	540	1.914565	0.937017	+	1	10.81714	9.880127
P14209	CD99	CD99 antig	18.848	185	1.914218	0.936755	+	1	10.93443	9.997673
P25205	MCM3	DNA replic	90.981	808	1.912579	0.935519	+	2	16.48654	15.55102
P31689	DNAJA1	DnaJ homo	44.868	397	1.911552	0.934745	+	2	16.98549	16.05074
P40937	RFC5	Replication	38.497	340	1.906574	0.930983	+	2	13.01137	12.08038
Q9Y3D9	MRPS23	28S riboso	21.771	190	1.90456	0.929458	+	2	15.63444	14.70498
Q13227	GPS2	G protein p	36.689	327	1.903053	0.928315	+	1	12.27044	11.34213
Q9H2W6	MRPL46	39S riboso	31.705	279	1.902908	0.928206	+	2	13.59968	12.67147
P49368	CCT3	T-complex	60.534	545	1.899969	0.925976	+	2	15.88362	14.95765
P52655	GTF2A1	Transcripti	41.514	376	1.899198	0.92539	+	1	12.28338	11.35799
Q9Y3U8	RPL36	60S riboso	12.254	105	1.898671	0.92499	+	2	16.78915	15.86416
P82664	MRPS10	28S riboso	22.999	201	1.897204	0.923875	+	2	12.89951	11.97563
O95831	AIFM1	Apoptosis-i	66.901	613	1.891788	0.919751	+	2	15.01276	14.093
O95394	PGM3	Phosphoac	59.852	542	1.890841	0.919028	+	1	11.29737	10.37835
P61026	RAB10	Ras-relatec	22.541	200	1.890263	0.918587	+	2	13.72888	12.81029
Q70UQ0	IKBIP	Inhibitor of	39.309	350	1.889478	0.917988	+	2	13.78453	12.86654
Q9Y6M7	SLC4A7	Sodium bic	136.044	1214	1.887842	0.916738	+	1	11.23978	10.32304
Q9BRD0	BUD13	BUD13 hor	70.521	619	1.887166	0.916222	+	2	13	12.08378
Q86YP4	GATAD2A	Transcripti	68.063	633	1.886475	0.915693	+	1	12.33893	11.42324
Q9P287	BCCIP	BRCA2 and	35.979	314	1.886184	0.915471	+	1	12.48165	11.56618
O43617	TRAPPC3	Trafficking	20.274	180	1.885713	0.91511	+	1	12.66447	11.74936
P62136	PPP1CA	Serine/thre	37.512	330	1.884959	0.914533	+	2	19.55377	18.63923
Q92900	UPF1	Regulator o	124.345	1129	1.88244	0.912604	+	2	16.17356	15.26096
Q14232	EIF2B1	Translation	33.712	305	1.881892	0.912184	+	1	11.80973	10.89754
P61201	COPS2	COP9 sign	51.597	443	1.881887	0.91218	+	1	11.96261	11.05043
Q8WWI1	LMO7	LIM domai	192.696	1683	1.873675	0.905871	+	2	19.70344	18.79757
Q9UQR0	SCML2	Sex comb o	77.257	700	1.871765	0.904399	+	1	10.67587	9.77147
P52594	AGFG1	Arf-GAP do	58.26	562	1.871458	0.904163	+	1	11.42432	10.52016
Q8WUW1	BRK1	Protein BR	8.745	75	1.866863	0.900616	+	2	14.59345	13.69283
Q15050	RRS1	Ribosome	41.193	365	1.862122	0.896947	+	2	14.41904	13.52209
P49411	TUFM	Elongation	49.542	452	1.862112	0.89694	+	2	16.8652	15.96826
Q9NXF1	TEX10	Testis-expr	105.674	929	1.860974	0.896058	+	1	11.55995	10.66389
P32119	PRDX2	Peroxiredo	21.892	198	1.860595	0.895764	+	2	18.22147	17.32571
Q13151	HNRNPA0	Heterogene	30.841	305	1.859157	0.894649	+	2	18.42	17.52535
Q9ULA0	DNPEP	Aspartyl an	53.41	485	1.857205	0.893133	+	1	12.12244	11.22931
Q562R1	ACTBL2	Beta-actin-	42.003	376	1.854957	0.891386	+	2	17.30733	16.41594
Q9Y312	AAR2	Protein AA	43.472	384	1.854802	0.891265	+	1	10.96225	10.07098
P29317	EPHA2	Ephrin type	108.266	976	1.854008	0.890648	+	2	15.25259	14.36194
Q9NUQ8	ABCF3	ATP-bindin	79.745	709	1.85198	0.889068	+	1	12.75752	11.86845
Q9H6Z9	EGLN3	Prolyl hydro	27.261	239	1.84621	0.884566	+	2	14.89728	14.01271
Q9BUP0	EFHD1	EF-hand do	26.928	239	1.844352	0.883114	+	2	16.63183	15.74872
Q9BPZ7	MAPKAP1	Target of ra	59.123	522	1.843889	0.882752	+	1	11.17255	10.2898
Q96PC5	MIA2	Melanoma	159.836	1412	1.833507	0.874606	+	1	11.08062	10.20601
P55036	PSMD4	26S protea	40.737	377	1.833381	0.874507	+	2	15.9704	15.09589
Q9BW66	CINP	Cyclin-depe	24.324	212	1.832388	0.873725	+	1	11.02348	10.14975
Q9GZT3	SLIRP	SRA stem-	12.349	109	1.829748	0.871645	+	2	16.27139	15.39975
P0DPB6	POLR1D	DNA-direct	15.237	133	1.829137	0.871163	+	2	14.57507	13.7039
O75955	FLOT1	Flotillin-1 C	47.355	427	1.823078	0.866377	+	2	18.6245	17.75812
Q8IZQ5	SELENOH	Selenoprot	13.453	122	1.821451	0.865088	+	1	10.93391	10.06882
Q9BSE5	AGMAT	Agmatinase	37.66	352	1.820508	0.864341	+	1	12.00359	11.13925

Q9UHB6	LIMA1	LIM domai	85.226	759	1.820191	0.86409	+	2	21.71175	20.84766
O95218	ZRANB2	Zinc finger	37.404	330	1.819848	0.863818	+	1	12.65439	11.79057
Q92888	ARHGEF1	Rho guanir	102.435	912	1.818099	0.862431	+	1	11.9705	11.10807
P32780	GTF2H1	General tra	62.032	548	1.816535	0.861189	+	1	11.02022	10.15903
Q9H6Q4	CIAO3	Cytosolic ir	53.02	476	1.812676	0.858121	+	1	12.20836	11.35024
Q7Z2W4	ZC3HAV1	Zinc finger	101.431	902	1.810879	0.85669	+	2	17.43298	16.57629
Q9BT92	TCHP	Trichoplein	61.072	498	1.806811	0.853445	+	1	11.13128	10.27783
Q07820	MCL1	Induced my	37.337	350	1.805668	0.852533	+	1	11.46337	10.61084
Q9H7Z7	PTGES2	Prostaglan	41.943	377	1.80468	0.851743	+	1	11.06184	10.2101
Q9BU61	NDUFAF3	NADH deh	20.35	184	1.80269	0.850151	+	1	11.96275	11.1126
Q5MNZ6	WDR45B	WD repeat	38.122	344	1.798761	0.847003	+	1	12.38872	11.54171
C9JLW8	MCRIP1	Mapk-regu	10.92	97	1.797455	0.845955	+	1	12.17305	11.3271
P09622	DLD	Dihydrolipo	54.177	509	1.79256	0.842021	+	2	17.69057	16.84855
Q86U44	METTL3	N6-adenos	64.474	580	1.789271	0.839372	+	1	10.77536	9.935992
Q96E14	RMI2	RecQ-med	15.865	147	1.78874	0.838944	+	1	11.87341	11.03446
Q15003	NCAPH	Condensin	82.563	741	1.782834	0.834172	+	1	12.12464	11.29047
Q14141	SEPTIN6	Septin-6 O	49.717	434	1.781418	0.833026	+	1	13.12729	12.29426
P78368	CSNK1G2	Casein kin	47.457	415	1.779294	0.831305	+	1	12.17814	11.34683
Q6ZU65	UBN2	Ubinuclein-	146.089	1347	1.777302	0.829689	+	1	13.32671	12.49702
P62937	PPIA	Peptidyl-pr	18.012	165	1.776863	0.829332	+	2	17.82997	17.00064
P51148	RAB5C	Ras-relate	23.483	216	1.77622	0.82881	+	2	14.54756	13.71875
Q96FW1	OTUB1	Ubiquitin th	31.284	271	1.774998	0.827818	+	1	12.21659	11.38878
Q92759	GTF2H4	General tra	52.186	462	1.773867	0.826898	+	1	12.6795	11.8526
Q13561	DCTN2	Dynactin su	44.231	401	1.77062	0.824254	+	2	17.12928	16.30503
Q9BZM5	ULBP2	UL16-bindi	27.368	246	1.770157	0.823877	+	2	18.15887	17.33499
P48382	RFX5	DNA-bindir	65.323	616	1.768312	0.822373	+	1	12.66636	11.84398
Q14103	HNRNPD	Heterogene	38.434	355	1.762684	0.817774	+	2	18.72707	17.9093
P52907	CAPZA1	F-actin-cap	32.923	286	1.758519	0.814361	+	2	20.29221	19.47785
O95163	ELP1	Elongator c	150.254	1332	1.757221	0.813296	+	1	12.05056	11.23727
Q8WVY7	UBLCP1	Ubiquitin-lil	36.805	318	1.756388	0.812612	+	1	11.15406	10.34145
Q9H1Y0	ATG5	Autophagy	32.447	275	1.755116	0.811566	+	1	11.11048	10.29892
O00186	STXBP3	Syntaxin-bi	67.764	592	1.748463	0.806087	+	1	11.25391	10.44782
Q13045	FLII	Protein figl	144.751	1269	1.747657	0.805422	+	2	17.77166	16.96624
Q9NPH2	ISYNA1	Inositol-3-p	61.068	558	1.743957	0.802364	+	1	13.00662	12.20426
Q69YQ0	SPECC1L	Cytospin-A	124.544	1117	1.743413	0.801914	+	2	18.17412	17.3722
P27144	AK4	Adenylate l	25.268	223	1.742456	0.801122	+	1	12.23924	11.43812
Q8N766	EMC1	ER membr	111.759	993	1.739758	0.798887	+	1	11.67992	10.88103
Q13724	MOGS	Mannosyl-c	91.918	837	1.738846	0.79813	+	2	14.57672	13.77859
P09382	LGALS1	Galectin-1	14.716	135	1.738027	0.797451	+	2	18.12369	17.32624
Q9BRP1	PDCD2L	Programm	39.417	358	1.733635	0.7938	+	1	12.86564	12.07184
P52735	VAV2	Guanine nu	101.289	878	1.732342	0.792724	+	1	11.47857	10.68584
Q9ULZ9	MMP17	Matrix met	66.653	603	1.731015	0.791618	+	1	11.28627	10.49465
Q10589	BST2	Bone marr	19.769	180	1.726348	0.787723	+	2	18.42968	17.64195
Q13242	SRSF9	Serine/argi	25.542	221	1.725627	0.787121	+	2	16.66484	15.87772
Q13501	SQSTM1	Sequestos	47.687	440	1.725173	0.786741	+	2	15.96459	15.17785
P14174	MIF	Macrophag	12.476	115	1.722921	0.784857	+	1	11.67375	10.88889
P36578	RPL4	60S riboso	47.697	427	1.720652	0.782956	+	2	17.3376	16.55465
Q12824	SMARCB1	SWI/SNF-r	44.141	385	1.720517	0.782842	+	1	12.65137	11.86852
P55084	HADHB	Trifunction	51.294	474	1.718449	0.781107	+	2	18.48903	17.70793
Q13405	MRPL49	39S riboso	19.198	166	1.71807	0.780789	+	2	15.03862	14.25783
Q9ULH0	KIDINS220	Kinase D-ir	196.542	1771	1.715022	0.778227	+	1	11.96322	11.18499
Q8N3U4	STAG2	Cohesin su	141.326	1231	1.711199	0.775007	+	1	12.11179	11.33678
Q86UY6	NAA40	N-alpha-ac	27.194	237	1.710484	0.774404	+	1	11.92203	11.14762
Q96S59	RANBP9	Ran-bindin	77.847	729	1.708989	0.773143	+	1	12.12522	11.35208
Q9BV57	ADI1	1,2-dihydrc	21.498	179	1.70302	0.768096	+	1	11.20683	10.43874
Q92747	ARPC1A	Actin-relate	41.569	370	1.697829	0.763691	+	2	14.40315	13.63945
O15392	BIRC5	Baculoviral	16.389	142	1.694913	0.761211	+	1	11.70183	10.94062
P21283	ATP6V1C1	V-type prot	43.942	382	1.69172	0.758491	+	1	13.02915	12.27066
P01111	NRAS	GTPase NI	21.229	189	1.69156	0.758355	+	1	11.75309	10.99474

Q7Z5K2	WAPL	Wings apa	132.946	1190	1.688976	0.756149	+	1	10.72067	9.964523
Q96NC0	ZMAT2	Zinc finger	23.612	199	1.687311	0.754726	+	1	11.86457	11.10985
O95747	OXSR1	Serine/thre	58.022	527	1.686713	0.754214	+	1	11.57903	10.82482
Q15020	SART3	Squamous	109.935	963	1.685807	0.75344	+	1	11.85335	11.09991
P57740	NUP107	Nuclear po	106.374	925	1.685162	0.752888	+	2	18.43388	17.68099
Q12906	ILF3	Interleukin	95.338	894	1.682414	0.750533	+	2	18.05367	17.30314
Q86X53	ERICH1	Glutamate-	48.984	443	1.681518	0.749764	+	1	11.18277	10.433
Q13131	PRKAA1	5'-AMP-act	64.009	559	1.681338	0.749609	+	1	11.14396	10.39435
P36406	TRIM23	E3 ubiquitin	64.067	574	1.679379	0.747927	+	2	15.34482	14.59689
P07237	P4HB	Protein disu	57.116	508	1.678534	0.747202	+	2	15.77342	15.02622
Q8N983	MRPL43	39S riboso	23.431	215	1.677923	0.746677	+	1	9.972118	9.225441
P17987	TCP1	T-complex	60.344	556	1.677309	0.746148	+	2	16.42522	15.67907
P63279	UBE2I	SUMO-con	18.007	158	1.67173	0.741341	+	2	14.83748	14.09614
Q92734	TFG	Protein TF(	43.448	400	1.669905	0.739766	+	1	12.26368	11.52391
Q9NZT1	CALML5	Calmodulin	15.893	146	1.669695	0.739585	+	1	10.68965	9.950064
Q13619	CUL4A	Cullin-4A C	87.68	759	1.668374	0.738442	+	1	12.33996	11.60152
Q9UPN7	PPP6R1	Serine/thre	96.724	881	1.662387	0.733256	+	1	11.47518	10.74192
Q8IX18	DHX40	Probable A	88.56	779	1.661391	0.732392	+	1	10.95739	10.225
P07355	ANXA2	Annexin A2	38.604	339	1.659953	0.731142	+	2	20.08018	19.34903
P0DMV9	HSPA1B	Heat shock	70.052	641	1.659902	0.731098	+	2	19.9238	19.1927
Q92932	PTPRN2	Receptor-ty	111.271	1015	1.656592	0.728218	+	2	16.13943	15.41121
Q9H8H2	DDX31	Probable A	94.087	851	1.656389	0.728041	+	1	11.22545	10.49741
P26038	MSN	Moesin OS	67.82	577	1.654535	0.726426	+	2	17.86194	17.13551
Q15528	MED22	Mediator of	22.221	200	1.649607	0.722123	+	1	11.35849	10.63636
Q99615	DNAJC7	DnaJ homo	56.441	494	1.644161	0.717351	+	2	14.88555	14.1682
O00571	DDX3X	ATP-deper	73.243	662	1.639933	0.713637	+	2	18.00333	17.28969
Q13148	TARDBP	TAR DNA-I	44.74	414	1.638952	0.712774	+	2	14.18673	13.47396
P31930	UQCRC1	Cytochrom	52.646	480	1.638299	0.712199	+	1	12.06814	11.35594
Q13283	G3BP1	Ras GTPas	52.164	466	1.629669	0.704579	+	2	17.90619	17.20161
Q13505	MTX1	Metaxin-1 (	51.463	466	1.629468	0.704401	+	1	11.58998	10.88558
Q96C19	EFHD2	EF-hand do	26.697	240	1.629116	0.70409	+	2	18.29697	17.59288
P29692	EEF1D	Elongation	31.122	281	1.622248	0.697995	+	2	17.55337	16.85538
P53675	CLTCL1	Clathrin he	187.03	1640	1.621287	0.697139	+	2	16.42748	15.73034
P04075	ALDOA	Fructose-b	39.42	364	1.621164	0.69703	+	2	16.15681	15.45978
Q9HBI1	PARVB	Beta-parvin	41.714	364	1.618392	0.694561	+	1	12.21769	11.52312
Q13421	MSLN	Mesothelin	68.986	630	1.61368	0.690355	+	2	17.87927	17.18892
Q8N1G2	CMTR1	Cap-specif	95.321	835	1.612906	0.689662	+	1	12.12454	11.43488
Q8NBT2	SPC24	Kinetochor	22.443	197	1.612218	0.689047	+	1	10.62954	9.940492
P15880	RPS2	40S riboso	31.324	293	1.612196	0.689027	+	2	18.17704	17.48801
Q8NC56	LEMD2	LEM doma	56.975	503	1.611425	0.688337	+	2	15.74294	15.0546
Q9Y606	PUS1	tRNA pseu	47.47	427	1.606358	0.683793	+	1	11.79831	11.11452
P18085	ARF4	ADP-ribosy	20.511	180	1.60477	0.682366	+	2	13.7462	13.06383
P42166	TMPO	Lamina-ass	75.492	694	1.603687	0.681392	+	2	18.96694	18.28555
O14684	PTGES	Prostaglan	17.102	152	1.601592	0.679506	+	1	10.79555	10.11605
Q13416	ORC2	Origin reco	65.972	577	1.601333	0.679273	+	1	11.62726	10.94799
P16455	MGMT	Methylated	21.646	207	1.600491	0.678515	+	1	10.80308	10.12457
Q53H96	PYCR3	Pyrroline-5	28.663	274	1.60038	0.678414	+	1	11.27315	10.59474
Q9HA64	FN3KRP	Ketosamin	34.412	309	1.599916	0.677996	+	1	12.04743	11.36943
Q7Z4S6	KIF21A	Kinesin-like	187.179	1674	1.599806	0.677897	+	1	12.12221	11.44432
P28066	PSMA5	Proteasom	26.411	241	1.59956	0.677675	+	2	15.36516	14.68749
P56385	ATP5ME	ATP syntha	7.933	69	1.596277	0.674711	+	2	14.09441	13.4197
Q9H1B7	IRF2BPL	Probable E	82.659	796	1.595846	0.674321	+	1	11.42548	10.75116
Q6WCQ1	MPRIP	Myosin pho	116.533	1025	1.594135	0.672774	+	2	18.46587	17.7931
Q96PU5	NEDD4L	E3 ubiquitin	111.932	975	1.590616	0.669585	+	1	11.38225	10.71266
O00401	WASL	Neural Wis	54.827	505	1.5902	0.669208	+	1	11.32716	10.65795
Q13557	CAMK2D	Calcium/ca	56.369	499	1.589658	0.668717	+	2	14.56968	13.90096
P53680	AP2S1	AP-2 comp	17.018	142	1.589033	0.668149	+	2	18.70239	18.03424
Q13451	FKBP5	Peptidyl-pro	51.212	457	1.588118	0.667318	+	1	11.87314	11.20582
A6NDB9	PALM3	Paralemmi	71.695	673	1.586425	0.66578	+	2	14.59799	13.93221

Q8NBZ0	INO80E	INO80 com	26.478	244	1.576031	0.656296	+	1	11.04842	10.39212
Q9Y6K9	IKBKG	NF-kappa-I	48.198	419	1.573958	0.654397	+	2	15.63645	14.98206
P16333	NCK1	Cytoplasmi	42.864	377	1.571762	0.652383	+	1	12.80347	12.15108
P62699	YPEL5	Protein yip	13.842	121	1.570062	0.650822	+	1	12.36309	11.71227
Q13228	SELENBP1	Methanethi	52.391	472	1.567377	0.648352	+	1	11.70619	11.05784
P38646	HSPA9	Stress-70 p	73.681	679	1.566539	0.647581	+	2	18.9533	18.30572
Q96II8	LRCH3	DISP comp	86.083	777	1.564641	0.645832	+	2	14.42298	13.77715
Q6IAA8	LAMTOR1	Ragulator c	17.745	161	1.564178	0.645405	+	2	15.81846	15.17305
Q02218	OGDH	2-oxoglutar	115.935	1023	1.563658	0.644925	+	2	18.51826	17.87334
Q96KG9	SCYL1	N-terminal	89.631	808	1.561619	0.643043	+	1	12.95163	12.30859
Q96HH9	GRAMD2B	GRAM don	47.869	432	1.559308	0.640906	+	1	11.13648	10.49557
Q53GA4	PHLDA2	Pleckstrin h	17.092	152	1.554895	0.636818	+	1	12.28892	11.65211
Q5H9R7	PPP6R3	Serine/thre	97.669	873	1.554179	0.636153	+	2	12.51328	11.87713
P42677	RPS27	40S riboso	9.461	84	1.551557	0.633717	+	2	17.16644	16.53272
Q03405	PLAUR	Urokinase	36.978	335	1.54971	0.631998	+	2	17.11776	16.48577
P19525	EIF2AK2	Interferon-i	62.094	551	1.548771	0.631124	+	2	15.71859	15.08746
P06493	CDK1	Cyclin-depe	34.095	297	1.542697	0.625454	+	2	16.69262	16.06716
P42696	RBM34	RNA-bindir	48.565	430	1.542604	0.625368	+	1	11.20071	10.57535
Q6P1K2	PMF1	Polyamine-	23.339	205	1.542544	0.625312	+	1	11.31826	10.69295
P35052	GPC1	Glypican-1	61.68	558	1.539467	0.622431	+	2	18.90965	18.28722
P23229	ITGA6	Integrin alp	126.606	1130	1.53217	0.615576	+	1	10.88836	10.27279
P52597	HNRNPF	Heterogene	45.672	415	1.529931	0.613467	+	2	18.6656	18.05213
Q13084	MRPL28	39S riboso	30.157	256	1.529654	0.613206	+	1	12.17049	11.55728
Q96G21	IMP4	U3 small nu	33.757	291	1.529251	0.612825	+	2	14.16969	13.55687
Q9NUL7	DDX28	Probable A	59.581	540	1.529097	0.612679	+	2	14.78581	14.17313
P12268	IMPDH2	Inosine-5'-r	55.805	514	1.52882	0.612419	+	2	17.96251	17.35009
Q13085	ACACA	Acetyl-CoA	265.554	2346	1.526694	0.610411	+	1	11.41754	10.80713
Q8NCF5	NFATC2IP	NFATC2-ir	45.817	419	1.525936	0.609694	+	1	11.98517	11.37547
P49589	CARS1	Cysteine--t	85.473	748	1.525689	0.609461	+	1	11.40237	10.79291
P54760	EPHB4	Ephrin type	108.27	987	1.524466	0.608304	+	1	12.1473	11.539
P20042	EIF2S2	Eukaryotic	38.388	333	1.523268	0.607169	+	2	15.76826	15.16109
Q9H5Q4	TFB2M	Dimethylad	45.349	396	1.51983	0.60391	+	1	11.73737	11.13346
Q00325	SLC25A3	Phosphate	40.095	362	1.518691	0.602828	+	2	15.54034	14.93751
P60981	DSTN	Destrin OS	18.506	165	1.518338	0.602493	+	2	15.69566	15.09317
Q9ULP9	TBC1D24	TBC1 dom	62.92	559	1.515779	0.600059	+	1	11.92807	11.32801
Q8N6R0	METTL13	eEF1A lysi	78.768	699	1.513578	0.597963	+	1	13.12595	12.52798
O43164	PJA2	E3 ubiquitir	78.214	708	1.513519	0.597907	+	1	12.2416	11.64369
P62820	RAB1A	Ras-relatec	22.678	205	1.512064	0.596519	+	1	11.54689	10.95037
Q02878	RPL6	60S riboso	32.728	288	1.511393	0.595879	+	2	17.2744	16.67852
P04083	ANXA1	Annexin A1	38.714	346	1.510816	0.595328	+	1	11.7719	11.17657
O14519	CDK2AP1	Cyclin-depe	12.365	115	1.509914	0.594466	+	1	11.46781	10.87334
O95391	SLU7	Pre-mRNA	68.387	586	1.509548	0.594116	+	2	13.23429	12.64018
Q9NXG2	THUMPD1	THUMP dc	39.315	353	1.508586	0.593197	+	1	11.25609	10.66289
P14625	HSP90B1	Endoplasm	92.469	803	1.508271	0.592895	+	2	16.19368	15.60078
Q9NWQ8	PAG1	Phosphopr	46.981	432	1.508191	0.592819	+	1	12.75035	12.15753
O60293	ZFC3H1	Zinc finger	226.356	1989	1.506872	0.591557	+	1	11.27514	10.68358
Q9HCM4	EPB41L5	Band 4.1-lil	81.856	733	1.506696	0.591388	+	2	16.57374	15.98235
P23526	AHCY	Adenosylhc	47.716	432	1.504708	0.589484	+	2	14.42869	13.8392
O95292	VAPB	Vesicle-ass	27.228	243	1.500829	0.58576	+	2	17.31336	16.7276
Q9UN86	G3BP2	Ras GTPas	54.121	482	1.495874	0.580989		2	17.68202	17.10103
Q86XK3	SFR1	Swi5-deper	28.262	245	1.494765	0.579918		1	10.4581	9.878181
Q9BSH4	TACO1	Translation	32.477	297	1.493857	0.579042		1	11.63027	11.05123
P49736	MCM2	DNA replic	101.896	904	1.493731	0.57892		2	15.39225	14.81333
Q9Y3B3	TMED7	Transmem	25.172	224	1.491048	0.576327		1	10.4418	9.865476
P49902	NT5C2	Cytosolic p	64.97	561	1.490032	0.575343		1	11.78664	11.21129
P08670	VIM	Vimentin O	53.652	466	1.484773	0.570242		2	21.11242	20.54217
Q6P3R8	NEK5	Serine/thre	81.445	708	1.483077	0.568593		2	17.99937	17.43078
Q16537	PPP2R5E	Serine/thre	54.699	467	1.479695	0.565299		1	11.11218	10.54688
Q86X29	LSR	Lipolysis-st	71.439	649	1.476119	0.561809		1	12.84772	12.28591

P10809	HSPD1	60 kDa hea	61.055	573	1.468123	0.553972	2	17.54766	16.99369
Q86SQ4	ADGRG6	Adhesion (	136.695	1221	1.467756	0.553612	1	12.09391	11.5403
P27708	CAD	CAD protei	242.984	2225	1.467687	0.553544	2	15.7129	15.15936
P37198	NUP62	Nuclear po	53.255	522	1.467523	0.553383	2	14.44327	13.88988
Q2TBE0	CWF19L2	CWF19-like	103.787	894	1.467349	0.553212	1	10.75338	10.20017
Q13555	CAMK2G	Calcium/ca	62.607	558	1.46663	0.552505	2	15.19676	14.64425
Q9Y4B6	DCAF1	DDB1- and	169.007	1507	1.464315	0.550226	1	11.79129	11.24106
Q01968	OCRL	Inositol pol	104.205	901	1.463757	0.549676	1	10.97692	10.42725
P43307	SSR1	Translocon	32.235	286	1.46359	0.549511	2	16.22536	15.67585
Q92841	DDX17	Probable A	80.272	729	1.463532	0.549454	2	19.3607	18.81124
P07437	TUBB	Tubulin bet	49.671	444	1.453275	0.539308	2	19.79364	19.25433
P02545	LMNA	Prelamin-A	74.139	664	1.452201	0.538241	2	19.32184	18.7836
Q9BPX5	ARPC5L	Actin-relate	16.941	153	1.451294	0.53734	2	16.62799	16.09065
Q8IZP0	ABI1	Abl interact	55.081	508	1.448007	0.534068	2	14.8392	14.30514
Q5GLZ8	HERC4	Probable E	118.563	1057	1.446657	0.532723	1	11.12703	10.5943
P16070	CD44	CD44 antig	81.538	742	1.446654	0.53272	2	17.46683	16.93411
P63104	YWHAZ	14-3-3 prot	27.745	245	1.443492	0.529564	2	17.63715	17.10758
Q05209	PTPN12	Tyrosine-pr	88.106	780	1.441181	0.527251	1	11.12186	10.59461
P15559	NQO1	NAD(P)H c	30.868	274	1.440505	0.526575	1	10.91685	10.39027
P27348	YWHAQ	14-3-3 prot	27.764	245	1.439225	0.525292	2	17.76104	17.23575
O95372	LYPLA2	Acyl-protein	24.737	231	1.439163	0.52523	1	12.34074	11.81551
Q8IY17	PNPLA6	Patatin-like	150.954	1375	1.438336	0.524401	1	11.7023	11.1779
O43464	HTRA2	Serine prot	48.841	458	1.435364	0.521417	1	10.76769	10.24627
Q53S33	BOLA3	BolA-like p	12.114	107	1.432185	0.518218	1	11.82177	11.30356
Q9NX24	NHP2	H/ACA ribo	17.201	153	1.427747	0.513741	2	14.93981	14.42607
O00257	CBX4	E3 SUMO-	61.368	560	1.42595	0.511923	1	10.78185	10.26993
Q15386	UBE3C	Ubiquitin-pr	123.923	1083	1.422602	0.508532	2	16.91917	16.41063
Q9UJ41	RABGEF1	Rab5 GDP	56.891	491	1.421933	0.507853	1	11.96967	11.46182
Q5VTR2	RNF20	E3 ubiquitin	113.662	975	1.41937	0.50525	1	11.44165	10.9364
Q92905	COPS5	COP9 sign	37.579	334	1.417382	0.503228	2	11.73978	11.23655
O60506	SYNCRIP	Heterogene	69.603	623	1.416125	0.501949	2	18.81127	18.30933
Q15366	PCBP2	Poly(rC)-bi	38.58	365	1.41482	0.500619	2	17.41138	16.91076
Q8TF64	GIPC3	PDZ doma	33.982	312	1.412094	0.497836	1	11.67772	11.17988
P11021	HSPA5	Endoplasm	72.333	654	1.408532	0.494193	2	20.09197	19.59778
P30622	CLIP1	CAP-Gly do	162.246	1438	1.406563	0.492174	1	12.10266	11.61049
Q76L83	ASXL2	Putative Po	153.82	1435	1.400411	0.48585	2	12.79057	12.30472
Q69YN4	VIRMA	Protein viril	202.025	1812	1.40005	0.485478	1	12.10545	11.61997
Q01105	SET	Protein SE	33.489	290	1.399869	0.485292	2	15.0642	14.5789
Q9H4L5	OSBPL3	Oxysterol-b	101.224	887	1.3995	0.484911	1	11.842	11.35709
Q7Z6I8	C5orf24	UPF0461 p	20.132	188	1.397871	0.483232	2	15.03716	14.55393
Q96SY0	INTS14	Integrator c	57.471	518	1.397786	0.483143	1	11.37199	10.88885
Q96AG4	LRRC59	Leucine-ric	34.93	307	1.396452	0.481766	2	16.2309	15.74914
P56524	HDAC4	Histone de	119.04	1084	1.396173	0.481477	1	10.59591	10.11443
Q9P0J7	KCMF1	E3 ubiquitin	41.945	381	1.395375	0.480653	1	11.06925	10.5886
P39023	RPL3	60S riboso	46.109	403	1.394538	0.479787	2	17.15086	16.67107
Q16643	DBN1	Drebrin OS	71.429	649	1.38966	0.474732	2	21.03792	20.56319
Q9HAV7	GRPEL1	GrpE prote	24.279	217	1.380766	0.465469	2	14.5073	14.04183
P30041	PRDX6	Peroxiredo	25.035	224	1.378908	0.463526	2	17.49217	17.02864
Q9Y697	NFS1	Cysteine de	50.196	457	1.377116	0.46165	1	12.83184	12.37019
P35232	PHB1	Prohibitin 1	29.804	272	1.376405	0.460905	2	16.10895	15.64805
Q9BUI4	POLR3C	DNA-direct	60.612	534	1.373792	0.458164	1	10.48563	10.02746
P52815	MRPL12	39S riboso	21.348	198	1.372847	0.457171	2	15.04793	14.59076
P50402	EMD	Emerin OS	28.994	254	1.371805	0.456076	2	18.31831	17.86224
Q96BQ5	CCDC127	Coiled-coil	30.834	260	1.368748	0.452857	2	13.0012	12.54834
Q96RQ3	MCCC1	Methylcroto	80.473	725	1.368208	0.452288	1	12.50797	12.05568
O43390	HNRNPR	Heterogene	70.943	633	1.36459	0.448467	2	18.01803	17.56956
Q13190	STX5	Syntaxin-5	39.673	355	1.364493	0.448365	1	12.02756	11.5792
P17535	JUND	Transcripti	35.174	347	1.364251	0.448109	1	11.5875	11.13939
P17081	RHOQ	Rho-relate	22.659	205	1.363072	0.446862	1	11.87244	11.42558

Q9NXH8	TOR4A	Torsin-4A (	46.914	423	1.362777	0.446549	1	11.33293	10.88638
Q15543	TAF13	Transcripti	14.287	124	1.361716	0.445426	1	12.29085	11.84543
P11142	HSPA8	Heat shock	70.898	646	1.360034	0.443643	2	20.61216	20.16852
Q86WR7	PROSER2	Proline anc	45.802	435	1.35605	0.439411	1	10.77025	10.33084
P14635	CCNB1	G2/mitotic-	48.337	433	1.355505	0.43883	1	11.63172	11.19289
P10515	DLAT	Dihydrolipo	68.997	647	1.352167	0.435273	2	19.35505	18.91978
Q9HDC5	JPH1	Junctophili	71.686	661	1.350642	0.433646	2	14.90068	14.46703
Q6NZY4	ZCCHC8	Zinc finger	78.577	707	1.349613	0.432546	1	12.62694	12.19439
Q9BSJ2	TUBGCP2	Gamma-tul	102.534	902	1.344949	0.427551	1	11.3936	10.96605
O43747	AP1G1	AP-1 comp	91.351	822	1.344948	0.42755	1	12.03115	11.6036
Q92560	BAP1	Ubiquitin ca	80.362	729	1.344404	0.426966	1	10.45615	10.02918
Q9P209	CEP72	Centrosom	71.718	647	1.343232	0.425709	1	10.75012	10.32441
Q14117	DPYS	Dihydropyri	56.63	519	1.343145	0.425615	2	21.96958	21.54397
Q04760	GLO1	Lactoylglut	20.778	184	1.342808	0.425254	2	14.26583	13.84058
O00483	NDUFA4	Cytochrom	9.37	81	1.34146	0.423804	2	15.83316	15.40936
Q9Y305	ACOT9	Acyl-coenz	49.902	439	1.339863	0.422085	1	11.64512	11.22303
P49821	NDUFV1	NADH deh	50.817	464	1.337955	0.42003	1	10.76553	10.3455
P61981	YWHAG	14-3-3 prot	28.303	247	1.337331	0.419357	2	17.11156	16.69221
Q96T60	PNKP	Bifunctiona	57.076	521	1.337062	0.419067	1	13.15801	12.73894
Q13636	RAB31	Ras-relatec	21.569	194	1.331633	0.413196	1	9.907477	9.49428
Q16204	CCDC6	Coiled-coil	53.291	474	1.331444	0.412992	1	12.36085	11.94785
O00461	GOLIM4	Golgi integ	81.88	696	1.330103	0.411538	1	11.55866	11.14712
Q9UPQ0	LIMCH1	LIM and ca	121.867	1083	1.329814	0.411225	2	18.85331	18.44208
Q14257	RCN2	Reticulocal	36.876	317	1.328545	0.409848	2	15.95756	15.54771
Q4G0J3	LARP7	La-related	66.899	582	1.327058	0.408231	1	10.92273	10.5145
Q8N0Z3	SPICE1	Spindle anc	96.264	855	1.311408	0.391117	1	11.14994	10.75882
P05187	ALPP	Alkaline ph	57.954	535	1.311276	0.390972	2	21.44733	21.05636
O75390	CS	Citrate synt	51.712	466	1.310394	0.39	2	13.31713	12.92713
P62424	RPL7A	60S riboso	29.996	266	1.308593	0.388017	2	17.75545	17.36743
Q08AD1	CAMSAP2	Calmodulin	168.089	1489	1.30619	0.385365	1	11.63635	11.25099
P26447	S100A4	Protein S1(	11.729	101	1.301751	0.380453	2	16.95015	16.56969
Q9H3K6	BOLA2	BolA-like p	10.117	86	1.299334	0.377773	2	16.53632	16.15855
P78317	RNF4	E3 ubiquitir	21.319	190	1.291925	0.369522	1	11.38003	11.01051
O14979	HNRNPDL	Heterogene	46.438	420	1.291714	0.369287	2	17.17163	16.80234
O43752	STX6	Syntaxin-6	29.176	255	1.289613	0.366938	1	11.48643	11.1195
P31947	SFN	14-3-3 prot	27.774	248	1.288983	0.366233	2	14.82451	14.45828
Q9BZC7	ABCA2	ATP-bindin	269.833	2435	1.288901	0.366142	2	14.76829	14.40215
Q9BUH6	PAXX	Protein PA	21.64	204	1.286702	0.363678	1	12.21556	11.85189
P62917	RPL8	60S riboso	28.025	257	1.283089	0.359622	2	15.98059	15.62096
P27824	CANX	Calnexin O	67.568	592	1.279303	0.355358	2	16.40293	16.04757
Q6IA86	ELP2	Elongator c	92.5	826	1.279184	0.355224	1	11.1814	10.82618
Q7Z5G4	GOLGA7	Golgin sub	15.824	137	1.27911	0.35514	2	15.59831	15.24317
Q9H3M7	TXNIP	Thioredoxi	43.661	391	1.278001	0.353889	1	10.40557	10.05168
Q9Y4B5	MTCL1	Microtubule	209.526	1905	1.276434	0.352119	2	21.92042	21.5683
P14866	HNRNPL	Heterogene	64.133	589	1.27572	0.351312	2	17.50207	17.15076
P51858	HDGF	Hepatoma-	26.788	240	1.274743	0.350207	2	12.7055	12.3553
P62140	PPP1CB	Serine/thre	37.187	327	1.272325	0.347467	2	18.34629	17.99882
P61978	HNRNPK	Heterogene	50.976	463	1.269597	0.344371	2	19.55354	19.20917
Q9Y4R8	TELO2	Telomere l	91.747	837	1.269051	0.34375	1	11.37183	11.02808
P16989	YBX3	Y-box-bind	40.09	372	1.26268	0.336489	2	19.2206	18.88411
Q9P0K7	RAI14	Ankycorbin	110.041	980	1.260381	0.33386	2	17.95299	17.61913
Q9GZL7	WDR12	Ribosome	47.708	423	1.258929	0.332197	1	11.37542	11.04322
Q92974	ARHGEF2	Rho guanir	111.543	986	1.258327	0.331507	2	15.18925	14.85774
Q58FF8	HSP90AB2	Putative he	44.349	381	1.257617	0.330693	2	15.66866	15.33797
P09234	SNRPC	U1 small nu	17.394	159	1.256114	0.328968	2	14.91135	14.58238
Q6Y7W6	GIGYF2	GRB10-inte	150.07	1299	1.254586	0.327212	1	11.93151	11.6043
Q16822	PCK2	Phosphoer	70.699	640	1.253331	0.325768	1	11.42007	11.0943
Q8WWM7	ATXN2L	Ataxin-2-lik	113.374	1075	1.252753	0.325101	2	16.87316	16.54805
P49773	HINT1	Adenosine	13.802	126	1.249555	0.321415	1	12.20689	11.88548

P27797	CALR	Calreticulin	48.142	417	1.247144	0.318628	2	15.22147	14.90285
P47929	LGALS7	Galectin-7	15.075	136	1.24398	0.314963	2	11.38024	11.06528
Q9P0W2	HMG20B	SWI/SNF-r	35.813	317	1.23707	0.306927	1	11.96773	11.6608
P35270	SPR	Sepiapterir	28.048	261	1.234297	0.30369	1	12.11097	11.80728
P24534	EEF1B2	Elongation	24.764	225	1.23114	0.299995	2	17.52787	17.22788
P55769	SNU13	NHP2-like	14.174	128	1.230399	0.299126	2	14.79223	14.4931
P09496	CLTA	Clathrin ligl	27.077	248	1.229359	0.297907	2	18.43131	18.1334
Q8WVJ2	NUDCD2	NudC dom	17.676	157	1.226595	0.294659	2	13.72014	13.42548
P04080	CSTB	Cystatin-B	11.14	98	1.226123	0.294104	2	16.03686	15.74276
O76094	SRP72	Signal recc	74.606	671	1.223847	0.291424	1	11.92035	11.62893
Q93096	PTP4A1	Protein tyrc	19.815	173	1.220887	0.28793	1	11.14638	10.85845
O00299	CLIC1	Chloride int	26.923	241	1.22088	0.287922	2	15.56314	15.27522
Q15365	PCBP1	Poly(rC)-bi	37.498	356	1.217657	0.284107	2	17.2473	16.96319
Q9NZ01	TECR	Very-long-c	36.034	308	1.215973	0.282111	2	15.0718	14.78969
Q99547	MPHOSPH	M-phase pl	19.024	160	1.215564	0.281625	1	11.70796	11.42634
P42771	CDKN2A	Cyclin-depe	16.533	156	1.207087	0.27153	2	15.32376	15.05223
Q9NQZ2	UTP3	Something	54.558	479	1.206981	0.271403	1	10.95855	10.68715
P20700	LMNB1	Lamin-B1 (	66.408	586	1.205788	0.269976	2	16.81541	16.54543
Q96ES7	SGF29	SAGA-asso	33.238	293	1.203222	0.266903	1	11.92166	11.65475
P08174	CD55	Compleme	41.4	381	1.203069	0.266719	2	21.71163	21.44491
Q9NYL9	TMOD3	Tropomodu	39.595	352	1.202165	0.265635	2	20.84843	20.58279
P27987	ITPKB	Inositol-tris	102.376	946	1.199297	0.262188	1	11.97696	11.71477
O75934	BCAS2	Pre-mRNA	26.131	225	1.198418	0.261131	2	15.02471	14.76358
P63244	RACK1	Receptor o	35.077	317	1.196919	0.259325	2	17.88232	17.623
Q8TED0	UTP15	U3 small nu	58.415	518	1.196063	0.258294	1	11.03232	10.77403
Q9P1Y5	CAMSAP3	Calmodulin	134.75	1249	1.193498	0.255197	1	11.76122	11.50602
Q92754	TFAP2C	Transcriptio	49.177	450	1.193081	0.254692	1	11.41606	11.16137
P21796	VDAC1	Voltage-de	30.773	283	1.188876	0.249599	2	16.28787	16.03828
Q92485	SMPDL3B	Acid sphing	50.814	455	1.187208	0.247572	2	15.48684	15.23926
P60866	RPS20	40S riboso	13.373	119	1.186098	0.246223	2	19.16379	18.91756
Q16526	CRY1	Cryptochro	66.395	586	1.181928	0.241143	1	9.702986	9.461844
O43324	EEF1E1	Eukaryotic	19.811	174	1.180854	0.23983	2	15.90431	15.66448
P67936	TPM4	Tropomyos	28.522	248	1.178229	0.23662	2	19.01487	18.77825
Q8IWZ8	SUGP1	SURP and	72.471	645	1.175191	0.232895	1	11.60904	11.37615
O95359	TACC2	Transformi	309.427	2948	1.17201	0.228985	1	12.09681	11.86783
P17676	CEBPB	CCAAT/en	36.106	345	1.167672	0.223635	2	15.92275	15.69911
Q9UKJ3	GPATCH8	G patch do	164.197	1502	1.166468	0.222147	1	11.87341	11.65126
O75610	LEFTY1	Left-right d	40.88	366	1.165207	0.220586	1	11.90805	11.68747
Q9Y3D3	MRPS16	28S riboso	15.345	137	1.163359	0.218296	2	14.38485	14.16656
P05783	KRT18	Keratin, typ	48.058	430	1.161431	0.215903	2	20.2573	20.0414
P26641	EEF1G	Elongation	50.119	437	1.158874	0.212724	2	16.97578	16.76306
P62318	SNRPD3	Small nucle	13.916	126	1.157571	0.2111	2	16.92091	16.70981
Q14738	PPP2R5D	Serine/thre	69.992	602	1.15723	0.210676	1	12.00257	11.79189
Q06830	PRDX1	Peroxiredo	22.11	199	1.157127	0.210548	2	20.29423	20.08369
Q14432	PDE3A	cGMP-inhil	124.979	1141	1.154109	0.206779	1	12.41276	12.20598
Q8IWR0	ZC3H7A	Zinc finger	110.538	971	1.151548	0.203575	1	11.59647	11.39289
P12277	CKB	Creatine ki	42.644	381	1.148881	0.20023	2	13.50258	13.30235
O75061	DNAJC6	Putative tyr	99.997	913	1.148615	0.199895	1	9.653938	9.454043
P21127	CDK11B	Cyclin-depe	92.62	795	1.148247	0.199433	2	14.03084	13.83141
O75030	MITF	Microphtha	58.795	526	1.146621	0.197389	2	15.45607	15.25868
Q9NY12	GAR1	H/ACA ribo	22.348	217	1.146165	0.196814	2	13.57199	13.37518
P43243	MATR3	Matrin-3 O	94.623	847	1.144278	0.194437	2	18.08135	17.88691
O15144	ARPC2	Actin-relate	34.333	300	1.141837	0.191357	2	15.92119	15.72983
Q9UKG1	APPL1	DCC-intera	79.663	709	1.14179	0.191297	1	10.86821	10.67691
Q15427	SF3B4	Splicing fac	44.386	424	1.141714	0.191201	2	15.6522	15.461
P82979	SARNP	SAP doma	23.671	210	1.141338	0.190726	1	12.97122	12.78049
Q8WXX5	DNAJC9	DnaJ homo	29.91	260	1.140625	0.189825	2	15.23695	15.04712
Q8NBJ5	COLGALT	Procollager	71.636	622	1.139165	0.187977	2	14.50631	14.31833
P24844	MYL9	Myosin reg	19.827	172	1.136092	0.184079	2	17.95606	17.77198

Q9H7L9	SUDS3	Sin3 histon	38.136	328	1.133775	0.181134		1	11.20555	11.02441
Q08J23	NSUN2	RNA cytosi	86.471	767	1.12708	0.17259		2	16.30201	16.12942
P56545	CTBP2	C-terminal-	48.945	445	1.125485	0.170547		1	10.95921	10.78866
P27635	RPL10	60S riboso	24.577	214	1.124935	0.169841		2	16.52787	16.35803
O95399	UTS2	Urotensin-2	14.296	124	1.122149	0.166264		2	18.61126	18.445
Q9UM54	MYO6	Unconventi	149.691	1294	1.122089	0.166186		2	17.28284	17.11665
Q9HB19	PLEKHA2	Pleckstrin h	47.255	425	1.12183	0.165854		1	10.73547	10.56962
O43795	MYO1B	Unconventi	131.985	1136	1.121403	0.165305		2	17.6035	17.43819
P12004	PCNA	Proliferating	28.769	261	1.120345	0.163943		2	16.47741	16.31347
O94992	HEXIM1	Protein HE	40.623	359	1.119981	0.163474		2	15.11362	14.95015
Q9H939	PSTPIP2	Proline-ser	38.858	334	1.118967	0.162168		1	11.42836	11.26619
P31943	HNRNPH1	Heterogene	49.229	449	1.117609	0.160416		2	19.85842	19.698
P49755	TMED10	Transmem	24.976	219	1.116674	0.159208		2	13.34027	13.18106
P09497	CLTB	Clathrin ligl	25.19	229	1.116519	0.159008		2	17.48919	17.33018
Q8N3C0	ASCC3	Activating s	251.46	2202	1.11647	0.158945		1	9.934915	9.775969
Q06203	PPAT	Amidophos	57.399	517	1.116464	0.158937		1	10.79612	10.63718
P36954	POLR2I	DNA-direct	14.523	125	1.115576	0.157789		1	11.48165	11.32386
Q07020	RPL18	60S riboso	21.634	188	1.113991	0.155737		2	18.04687	17.89113
Q15269	PWP2	Periodic try	102.452	919	1.113141	0.154636		1	9.695211	9.540575
P04818	TYMS	Thymidylat	35.716	313	1.112216	0.153437		1	10.64584	10.4924
Q9NT62	ATG3	Ubiquitin-lik	35.864	314	1.112008	0.153167		1	11.82536	11.67219
Q9Y3I0	RTCB	RNA-splicin	55.21	505	1.110058	0.150635		2	17.61115	17.46052
P23528	CFL1	Cofilin-1 OS	18.502	166	1.109629	0.150077		2	18.76361	18.61353
P26368	U2AF2	Splicing fac	53.501	475	1.108482	0.148586		2	16.78481	16.63623
Q0VGL1	LAMTOR4	Ragulator c	10.741	99	1.10789	0.147814		2	14.54134	14.39352
P23246	SFPQ	Splicing fac	76.149	707	1.106926	0.146559		2	17.27594	17.12938
Q00839	HNRNPU	Heterogene	90.584	825	1.105513	0.144716		2	19.77758	19.63286
Q15833	STXBP2	Syntaxin-bi	66.453	593	1.104796	0.143779		1	12.08208	11.9383
P49189	ALDH9A1	4-trimethyla	53.802	494	1.104212	0.143017		1	11.18748	11.04446
Q9NZI8	IGF2BP1	Insulin-like	63.481	577	1.103306	0.141833		2	18.05505	17.91322
P10599	TXN	Thioredoxin	11.738	105	1.103003	0.141437		2	18.31199	18.17055
Q7Z2E3	APTX	Aprataxin O	40.74	356	1.101691	0.13972		1	10.20665	10.06693
P49720	PSMB3	Proteasom	22.949	205	1.101623	0.13963		2	13.35287	13.21324
Q3MHD2	LSM12	Protein LSI	21.701	195	1.099253	0.136524		2	17.33132	17.1948
P59998	ARPC4	Actin-relate	19.667	168	1.099157	0.136397		2	16.63183	16.49543
Q9H974	QTRT2	Queuine tR	46.713	415	1.098609	0.135677		1	11.03184	10.89616
Q9NRM1	ENAM	Enamelin O	128.785	1142	1.097436	0.134137		2	16.94604	16.8119
Q9NPJ3	ACOT13	Acyl-coenz	14.96	140	1.096654	0.133108		1	11.00878	10.87567
P30050	RPL12	60S riboso	17.819	165	1.09551	0.131603		2	18.54909	18.41749
Q01081	U2AF1	Splicing fac	27.872	240	1.088101	0.121813		2	15.66027	15.53846
P25789	PSMA4	Proteasom	29.484	261	1.086128	0.119194		2	15.16188	15.04269
P61586	RHOA	Transformi	21.768	193	1.085468	0.118318		2	17.67521	17.5569
Q15025	TNIP1	TNFAIP3-in	71.864	636	1.08448	0.117003		1	10.94398	10.82698
P14649	MYL6B	Myosin ligh	22.764	208	1.081851	0.113502		2	17.93882	17.82532
Q8NE86	MCU	Calcium ur	39.867	351	1.079093	0.109819		1	11.01597	10.90615
P38606	ATP6V1A	V-type prot	68.304	617	1.077876	0.108191		2	14.36167	14.25348
O43865	AHCYL1	S-adenosyl	58.951	530	1.077166	0.10724		2	13.8746	13.76736
O00194	RAB27B	Ras-related	24.608	218	1.076927	0.106921		1	9.922094	9.815173
P52272	HNRNPM	Heterogene	77.516	730	1.075463	0.104958		2	17.65382	17.54887
Q7Z6Z7	HUWE1	E3 ubiquitin	481.891	4374	1.070922	0.098853		1	11.91719	11.81833
Q9BYN8	MRPS26	28S riboso	24.212	205	1.070184	0.09786		2	14.92704	14.82918
Q9NYR9	NKIRAS2	NF-kappa-I	21.508	191	1.070036	0.097659		1	11.06535	10.96769
Q96KP1	EXOC2	Exocyst co	104.066	924	1.069265	0.09662		1	10.79547	10.69885
Q9Y535	POLR3H	DNA-direct	22.918	204	1.0667	0.093154		1	12.08381	11.99066
P0DP25	CALM3	Calmodulin	16.838	149	1.066043	0.092265		2	21.97963	21.88736
P09493	TPM1	Tropomyos	32.709	284	1.064935	0.090765		2	17.08036	16.98959
P06753	TPM3	Tropomyos	32.95	285	1.062108	0.08693		2	20.19725	20.11032
Q5T9A4	ATAD3B	ATPase fan	72.573	648	1.061897	0.086644		2	16.10975	16.0231
P62306	SNRPF	Small nucle	9.725	86	1.060614	0.0849		2	15.24674	15.16184

Q9NP72	RAB18	Ras-relate	22.977	206	1.060162	0.084285	2	14.96583	14.88154
P07951	TPM2	Tropomyos	32.851	284	1.059929	0.083967	2	20.6118	20.52784
Q01658	DR1	Protein Dr1	19.444	176	1.059243	0.083033	2	16.46175	16.37872
Q5T0N5	FNBP1L	Formin-bin	70.065	605	1.058896	0.082561	1	11.44139	11.35883
P03928	MT-ATP8	ATP synth	7.992	68	1.056481	0.079267	2	15.16714	15.08788
P46781	RPS9	40S riboso	22.591	194	1.052707	0.074103	2	18.0202	17.9461
Q8NCA5	FAM98A	Protein FA	55.273	518	1.052245	0.073471	2	17.45161	17.37814
P47813	EIF1AX	Eukaryotic	16.46	144	1.051445	0.072373	2	14.25709	14.18472
Q9UK41	VPS28	Vacuolar p	25.425	221	1.050813	0.071506	1	11.30987	11.23837
O75340	PDCD6	Programm	21.868	191	1.050088	0.070511	1	10.85058	10.78007
Q15021	NCAPD2	Condensin	157.182	1401	1.045603	0.064336	2	12.21741	12.15308
P62304	SNRPE	Small nucl	10.804	92	1.044958	0.063445	2	15.98735	15.92391
Q6L8Q7	PDE12	2',5'-phosp	67.352	609	1.044107	0.062269	1	9.71134	9.649071
P40429	RPL13A	60S riboso	23.577	203	1.043767	0.0618	2	17.23088	17.16908
Q9Y2Q3	GSTK1	Glutathion	25.497	226	1.043339	0.061208	1	11.62981	11.5686
P37802	TAGLN2	Transgelin-	22.391	199	1.043026	0.060775	2	14.10722	14.04644
Q96AQ6	PBXIP1	Pre-B-cell l	80.643	731	1.042892	0.06059	1	11.41183	11.35124
P81877	SSBP2	Single-stra	37.828	361	1.038783	0.054894	2	15.30845	15.25355
P20340	RAB6A	Ras-relate	23.593	208	1.036894	0.052268	2	16.11823	16.06596
P09012	SNRPA	U1 small n	31.28	282	1.034721	0.049242	2	13.13043	13.08118
O43143	DHX15	ATP-deper	90.933	795	1.033976	0.048203	2	16.30692	16.25871
P62857	RPS28	40S riboso	7.841	69	1.033795	0.04795	2	18.28622	18.23827
P11182	DBT	Lipoamide	53.487	482	1.033591	0.047665	2	17.70002	17.65235
P22061	PCMT1	Protein-L-is	24.636	227	1.031744	0.045085	2	16.32625	16.28117
P17844	DDX5	Probable A	69.148	614	1.03158	0.044856	2	17.76946	17.72461
Q99798	ACO2	Aconitate h	85.425	780	1.03026	0.043009	1	9.687411	9.644402
Q9UPW6	SATB2	DNA-bindir	82.555	733	1.023931	0.034118	1	12.2035	12.16938
O75787	ATP6AP2	Renin rece	39.008	350	1.020621	0.029447	2	16.07008	16.04063
Q9BUT9	MCRIP2	MAPK regu	17.828	160	1.017524	0.025063	1	12.18797	12.16291
Q92499	DDX1	ATP-deper	82.432	740	1.015435	0.022098	2	17.29239	17.2703
Q7Z417	NUFIP2	Nuclear fra	76.121	695	1.014501	0.020771	2	17.04281	17.02204
Q6P2H3	CEP85	Centrosom	85.639	762	1.013401	0.019205	1	10.97993	10.96072
O00139	KIF2A	Kinesin-like	79.955	706	1.009812	0.014086	1	10.52209	10.508
Q9H3F6	KCTD10	BTB/POZ c	35.432	313	1.009645	0.013848	2	14.3537	14.33985
P00492	HPRT1	Hypoxanthi	24.579	218	1.009629	0.013826	2	13.44009	13.42626
P47985	UQCRFS1	Cytochrom	29.668	274	1.009489	0.013625	1	11.86299	11.84936
Q6P597	KLC3	Kinesin ligh	55.364	504	1.009446	0.013564	1	10.94464	10.93108
P51991	HNRNPA3	Heterogen	39.595	378	1.006178	0.008886	2	18.77272	18.76383
O15143	ARPC1B	Actin-relate	40.95	372	1.002889	0.004162	2	16.80574	16.80158
O76071	CIAO1	Probable c	37.84	339	0.999921	-0.00011	2	13.6339	13.63402
O00159	MYO1C	Unconventi	121.682	1063	0.999093	-0.00131	2	19.91838	19.91969
Q96EY4	TMA16	Translation	23.864	203	0.999038	-0.00139	2	15.26781	15.2692
P35580	MYH10	Myosin-10	228.999	1976	0.998737	-0.00182	2	20.07888	20.0807
P82909	MRPS36	Alpha-keto	11.466	103	0.996653	-0.00484	2	18.32127	18.3261
Q15084	PDIA6	Protein dis	48.121	440	0.994581	-0.00784	2	16.08207	16.08991
P17980	PSMC3	26S protea	49.204	439	0.993868	-0.00887	2	17.48274	17.49162
Q9HCU5	PREB	Prolactin re	45.468	417	0.992654	-0.01064	1	10.69653	10.70717
P08238	HSP90AB1	Heat shock	83.264	724	0.989263	-0.01557	2	17.90295	17.91853
P68363	TUBA1B	Tubulin alp	50.152	451	0.98924	-0.01561	2	20.06118	20.07679
P12236	SLC25A6	ADP/ATP t	32.866	298	0.987274	-0.01848	2	18.0914	18.10988
Q9H2J4	PDCL3	Phosducin-	27.614	239	0.981146	-0.02746	1	9.055282	9.082743
Q15233	NONO	Non-POU c	54.232	471	0.981054	-0.0276	2	16.84916	16.87676
Q9Y3E7	CHMP3	Charged m	25.073	222	0.977799	-0.03239	2	13.69925	13.73164
P06576	ATP5F1B	ATP synth	56.56	529	0.975676	-0.03553	2	16.72467	16.7602
Q9Y608	LRRFIP2	Leucine-ric	82.171	721	0.974323	-0.03753	2	19.70349	19.74102
Q12913	PTPRJ	Receptor-t	145.941	1337	0.970919	-0.04258	1	9.872475	9.915051
P68104	EEF1A1	Elongation	50.141	462	0.970298	-0.0435	2	19.6843	19.7278
P62491	RAB11A	Ras-relate	24.394	216	0.96981	-0.04423	2	14.12711	14.17133
Q9ULT8	HECTD1	E3 ubiquiti	289.368	2610	0.96933	-0.04494	1	12.11224	12.15718

Q9BRJ7	NUDT16L1	Tudor-inter	23.338	211	0.968745	-0.04581	2	14.61218	14.65799
Q3MIN7	RGL3	Ral guanin	78.079	710	0.967899	-0.04707	1	12.06127	12.10834
Q9UI30	TRMT112	Multifunctic	14.199	125	0.965128	-0.05121	2	16.30432	16.35552
Q04206	RELA	Transcripti	60.219	551	0.964922	-0.05152	1	10.66711	10.71863
Q96AC1	FERMT2	Fermitin fa	77.861	680	0.964434	-0.05225	1	9.56672	9.618965
Q16630	CPSF6	Cleavage a	59.21	551	0.963553	-0.05356	2	14.92049	14.97406
Q71UM5	RPS27L	40S ribosoi	9.477	84	0.962853	-0.05461	2	13.80191	13.85652
Q14919	DRAP1	Dr1-associ	22.35	205	0.962053	-0.05581	2	16.2916	16.34741
Q92526	CCT6B	T-complex	57.821	530	0.96108	-0.05727	2	14.89287	14.95015
P18124	RPL7	60S ribosoi	29.226	248	0.959092	-0.06026	2	17.51699	17.57725
Q14444	CAPRIN1	Caprin-1 O	78.366	709	0.95693	-0.06351	2	17.4103	17.47382
Q9Y679	AUP1	Lipid drople	45.787	410	0.956622	-0.06398	1	9.788604	9.852583
Q7L273	KCTD9	BTB/POZ d	42.567	389	0.953003	-0.06945	1	11.52826	11.59771
Q8WW01	TSEN15	tRNA-splici	18.641	171	0.952221	-0.07063	1	11.31781	11.38844
Q96TA1	NIBAN2	Protein Nib	84.138	746	0.951962	-0.07102	1	10.85907	10.93009
P63208	SKP1	S-phase ki	18.658	163	0.947069	-0.07846	2	16.82972	16.90818
O75494	SRSF10	Serine/argi	31.301	262	0.944885	-0.08179	2	15.06542	15.1472
Q8NDT2	RBM15B	Putative RI	97.205	890	0.944061	-0.08305	1	10.395	10.47805
P28288	ABCD3	ATP-bindin	75.476	659	0.942688	-0.08515	1	11.76885	11.85399
Q8NBU5	ATAD1	Outer mito	40.744	361	0.940828	-0.088	1	11.00899	11.09699
P10398	ARAF	Serine/thre	67.585	606	0.939863	-0.08948	1	11.56634	11.65582
Q9BQ75	CMSS1	Protein CM	31.884	279	0.933318	-0.09956	1	11.48018	11.57974
Q9Y4I1	MYO5A	Unconventi	215.405	1855	0.932622	-0.10064	2	15.56078	15.66142
P33316	DUT	Deoxyuridi	26.563	252	0.931384	-0.10255	2	14.75994	14.86249
O15232	MATN3	Matrilin-3 C	52.817	486	0.930566	-0.10382	2	16.56836	16.67218
O14618	CCS	Copper cha	29.041	274	0.928432	-0.10713	1	11.98606	12.09319
Q7L0Y3	TRMT10C	tRNA meth	47.347	403	0.927624	-0.10839	2	14.61586	14.72425
Q9Y291	MRPS33	28S ribosoi	12.629	106	0.92634	-0.11039	1	10.27391	10.3843
Q8WU76	SCFD2	Sec1 family	75.127	684	0.917602	-0.12406	1	10.51934	10.6434
Q9UDY4	DNAJB4	DnaJ homo	37.807	337	0.917536	-0.12416	2	10.48492	10.60909
Q9BZF1	OSBPL8	Oxysterol-b	101.196	889	0.916958	-0.12507	2	14.31904	14.44411
P21964	COMT	Catechol C	30.037	271	0.91271	-0.13177	1	11.76806	11.89983
P04439	HLA-A	HLA class	40.841	365	0.911666	-0.13342	2	15.94419	16.07761
Q93100	PHKB	Phosphoryl	124.884	1093	0.910449	-0.13535	2	17.38625	17.5216
Q14165	MLEC	Malectin O	32.234	292	0.909385	-0.13704	1	11.01304	11.15008
Q07021	C1QBP	Compleme	31.362	282	0.908297	-0.13876	2	17.81616	17.95492
P19484	TFEB	Transcripti	52.865	476	0.9082	-0.13892	2	15.58924	15.72816
Q9Y4L5	RNF115	E3 ubiquitir	33.703	304	0.90656	-0.14153	1	10.91722	11.05875
O60645	EXOC3	Exocyst co	85.567	745	0.90153	-0.14955	1	11.35706	11.50661
O95861	BPNT1	3'(2'),5'-bis	33.392	308	0.897607	-0.15584	1	10.42385	10.5797
O15479	MAGEB2	Melanoma-	35.277	319	0.897059	-0.15672	2	17.83603	17.99275
O60508	CDC40	Pre-mRNA	65.521	579	0.895753	-0.15883	1	10.82703	10.98585
P49458	SRP9	Signal reco	10.112	86	0.893052	-0.16318	2	16.98181	17.145
P28325	CST5	Cystatin-D	16.08	142	0.891412	-0.16584	2	17.86496	18.0308
Q9NRW7	VPS45	Vacuolar p	65.077	570	0.889586	-0.16879	1	11.29117	11.45997
Q96JA3	PLEKHA8	Pleckstrin I	58.261	519	0.889435	-0.16904	1	11.0337	11.20274
P08865	RPSA	40S ribosoi	32.854	295	0.886714	-0.17346	2	17.67521	17.84867
O15460	P4HA2	Prolyl 4-hy	60.902	535	0.879979	-0.18446	1	11.19297	11.37743
P25705	ATP5F1A	ATP synth	59.751	553	0.878129	-0.18749	2	17.23575	17.42325
P19623	SRM	Spermidine	33.825	302	0.876958	-0.18942	2	13.50755	13.69697
Q9Y224	RTRAF	RNA transc	28.068	244	0.872743	-0.19637	2	18.58255	18.77892
Q9Y5B9	SUPT16H	FACT com	119.914	1047	0.872311	-0.19709	2	12.72858	12.92567
Q13952	NFYC	Nuclear tra	50.302	458	0.871434	-0.19854	2	13.24116	13.4397
Q86WB0	ZC3HC1	Nuclear-int	55.262	502	0.870981	-0.19929	1	11.78815	11.98743
Q9NWU2	GID8	Glucose-in	26.749	228	0.864945	-0.20932	2	16.94203	17.15135
Q8IWS0	PHF6	PHD finger	41.29	365	0.863587	-0.21159	1	11.27514	11.48672
P19105	MYL12A	Myosin reg	19.794	171	0.86095	-0.216	2	22.94129	23.15729
P15531	NME1	Nucleoside	17.149	152	0.85822	-0.22058	2	15.40787	15.62845
Q9H1I8	ASCC2	Activating s	86.36	757	0.852284	-0.23059	1	11.35028	11.58087

Q969G3	SMARCE1	SWI/SNF-r	46.649	411	0.851578	-0.23179	2	13.64002	13.87181
Q9HD42	CHMP1A	Charged m	21.703	196	0.851486	-0.23194	2	14.34395	14.57589
O76081	RGS20	Regulator c	43.692	388	0.847996	-0.23787	1	10.76296	11.00083
P62258	YWHAE	14-3-3 prot	29.174	255	0.847343	-0.23898	2	18.01465	18.25364
Q9H444	CHMP4B	Charged m	24.95	224	0.843093	-0.24624	2	17.24656	17.49279
Q5JTC6	AMER1	APC memt	124.029	1135	0.839495	-0.25241	1	9.360518	9.612925
P31942	HNRNPH3	Heterogene	36.926	346	0.838309	-0.25445	2	17.47548	17.72993
P31431	SDC4	Syndecan-4	21.642	198	0.838214	-0.25461	2	16.09369	16.3483
Q5JVF3	PCID2	PCI domain	46.03	399	0.83321	-0.26325	1	10.78382	11.04706
Q86WR0	CCDC25	Coiled-coil	24.479	208	0.832426	-0.26461	1	10.57913	10.84373
Q6GMV3	PTRHD1	Putative pe	15.805	140	0.831269	-0.26661	1	10.81634	11.08296
P48735	IDH2	Isocitrate d	50.909	452	0.831237	-0.26667	1	11.14644	11.41311
A1L0T0	ILVBL	2-hydroxya	67.868	632	0.830045	-0.26874	1	12.20405	12.47279
P12036	NEFH	Neurofilam	112.479	1026	0.827513	-0.27315	2	17.51237	17.78552
Q02241	KIF23	Kinesin-like	110.059	960	0.826314	-0.27524	1	11.38046	11.6557
P49841	GSK3B	Glycogen s	46.744	420	0.825513	-0.27664	1	10.91132	11.18795
Q96A35	MRPL24	39S riboso	24.915	216	0.82541	-0.27682	1	11.08008	11.3569
Q9UDT6	CLIP2	CAP-Gly do	115.837	1046	0.822895	-0.28122	1	10.01207	10.29329
O95208	EPN2	Epsin-2 OS	68.482	641	0.822216	-0.28241	2	12.71669	12.9991
P46734	MAP2K3	Dual specif	39.318	347	0.821064	-0.28443	2	17.33856	17.623
P50914	RPL14	60S riboso	23.432	215	0.821064	-0.28443	2	17.67859	17.96302
Q9Y6Y8	SEC23IP	SEC23-inte	111.076	1000	0.820209	-0.28594	1	10.95529	11.24122
P63261	ACTG1	Actin, cytop	41.793	375	0.819057	-0.28796	2	23.96878	24.25674
P62241	RPS8	40S riboso	24.205	208	0.8171	-0.29141	2	18.10001	18.39142
Q15554	TERF2	Telomeric r	59.594	542	0.813424	-0.29792	1	11.39548	11.6934
P22626	HNRNPA2B	Heterogene	37.43	353	0.810593	-0.30295	2	19.7154	20.01835
O75746	SLC25A12	Calcium-bin	74.762	678	0.809457	-0.30497	1	10.39489	10.69987
P35637	FUS	RNA-bindin	53.426	526	0.808429	-0.30681	2	17.26142	17.56822
P13010	XRCC5	X-ray repai	82.705	732	0.807843	-0.30785	2	19.66131	19.96916
P18827	SDC1	Syndecan-	32.462	310	0.807776	-0.30797	2	17.24563	17.5536
Q04759	PRKCQ	Protein kin	81.865	706	0.807184	-0.30903	1	10.07775	10.38678
P36957	DLST	Dihydrolipo	48.755	453	0.806593	-0.31009	2	20.49277	20.80286
Q13595	TRA2A	Transforme	32.689	282	0.805661	-0.31175	2	15.44204	15.75379
Q9P107	GMIP	GEM-intera	106.683	970	0.805209	-0.31256	2	16.66456	16.97712
O95825	CRYZL1	Quinone ox	38.697	349	0.805128	-0.31271	1	11.16434	11.47705
Q7RTP6	MICAL3	[F-actin]-m	224.295	2002	0.802221	-0.31793	2	16.05301	16.37094
Q14573	ITPR3	Inositol 1,4	304.106	2671	0.801828	-0.31864	2	13.00976	13.32839
Q5JRA6	MIA3	Transport a	213.702	1907	0.801645	-0.31897	1	10.38651	10.70547
P19447	ERCC3	General tra	89.278	782	0.799292	-0.32321	1	12.16914	12.49235
P62249	RPS16	40S riboso	16.445	146	0.797405	-0.32662	2	18.50428	18.8309
Q9BTV4	TMEM43	Transmem	44.876	400	0.796439	-0.32836	1	12.00454	12.3329
Q32P28	P3H1	Prolyl 3-hyd	83.394	736	0.796305	-0.32861	1	9.666721	9.995327
Q10713	PMPCA	Mitochondr	58.253	525	0.795539	-0.33	1	10.01025	10.34024
Q9UJS0	SLC25A13	Calcium-bin	74.176	675	0.795067	-0.33085	2	15.65072	15.98157
O00499	BIN1	Myc box-de	64.699	593	0.794873	-0.3312	1	10.94603	11.27723
P38159	RBMX	RNA-bindin	42.332	391	0.793637	-0.33345	2	18.356	18.68945
Q9ULE4	FAM184B	Protein FAI	121.044	1060	0.793446	-0.3338	2	15.97374	16.30754
Q15363	TMED2	Transmem	22.761	201	0.792314	-0.33586	1	9.491452	9.827308
Q9NVH1	DNAJC11	DnaJ homo	63.278	559	0.790005	-0.34007	1	10.43139	10.77146
Q9H0U4	RAB1B	Ras-related	22.171	201	0.788512	-0.3428	2	16.09077	16.43357
Q15149	PLEC	Plectin OS	531.791	4684	0.785953	-0.34748	2	16.1803	16.52778
P62308	SNRPG	Small nucle	8.496	76	0.783722	-0.35159	2	15.80024	16.15183
P17066	HSPA6	Heat shock	71.028	643	0.782664	-0.35354	2	19.65213	20.00566
O14936	CASK	Peripheral	105.123	926	0.782004	-0.35475	1	10.60261	10.95736
Q2NL82	TSR1	Pre-rRNA-p	91.81	804	0.781537	-0.35561	2	16.09063	16.44624
O75970	MPDZ	Multiple PD	221.618	2070	0.780714	-0.35713	1	11.36288	11.72001
Q9HAF1	MEAF6	Chromatin	21.635	191	0.780263	-0.35797	1	11.60377	11.96173
P39656	DDOST	Dolichyl-dip	50.801	456	0.780253	-0.35799	2	13.97244	14.33043
P52701	MSH6	DNA mism	152.786	1360	0.779573	-0.35924	2	15.58798	15.94723

Q8IXM3	MRPL41	39S riboso	15.383	137	0.779477	-0.35942		2	13.78013	14.13955
P19532	TFE3	Transcripti	61.521	575	0.779147	-0.36003		2	15.2537	15.61373
Q13243	SRSF5	Serine/argi	31.264	272	0.776933	-0.36414		2	16.21487	16.57901
P09038	FGF2	Fibroblast g	30.77	288	0.775619	-0.36658		2	14.31345	14.68003
P12694	BCKDHA	2-oxoisova	50.471	445	0.774416	-0.36882		1	10.8181	11.18692
Q9BYV8	CEP41	Centrosom	41.368	373	0.773495	-0.37054		1	9.723507	10.09404
P56134	ATP5MF	ATP syntha	10.918	94	0.772455	-0.37248		2	13.56784	13.94031
Q13823	GNL2	Nucleolar G	83.655	731	0.771137	-0.37494		1	10.85689	11.23183
Q6P2E9	EDC4	Enhancer o	151.661	1401	0.77099	-0.37522		2	18.82582	19.20103
O75531	BANF1	Barrier-to-a	10.059	89	0.770989	-0.37522		2	20.79416	21.16938
P35579	MYH9	Myosin-9 O	226.532	1960	0.769206	-0.37856		2	20.87147	21.25003
P06703	S100A6	Protein S10	10.18	90	0.768691	-0.37952		2	14.09754	14.47706
Q9NQX4	MYO5C	Unconventi	202.81	1742	0.766903	-0.38288		1	9.781393	10.16428
O95347	SMC2	Structural r	135.656	1197	0.764929	-0.3866		1	11.60205	11.98865
Q9Y2R4	DDX52	Probable A	67.466	599	0.762929	-0.39038		1	11.35805	11.74843
O60292	SIPA1L3	Signal-indu	194.61	1781	0.761972	-0.39219		2	17.19499	17.58718
P22612	PRKACG	cAMP-depe	40.434	351	0.761624	-0.39285		2	20.46404	20.85688
Q9BYI3	FAM126A	Hyccin OS=	57.625	521	0.760586	-0.39482		2	10.90433	11.29915
P09651	HNRNPA1	Heterogene	38.747	372	0.75801	-0.39971		2	20.16226	20.56197
Q9UHV9	PFDN2	Prefoldin su	16.648	154	0.757578	-0.40053		2	15.02717	15.42771
Q9UHD9	UBQLN2	Ubiquilin-2	65.696	624	0.756815	-0.40199		1	10.48442	10.88641
P27694	RPA1	Replication	68.138	616	0.755376	-0.40473		2	17.39615	17.80089
Q02543	RPL18A	60S riboso	20.762	176	0.755227	-0.40502		2	16.57233	16.97735
Q15717	ELAVL1	ELAV-like p	36.092	326	0.753305	-0.40869		2	17.26279	17.67149
O60271	SPAG9	C-Jun-amin	146.205	1321	0.752244	-0.41073		1	10.56205	10.97278
P23396	RPS3	40S riboso	26.688	243	0.751842	-0.4115		2	18.02832	18.43981
P51812	RPS6KA3	Ribosomal	83.736	740	0.750499	-0.41408		1	10.81162	11.22569
P09661	SNRPA1	U2 small nu	28.416	255	0.747838	-0.4192		2	15.34696	15.76617
Q96P11	NSUN5	28S rRNA (	46.692	429	0.743585	-0.42743		2	13.42285	13.85028
O15085	ARHGEF1	Rho guanin	167.704	1522	0.742913	-0.42873		2	15.00655	15.43528
P11940	PABPC1	Polyadenyl	70.671	636	0.742698	-0.42915		2	20.586	21.01516
Q9BUP3	HTATIP2	Oxidoreduc	27.049	242	0.741779	-0.43094		1	10.92874	11.35968
P17096	HMGA1	High mobili	11.676	107	0.734541	-0.44509		2	20.24342	20.68851
Q8N3E9	PLCD3	1-phosphat	89.258	789	0.733894	-0.44636		2	16.6647	17.11105
Q13310	PABPC4	Polyadenyl	70.783	644	0.728722	-0.45656		2	18.6648	19.12136
P42765	ACAA2	3-ketoacyl-	41.924	397	0.72707	-0.45983		1	10.25668	10.71651
P23381	WARS1	Tryptophan	53.165	471	0.7256	-0.46275		2	14.90722	15.36997
Q8NBK3	SUMF1	Formylglyc	40.556	374	0.724303	-0.46533		1	10.41975	10.88508
P46779	RPL28	60S riboso	15.748	137	0.724152	-0.46564		2	16.70658	17.17221
P36873	PPP1CC	Serine/thre	36.984	323	0.721784	-0.47036		2	15.62973	16.10009
Q13425	SNTB2	Beta-2-syn	57.95	540	0.705403	-0.50348		1	10.04398	10.54746
Q9NV56	MRGBP	MRG/MOR	22.417	204	0.700581	-0.51338		2	14.37327	14.88665
O43684	BUB3	Mitotic che	37.155	328	0.698722	-0.51721		2	16.4073	16.92451
P27105	STOM	Stomatin O	31.731	288	0.697339	-0.52007		2	16.28164	16.80171
Q9NVI7	ATAD3A	ATPase fa	71.369	634	0.693918	-0.52716		2	16.24765	16.77481
Q9H8G2	CAAP1	Caspase a	38.368	361	0.690877	-0.5335		1	11.44476	11.97826
Q6P587	FAHD1	Acylpyruva	24.843	224	0.689524	-0.53633		1	12.04145	12.57778
P62753	RPS6	40S riboso	28.681	249	0.688549	-0.53837		2	18.10503	18.6434
P16435	POR	NADPH--cy	76.69	677	0.68669	-0.54227		1	10.94983	11.49209
Q9UBU9	NXF1	Nuclear RN	70.182	619	0.685576	-0.54461		2	14.28951	14.83413
P56192	MARS1	Methionine	101.116	900	0.679083	-0.55834		2	16.65746	17.2158
P30101	PDIA3	Protein disu	56.782	505	0.677871	-0.56092		2	14.16844	14.72936
O75400	PRPF40A	Pre-mRNA	108.805	957	0.67637	-0.56412		2	15.20892	15.77304
Q8WYQ5	DGCR8	Microproce	86.045	773	0.674284	-0.56857		1	10.17005	10.73862
P46782	RPS5	40S riboso	22.876	204	0.67353	-0.57019		2	17.44387	18.01405
P30048	PRDX3	Thioredoxin	27.693	256	0.673426	-0.57041		2	14.36646	14.93687
Q96IX5	ATP5MK	ATP syntha	6.458	58	0.671659	-0.5742		2	14.88574	15.45994
Q14676	MDC1	Mediator of	226.666	2089	0.66868	-0.58061		1	9.19101	9.771623
Q92917	GPKOW	G-patch do	52.229	476	0.664705	-0.58921	-	2	16.98671	17.57592

P62888	RPL30	60S riboso	12.784	115	0.664632	-0.58937	-	2	16.74649	17.33586
P11441	UBL4A	Ubiquitin-lik	17.777	157	0.660187	-0.59905	-	2	13.84402	14.44307
Q16698	DECR1	2,4-dienoyl	36.068	335	0.659158	-0.6013	-	1	10.31152	10.91283
Q8N3D4	EHBP1L1	EH domain	161.854	1523	0.659107	-0.60142	-	1	11.17941	11.78083
Q16654	PDK4	[Pyruvate c	46.469	411	0.658685	-0.60234	-	1	10.82233	11.42467
P07195	LDHB	L-lactate de	36.638	334	0.658299	-0.60319	-	2	14.47763	15.08082
P22392	NME2	Nucleoside	17.298	152	0.654311	-0.61195	-	2	13.20113	13.81308
P60660	MYL6	Myosin ligh	16.93	151	0.650249	-0.62094	-	2	22.85264	23.47358
Q6UB35	MTHFD1L	Monofuncti	105.79	978	0.647164	-0.6278	-	2	12.41812	13.04591
Q8NI35	PATJ	InaD-like p	196.368	1801	0.643961	-0.63496	-	2	15.88698	16.52194
Q7LBC6	KDM3B	Lysine-spe	191.581	1761	0.641595	-0.64026	-	1	11.51978	12.16005
Q99081	TCF12	Transcriptio	72.965	682	0.640281	-0.64322	-	1	10.49625	11.13948
Q6PD62	CTR9	RNA polym	133.502	1173	0.637589	-0.6493	-	1	9.2174	9.866702
Q71UI9	H2AZ2	Histone H2	13.509	128	0.63743	-0.64966	-	2	15.54798	16.19764
P04406	GAPDH	Glyceralde	36.053	335	0.635138	-0.65486	-	2	17.31487	17.96972
P35080	PFN2	Profilin-2 C	15.046	140	0.630836	-0.66466	-	2	16.2816	16.94627
Q9NY93	DDX56	Probable A	61.59	547	0.63075	-0.66486	-	2	13.65072	14.31557
O43491	EPB41L2	Band 4.1-lil	112.588	1005	0.627758	-0.67172	-	1	10.12218	10.7939
Q8N9T8	KRI1	Protein KR	82.598	703	0.627414	-0.67251	-	2	13.75343	14.42594
Q9UJX4	ANAPC5	Anaphase-	85.077	755	0.627355	-0.67265	-	1	11.20457	11.87722
O14879	IFIT3	Interferon-i	55.985	490	0.625235	-0.67753	-	2	15.1476	15.82513
Q9H1A4	ANAPC1	Anaphase-	216.5	1944	0.617929	-0.69449	-	1	10.54429	11.23878
P12956	XRCC6	X-ray repai	69.843	609	0.617544	-0.69539	-	2	20.30017	20.99555
Q9H9A6	LRRC40	Leucine-ric	68.25	602	0.616188	-0.69856	-	1	11.38726	12.08582
Q92522	H1-10	Histone H1	22.487	213	0.614977	-0.7014	-	2	15.77061	16.47201
Q5TEC6	H3-7	Histone H3	15.43	136	0.611883	-0.70867	-	2	15.67496	16.38364
P35658	NUP214	Nuclear po	213.62	2090	0.611091	-0.71054	-	2	14.15553	14.86607
P0C7P3	SLFN14	Protein SLI	103.907	912	0.610229	-0.71258	-	2	15.23672	15.9493
P84090	ERH	Enhancer o	12.259	104	0.609893	-0.71337	-	2	18.61177	19.32514
O00468	AGRN	Agrin OS=H	217.32	2068	0.609808	-0.71357	-	1	11.91498	12.62855
Q93077	H2AC6	Histone H2	14.105	130	0.607865	-0.71818	-	2	20.06171	20.77989
P39019	RPS19	40S riboso	16.061	145	0.604552	-0.72606	-	2	17.79627	18.52233
P62244	RPS15A	40S riboso	14.84	130	0.603367	-0.72889	-	2	18.60317	19.33206
P37108	SRP14	Signal reco	14.57	136	0.602922	-0.72996	-	2	18.20271	18.93266
Q9H4A4	RNPEP	Aminopepti	72.596	650	0.602175	-0.73175	-	1	9.978996	10.71074
P36542	ATP5F1C	ATP syntha	32.996	298	0.601469	-0.73344	-	2	15.84909	16.58252
Q9UJ83	HACL1	2-hydroxya	63.729	578	0.601116	-0.73428	-	1	10.42626	11.16055
Q8TCT9	HM13	Minor histo	41.488	377	0.598108	-0.74152	-	1	11.04651	11.78803
Q9UJV9	DDX41	Probable A	69.838	622	0.596929	-0.74437	-	2	14.28345	15.02782
P14618	PKM	Pyruvate ki	57.937	531	0.595338	-0.74822	-	2	17.4165	18.16472
P46778	RPL21	60S riboso	18.565	160	0.593989	-0.75149	-	2	16.89581	17.6473
Q15477	SKIV2L	Helicase S	137.755	1246	0.593623	-0.75238	-	1	9.752247	10.50463
Q8N257	H2BU1	Histone H2	13.908	126	0.592084	-0.75613	-	2	17.78622	18.54234
Q9NX63	CHCHD3	MICOS co	26.152	227	0.590941	-0.75891	-	2	17.20619	17.96511
P15927	RPA2	Replication	29.247	270	0.590195	-0.76074	-	2	17.18302	17.94375
O94888	UBXN7	UBX doma	54.862	489	0.589841	-0.7616	-	1	10.89618	11.65778
Q9UKM9	RALY	RNA-bindir	32.463	306	0.58818	-0.76567	-	2	15.28584	16.05151
Q9Y3B4	SF3B6	Splicing fac	14.585	125	0.580762	-0.78398	-	2	15.54306	16.32704
Q13769	THOC5	THO comp	78.508	683	0.58023	-0.7853	-	1	10.19722	10.98252
P29401	TKT	Transketola	67.878	623	0.578271	-0.79018	-	2	13.46263	14.25281
P36402	TCF7	Transcriptio	41.642	384	0.576999	-0.79336	-	2	13.20567	13.99903
P06733	ENO1	Alpha-enola	47.169	434	0.576391	-0.79488	-	2	18.13004	18.92492
Q12774	ARHGEF5	Rho guanir	176.799	1597	0.57531	-0.79759	-	1	11.15678	11.95437
Q6P1L8	MRPL14	39S riboso	15.948	145	0.572492	-0.80467	-	2	14.27569	15.08036
P18077	RPL35A	60S riboso	12.538	110	0.569357	-0.8126	-	2	15.68604	16.49863
O60313	OPA1	Dynamin-lil	111.631	960	0.567668	-0.81688	-	1	11.29198	12.10886
P62995	TRA2B	Transforme	33.666	288	0.566154	-0.82073	-	2	16.50629	17.32703
Q16891	IMMT	MICOS co	83.678	758	0.563064	-0.82863	-	2	17.36145	18.19008
P13674	P4HA1	Prolyl 4-hyd	61.049	534	0.562664	-0.82966	-	1	9.966217	10.79587

E9PAV3	NACA	Nascent pc	205.422	2078	0.56201	-0.83133	-	2	15.62025	16.45158
P33993	MCM7	DNA replic	81.308	719	0.561349	-0.83303	-	2	19.0789	19.91193
P35244	RPA3	Replication	13.569	121	0.558881	-0.83939	-	2	16.88489	17.72427
Q9BW91	NUDT9	ADP-ribose	39.125	350	0.557523	-0.8429	-	1	10.20225	11.04514
P61353	RPL27	60S riboso	15.798	136	0.555888	-0.84713	-	2	17.28446	18.1316
Q9BWC9	CCDC106	Coiled-coil	32.032	280	0.553511	-0.85332	-	1	9.845192	10.69851
Q9NR50	EIF2B3	Translation	50.24	452	0.552521	-0.8559	-	1	9.989678	10.84558
Q6BDS2	UHRF1BP1	UHRF1-bir	159.485	1440	0.546477	-0.87177	-	2	17.67231	18.54408
P38935	IGHMBP2	DNA-bindir	109.149	993	0.544711	-0.87644	-	1	10.86132	11.73776
P20339	RAB5A	Ras-relatec	23.659	215	0.543291	-0.8802	-	2	14.77695	15.65715
Q13043	STK4	Serine/thre	55.63	487	0.542769	-0.88159	-	1	9.355725	10.23732
Q96I25	RBM17	Splicing fac	44.962	401	0.540494	-0.88765	-	2	14.82595	15.7136
P49756	RBM25	RNA-bindir	100.186	843	0.540181	-0.88849	-	2	14.76254	15.65102
Q6YN16	HSDL2	Hydroxyste	45.395	418	0.53952	-0.89025	-	1	12.28207	13.17233
O00116	AGPS	Alkyldihydr	72.912	658	0.538343	-0.8934	-	1	10.77964	11.67304
Q9H173	SIL1	Nucleotide	52.085	461	0.536977	-0.89707	-	1	11.45234	12.34941
P69849	NOMO3	Nodal mod	134.134	1222	0.533478	-0.9065	-	2	16.43084	17.33734
Q03169	TNFAIP2	Tumor nec	72.661	654	0.528903	-0.91893	-	2	13.56332	14.48224
Q9UHW5	GPN3	GPN-loop (	32.761	284	0.528844	-0.91909	-	1	9.401648	10.32073
O00339	MATN2	Matrilin-2 C	106.837	956	0.52823	-0.92076	-	2	15.9102	16.83096
Q99623	PHB2	Prohibitin-2	33.296	299	0.527458	-0.92287	-	2	15.1014	16.02427
Q9Y3C7	MED31	Mediator of	15.805	131	0.524803	-0.93015	-	1	11.78957	12.71973
Q96A65	EXOC4	Exocyst co	110.498	974	0.522705	-0.93593	-	1	10.03025	10.96618
Q96EN8	MOCOS	Molybdenu	98.12	888	0.520138	-0.94303	-	1	9.11017	10.0532
Q15056	EIF4H	Eukaryotic	27.385	248	0.519824	-0.9439	-	2	13.40966	14.35356
P36639	NUDT1	Oxidized pu	17.952	156	0.51901	-0.94616	-	2	13.1442	14.09036
P84103	SRSF3	Serine/argi	19.33	164	0.518817	-0.9467	-	2	16.98815	17.93485
Q5T1V6	DDX59	Probable A	68.81	619	0.517629	-0.95001	-	1	10.37395	11.32396
Q8IUD2	ERC1	ELKS/Rab6	128.086	1116	0.516858	-0.95216	-	2	14.14163	15.09379
O75600	GCAT	2-amino-3-	45.285	419	0.512797	-0.96354	-	1	10.77017	11.73371
P43490	NAMPT	Nicotinamid	55.521	491	0.509771	-0.97208	-	1	9.788457	10.76053
P53992	SEC24C	Protein tran	118.325	1094	0.50928	-0.97347	-	2	14.97042	15.94389
P26373	RPL13	60S riboso	24.261	211	0.502461	-0.99292	-	2	17.57274	18.56566
Q9UHR5	SAP30BP	SAP30-bin	33.87	308	0.500097	-0.99972	-	2	14.49467	15.49439
Q9UGP4	LIMD1	LIM domain	72.19	676	0.499622	-1.00109	-	1	11.70684	12.70793
Q5VYK3	ECPAS	Proteasom	204.291	1845	0.496553	-1.00998	-	1	10.65714	11.66712
P02792	FTL	Ferritin ligh	20.02	175	0.494776	-1.01515	-	2	13.51385	14.529
P17655	CAPN2	Calpain-2 c	79.995	700	0.494251	-1.01668	-	1	11.35893	12.37561
Q8N1G4	LRRC47	Leucine-ric	63.473	583	0.489023	-1.03203	-	2	15.69411	16.72614
P83111	LACTB	Serine beta	60.694	547	0.488001	-1.03505	-	2	14.30585	15.34089
O43805	SSNA1	Sjoegren s	13.596	119	0.486993	-1.03803	-	1	8.618275	9.656302
Q9BV73	CEP250	Centrosom	281.137	2442	0.481106	-1.05557	-	2	15.33127	16.38684
P84098	RPL19	60S riboso	23.466	196	0.480166	-1.05839	-	2	18.22091	19.2793
P07910	HNRNPC	Heterogene	33.67	306	0.479861	-1.05931	-	2	18.97994	20.03926
Q92979	EMG1	Ribosomal	26.72	244	0.479823	-1.05943	-	1	10.41532	11.47475
Q9NVM9	INTS13	Integrator c	80.225	706	0.478805	-1.06249	-	1	10.79888	11.86137
O43252	PAPSS1	Bifunctiona	70.833	624	0.477872	-1.06531	-	1	11.28575	12.35105
P48047	ATP5PO	ATP syntha	23.277	213	0.477608	-1.0661	-	2	16.83997	17.90607
Q14789	GOLGB1	Golgin sub	376.019	3259	0.471384	-1.08502	-	2	16.65396	17.73899
Q15054	POLD3	DNA polym	51.4	466	0.467799	-1.09604	-	1	9.689876	10.78591
Q86VS8	HOOK3	Protein Ho	83.126	718	0.467762	-1.09615	-	1	10.46852	11.56468
P56179	DLX6	Homeobox	19.708	175	0.465129	-1.1043	-	2	14.06508	15.16938
O43426	SYNJ1	Synaptojan	173.103	1573	0.463279	-1.11005	-	2	13.5753	14.68535
P27695	APEX1	DNA-(apur	35.555	318	0.459897	-1.12062	-	1	9.48774	10.60836
Q9HCD6	TANC2	Protein TAI	219.65	1990	0.457364	-1.12859	-	1	10.90456	12.03314
P17612	PRKACA	cAMP-depe	40.59	351	0.457321	-1.12872	-	2	13.51767	14.64639
Q9Y421	FAM32A	Protein FAI	13.178	112	0.453878	-1.13962	-	1	11.40482	12.54445
P00338	LDHA	L-lactate de	36.689	332	0.453119	-1.14204	-	2	14.97065	16.11268
P29353	SHC1	SHC-transf	62.822	583	0.451535	-1.14709	-	2	13.218	14.36509

P49207	RPL34	60S riboso	13.293	117	0.447851	-1.15891	-	2	15.87791	17.03682
Q9Y3C1	NOP16	Nucleolar p	21.188	178	0.446576	-1.16302	-	2	15.6025	16.76552
P62854	RPS26	40S riboso	13.015	115	0.445974	-1.16497	-	2	17.53488	18.69985
Q5JR59	MTUS2	Microtubule	150.195	1369	0.445083	-1.16785	-	1	10.57696	11.74481
O75717	WDHD1	WD repeat	125.967	1129	0.442517	-1.17619	-	2	11.02001	12.1962
O94905	ERLIN2	Erlin-2 OS=	37.84	339	0.440221	-1.1837	-	2	13.47763	14.66133
P50897	PPT1	Palmitoyl-p	34.194	306	0.436502	-1.19594	-	1	9.166339	10.36228
Q9BRS2	RIOK1	Serine/thre	65.583	568	0.436035	-1.19748	-	2	11.02908	12.22656
O43809	NUDT21	Cleavage a	26.227	227	0.434481	-1.20263	-	2	15.78307	16.98571
Q6ZRP7	QSOX2	Sulfhydryl o	77.529	698	0.43447	-1.20267	-	1	9.897165	11.09983
Q7Z7K6	CENPV	Centromere	29.946	275	0.431778	-1.21164	-	1	11.08687	12.2985
Q13797	ITGA9	Integrin alp	114.489	1035	0.428953	-1.22111	-	2	17.67079	18.8919
Q96S19	METTL26	Methyltrans	22.578	204	0.428542	-1.22249	-	1	9.85292	11.07541
Q9NPE3	NOP10	H/ACA ribo	7.706	64	0.427828	-1.2249	-	2	12.858	14.0829
P23284	PPIB	Peptidyl-pro	23.743	216	0.426038	-1.23094	-	2	13.81067	15.04162
P51648	ALDH3A2	Aldehyde d	54.848	485	0.424298	-1.23685	-	1	10.17904	11.41589
Q9Y2W2	WBP11	WW doma	69.998	641	0.423897	-1.23821	-	2	13.33022	14.56843
Q92530	PSMF1	Proteasom	29.817	271	0.423533	-1.23945	-	1	9.303963	10.54342
Q96TC7	RMDN3	Regulator o	52.118	470	0.422688	-1.24233	-	2	13.85038	15.09272
P26358	DNMT1	DNA (cytos	183.165	1616	0.419845	-1.25207	-	2	16.67852	17.93059
O60783	MRPS14	28S riboso	15.139	128	0.41906	-1.25477	-	2	14.40341	15.65818
P08758	ANXA5	Annexin A5	35.937	320	0.41899	-1.25501	-	1	10.03094	11.28596
P13861	PRKAR2A	cAMP-depe	45.518	404	0.418236	-1.25761	-	2	13.87565	15.13326
P62851	RPS25	40S riboso	13.742	125	0.418056	-1.25823	-	2	19.74531	21.00354
P62910	RPL32	60S riboso	15.86	135	0.416235	-1.26453	-	2	15.08385	16.34838
Q9Y5L4	TIMM13	Mitochondr	10.5	95	0.413167	-1.2752	-	2	14.1225	15.39771
O75251	NDUFS7	NADH dehy	23.564	213	0.408408	-1.29192	-	1	11.05643	12.34835
Q93034	CUL5	Cullin-5 OS	90.955	780	0.405407	-1.30256	-	1	10.70009	12.00265
P62263	RPS14	40S riboso	16.273	151	0.405238	-1.30316	-	2	18.7477	20.05086
O14880	MGST3	Microsoma	16.516	152	0.403653	-1.30881	-	2	14.82804	16.13685
Q07955	SRSF1	Serine/argi	27.745	248	0.396353	-1.33514	-	2	17.25174	18.58688
P61247	RPS3A	40S riboso	29.945	264	0.396306	-1.33531	-	2	18.41576	19.75107
Q14764	MVP	Major vault	99.327	893	0.395974	-1.33652	-	2	13.0396	14.37613
Q9HAS0	C17orf75	Protein Njn	44.622	396	0.394734	-1.34105	-	1	9.909668	11.25072
O75165	DNAJC13	DnaJ homo	254.415	2243	0.392324	-1.34988	-	1	10.03246	11.38234
Q9UPN3	MACF1	Microtubule	838.308	7388	0.391535	-1.35279	-	2	15.18003	16.53281
Q9H8M2	BRD9	Bromodom	67	597	0.39092	-1.35505	-	2	16.58726	17.94232
Q13428	TCOF1	Treacle pro	152.106	1488	0.390729	-1.35576	-	2	16.70104	18.0568
Q9HDC9	APMAP	Adipocyte p	46.48	416	0.388543	-1.36385	-	2	16.60289	17.96674
Q8N1F7	NUP93	Nuclear po	93.488	819	0.382827	-1.38524	-	2	15.47282	16.85805
Q14155	ARHGEF7	Rho guanin	90.012	803	0.382252	-1.3874	-	1	10.01151	11.39891
Q9H6Y2	WDR55	WD repeat	42.07	383	0.380456	-1.3942	-	1	10.94061	12.33481
Q9P2D1	CHD7	Chromodon	335.927	2997	0.377267	-1.40634	-	1	10.25927	11.66561
P46783	RPS10	40S riboso	18.898	165	0.376344	-1.40988	-	2	16.83096	18.24084
Q04837	SSBP1	Single-stra	17.26	148	0.373792	-1.41969	-	2	18.52352	19.94321
P62913	RPL11	60S riboso	20.252	178	0.371036	-1.43037	-	2	19.01933	20.4497
P15104	GLUL	Glutamine	42.064	373	0.366582	-1.44779	-	1	9.316825	10.76462
P62826	RAN	GTP-bindin	24.423	216	0.365928	-1.45037	-	2	16.84879	18.29916
Q9NV70	EXOC1	Exocyst co	101.982	894	0.36125	-1.46893	-	1	9.872567	11.3415
P15924	DSP	Desmoplak	331.774	2871	0.353222	-1.50135	-	2	15.06221	16.56357
Q9H2G2	SLK	STE20-like	142.695	1235	0.350516	-1.51245	-	2	15.01372	16.52616
Q9H425	C1orf198	Uncharacte	36.346	327	0.350405	-1.5129	-	2	13.18927	14.70217
Q96PK6	RBM14	RNA-bindin	69.492	669	0.347738	-1.52393	-	2	18.68157	20.2055
Q9HCY8	S100A14	Protein S10	11.662	104	0.346361	-1.52965	-	2	12.36318	13.89283
Q8IY81	FTSJ3	pre-rRNA 2	96.558	847	0.344999	-1.53534	-	2	16.20892	17.74426
Q9BRT8	CBWD1	COBW dor	44.068	395	0.342052	-1.54771	-	2	13.83832	15.38603
Q13442	PDAP1	28 kDa hea	20.63	181	0.336415	-1.57169	-	1	9.251743	10.82343
Q9NSI2	SLX9	Ribosome	25.456	230	0.333162	-1.5857	-	2	14.76648	16.35218
Q9Y6A4	CFAP20	Cilia- and fl	22.774	193	0.331739	-1.59188	-	2	13.8573	15.44918

Q86YT6	MIB1	E3 ubiquitir	110.136	1006	0.328254	-1.60711	-		2	20.81956	22.42668
Q9P260	RELCH	RAB11-bin	134.63	1216	0.325673	-1.61851	-		1	9.438979	11.05748
Q9BYD2	MRPL9	39S riboso	30.243	267	0.32469	-1.62287	-		2	13.66456	15.28742
P62280	RPS11	40S riboso	18.431	158	0.32241	-1.63303	-		2	17.48148	19.11452
P32969	RPL9	60S riboso	21.863	192	0.322197	-1.63399	-		2	17.44799	19.08197
P49006	MARCKSL	MARCKS-r	19.529	195	0.315217	-1.66558	-		2	15.96412	17.6297
P55786	NPEPPS	Puromycin-	103.276	919	0.312031	-1.68024	-		2	14.71258	16.39282
P62273	RPS29	40S riboso	6.677	56	0.306322	-1.70688	-		2	16.91193	18.61881
A8MT69	CENPX	Centromer	8.959	81	0.301665	-1.72898	-		1	9.110301	10.83928
Q8ND24	RNF214	RING finge	77.667	703	0.299697	-1.73843	-		1	10.58158	12.32
Q99880	H2BC13	Histone H2	13.952	126	0.298876	-1.74238	-		2	19.75132	21.4937
Q9UPT8	ZC3H4	Zinc finger	140.257	1303	0.294812	-1.76213	-		2	13.09997	14.8621
P62829	RPL23	60S riboso	14.865	140	0.292675	-1.77263	-		2	17.62357	19.3962
P81605	DCD	Dermcidin	11.284	110	0.288916	-1.79128	-		2	14.88021	16.67149
P62805	H4C1	Histone H4	11.367	103	0.285238	-1.80976	-		2	17.69561	19.50537
Q6P9B6	MEAK7	MTOR-ass	50.994	456	0.284686	-1.81255	-		1	9.345228	11.15778
Q9H9F9	ACTR5	Actin-relate	68.298	607	0.277535	-1.84926	-		1	10.00267	11.85193
O00623	PEX12	Peroxisom	40.797	359	0.277111	-1.85146	-		1	8.696168	10.54763
P35268	RPL22	60S riboso	14.787	128	0.274341	-1.86596	-		2	18.34906	20.21502
Q14157	UBAP2L	Ubiquitin-a	114.535	1087	0.273929	-1.86812	-		2	17.36709	19.23521
Q7Z406	MYH14	Myosin-14	227.871	1995	0.272409	-1.87615	-		2	19.05513	20.93128
P08574	CYC1	Cytochrom	35.422	325	0.271285	-1.88212	-		1	11.5616	13.44372
Q8IY37	DHX37	Probable A	129.545	1157	0.269638	-1.89091	-		2	12.73206	14.62297
P62899	RPL31	60S riboso	14.463	125	0.269634	-1.89092	-		2	19.00818	20.89911
L0R819	ASDURF	ASNSD1 u	11.25	96	0.267001	-1.90509	-		2	12.53734	14.44243
P55081	MFAP1	Microfibrilla	51.958	439	0.26557	-1.91284	-		2	13.6314	15.54424
Q86UK7	ZNF598	E3 ubiquitir	98.637	904	0.262056	-1.93206	-		1	9.63657	11.56863
Q02952	AKAP12	A-kinase ar	191.482	1782	0.262043	-1.93213	-		1	10.84227	12.7744
Q6NUM9	RETSAT	All-trans-re	66.82	610	0.261116	-1.93724	-		1	9.085526	11.02276
Q9NPJ8	NXT2	NTF2-relat	16.228	142	0.259065	-1.94861	-		1	11.42149	13.3701
Q92820	GGH	Gamma-glu	35.964	318	0.257354	-1.95817	-		1	8.807419	10.76559
P63173	RPL38	60S riboso	8.218	70	0.253474	-1.98009	-		2	17.65948	19.63957
P41208	CETN2	Centrin-2 C	19.738	172	0.251708	-1.99018	-		2	13.70704	15.69721
Q1ZZU3	SWI5	DNA repair	26.739	235	0.24945	-2.00318	-		1	10.5077	12.51087
P83731	RPL24	60S riboso	17.779	157	0.248526	-2.00853	-		2	17.16202	19.17055
P05141	SLC25A5	ADP/ATP t	32.852	298	0.247434	-2.01488	-		2	15.91784	17.93272
Q9NVZ3	NECAP2	Adaptin ea	28.339	263	0.24671	-2.01911	-		2	13.12152	15.14063
Q16629	SRSF7	Serine/argi	27.367	238	0.244206	-2.03383	-		2	15.43983	17.47366
O75150	RNF40	E3 ubiquitir	113.678	1001	0.241898	-2.04753	-		2	13.15433	15.20186
Q9BTA9	WAC	WW doma	70.724	647	0.241736	-2.04849	-		1	9.641564	11.69006
Q9BVP2	GNL3	Guanine nu	61.993	549	0.238298	-2.06916	-		2	14.45578	16.52494
O00743	PPP6C	Serine/thre	35.144	305	0.237024	-2.07689	-		2	13.83635	15.91324
Q13496	MTM1	Myotubular	69.932	603	0.236974	-2.0772	-		1	8.298063	10.37526
P02788	LTF	Lactotransf	78.182	710	0.235155	-2.08832	-		2	12.75374	14.84206
Q8NB16	MLKL	Mixed linea	54.479	471	0.230932	-2.11446	-		1	9.453374	11.56783
Q9H0R4	HDHD2	Haloacid de	28.536	259	0.228267	-2.13121	-		1	9.06851	11.19972
P35250	RFC2	Replication	39.157	354	0.225127	-2.15119	-		2	16.71008	18.86127
P00966	ASS1	Argininosu	46.53	412	0.224195	-2.15717	-		2	13.09276	15.24993
Q9BPW8	NIPSNAP1	Protein Nip	33.31	284	0.222818	-2.16606	-		1	10.33445	12.50052
Q8IWE2	FAM114A1	Protein NO	60.742	563	0.22202	-2.17124	-		1	9.571961	11.7432
O75376	NCOR1	Nuclear rec	270.21	2440	0.22123	-2.17638	-		1	9.188193	11.36457
P08708	RPS17	40S riboso	15.55	135	0.219416	-2.18826	-		2	19.74435	21.93261
P35610	SOAT1	Sterol O-ac	64.735	550	0.21872	-2.19284	-		1	9.211986	11.40483
Q5TEU4	NDUFAF5	Arginine-hy	38.918	345	0.217623	-2.2001	-		1	9.938448	12.13854
Q8N5H3	FAM89B	Leucine rep	20.147	189	0.215429	-2.21472	-		1	9.057207	11.27192
Q8TBC3	SHKBP1	SH3KBP1-	76.344	707	0.215383	-2.21502	-		2	14.80322	17.01824
Q9UEG4	ZNF629	Zinc finger	96.62	869	0.208673	-2.26069	-		1	10.08799	12.34868
P13667	PDIA4	Protein dis	72.932	645	0.207529	-2.26861	-		2	11.63517	13.90379
P13987	CD59	CD59 glyco	14.177	128	0.206597	-2.27511	-		2	14.90558	17.18069

Q13671	RIN1	Ras and Ra	84.099	783	0.20441	-2.29046	-	1	9.238214	11.52867
O00488	ZNF593	Zinc finger	15.199	134	0.202902	-2.30115	-	2	15.25949	17.56063
Q9UNQ2	DIMT1	Probable d	35.236	313	0.202567	-2.30353	-	2	13.89614	16.19967
P18583	SON	Protein SO	263.83	2426	0.20164	-2.31015	-	2	13.52992	15.84006
P14314	PRKCSH	Glucosidas	59.425	528	0.200812	-2.31608	-	1	10.32351	12.63959
P54578	USP14	Ubiquitin ca	56.069	494	0.200543	-2.31802	-	1	9.750053	12.06807
Q9P2J5	LARS1	Leucine--tF	134.466	1176	0.190053	-2.39553	-	2	13.95819	16.35372
P47914	RPL29	60S riboso	17.752	159	0.183334	-2.44745	-	2	18.10789	20.55534
Q9Y618	NCOR2	Nuclear rec	273.657	2514	0.183247	-2.44814	-	2	17.01781	19.46595
P62701	RPS4X	40S riboso	29.598	263	0.182094	-2.45725	-	2	18.97043	21.42767
P18621	RPL17	60S riboso	21.397	184	0.180903	-2.46671	-	2	18.86293	21.32965
Q96HS1	PGAM5	Serine/thre	32.004	289	0.173652	-2.52573	-	2	16.69629	19.22202
P62841	RPS15	40S riboso	17.04	145	0.172717	-2.53352	-	2	20.0478	22.58132
Q9BYG3	NIFK	MKI67 FHA	34.222	293	0.171359	-2.54491	-	2	14.45738	17.00229
P34897	SHMT2	Serine hydr	55.993	504	0.170893	-2.54883	-	2	13.88054	16.42937
Q9BRT6	LLPH	Protein LLF	15.225	129	0.170712	-2.55037	-	2	16.88774	19.43811
P17812	CTPS1	CTP syntha	66.69	591	0.163747	-2.61046	-	2	15.4514	18.06187
Q8TEM1	NUP210	Nuclear po	205.111	1887	0.161635	-2.62919	-	2	11.6591	14.28829
Q01469	FABP5	Fatty acid-l	15.164	135	0.155517	-2.68486	-	2	12.97266	15.65751
Q9UNX3	RPL26L1	60S riboso	17.256	145	0.154704	-2.69242	-	2	17.86731	20.55974
Q9UGY1	NOL12	Nucleolar p	24.663	213	0.154485	-2.69446	-	2	14.93281	17.62728
Q9UHX1	PUF60	Poly(U)-bin	59.875	559	0.152362	-2.71443	-	2	17.0281	19.74253
P62277	RPS13	40S riboso	17.222	151	0.151248	-2.72501	-	2	18.7123	21.43731
P07737	PFN1	Profilin-1 C	15.054	140	0.148056	-2.75579	-	2	14.39814	17.15393
P62861	FAU	FAU ubiqui	14.39	133	0.144564	-2.79022	-	2	21.74049	24.53071
P02794	FTH1	Ferritin hea	21.226	183	0.143981	-2.79605	-	2	14.0438	16.83984
O14828	SCAMP3	Secretory c	38.287	347	0.139735	-2.83923	-	2	14.03368	16.87292
Q9GZZ8	LACRT	Extracellula	14.246	138	0.133566	-2.90438	-	2	13.20114	16.10552
Q9Y5L0	TNPO3	Transportir	104.204	923	0.132542	-2.91547	-	2	12.00734	14.92282
Q03001	DST	Dystonin O	860.662	7570	0.132219	-2.919	-	2	22.03369	24.95269
Q9BQ48	MRPL34	39S riboso	10.165	92	0.129866	-2.9449	-	2	14.67071	17.61562
E9PRG8	C11orf98	Uncharacte	14.234	123	0.128845	-2.95629	-	2	16.94054	19.89683
P02647	APOA1	Apolipoprot	30.778	267	0.128443	-2.9608	-	2	12.59157	15.55237
P62266	RPS23	40S riboso	15.808	143	0.127932	-2.96656	-	2	18.12096	21.08751
Q9H5V9	STEEP1	STING ER	25.625	222	0.12203	-3.0347	-	2	13.95201	16.98671
Q15517	CDSN	Corneodes	51.522	529	0.121994	-3.03511	-	1	9.600359	12.63547
Q9UMY1	NOL7	Nucleolar p	29.426	257	0.116373	-3.10318	-	2	14.59799	17.70117
P62847	RPS24	40S riboso	15.423	133	0.115025	-3.11998	-	2	17.99269	21.11267
P62750	RPL23A	60S riboso	17.695	156	0.112921	-3.14661	-	2	19.17268	22.31929
Q96T76	MMS19	MMS19 nu	113.29	1030	0.111797	-3.16105	-	1	7.541406	10.70245
P62081	RPS7	40S riboso	22.127	194	0.110516	-3.17767	-	2	19.35756	22.53524
Q9BY44	EIF2A	Eukaryotic	64.99	585	0.110188	-3.18195	-	2	10.71759	13.89955
Q8WXF7	ATL1	Atlastin-1 C	63.544	558	0.109679	-3.18865	-	2	13.67353	16.86218
P06702	S100A9	Protein S1(	13.242	114	0.106057	-3.23709	-	2	14.03557	17.27267
Q08378	GOLGA3	Golgin sub	167.355	1498	0.105321	-3.24714	-	2	15.0702	18.31734
P16401	H1-5	Histone H1	22.58	226	0.104599	-3.25705	-	2	18.56815	21.8252
P0C0L4	C4A	Compleme	192.785	1744	0.103952	-3.26601	-	2	11.8922	15.15822
Q03135	CAV1	Caveolin-1	20.472	178	0.100993	-3.30768	-	2	16.50642	19.81409
Q9H6T3	RPAP3	RNA polym	75.719	665	0.100593	-3.3134	-	2	16.81541	20.1288
P07305	H1-0	Histone H1	20.863	194	0.099503	-3.32912	-	2	17.93226	21.26138
P10412	H1-4	Histone H1	21.865	219	0.097948	-3.35185	-	2	19.37428	22.72613
Q9Y2Z0	SUGT1	Protein SG	41.024	365	0.095716	-3.3851	-	1	8.307337	11.69244
Q9NWU5	MRPL22	39S riboso	23.641	206	0.094983	-3.39619	-	2	13.52589	16.92207
P20226	TBP	TATA-box-	37.698	339	0.094889	-3.39762	-	2	12.7076	16.10521
P62269	RPS18	40S riboso	17.719	152	0.091887	-3.44399	-	2	21.10258	24.54657
P53999	SUB1	Activated F	14.395	127	0.090514	-3.46572	-	2	12.43936	15.90508
O95232	LUC7L3	Luc7-like p	51.466	432	0.088902	-3.49165	-	2	13.68486	17.1765
P19474	TRIM21	E3 ubiquitir	54.17	475	0.088831	-3.4928	-	2	14.72664	18.21944
P46776	RPL27A	60S riboso	16.561	148	0.087408	-3.5161	-	2	17.92329	21.43939

Q6NT76	HMBOX1	Homeobox	47.278	420	0.085941	-3.54051	-	2	11.68681	15.22732
Q9NR28	DIABLO	Diablo IAP-	27.131	239	0.085215	-3.55275	-	1	9.952938	13.50569
P28799	GRN	Progranulin	63.544	593	0.084969	-3.55692	-	2	15.37534	18.93226
Q7Z3Z3	PIWIL3	Piwi-like pr	101.089	882	0.081859	-3.61071	-	2	15.73447	19.34518
A0A0C4DH	IGKV3D-7	Immunoglo	13.148	119	0.079867	-3.64626	-	2	14.97652	18.62278
O00471	EXOC5	Exocyst co	81.853	708	0.079306	-3.65642	-	2	17.82978	21.48621
Q9Y597	KCTD3	BTB/POZ d	88.984	815	0.079053	-3.66104	-	2	12.66742	16.32846
Q6ZUT1	NKAPD1	Uncharacte	34.11	292	0.078945	-3.663	-	2	12.7453	16.4083
P42766	RPL35	60S riboso	14.551	123	0.077597	-3.68786	-	2	18.83895	22.52681
O94913	PCF11	Pre-mRNA	173.05	1555	0.077391	-3.69169	-	1	8.799767	12.49145
P61513	RPL37A	60S riboso	10.275	92	0.07596	-3.71862	-	2	18.56774	22.28636
P25311	AZGP1	Zinc-alpha-	34.259	298	0.07586	-3.72052	-	1	10.30601	14.02652
Q08554	DSC1	Desmocolli	99.987	894	0.075811	-3.72146	-	2	10.40525	14.1267
Q6NZI2	CAVIN1	Caveolae-a	43.476	390	0.074666	-3.74341	-	2	17.0202	20.76361
O94808	GFPT2	Glutamine-	76.931	682	0.072237	-3.79111	-	2	12.96544	16.75656
Q7Z6E9	RBBP6	E3 ubiquitin	201.564	1792	0.060613	-4.04423	-	2	13.0179	17.06213
Q14331	FRG1	Protein FR	29.172	258	0.060502	-4.04687	-	2	11.94808	15.99495
Q9UQ35	SRRM2	Serine/argi	299.615	2752	0.058917	-4.08517	-	2	16.24934	20.33451
P05109	S100A8	Protein S10	10.835	93	0.056505	-4.14549	-	2	12.49625	16.64174
P0DOX7		Immunoglo	23.379	214	0.056051	-4.15713	-	2	14.65374	18.81087
Q66PJ3	ARL6IP4	ADP-ribosy	26.375	237	0.055179	-4.17974	-	2	11.14408	15.32383
Q14790	CASP8	Caspase-8	55.391	479	0.054693	-4.19251	-	1	8.43016	12.62267
P54886	ALDH18A1	Delta-1-pyr	87.302	795	0.05415	-4.20688	-	2	16.29738	20.50426
P0DOX5		Immunoglo	49.329	449	0.054139	-4.20719	-	2	22.29611	26.50331
Q14687	GSE1	Genetic su	136.164	1217	0.053926	-4.21288	-	2	11.94628	16.15916
P07942	LAMB1	Laminin su	198.038	1786	0.053688	-4.21925	-	2	23.10824	27.32749
Q7L014	DDX46	Probable A	117.362	1031	0.051018	-4.29285	-	2	13.57802	17.87087
Q8IYB3	SRRM1	Serine/argi	102.335	904	0.049215	-4.34476	-	2	13.36673	17.71149
Q8TER5	ARHGEF4	Rho guanin	164.658	1519	0.046052	-4.44058	-	1	7.915103	12.35569
Q01085	TIAL1	Nucleolysin	41.591	375	0.045468	-4.45901	-	2	17.26279	21.7218
Q9NZC2	TREM2	Triggering	25.447	230	0.043255	-4.531	-	2	18.12354	22.65454
Q8NEM2	SHCBP1	SHC SH2 d	75.69	672	0.041515	-4.59024	-	2	11.61687	16.20711
O75344	FKBP6	Inactive pe	37.214	327	0.040848	-4.61358	-	2	14.5094	19.12298
Q9Y383	LUC7L2	Putative RI	46.514	392	0.039993	-4.64412	-	2	17.24098	21.88509
Q9HD33	MRPL47	39S riboso	29.45	250	0.037993	-4.71811	-	2	12.93347	17.65158
Q9NQ29	LUC7L	Putative RI	43.728	371	0.037449	-4.73891	-	2	12.28887	17.02778
O75394	MRPL33	39S riboso	7.619	65	0.037384	-4.74142	-	1	9.99609	14.73751
P31025	LCN1	Lipocalin-1	19.25	176	0.036998	-4.75641	-	2	12.44644	17.20285
Q02413	DSG1	Desmoglein	113.748	1049	0.035589	-4.81242	-	1	10.02802	14.84043
O15042	U2SURP	U2 snRNP-	118.292	1029	0.034975	-4.83755	-	2	10.20958	15.04712
Q9BXU1	STK31	Serine/thre	115.694	1019	0.034699	-4.84898	-	2	19.68197	24.53095
Q9BQC6	MRPL57	Ribosomal	12.266	102	0.034487	-4.85781	-	1	9.283897	14.14171
Q969G5	CAVIN3	Caveolae-a	27.701	261	0.031856	-4.97229	-	2	13.75249	18.72477
P62979	RPS27A	Ubiquitin-4	17.965	156	0.03095	-5.0139	-	2	19.19051	24.20441
P35030	PRSS3	Trypsin-3 (	32.529	304	0.030947	-5.01408	-	2	16.71089	21.72497
P31350	RRM2	Ribonucleo	44.878	389	0.03048	-5.03598	-	2	15.18743	20.22341
Q9NWB6	ARGLU1	Arginine ar	33.216	273	0.03035	-5.04217	-	2	16.41812	21.46029
Q6TDU7	DNAI7	Dynein axo	83.16	716	0.030145	-5.05192	-	2	17.00163	22.05355
P0DOX8		Immunoglo	22.83	216	0.027965	-5.16026	-	2	14.03377	19.19403
Q14498	RBM39	RNA-bindin	59.38	530	0.027501	-5.18439	-	2	15.48771	20.67211
Q15043	SLC39A14	Metal catio	54.212	492	0.025146	-5.31354	-	1	10.36385	15.67739
Q9Y691	KCNMB2	Calcium-ac	27.13	235	0.023985	-5.38174	-	1	9.400132	14.78187
P01023	A2M	Alpha-2-ma	163.291	1474	0.022442	-5.47763	-	2	10.55651	16.03413
Q8WVK2	SNRNP27	U4/U6.U5 s	18.86	155	0.021516	-5.53846	-	1	9.738934	15.2774
P12273	PIP	Prolactin-in	16.572	146	0.019798	-5.65854	-	2	10.67649	16.33502
Q7RTY7	OVCH1	Ovochyma	125.066	1134	0.016015	-5.96446	-	2	14.40913	20.37358
Q9NP64	ZCCHC17	Nucleolar p	27.57	241	0.015251	-6.035	-	2	12.3573	18.3923
P01859	IGHG2	Immunoglo	35.901	326	0.015081	-6.05115	-	2	8.708842	14.75999
Q13523	PRPF4B	Serine/thre	116.987	1007	0.012527	-6.31877	-	2	10.15861	16.47738

Q9NPA3	MID1IP1	Mid1-intera	20.202	183	0.010023	-6.6406	-	2	9.489266	16.12987
P05423	POLR3D	DNA-direct	44.396	398	0.009473	-6.72199	-	2	12.27336	18.99535
Q8TA86	RP9	Retinitis pig	26.107	221	0.008989	-6.79763	-	2	11.10944	17.90707
P16403	H1-2	Histone H1	21.365	213	0.007717	-7.01779	-	2	12.39607	19.41386
Q96IV6	FAXDC2	Fatty acid h	39.002	333	0.004502	-7.79531	-	2	15.4962	23.29151
Q96P48	ARAP1	Arf-GAP wi	162.192	1450	0.004493	-7.79824	-	1	9.92617	17.72441
P0DP09	IGKV1-13	Immunoglo	12.569	117	0.004276	-7.86954	-	1	10.47124	18.34078
Q05519	SRSF11	Serine/argi	53.542	484	0.003654	-8.09627	-	2	10.14364	18.23991
Q08752	PPID	Peptidyl-pro	40.764	370	0.003456	-8.17655	-	2	11.64552	19.82207
P61626	LYZ	Lysozyme (	16.537	148	0.002826	-8.4671	-	1	9.564987	18.03209
P34896	SHMT1	Serine hydr	53.083	483	0.002375	-8.71756	-	2	9.282532	18.00009
Q86XI8	ZSWIM9	Uncharacte	70.073	627	0.001214	-9.68599	-	1	9.959576	19.64556
Q9HCU9	BRMS1	Breast canc	28.461	246	0.001154	-9.75946	-	1	9.493325	19.25278
Q9NRY4	ARHGAP3	Rho GTPas	170.514	1499	0.001004	-9.96058	-	1	8.936323	18.89691
P01594	IGKV1-33	Immunoglo	12.848	117	0.000354	-11.4656	-	1	8.60859	20.07418
P01619	IGKV3-20	Immunoglo	12.557	116	0.000267	-11.8729	-	1	9.57946	21.45232
